# Palladium (II)–Salan Complexes as Catalysts for Suzuki–Miyaura C–C Cross-Coupling in Water and Air. Effect of the Various Bridging Units within the Diamine Moieties on the Catalytic Performance

**DOI:** 10.3390/molecules25173993

**Published:** 2020-09-02

**Authors:** Szilvia Bunda, Krisztina Voronova, Ágnes Kathó, Antal Udvardy, Ferenc Joó

**Affiliations:** 1 Department of Physical Chemistry, University of Debrecen, P.O. Box 400, H-4002 Debrecen, Hungary; bunda.szilvia@science.unideb.hu (S.B.); katho.agnes@science.unideb.hu (Á.K.); 2 Doctoral School of Chemistry, University of Debrecen, P.O. Box 400, H-4002 Debrecen, Hungary; 3 Department of Chemistry, University of Nevada, Reno, Reno, NV 89557, USA; kvoronova@unr.edu; 4 MTA-DE Redox and Homogeneous Catalytic Reaction Mechanisms Research Group, P.O. Box 400, H-4002 Debrecen, Hungary

**Keywords:** catalysis in water, C–C cross-coupling, Suzuki–Miyaura reaction, palladium, sulfonated salan

## Abstract

Water-soluble salan ligands were synthesized by hydrogenation and subsequent sulfonation of salens (*N*,*N*’-*bis*(slicylidene)ethylenediamine and analogues) with various bridging units (linkers) connecting the nitrogen atoms. Pd (II) complexes were obtained in reactions of sulfosalans and [PdCl_4_]^2−^. Characterization of the ligands and complexes included extensive X-ray diffraction studies, too. The Pd (II) complexes proved highly active catalysts of the Suzuki–Miyaura reaction of aryl halides and arylboronic acid derivatives at 80 °C in water and air. A comparative study of the Pd (II)–sulfosalan catalysts showed that the catalytic activity largely increased with increasing linker length and with increasing steric congestion around the N donor atoms of the ligands; the highest specific activity was 40,000 (mol substrate) (mol catalyst × h)^−1^. The substrate scope was explored with the use of the two most active catalysts, containing 1,4-butylene and 1,2-diphenylethylene linkers, respectively.

## 1. Introduction

Salen (*N*,*N*’-*bis*(salicylaldiminato)-1,2-diaminoethane) and its derivatives, which can be easily obtained by condensation of salicylaldehyde and ethylendiamine or their various substituted analogues, have played prominent roles as ligands in coordination chemistry and catalysis throughout the years [1,2,3,4,5]. Salan (*N*,*N*’-*bis*(*o*-hydroxybenzyl)-1,2-diaminoethane) is the tetrahydro derivative of salen, usually obtained from the latter by reduction with NaBH_4_ [1,6,7,8,9]; however, direct synthesis via Mannich reaction is also known [10]. Salan has become a general name for analogous *N*,*N*’-*bis*(*o*-hydroxybenzyl)-α,ω-diaminoalkanes, too, which may have diverse linker groups between nitrogen atoms and/or variously substituted *o*-hydroxybenzyl moieties. As secondary amines, salans are much less vulnerable to hydrolysis than their diimine parent compounds, and for this reason, they are more suitable for applications in aqueous media [11,12]. Transition metal complexes of salans have earned important applications in catalysis of various reactions such as polymerization [13,14], sulfoxidation [15], oxygen transfer [9], fluorination and hydroxylation [16], to name a few. The promising biomedical and catalytic properties and applications of salan complexes have been reviewed recently [1].

Carbon–carbon cross-coupling reactions are of fundamental importance in organic synthesis as shown by the high number of publications (413 for the Suzuki–Miyaura reaction in 2019 (Scopus, Elsevier)) and can be conveniently practiced in fully organic media [17,18,19]. On the other hand, health and environmental safety requires the elimination of organic solvents from chemical processes as much as possible. A viable alternative to the use of organic solvents is the application of water as the reaction medium [20,21,22]. Organometallic catalysis in aqueous systems has great potential for green chemistry, and this approach has been extended to the field of C–C cross-couplings, too [23,24,25,26,27,28,29]. Not only the replacement of volatile and harmful organic solvents but also improved process characteristics (fire safety, catalyst recycling, etc.) and product quality are attractive features of aqueous procedures.

In homogeneously catalysed aqueous/organic biphasic reactions, such as the Pd-catalysed cross-coupling of aryl halides and arylboronic acids, the catalyst should be preferentially soluble in water. Hydrophilic palladacycles [30], complexes of tertiary phosphines [23,31,32], N-heterocyclic carbenes [33,34,35] and water-soluble complexes with salen ligands [2,36,37] have already been applied as catalysts in aqueous C–C cross-couplings. Alternatively, the reactants and the catalyst have to be incorporated into micelles formed by appropriate surfactants within the bulk aqueous phase [38,39,40,41,42]. Both methods allowed the design of outstandingly productive and robust catalytic procedures.

We have been interested in aqueous organometallic catalysis for several years [21] and employed as catalysts complexes of transition metals with water-soluble tertiary phosphine and/or N-heterocyclic carbene ligands. Recently, we launched a program to study in aqueous media the catalytic properties of sulfonated salan-based complexes in reactions such as hydrogenation of alkenes and ketones [43], redox isomerization of allylic alcohols [44,45] and carbon–carbon cross-coupling reactions [46]. In particular, some Pd (II)–salan complexes were found to be highly effective catalysts for the Sonogashira and the Suzuki–Miyaura cross-coupling reactions [46,47].

**Scheme 1:**
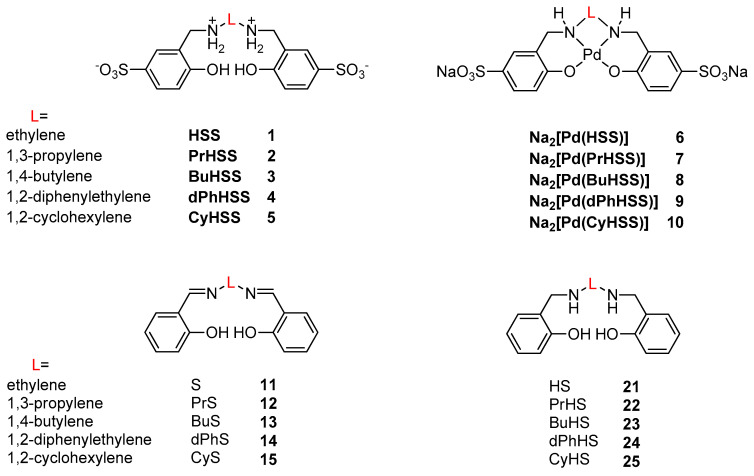
Salan ligands (hydrogenated sulfonated salens, **1**–**5**) and their Pd (II) complexes (**6**–**10)** used in this study, together with the intermediates of their synthesis (salens **11**–**15** and hydrogenated salens **21**–**25**): ligands **1**–**5** were isolated as zwitterions, and complexes **6**–**10** were isolated as Na salts.

In contrast to what may be suggested by the simplified formulae in Scheme 1, the structure of even the simplest sulfosalan, HSS (1), deviates from planarity and the free rotation around the C–N bonds gives high flexibility to the ligands in coordination to a metal ion. This flexibility is largely influenced by the length of the bridging unit between the secondary amine nitrogens (e.g., C_2_ vs C_4_ alkyl chains). The structure, rigidity and steric requirements of the linker unit (e.g., ethyl, *cis*- or *trans*-1,2-cyclohexyl, 1,2-diphenylethyl linkers) similarly may have large effects on the coordination ability of the sulfosalan ligands, which may be manifested also in the catalytic properties of the resulting complexes. During our studies, we noted important differences in the catalytic activities of Pd (II)–sulfosalan complexes; therefore, we decided to perform a comparative study of a reasonably large series of such complexes. In this paper, we present the results of a comparative study of the catalytic performance of complexes **6**–**10** (Scheme 1) in Suzuki–Miyaura cross-coupling reactions. For the purpose of these studies, we synthesized the new ligands **4**, **5b** and **5c** and the new complexes Na_2_[Pd(PrHSS)] (**7**), Na_2_[Pd(dPhHSS)] (**9**), Na_2_[Pd(*trans*-CyHSS)] (**10b**) and Na_2_[Pd(*cis*-CyHSS)] (**10c**). To gain more insight into the structural features of the sulfosalan ligands and their Pd (II)–complexes, all sulfosalan ligands, **1**–**5,** as well as complexes **6** and **7** were studied in detail by single crystal X-ray diffraction (SC-XRD) (**1** and **3** by powder X-ray diffraction, too).

## 2. Results and Discussion

### 2.1. Synthesis

**Figure 1:**
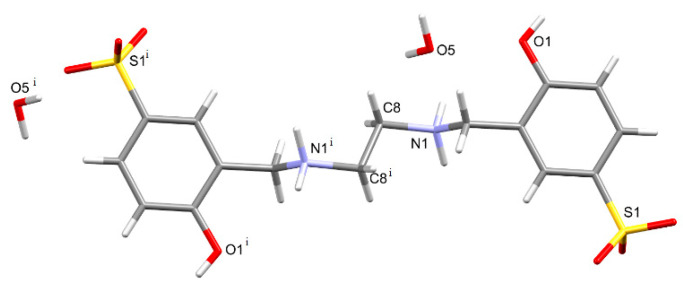
Capped sticks representations of **1** × 2H_2_O. Symmetry code: (i) –x, 1–y, –z.

The new ligands, **4**, **5b** and **5c**, and the Pd (II) complexes **7**, **9**, **10b** and **10c**, were synthesized according to the procedure used by us earlier for the rest of the compounds, **1**–**3**, **6**, **8** and **10a** [44,45,46,47]. Briefly, the starting salens were obtained by condensation of salicylaldehyde and the appropriate diamine, and the latter were reduced to the hydrogenated salens with four equivalents of NaBH_4_ in methanol. The white hydrogenated salen products were sulfonated in an ice-cold 4:1 mixture of fuming sulfuric acid (20%) and concentrated (96%) sulfuric acid. Addition of the reaction mixtures to cold water and adjustment of the pH to 4 led to formation of white precipitates of the salan ligands (Figure 1).

Na_2_[Pd(PrHSS)] (**7**), Na_2_[Pd(dPhHSS)] (**9**) and Na_2_[Pd(CyHSS)] (**10**) were prepared from equivalent amounts of the sulfosalan ligand and (NH_4_)_2_[PdCl_4_] in aqueous solutions adjusted to pH 7.5 with concentrated NaOH solution and kept at 60 °C for 10 h. The yellow complexes were precipitated from the cooled reaction mixtures with the addition of ice-cold ethanol.

All compounds showed the characteristic A_1_ sulfonate stretching frequency in the infrared spectrum within the 1029.0–1033.4 cm^−1^ range and displayed the expected ^1^H and ^13^C-NMR signals, as well as the correct electrospray ionization (ESI) MS molecular ion peaks. Data are given in the Materials and Methods section, and the ^1^H and ^13^C{^1^H} NMR spectra are collected in the Supplementary Material.

### 2.2. Crystallographic Characterization of Sulfonated Salan Ligands 1–5 and Palladium (II) Complexes of PrHSS (***7***) and BuHSS (***8***)

#### 2.2.1. Sulfonated salan ligands **1**–**5**

Although complexes of sulfonated salens and non-sulfonated salans have been used already as homogeneous catalysts, the water-soluble Pd (II) complexes of sulfonated salans were first synthesized and applied in our laboratory to catalyse C–C cross-coupling reactions in water. Ligands **1**–**5** were obtained by an improved method consisting of sulfonation of the diamine precursors **21**–**25**, and Pd (II) complexes **6**–**10** were synthesized in reactions of the ligands with (NH_4_)_2_[PdCl_4_]. The compounds obtained in this work have not been characterized earlier by SC-XRD despite the considerable structural differences that can be expected between the complexes depending on the nature and size of the bridging unit of their sulfosalan ligand. For this reason, we undertook a structural study of the ligands and complexes available in the form of crystals suitable for X-ray diffraction measurements. Luckily, good quality crystals could be grown from water in the cases of **1** × 2H_2_O, PrHSS (**2**), BuHSS (**3**), (±)-*trans*-CyHSS (**5b**), **5ca** and **5cb**. Unfortunately, we could not obtain crystals of dPhHSS (**4**) from water and this latter compound was crystallized from wet dimethylsulfoxide (DMSO). Na_2_[Pd(PrHSS)] (**7**) and Na_2_[Pd(BuHSS)] (**8**) were dissolved in 1M KOH solution layered by 2-propanol. All efforts to grow crystals of **6**, **9** and **10** remained so far unsuccessful.

Full details of the crystallographic results are outside the scope of this manuscript but are amply described in the Supplementary Material. Nevertheless, a few basic findings are mentioned below.

**Figure 2:**
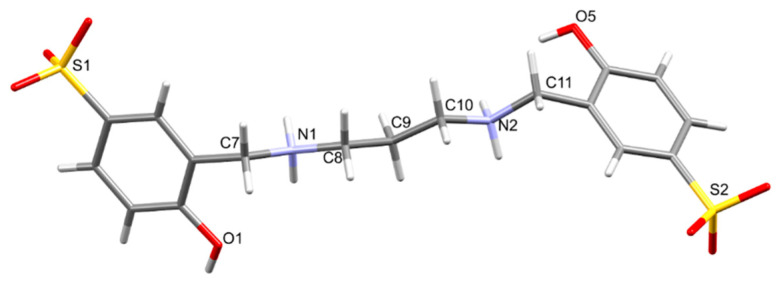
Capped sticks representation of **2** × 5.5H_2_O. Lattice water molecules are omitted for clarity.

**Figure 3:**
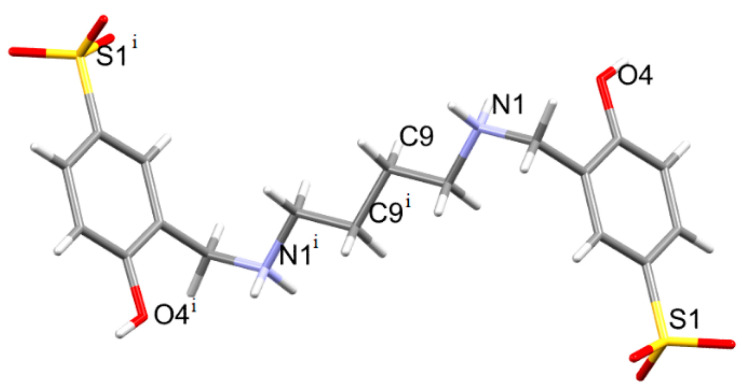
Capped sticks representation of **3**. Symmetry code: (i) –x, 1–y, –z; *Z*’ = 0.5.

**Figure 4:**
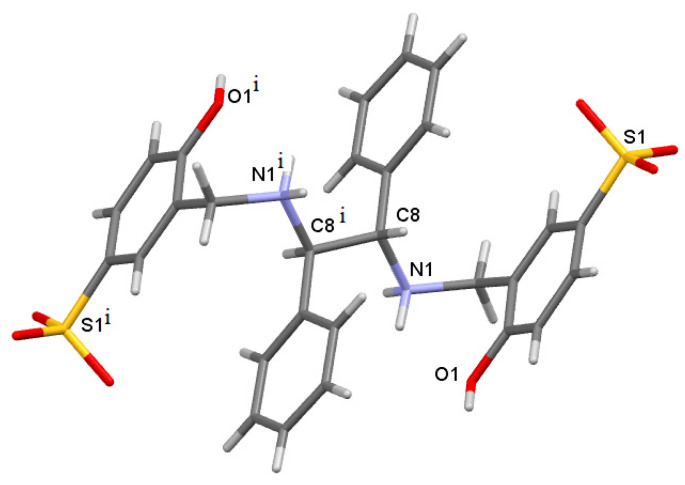
Capped sticks representation of **4** × H_2_O × DMSO. Solvents molecules are omitted for clarity. Symmetry code: (i) 1–x, 1–y, 1–z.

**Figure 5:**
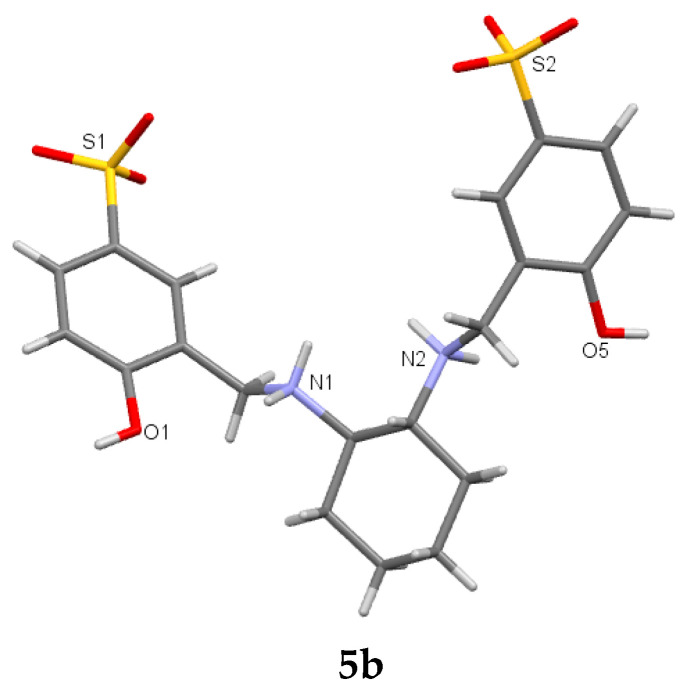

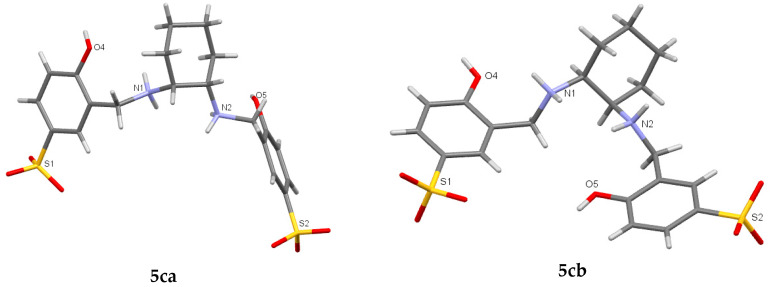
Structures of (±)-*trans-*CyHSS × 7H_2_O (**5b**; P1), *cis*-CyHSS × 2H_2_O (**5ca**; P2_1_/c) and *cis*-CyHSS × 6H_2_O (**5cb**; C2/c). Water molecules are omitted for clarity.

Scarcely any similar compounds have been reported that could be compared to our new structures. However, in such cases, a great degree of similarity is found. For example, the major difference in the bond distances of **1** × 2H_2_O (Figure 1) and its already known solvomorph [44], **1** × DMSO, is in the C8–C8^(i)^ bond length (1.529(11) Å vs. 1.495 Å). The starting compound for the synthesis of PrHSS (**2**), i.e., *N*,*N*’-*bis*(2-hydroxybenzyl)-1,3-diaminopropane, PrHS, was previously crystallized with various aromatic polycarboxylates [48] and SC-XRD studies revealed the protonation of the secondary amine groups of PrHS, similar to the case of PrHSS (**2**) (Figure 2). Comparison of the structure of n-K_4_[μ_8_-BuHSS][μ_2_-H_2_O]_4_[H_2_O]_6_ published by us earlier [46] to the one of **3** in this study (Figure 3), shows, that the N1–C7–C1 angles are almost the same (114.28° and 114.4°) in the two molecules, and only the positions of the aromatic groups are different (Figure S15). Superposition of the structures of the salan ligand, *meso* (RS,SR)-*N,N*’-*bis*(2-hydroxybenzyl)-1,2-diphenyl-1,2-diaminoethane [49] and its sulfonated product, dPhHSS (**4**) (Figure 4) also shows high degree of similarity (Figure S20) and proves that the starting salen underwent hydrogenation as well as sulfonation in the *p-*position relative to the phenolic oxygen. The major difference between the structures of **5b** (Figure 5) and its starting material for synthesis, i.e., (±)-*trans*-CyS [50] is in the position of the aromatic rings (Figure S23). Perhaps the most important information is that, during the synthesis of *cis*-CyHSS × 2H_2_O (**5ca**) (Figure 5), the *cis*-conformation in the Schiff base formed in the reaction of salicylaldehyde and *cis*-1,2-diaminocyclohexane is retained throughout hydrogenation and sulfonation. An interesting observation is that, when a racemic mixture of *cis*-CyHSS and *trans*-CyHSS was subjected to crystallization from water, the procedure yielded only crystals of *cis*-CyHSS (**5cb**) (Figure 5). The cyclohexyl ring of the sulfonated product *cis*-CyHSS overlaps precisely with the cyclohexyl ring in *N,N’*-di-5-nitrosalicylidene-(*R,S*)-l,2-cyclohexanediamine, published by Desiraju et al. [51] (see superposition of the molecules, Figure S27).

Powder diffraction patterns of **1** × 2H_2_O and **3** were calculated from the cell parameters of the crystals obtained from water and the ones measured experimentally on the powdery products yielded by the synthesis; a good agreement was found with the experimentally determined diffractograms (Figures S5 and S16). This shows that the direct products of syntheses and the crystals grown from water have the same composition.

It is the general characteristics of the crystals of **1**–**5** that they contain various numbers of solvent molecules, in most cases water. Due to the large number of water molecules and to the presence of O- and N-atoms in the ligands, strong hydrogen bonds are formed within the lattices. In addition to the hydrogen bonds, the crystal architecture is also stabilized by the π−π interactions between the aromatic rings. Quantitative details are included in Tables S1–S7 and shown on the relevant crystal packing diagrams of **1**–**5** in Supplementary Material.

#### 2.2.2. Palladium (II) Complexes of PrHSS (**7**) and BuHSS (**8**)

Crystals of K_2_[Pd(PrHSS)] (**7′**) K_2_[Pd(BuHSS)] (**8′**) were obtained from solutions of Na_2_[Pd(PrHSS)] (**7**) and Na_2_[Pd(BuHSS)] (**8**) in 1M KOH solution layered by 2-propanol and were subjected to SC-XRD measurements at 5 °C. The packing diagrams of the two complexes reveal that the complexes are placed within the lattice in layers and that the sulfosalan complexes are held together by inorganic polymer chains (Figures S32–S35). In the case of both complexes, the 2D structures are shaped by the electrostatic and van der Waals interactions between the K^+^ ions and the O-atoms of the sulfonate groups of the ligand and water molecules, together with the hydrogen bonds within the lattice. Similar polymeric chains were detected by us in crystals of the n-K_4_[μ_8_-BuHSS][μ_2_-H_2_O]_4_[H_2_O]_6_ sulfosalan [46] and in the cases of Ni(II) and Cu(II) complexes of *bis*(salicylidene)-1,2-diaminocyclohexane, CyS [52].

**Figure 6:**
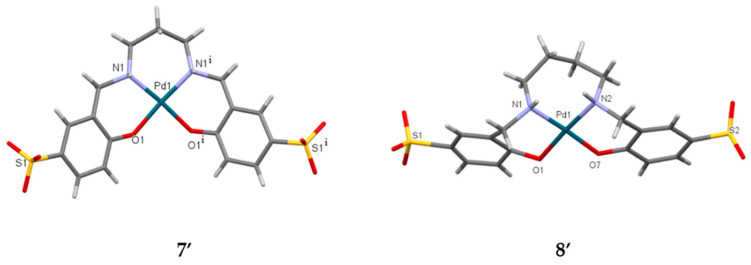
Capped sticks views of K_2_[Pd(PrHSS)] (**7′**). Symmetry code: (i) +x, 1/2–y, +z and K_2_[Pd(BuHSS)] (**8′**). Solvents and the flexible polymer chains linked together by K^+^ and water molecules are omitted for clarity.

Diffraction measurements were made on several crystals of both complexes at 150 K and at room temperature. Since the crystals were twinned and the polymer chains were flexible, despite all our efforts, all *R* values were higher than 10%, together with *wR2*-s > 25%. Due to these errors, the bond lengths and angles determined for the complexes are not suitable for discussion. Nevertheless, the SC-XRD measurements yielded clear atomic connectivities in both cases (Figure 6) and, together with the spectroscopic data, prove the structures of the complexes. These are the first solid state structures obtained for Pd (II)–sulfosalan complexes that, despite all uncertainties, show clearly the steric differences imposed by C3 and C4 bridging alkyl chains in Pd (II)–sulfosalan complexes.

### 2.3. Catalytic Properties of the Pd(II)–Sulfosalan Complexes in Suzuki–Miyaura Cross-Coupling Reactions

Earlier, we have established that some of the Pd (II)-sulfonated salan complexes were active catalysts for the Suzuki–Miyaura cross-coupling reactions in aqueous media. The reactions could be performed under aerobic conditions, and the catalysts showed outstanding stability in aqueous solutions. One of the aims of the present study was the comparison of catalytic properties of Pd (II)-sulfonated salan complexes with various linker groups, L, in the Suzuki–Miyaura cross-coupling and the exploration of the usefulness of the best catalysts for the reactions of a wide range of substrates under various conditions. For this purpose, in addition to the already known sulfosalans, we synthesized new ligands of such types starting with *cis*- and *trans*-isomers of 1,2-cyclohexanediamine and developed synthetic procedures for **7** and **9**, too.

**Figure 7:**
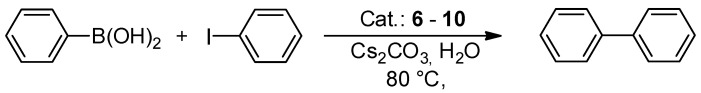
Suzuki–Miyaura cross-coupling of iodobenzene and phenylboronic acid catalysed by Pd (II)–sulfosalan complexes in water.

**Figure 8:**
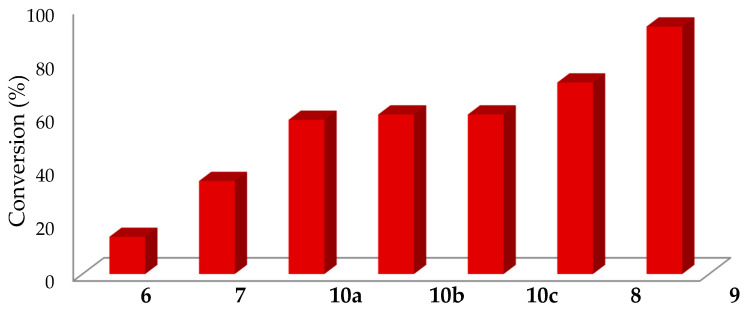
Comparison of the catalytic activity of Pd (II)–sulfosalan complexes **6**–**10** in the Suzuki–Miyaura cross-coupling reaction of iodobenzene and phenylboronic acid: Conversions are calculated for iodobenzene. Catalysts: Na_2_[Pd(HSS)] (**6**), Na_2_[Pd(PrHSS)] (**7**), Na_2_[Pd(BuHSS)] (**8**), Na_2_[Pd(dPhHSS)] (**9**), *rac*-Na_2_[Pd(CyHSS)] (**10a**), Na_2_[Pd(*trans*-CyHSS)] (**10b**) and Na_2_[Pd(*cis*-CyHSS)] (**10c**). Conditions: 2.0 × 10^−8^ mol catalyst, 5.0 × 10^−4^ mol iodobenzene, 7.5 × 10^−4^ mol phenylboronic acid, 5 × 10^−4^ mol Cs_2_CO_3_, solvent: H_2_O (V = 3 mL), T = 80 °C and t = 30 min.

For the comparison of the Pd (II)–sulfosalan catalysts **6**–**10**, the Suzuki–Miyaura cross-coupling of iodobenzene and phenylboronic acid were chosen as a standard reaction (Figure 7). With all catalysts, fast and clean reactions were observed. The reaction mixtures retained their original yellow colour throughout the reaction, and no metal precipitation was detected. Conversions (calculated for iodobenzene) were established by gas chromatography after extraction of the reaction mixtures with CHCl_3_. The results are shown Figure 8.

Figure 8 shows that there are substantial differences in the catalytic activities of the various Pd (II)–sulfosalan complexes, with Na_2_[Pd(HSS)] (**6**) being the least effective (14% conversion) and Na_2_[Pd(dPhHSS)] (**9**) being the most active (93% conversion) catalyst. The exact reaction mechanism of the Suzuki–Miyaura cross-couplings catalysed by Pd (II)–sulfosalan complexes in aqueous media is presently unknown. For the reaction of Na_2_[Pd(HSS)] (**6**) and Na_2_[Pd(BuHSS)] (**8**) with H_2_, we obtained evidence of the need for a vacant coordination site for the oxidative addition of H_2_ [43,44]. In the present case, the catalytic activity increased with increasing length of the linker chain in the order **6** (14%) < **7** (35%) < **8** (72%). This is also the order of increasing flexibility of the coordination sphere around the Pd (II) central ion as can be judged also from the solid state structures of **7** and **8** (Figure 7). The Pd (II) complexes with sulfosalan ligands derived from 1,2-diaminocyclohexanes (**10a**–**10c**) catalysed the Suzuki–Miyaura cross-coupling of iodobenzene and phenylboronic acid with equal activities (58%, 60% and 60%, respectively) which is significantly higher than that of Na_2_[Pd(HSS)] (**6**), having also a two-carbon linker group between the N-atoms of the ligand. The conversion data also show that the catalytic performance is insensitive to the stereochemistry of the ligands in **10b** and **10c**. Finally, the outstandingly high catalytic activity of Na_2_[Pd(dPhHSS)] (**9**) (which also contains a two-carbon linker group in its ligand) may stem from the space requirement of the two phenyl substituents. All these observations are in agreement with the assumption that longer and more substituted linker groups in the sulfosalan ligands may facilitate de-coordination of one of the phenolate oxygens and, in such a way, may lead to creation of a vacant coordination site on Pd (II) which is manifested in higher catalytic activities.

The catalytic properties of the two most active catalysts for the Suzuki–Miyaura cross-coupling reactions, Na_2_[Pd(dPhHSS)] (**9**) and Na_2_[Pd(BuHSS)] (**8**), were studied in some detail, mostly from a synthetic viewpoint.

Table 1 shows conversion of reactions between a variety of aryl halides and arylboronic acids (two heteroarylboronic acids were also included). The data show that **9** is able to catalyse the reaction with very high activity, with turnover frequencies (TOF) up to 40,000 h^−1^ (TOF = (mol reacted substrate) (mol catalyst × time)^−1^). As generally observed, aryl iodides reacted faster than aryl bromides (entries 1/14, 6/11 and 12/13); however, with extended reaction times, medium to high conversions could be achieved with aryl bromides, too (entries 8, 9, 11 and 16). The catalyst tolerates several common functional groups; however, aryl or hetaryl halides containing good donor atoms for Pd (II) reacted slower (entries 6, 11, 17 and 20).

Since aryl halides have limited solubility in water, in fact, these reactions take place in aqueous-organic biphasic systems and the actual concentration of the substrates in the catalyst-containing aqueous phase may be very low—this can also lead to low conversions and TOF-s and may mask the chemical differences in reactivity.

Under otherwise identical conditions, the reaction rate depends on the arylboronic acid to aryl halide molar ratio. This is exemplified in Table 2. In view of the data in the table, in most of our experiments, a 50 mol % excess of a boron derivative over the aromatic halide was used.

The catalytic performance and substrate scope of Na_2_[Pd(dPhHSS)] (**9**) and Na_2_[Pd(BuHSS)] (**8**) are further demonstrated by the data in Table 3 and Table 4, respectively. It seems that the chemical nature of the substituents in the boronic acid derivative or in the aryl halide has only a limited influence on the rate of formation of the appropriate biphenyls.

Na_2_[Pd(dPhHSS)] catalysed also the Suzuki–Miyaura cross-coupling of phenylboronic acid with various aryl dihalides; the results are shown in Table 5. It is interesting to see that, with this catalyst, the major (in most cases exclusive) products were the corresponding terphenyl derivatives (entries 2–4). Only in the case of an aryl dihalide with two different halide substituents was a small conversion to the corresponding halogenated biphenyl detected. Such a high selectivity is not generally observed; see the results with the Na_2_[Pd(BuHSS)] catalyst below.

It is shown by the data in Table 3 (entries 5 and 10) that both NaBPh_4_ and KBF_3_Ph can be used as phenyl group donors in the Suzuki–Miyaura reaction with Na_2_[Pd(dPhHSS)] as the catalyst. Although both salts are water-soluble, their use results in modest or medium high conversions. Na-tetraphenylborate was used in Suzuki–Miyaura cross-coupling with aryl dihalides catalysed by Na_2_[Pd(dPhHSS)]; however, the reactions proceeded with low yields (in 1 h reaction time) and incomplete selectivity (Table 6).

The catalytic features of Na_2_[Pd(dPhHSS)] in the Suzuki–Miyaura cross-coupling of aromatic dihalides were compared to those of Na_2_[Pd(BuHSS)]; the latter showed the second highest activity (Figure 8) in cross-coupling of phenylboronic acid and iodobenzene. According to the data in Table 7, Na_2_[Pd(BuHSS)] is also a very active catalyst for this reaction, since in the cases of phenylboronic and 4-tolylboronic acids, uniformly high (close or above 90%) total conversions of the dihalides were achieved (4-methoxyphenylboronic acid reacted less readily). However, although the yield of biphenyls was generally lower than those of the terphenyls, the reactions were far from selective even with aromatic halides containing two identical halogens. The highest biphenyl–terphenyl selectivity was 17:74, obtained in the reaction of 4-tolylboronic acid and 4-bromo-1-iodobenzene.

## 3. Materials and Methods

With the exception of the salan ligands and their Pd complexes, all chemicals and solvents were high-quality commercial products purchased from Sigma-Aldrich/Merck, St. Louis, Missouri, USA; VVR International, West Chester, Pennsylvania, USA; and Molar Chemicals Kft., Halásztelek, Hungary and were used without further purification. Good quality ion-exchanged water was used throughout (S ≤ 2 μS). Gases (Ar and N_2_) were supplied by Linde Gáz Magyarország Zrt., Répcelak, Hungary.

### 3.1. Synthesis of the Sulfosalan Ligands

HSS [44], PrHSS [45], BuHSS [46] and *rac*-CyHSS [46] were synthesized according to published methods. Synthetic procedures for dPhHSS as well as for *cis*- and *trans*-CyHSS are described below.

#### 3.1.1. 1,2-Diphenyl-*N*,*N*’-*bis*(2-hydroxy-5-sulfonatobenzyl)-1,2-diaminoethane-dPhHSS

This was prepared from the appropriate salen derivative (dPhS) by hydrogenation to afford the benzylamino intermediate (dPhHS) followed by sulfonation to yield dPhHSS.

Synthesis of dPhS:

*meso*-1,2-Diphenyl-ethylenediamine (4.0 g, 18.80 mol) was added into a round-bottom flask containing 50 mL ethanol. To this solution, salicylaldehyde (3.70 mL, 37.60 mmol) was added and the mixture was stirred at 25 °C for 1 h, resulting in formation of a yellow precipitate. The reaction mixture was filtered, and the product was washed with ethanol to obtain dPhS as a yellow crystalline solid. Yield was 7.58 g (17.93 mmol), 95%, yellow crystalline solid.

^1^H-NMR (*d*^6^-DMSO, 360 MHz, *δ*): 5.06 (s, 2H, –CH–CH–), 6.85 (d, *J* = 8.0 Hz, 4H, CH_arom_), 7.20–7.32 (m, 12H, CH_arom_), 8.43 (s, 2H, CH=N–), 13.17 (s, 2H, –OH).

^13^C{^1^H} NMR (*d*^6^-DMSO, 90 MHz, *δ*): 166.17, 160.11, 139.93, 132.53, 131.75, 128.23, 127.86, 127.44, 118.69, 118.50, 116.34, 77.70.

Synthesis of dPhHS

dPhS (4.00 g, 6.87 mmol) was dissolved in methanol (300 mL) followed by the addition of 4 equivalents (1.04 g, 27.48 mmol) of sodium borohydride in 100 mL of methanol under constant stirring at room temperature. The mixture was then stirred at reflux for 30 min. The hot reaction mixture was added dropwise into 600 mL of water with continuous stirring. The white precipitate was filtered, washed with water and dried under vacuum. Yield was 3.90 g (6.67 mmol), 97%, white solid.

^1^H-NMR (*d*^6^-DMSO, 360 MHz, *δ*): 3.00 (d, 14.0 Hz, 2H, CH_2_–NH) and 3.11 (d, 14.0 Hz, CH_2_–NH), 3.49 (s, 2H, –CH–CH–), 6.28–6.32 (m, 4H, CH_arom_), 6.46 (d, *J* = 7.2 Hz, 2H, CH_arom_), 6.68 (t, *J* = 7.3 Hz, 2H, CH_arom_), 6.94–7.04 (m, 10H, CH_arom_).

^13^C{^1^H} NMR (*d*^6^-DMSO, 90 MHz, *δ*: 156.51, 140.81, 128.41, 128.04, 127.98, 127.65, 127.13, 124.60, 118.32, 115.07, 66.82, 47.75.

Synthesis of dPhHSS

In a round-bottom flask, dPhHS (1.00 g, 2.34 mmol) was added in small portions to a mixture of 4 mL of 20% fuming sulfuric acid (oleum) and 1 mL of concentrated sulfuric acid. The flask was cooled in ice water, and the mixture was stirred for 60 min. Then, the content of the flask was carefully added to 25 mL of cold water. The pH of the reaction mixture was set to 4 with a concentrated NaOH solution. Then, the mixture was cooled for 24 h, during which a white precipitate formed. The solid was collected by filtration, washed with cold water and dried under vacuum. The compound thus obtained is the zwitterionic free acid form of the ligand, which is slightly soluble in water.

Yield was 825.05 mg (1.41 mmol), 60%, white solid.

^1^H-NMR (D_2_O, 360 MHz, *δ*): 3.14 (d, *J* = 14.0 Hz, 2H, CH_2_–NH), 3.27 (d, *J* = 14.0 Hz, 2H, CH_2_–NH), 3.89 (s, 2H, –CH–CH–), 6.43 (d, *J* = 8.4 Hz, 2H, CH_arom_), 7.05 (s, 2H, CH_arom_), 7.36–7.54 (m, 12H, CH_arom_).

^13^C{^1^H} NMR (D_2_O, 90 MHz, *δ*): 169.22, 139.50, 129.10, 128.28, 127.26, 126.64, 126.29, 125.56, 118.33, 66.02, 46.27.

IR (ATR), ν/cm^−1^: 594.7, 697.8, 759.0, 1033.6, 1101.7, 1181.0, 1285.9, 1590.8.

ESI-MS for C_28_H_28_N_2_O_8_S_2_ (*m/z*): calcd for [M − H]^−^ 583.121, found 583.121.

#### 3.1.2. *N*,*N*’-*bis*(2-Hydroxy-5-sulfonatobenzyl)-*cis*-1,2-diaminocyclohexane-*cis*-CyHSS

This was prepared from the appropriate salen derivative (*cis*-CyS) by hydrogenation to afford the benzylamino intermediate (*cis*-CyHS) followed by sulfonation to yield *cis*-CyHSS.

Synthesis of *cis*-CyS

According to Section 3.1.1, 1.05 mL (8.76 mmol) of *cis*-1,2-diaminocyclohexane and 1.83 mL (17.51 mmol) of salicylaldehyde yielded 2.50 g (7.71 mmol), 88%, yellow crystalline solid.

^1^H-NMR (*d*^6^-DMSO, 360 MHz, *δ*): 1.54–1.87 (m, 8H, –CH_2_–CH_2_–), 3.67 (s, 2H, CH–CH), 6.82–6.9 (m, 4H, CH_arom_), 7.31 (t, *J* = 7.4 Hz, 2H, CH_arom_), 7.41 (d, *J* = 7.3, 2H, CH_arom_), 8.56 (s, 2H, –CH=N–), 13.66 (s, 2H, –OH).

^13^C{^1^H} NMR (*d*^6^-DMSO, 90 MHz, *δ*): 165.05, 160.76, 132.25, 131.72, 118.66, 118.42, 116.45, 68.12, 30.42, 21.95.

Synthesis of *cis*-CyHS

According to Section 3.1.1, 2.50 g (7.71 mmol) of *cis*-CyS and 1.17 g (30.84 mmol) of sodium borohydride yielded 2.23 g (6.79 mmol), 88%, white crystalline solid.

^1^H-NMR (*d*^6^-DMSO, 360 MHz, *δ*): 1.25–1.36 (m, 4H, –CH_2_–CH_2_–), 1.54–1.65 (m, 4H, –CH_2_–CH_2_–), 2.72 (s, 2H, –CH–CH–), 3.69 (d, *J* = 13.9 Hz, CH_2_–NH), 3.78 (d, *J* = 13.9 Hz, CH_2_–NH), 6.72 (s, 2H, CH_arom_), 7.07 (d, *J* = 7. 6 Hz, 2H, CH_arom_).

^13^C{^1^H} NMR (*d*^6^-DMSO, 90 MHz, *δ*): 157.02, 128.74, 127.79, 125.07, 118.50, 115.30, 55.32, 47.58, 27.11, 21.97.

Synthesis of *cis*-CyH*SS*

According to Section 3.1.1, 1 g (3.06 mmol) CyHS, 4 mL of 20% fuming sulfuric acid (oleum) and 1 mL of concentrated sulfuric acid yielded 821 mg (1.68 mmol), 55%, white crystalline solid.

^1^H-NMR (D_2_O, 360 MHz, *δ*): 1.33–1.69 (m, 8H, –CH_2_–CH_2_–), 2.83 (s, 2H, –CH–CH–), 3.59 (d, *J* = 13 Hz, CH_2_–NH), 3.69 (d, *J* = 13 Hz, CH_2_–NH), 6.60 (d, *J* = 8.7 Hz, 2H, CH_arom_), 7.43–7.47 (m, 4H, CH_arom_).

^13^C{^1^H} NMR (D_2_O, 90 MHz, *δ*): 169.31, 127.79, 127.39, 126.46, 126.18, 118.57, 55.69, 46.69, 26.81, 21.81.

IR (ATR), ν/cm^−1^: 588.8, 6923.0, 846.2, 1039.3, 1101.6, 1138,2, 1435.8, 1604.7.

ESI-MS for C_20_H_26_N_2_O_8_S_2_ (*m/z*): calcd for [M + H]^+^ 487.120, found 487.122 and [M + Na^+^]^+^ 509.102, found 509.104.

#### 3.1.3. *N*,*N*’-*bis*(2-Hydroxy-5-sulfonatobenzyl)-*trans*-1,2-diaminocyclohexane - *trans*-CyHSS

This was prepared from the appropriate salen derivative (*trans*-CyS) by hydrogenation to afford the benzylamino intermediate (*trans*-CyHS) followed by sulfonation to yield *trans*-CyHSS.

Synthesis of *trans*-CyS

According to Section 3.1.1, 3.06 mL (25.50 mmol) of *trans*-1,2-diaminocyclohexane and 5.0 mL (51.00 mmol) of salicylaldehyde yielded 7.89 g (24.32 mmol), 95%, yellow crystalline solid.

^1^H-NMR (*d*^6^-DMSO, 360 MHz, *δ*): 1.39–1.45 (m, 2H, –CH_2_–CH_2_–), 1.59–1.62 (m, 2H, –CH_2_–CH_2_–), 1.76–1.88 (m, 4H, –CH_2_–CH_2_–), 3.36 (s, 2H, CH–CH), 6.81 (d, *J* = 8.0 Hz, 4H, CH_arom_), 7.24–7.29 (t, *J* = 8.1 Hz, 2H, CH_arom_), 7.32–7.35 (d, *J* = 7.3, 2H, CH_arom_), 8.47 (s, 2H, –CH=N–), 13.32 (s, 2H, –OH).

^13^C{^1^H} NMR (*d*^6^-DMSO, 90 MHz, *δ*): 165.00, 160.33, 132.16, 131.54, 118.48, 116.29, 71.29, 32.48, 23.67.

Synthesis of *trans*-CyHS

According to Section 3.1.1, 7.90 g (24.04 mmol) of *trans*-CyS and 3.64 g (61.65 mmol) of sodium borohydride yielded 7.11 g (21.65 mmol), 90%, white crystalline solid.

^1^H-NMR (*d*^6^-DMSO, 360 MHz, *δ*): 1.08–1.23 (m, 4H, –CH_2_–CH_2_–), 1.67 (s, 2H, –CH_2_–), 2.07–2.10 (m, 2H, –CH_2_–), 2.51 (s, 2H, –CH–CH–), 3.79 (d, *J* = 13.7 Hz, 2H, CH_2_–NH), 3.92 (d, *J* = 13.7 Hz, 2H, CH_2_–NH), 6.74–6.78 (t, *J* = 7.2 Hz, 2H, CH_arom_), 6.86 (d, *J* = 7.9 Hz, 2H, CH_arom_), 7.08–7.12 (t, *J* = 7.7 Hz, 2H, CH_arom_), 7.24 (d, *J* = 7.2 Hz, 2H, CH_arom_).

^13^C{^1^H} NMR (*d*^6^-DMSO, 90 MHz, *δ*): 156.04, 129.63, 128.53, 123.28, 118.80, 115.15, 58.91, 44.95, 28.73, 24.01.

Synthesis of *trans*-CyHSS

According to Section 3.1.1, 1 g (3.06 mmol) *trans*-CyHS, 4 mL of 20% fuming sulfuric acid (oleum) and 1 mL of concentrated sulfuric acid yielded 868 mg (1.78 mmol), 58%, white crystalline solid.

^1^H-NMR (D_2_O, 360 MHz, *δ*): 1.05–1.17 (m, 4H, –CH_2_–CH_2_–), 1.57–1.60 (m, 2H, –CH_2_–), 1.89–1.92 (m, 2H, –CH_2_–), 2.34–2.37 (m, 2H, –CH_2_–NH–), 3.59–3.67 (m, 4H, CH_2_–NH), 6.55 (d, *J* = 8.1 Hz, 2H, CH_arom_), 7.38–7.45 (m, 4H, CH_arom_).

^13^C{^1^H} NMR (D_2_O, 90 MHz), *δ*: 169.13, 128.13, 127.18, 126.34, 126.10, 118.51, 59.47, 46.48, 29.68, 24.06.

IR (ATR), ν/cm^−1^: 587.5, 694.4, 840.3, 1033.3, 1165.4, 1207.3, 1281.3, 1598.1

ESI-MS for C_20_H_26_N_2_O_8_S_2_ (*m/z*): calcd for [M + H]^+^ 487.119, found 487.121 and [M + Na^+^]^+^ 509.102, found 509.104.

### 3.2. Synthesis of the Pd–Sulfosalan Complexes

#### 3.2.1. Synthesis of Na_2_[Pd(PrHSS)]

In water (4 mL), 106.75 mg (0.24 mmol) of PrHSS and 73.9 mg (0.26 mmol) of (NH_4_)_2_[PdCl_4_] were dissolved. The pH was set to 7.5 with concentrated NaOH, and the reaction mixture was stirred at 60 °C for 10 h. Then, the solution was cooled to room temperature, and Na_2_[Pd(PrHSS)] was precipitated by addition of 25 mL ice-cold ethanol. The solid was filtered, washed with absolute ethanol and dried under vacuum.

Yield: 129 mg (0.22 mmol), 92%, yellow solid.

^1^H-NMR (D_2_O, 273 K, 360 MHz, *δ*): 1.37–1.49 (m, 1H, −CH_2_CH_2_−), 1.89 (d, *J* = 15.9 Hz, 1H, −CH_2_CH_2_−), 2.48 (d, *J* = 12.7 Hz, 2H, −CH_2_CH_2_−), 2.73 (t, *J* = 12.4 Hz, 2H, CH_2_CH_2_), 3.23 (d, *J* = 12.5 Hz, 2H, CH_2_−NH), 3.29 (d, *J* = 12.5 Hz, 2H, CH_2_−NH), 6.84 (d, *J* = 9.0 Hz, 2H, CH_arom_), 7.36 (s, 2H, CH_arom_), 7.44–7.47 (m, 2H, CH_arom_).

^13^C{^1^H} NMR (D_2_O, 90 MHz, *δ/ppm*): 167.27, 130.40, 128,17, 127.69, 126.47, 118.43, 52.47, 51.98, 26.67.

IR (ATR), ν/cm^−1^: 602.5, 707.8, 823.7, 1038.48, 1103.5, 1198.2, 1290.5, 1477.9

ESI-MS C_17_H_18_N_2_O_8_S_2_PdNa_2_ (*m/z*): calcd for [M − Na^+^]^−^ 570.945, found 570.945.

#### 3.2.2. Synthesis of Na_2_[Pd(dPhHSS)]

According to Section 3.2.1, 140.32 mg (0.24 mmol) of dPhHSS and 73.9 mg (0.26 mmol) of (NH_4_)_2_[PdCl_4_] yielded 149 mg (0.20 mmol), 84%, yellow solid.

^1^H-NMR (D_2_O, 298K, 360 MHz, *δ*): 2.95 (d, *J* = 13.0 Hz, 1H, CH_2_–NH), 3.22 (d, *J* = 13.0 Hz, 1H, CH_2_–NH), 3.80 (d, *J* = 13.0, 1H, CH_2_–NH), 4.25 (d, *J* = 13.0, 1H, CH_2_–NH), 4.29 (d, *J* = 4.0 Hz, 1H, CH–CH), 4.61 (d, *J* = 4.0 Hz 1H, CH–CH), 6.88–7.55 (m, 16H, CH_arom_).

^13^C{^1^H} NMR (D_2_O, 90 MHz, *δ*): 164.47, 163.66, 130.46, 128.86, 128.68, 128.01, 127.58, 126.89, 126.67, 126.55, 123.15, 121.46, 117.98, 117.69, 72.85, 70.36, 51.39, 48.12.

IR (ATR), ν/cm^−1^: 605.8, 708.8, 1028.7, 1106.1, 1175.4, 1300.8, 1473.1, 1592.7

ESI-MS for C_28_H_24_Na_2_N_2_O_8_S_2_Pd (*m/z*): calcd for [M − 2Na]^2−^ 343.001; found 342.992.

#### 3.2.3. Synthesis of Na_2_[Pd(*cis*-CyHSS)]

According to Section 3.2.1, 116.77 mg (0.24 mmol) of *cis*-CyHSS and 73.9 mg (0.26 mmol) of (NH_4_)_2_[PdCl_4_] yielded 121 mg (0.19 mmol), 79%, yellow solid.

^1^H-NMR (D_2_O, 298 K, 360 MHz, *δ*): 1.39–2.21 (m, 8H, CH_2_–CH_2_), 3.59–3.69 (m, 4H, CH_2_–NH), 4.44–4.47 (m, 2H CH–CH), 6.89–6.91 (m, 2H, CH_arom_), 7.53–7.57 (m, 4H, CH_arom_).

^1^H-NMR (D_2_O, 268 K, 360 MHz, *δ*): 1.19–2.01 (m, 8H, CH_2_–CH_2_*),* 3.14 (d, *J* = 13.4, 1H, CH_2_–N), 3.41 (d, *J*_2_ = 12.9 Hz, 2H, CH–CH), 3.47 (d, *J* = 13.4, 1H, CH_2_–N), 3.89 (d, *J* = 13.4, 1H, CH_2_–N), 4.24 (d, *J* = 13.4, 1H, CH_2_–N), 6.70 (d, *J =* 8.4 Hz, 2H, CH_arom_), 7.32–7.38 (m, 4H, CH_arom_).

^13^C{^1^H} NMR (D_2_O, 90 MHz), *δ*: 165.52, 129.41, 127.70, 123.60, 118.70, 65.11, 51.49, 24.26, 20.61.

IR (ATR), ν/cm^−1^: 596.1, 634.3, 707.9, 1028.4, 1107.2, 1170.8, 1301.2, 1475.2, 1592.5

ESI-MS for C_20_H_22_Na_2_N_2_O_8_S_2_Pd (*m/z*): calcd for [M + Na]^+^ 656.954; found 656.956.

#### 3.2.4. Synthesis of [Pd(*trans*-CyHSS)]

According to Section 3.2.1, 116.77 mg (0.24 mmol) of *trans*-CyHSS and 73.9 mg (0.26 mmol) of (NH_4_)_2_[PdCl_4_] yielded 132 mg (0.21 mmol), 88%, yellow solid.

^1^H-NMR (D_2_O, 298 K, 360 MHz, *δ*): 1.25 (s, 4H, CH_2_–CH_2_), 1.80 (s, 2H, CH_2_–CH_2_), 2.51 (s, 2H CH_2_–CH_2_), 2.79 (s, 2H, CH_2_–CH_2_), 3.74 (d, *J =* 13.3 Hz, 2H, CH_2_–NH), 4.17 (d, *J =* 13.3 Hz, 2H, CH–NH), 6.87–6.90 (m, 2H, CH_arom_), 7.52–7.55 (m, 4H, CH_arom_).

^1^H-NMR (D_2_O, 268 K, 360 MHz, *δ*): 0.75 (s, 4H, CH_2_–CH_2_), 1.30 (s, 2H, CH_2_–CH_2_), 2.04 (s, 2H CH_2_–CH_2_), 2.30 (s, 2H, CH–CH), 3.28 (d, *J =* 13.3 Hz, 2H, CH_2_–NH), 3.68 (d, *J =* 13.3 Hz, 2H, CH–NH), 6.38 (d, *J =* 8.3 Hz, 2H, CH_arom_), 7.01–7.04 (m, 4H, CH_arom_).

^13^C{^1^H} NMR (D_2_O, 90 MHz, *δ*): 16.54, 129.40, 128.29, 127.72, 123.53, 118.74, 67.34, 50.29, 29.44, 24.14.

IR (ATR), ν/cm^−1^: 606.7, 708.8, 1033.3, 1105.3, 1165.3, 1298.4, 1473.3, 1590.4

ESI-MS for C_20_H_22_Na_2_N_2_O_8_S_2_Pd (*m/z*): calcd for [M + Na]^+^ 656.954; found 656.956.

#### 3.2.5. Preparation of Pd–Salan Stock Solutions

In water (10 mL), 0.1 mmol of the appropriate salan and 28.4 mg (0.1 mmol) of (NH_4_)_2_[PdCl_4_] were dissolved. The pH was set to 7.5 with 5 M NaOH, and the solution was stirred at 60 °C for 10 h. With time, the light brown solution turned bright yellow. Aliquots of such stock solutions of the catalysts were added to the C–C cross-coupling reaction mixtures. ^1^H-NMR spectra of these stock solutions are identical to those prepared by dissolution of isolated complexes (Figures S110 and S111).

### 3.3. General Procedure

^1^H and ^13^C{^1^H} NMR spectra were recorded on a Bruker Avance 360 MHz spectrometer (Bruker, Billerica, MA, USA) and were referenced to residual solvent peaks. Single crystal X-ray diffraction (SC-XRD) measurements were performed using a Bruker D8 Venture diffractometer, SuperNova X-ray diffractometer system, and the methods and software described in [53,54,55,56,57,58,59,60]. The crystallographic data for all compounds were deposited in the Cambridge Crystallographic Data Centre (CCDC) with the No. CCDC 2020275–2020282 and 2020437. Details of the structure determinations are found in the Supplementary Material.

Infrared spectra were recorded on a Perkin Elmer Spectrum Two FT-IR Spectrometer in attenuated total reflectance (ATR) mode.

Gas chromatographic measurements were done with the use of an Agilent Technologies 7890A instrument (Agilent Technologies, Santa Clara, CA, USA) equipped with a HP-5, 0.25 µm × 30 m × 0.32 mm or an OPTIMA (30 m × 0.32 mm × 1.25 µm) column, and a flame ionization detector 300 °C; the carrier gas was nitrogen 1.9 mL/min.

ESI-TOF-MS measurements were carried out on a BRUKER BioTOF II ESI-TOF spectrometer in positive ion mode or on a Bruker maXis II MicroTOF-Q type Qq-TOF-MS instrument (Bruker Daltonik, Bremen, Germany) both in positive and negative ion modes. The mass spectra were calibrated internally using the exact masses of sodium formate clusters. The spectra were evaluated using Compass Data Analysis 4.4 software from Bruker.

All catalytic Suzuki–Miyaura cross-coupling reactions were carried out under air. The reaction temperatures were kept constant by using a thermostated circulator (set to 80.0 ± 0.1 °C). The products were identified by comparison of their retention time with those of known standard compounds.

## 4. Conclusions

All investigated Pd (II)–sulfosalan complexes 6–10 showed high catalytic activities in the Suzuki–Miyaura reactions of aryl halides and phenylboronic acid derivatives in water and air at 80 °C. The catalytic activity of a particular complex depended on the length of the linker group between the secondary N-atoms of the sulfosalan ligand and/or on the steric congestion around these donor atoms. With the most active catalyst, Na_2_[Pd(dPhHSS)] (9), a TOF = 40,000 h^−1^ was achieved in the reaction of iodobenzene and phenylboronic acid.

## Figures and Tables

**Table 1:**
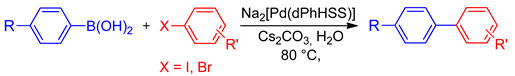
Suzuki–Miyaura cross-coupling reactions of various boronic acids with different aryl halides catalysed by Na_2_[Pd(dPhHSS)].

	Product	(ArX)/(Catalyst) Ratio	Reaction Time (min)	Conversion (%)	TOF (h^−1^)
**1** ^a^	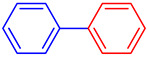	25,000	30	80	40,000
**2** ^a^	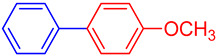	25,000	30	58	29,000
**3** ^a^	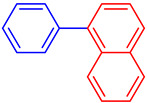	25,000	30	13	6500
**4** ^a^	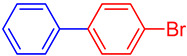	25,000	30	34	17,000
**5** ^a^	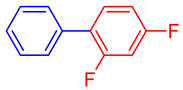	25,000	30	35	17,500
**6** ^a^	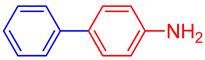	5000	120	86	2150
**7** ^a^	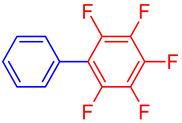	3000	60	38	1140
**8** ^b^	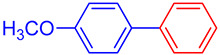	3000	120	70	1050
**9** ^b^	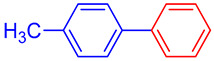	3000	120	62	930
**10** ^a^	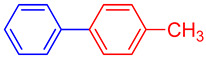	3000	60	27	810
**11** ^b^	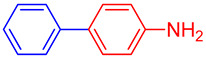	1000	60	77	770
**12** ^a^	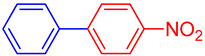	1000	30	100	2000
**13** ^b^	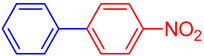	1000	30	82	1640
**14** ^b^	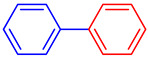	1000	60	71	500
**15** ^b^	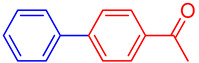	1000	15	100	4000
**16** ^b^	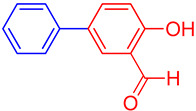	1000	60	95	950
**17** ^b^	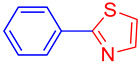	1000	60	50	500
**18** ^b^	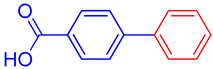	1000	15	100 ^c^	
**19** ^b^	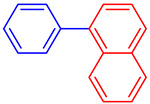	500	60	10	50
**20** ^a^	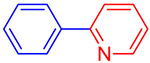	500	60	9	45

Conditions: 1.0 × 10^−6^–2.0 × 10^−8^ mol Na_2_[Pd(dPhHSS)] catalyst, 5.0 × 10^−4^ mol aryl halide, 7.5 × 10^−4^ mol boronic acid derivative, 5.0 × 10^−4^ mol Cs_2_CO_3_, solvent: H_2_O (V = 3 mL) and T = 80 °C. ^a^ Aryl iodide. ^b^ Aryl bromide. ^c^ Conversion determined by ^1^H-NMR.

**Table 2:** Effect of the (phenylboronic acid)/(iodobenzene) ratio on the reaction rate of their Suzuki–Miyaura cross-coupling catalysed by Na_2_[Pd(dPhHSS)].

(Phenylboronic acid)/(Iodobenzene) Ratio	Conversion (%)	TOF (h^−1^)
1.5/1	80	40000
1.25/1	65	32500
1/1	51	25500

Conditions: 2.0 × 10^−8^ mol Na_2_[Pd(dPhHSS)], 5.0 × 10^−4^ mol iodobenzene, 5.0 × 10^−4^ mol Cs_2_CO_3_, solvent: H_2_O (V = 3 mL), T = 80 °C and t = 30 min.

**Table 3:**
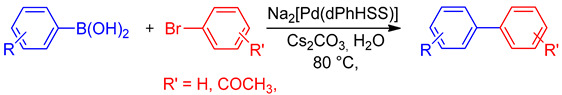
Suzuki–Miyaura cross-coupling reactions of boronic acid derivatives with bromobenzene and 4-bromoacetophenone.

	Boronic Acid	Conversion (%) R’ = H	Conversion (%) R’ = COCH_3_
**1**	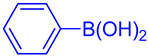	68	66
**2**	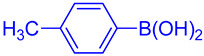	86	73
**3**	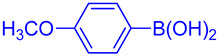	70	71
**4**	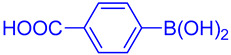	100	100
**5**	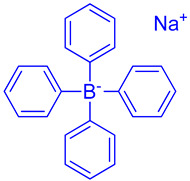	20	27
**6**	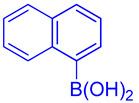	43	63
**7**	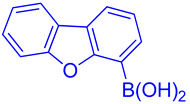	92	81
**8**	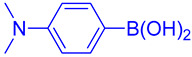	74	96
**9**	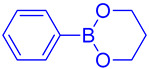	78	42
**10**	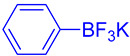	56	62

Conditions: 1.7 × 10^−7^ mol Na_2_[Pd(dPhHSS)], 5.0 × 10^−4^ mol aryl halide, 1.5 × 10^−3^ mol boronic acid, 5.0 × 10^−4^ mol Cs_2_CO_3_, solvent: H_2_O (V = 3 mL), T = 80 °C and t = 1 h.

**Table 4:**
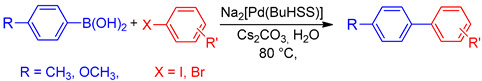
Suzuki–Miyaura cross-coupling reactions of 4-tolylboronic and 4-methoxyphenylboronic acids with various aryl halides.

	Aryl Halide	Conversion (%) R = CH_3_	Conversion (%) R = OCH_3_
**1**	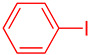	100	100
**2**	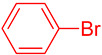	100	65
**3**	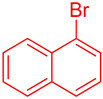	81	41
**4**	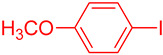	94	89
**5**	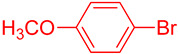	82	78
**6**	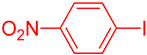	100	72
**7**	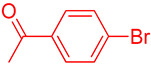	100	100

Conditions: 5.0 × 10^−7^ mol Na_2_[Pd(BuHSS)], 5.0 × 10^−4^ mol aryl halide, 7.5 × 10^−4^ mol 4-tolylboronic acid or 4-methoxyphenylboronic acid, 5.0 × 10^−4^ mol Cs_2_CO_3_, solvent: H_2_O (V = 3 mL), T = 80 °C and t = 1 h.

**Table 5:**
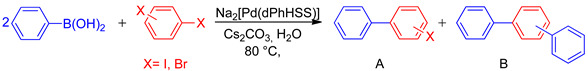
Suzuki–Miyaura cross-coupling of phenylboronic acid and aryl dihalides catalysed by Na_2_[Pd(dPhHSS)].

	Aryl Dihalide	(Substrate)/(Catalyst)	Yield (%)	
			A	B
**1**	4-Bromo-1-iodobenzene	3000/1	5	54
**2**	1,2-Dibromobenzene	3000/1	0	12
**3**	1,3-Dibromobenzene	3000/1	0	53
**4**	1,4-Dibromobenzene	3000/1	0	18

Conditions: 1.7 × 10^−7^ mol [Pd(dPhHSS)], 5.0 × 10^−4^ mol aryl dihalide, 1.5 × 10^−3^ mol phenylboronic acid, 5.0 × 10^−4^ mol Cs_2_CO_3_, solvent: H_2_O (V = 3 mL), T = 80 °C and t = 1 h.

**Table 6:**
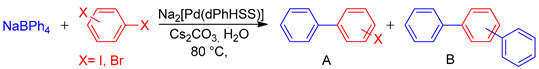
Suzuki–Miyaura cross-coupling reactions of Na-tetraphenylborate with aryl dihalides catalysed by Na_2_[Pd(dPhHSS)].

	Aryl Dihalide	(Substrate)/(Catalyst)	Yield (%)	
			A	B
**1**	4-Bromo-1-iodobenzene	3000/1	3	11
**2**	1,2-Dibromobenzene	3000/1	0	16
**3**	1,3-Dibromobenzene	3000/1	0	4
**4**	1,4-Dibromobenzene	3000/1	4	4

Conditions: 1.7 × 10^−7^ mol Na_2_[Pd(dPhHSS)], 5.0 × 10^−4^ mol aryl dihalide, 1.5 × 10^−3^ mol NaBPh_4_, 5.0 × 10^−4^ mol Cs_2_CO_3_, solvent: H_2_O (V = 3 mL), T = 80 °C and t = 1 h.

**Table 7:**
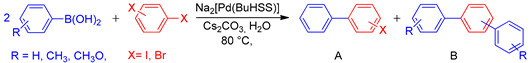
Suzuki–Miyaura cross-coupling reactions of phenylboronic, 4-tolylboronic and 4-methoxyphenylboronic acids with aryl dihalides catalysed by Na_2_[Pd(BuHSS)].

Aryl Halide		Yield (%) R = H		Yield (%) R = CH_3_		Yield (%) R = CH_3_O	
		A	B	A	B	A	B
**1**	4-Bromo-1-iodobenzene	42	53	17	74	14	64
**2**	1,2-Dibromobenzene	17	34	30	64	25	27
**3**	1,3-Dibromobenzene	24	68	27	68	20	37
**4**	1,4-Dibromobenzene	15	50	15	70	30	22

Conditions: 5.0 × 10^−7^ mol Na_2_[Pd(BuHSS)]; 5.0 × 10^−4^ mol aryl dihalide; 1.5 × 10^−3^ mol phenylboronic acid, 4-tolylboronic acid or 4-methoxyphenylboronic acid; 5.0 × 10^−4^ mol Cs_2_CO_3_; solvent: H_2_O (V = 3 mL); T = 80 °C; and t = 1 h.

## Supplementary Materials

The following are available online, ORTEP views of ligands and Pd complexes (9), crystal lattice packing views with indication of π–π interactions and H-bond networks (17), tables of hydrogen bonds in ligands and complexes (7), calculated and experimental powder diffraction patterns for the crystals of **1** × 2 H_2_O and **3**, comparison (superposition) of known and newly determined structures of the ligands (7), table of crystal data and diffraction measurements, ^1^H and ^13^C NMR spectra (Figures S37–S111) of ligands **1**–**5**, Pd(II)-complexes **6**–**10**, starting materials (salens **11**–**15**) and synthetic intermediates (salans **21**–**25**).

## Abbreviations

Na_2_[Pd(HSS)] (6)	Disodium[(*N*,*N*’-*bis*(2-hydroxy-5-sulfonatobenzyl)-1,2-diamino
Na_2_[Pd(PrHSS)] (7)	Disodium[(*N*,*N*’-*bis*(2-hydroxy-5-sulfonatobenzyl)-1,3-diaminopropano)palladate(II)]
Na_2_[Pd(BuHSS)] (8)	Disodium[(*N*,*N*’-*bis*(2-hydroxy-5-sulfonatobenzyl)-1,4-diaminobutano)palladate(II)]
Na_2_[Pd(dPhHSS)] (9)	Disodium[(*N*,*N*’-*bis*(2-hydroxy-5-sulfonatobenzyl)-1,2-diphenyl-1,2-diaminoethano)palladate(II)]
Na_2_[Pd(CyHSS)] (10)	Disodium[*N*,*N*’-*bis*(2-hydroxy-5-sulfonatobenzyl)-1,2-diamino-cyclohexano)palladate(II)]; 10 was synthesized from 5a, 5b and 5c as the ligands, yielding 10a, 10b and 10c, respectively
BuHSS (3)	*N*,*N*’-*bis*(2-hydroxy-5-sulfonatobenzyl)-1,4-diaminobutane
CyHSS (5)	*N*,*N*’-*bis*(2-hydroxy-5-sulfonatobenzyl)-1,2-diaminocyclohexane; 5 was synthesized from racemic 1,2-diaminocyclohexane (5a) and from *trans*- and *cis*-1,2- diaminocyclohexane (5b and 5c, respectively)
dPhHSS (4)	*N*,*N*’-*bis*(2-hydroxy-5-sulfonatobenzyl)-1,2-diphenyl-1,2-diaminoethane
HSS (1)	*N*,*N*’-*bis*(2-hydroxy-5-sulfonatobenzyl)-1,2-diaminoethane
PrHSS (2)	*N*,*N*’-*bis*(2-hydroxy-5-sulfonatobenzyl)-1,3-diaminopropane

## References

[1] Pessoa J.C., Correia I. (2019). Salan vs. salen metal complexes in catalysis and medicinal applications. Coord. Chem. Rev..

[2] Borhade S.R., Waghmode S.B. (2008). Phosphine-free Pd–salen complexes as efficient and inexpensive catalysts for Heck and Suzuki reactions under aerobic conditions. Tetrahedron Lett..

[3] Henrici-Olivé G., Olivé S.A. (1974). Palladium (II) complex as hydrogenase model. Angew. Chem..

[4] Dewan A.A. (2014). Highly efficient and inexpensive Palladium-salen complex for room temperature Suzuki-Miyaura reaction. Bull. Korean Chem. Soc..

[5] Diehl H., Hach C.C. (1950). *Bis* (*N*,*N*′-Disalicylalethylenediamine)-μ–Aquodicobalt(II). Inorg. Synth..

[6] Correia I., Pessoa J.C., Veiros L.F., Jakusch T., Dornyei A., Kiss T., Castro M.M.C.A., Geraldes C.F.G.C., Avecilla K. (2005). Vanadium (IV and V) complexes of Schiff bases and reduced Schiff bases derived from the reaction of aromatic o-hydroxyaldehydes and diamines: Synthesis, characterisation and solution studies. Eur. J. Inorg. Chem..

[7] Pessoa J.C., Marcão S., Correia I., Gonçalves G., Dörnyei Á., Kiss T., Jakusch T., Tomaz I., Castro M.M.C.A., Geraldes C.F.G.C. (2006). Vanadium (IV and V) complexes of reduced Schiff bases derived from the reaction of aromatic o-hydroxyaldehydes and diamines containing carboxyl groups. Eur. J. Inorg. Chem..

[8] Correia I., Dörnyei Á., Jakusch T., Avecilla F., Kiss T., Pessoa J.C. (2006). Water-soluble sal_2_en- and reduced sal_2_en-type ligands: Study of their Cu^II^ and Ni^II^ complexes in the solid state and in solution. Eur. J. Inorg. Chem..

[9] Adão P., Pessoa J.C., Henriques R.T., Kuznetsov M.L., Avecilla F., Maurya M.R., Kumar U., Correia I. (2009). Synthesis, characterization, and application of vanadium–salan complexes in oxygen transfer reactions. Inorg. Chem..

[10] Tshuva E.Y., Gendeziuk N., Kol M. (2001). Single-step synthesis of salans and substituted salans by Mannich condensation. Tetrahedron Lett..

[11] Sukanya D., Evans M.R., Zeller M., Natarajan K. (2007). Hydrolytic cleavage of Schiff bases by [RuCl_2_(DMSO)_4_]. Polyhedron.

[12] Sippola V.O., Krause A.O.I. (2003). Oxidation activity and stability of homogeneous Cobalt-sulphosalen catalyst: Studies with a phenolic and a non-phenolic lignin model compound in aqueous alkaline medium. J. Mol. Catal. A Chem..

[13] Ebrahimi T., Aluthge D.C., Patrick B.O., Hatzikiriakos S.G., Mehrkhodavandi P. (2017). Air- and moisture-stable Indium–salan catalysts for living multiblock PLA formation in air. ACS Catal..

[14] Ding L., Chu Z., Chen L., Lü X., Yan B., Song J., Fan D., Bao F. (2011). Pd–salen and Pd–salan complexes: Characterization and application in styrene polymerization. Inorg. Chem. Commun..

[15] Maru M.S., Barroso S., Adão P., Alves L.G., Martins A.M. (2018). New salan and salen vanadium complexes: Syntheses and application in sulfoxidation catalysis. J. Organomet. Chem..

[16] Gu X., Zhang Y., Xu Z.-J., Che C.-M. (2014). Iron(II)–salan complexes catalysed highly enantioselective fluorination and hydroxylation of *β*-keto esters and *N*-Boc oxindoles. Chem. Commun..

[17] Nishihara Y. (2013). Applied Cross-Coupling Reactions.

[18] Miyaura N., De Meijere A., Diderich F. (2004). Metal-Catalyzed Cross-Coupling Reactions of Organoboron Compounds with Organic Halides. Metal-Catalyzed Cross-Coupling Reactions.

[19] Beletskaya I.P., Alonso F., Tyurin V. (2019). The Suzuki-Miyaura reaction after the Nobel Prize. Coord. Chem. Rev..

[20] Dixneuf P.H., Cadierno V. (2013). Metal—Catalyzed Reactions in Water.

[21] Joó F. (2001). Aqueous Organometallic Catalysis.

[22] Shaughnessy K.H. (2009). Hydrophilic ligands and their application in aqueous-phase metal-catalyzed reactions. Chem. Rev..

[23] Casalnuovo A.L., Calabrese J.C. (1990). Palladium-catalyzed alkylations in aqueous media. J. Am. Chem. Soc..

[24] Li C.-J. (2005). Organic reactions in aqueous media with a focus on carbon−carbon bond formations: A decade update. Chem. Rev..

[25] Alonso D.A., Nájera C., Kobayashi S. (2012). Water in organic synthesis. Science of Synthesis.

[26] Mori Y., Kobayashi S., Kobayashi S. (2012). Water in Organic Synthesis. Science of Synthesis.

[27] Alonso F., Beletskaya I.P., Yus M. (2008). Non-conventional methodologies for transition-metal catalysed carbon–carbon coupling: A critical overview. Part 2: The Suzuki reaction. Tetrahedron.

[28] Chatterjee A., Ward T.R. (2016). Recent advances in the palladium catalyzed Suzuki–Miyaura cross-coupling reaction in water. Catal. Lett..

[29] Shaughnessy K.H., Colacot T. (2015). Greener Approaches to Cross-Coupling. New Trends in Cross-Coupling: Theory and Applications.

[30] Marziale A.N., Jantke D., Faul S.H., Reiner T., Herdtweck E., Eppinger J. (2011). An efficient protocol for the Palladium-catalysed Suzuki–Miyaura cross-coupling. Green Chem..

[31] DeVasher R.B., Moore L.R., Shaughnessy K.H. (2004). Aqueous-Phase, Palladium-Catalyzed Cross-Coupling of Aryl Bromides under Mild Conditions, Using Water-Soluble, Sterically Demanding Alkylphosphines. J. Org. Chem..

[32] Weeden J.A., Huang R., Galloway K.D., Gingrich P.W., Frost B.J. (2011). The Suzuki reaction in aqueous media promoted by P,N Ligands. Molecules.

[33] Godoy F., Segarra C., Poyatos M., Perís E. (2011). Palladium catalysts with Sulfonate-functionalized-NHC ligands for Suzuki−Miyaura cross-coupling reactions in water. Organometallics.

[34] Roy S., Plenio H. (2010). Sulfonated N-Heterocyclic Carbenes for Pd-catalyzed Sonogashira and Suzuki–Miyaura Coupling in aqueous solvents. Adv. Synth. Catal..

[35] Levin E., Ivry E., Diesendruck C.E., Lemcoff N.G. (2015). Water in N-Heterocyclic Carbene-assisted catalysis. Chem. Rev..

[36] Shahnaz N., Puzari A., Paul B., Das P. (2016). Activation of Aryl Chlorides in Water for Suzuki Coupling with a Hydrophilic Salen-Pd(II) Catalyst. Catal. Commun..

[37] Liu Y.-S., Gu N.-N., Liu P., Ma X.-W., Liu Y., Xie J.-W., Dai B. (2015). Water-Soluble Salen–Pd Complex as an Efficient Catalyst for Suzuki–Miyaura Reaction of Sterically Hindered Substrates in Pure Water. Tetrahedron.

[38] Isley N.A., Wang Y., Gallou F., Handa S., Aue D.H., Lipshutz B.H. (2017). A micellar catalysis strategy for Suzuki–Miyaura cross-couplings of 2-Pyridyl MIDA boronates: No copper, in water, very mild conditions. ACS Catal..

[39] Lipshutz B.H., Ghorai S. (2014). Transitioning organic synthesis from organic solvents to water. What’s your E factor?. Green Chem..

[40] Handa S., Smith J.D., Hageman M.S., Gonzalez M., Lipshutz B.H. (2016). Synergistic and selective copper/ppm Pd-catalyzed Suzuki–Miyaura couplings: In water, mild conditions, with recycling. ACS Catal..

[41] Landstrom E.B., Handa S., Aue D.H., Gallou F., Lipshutz B.H. (2018). EvanPhos: A ligand for ppm level Pd-catalyzed Suzuki–Miyaura couplings in either organic solvent or water. Green Chem..

[42] Lipshutz B.H., Gallou F., Handa S. (2016). Evolution of solvents in organic chemistry. ACS Sustain. Chem. Eng..

[43] Gombos R., Nagyházi B., Joó F. (2018). Hydrogenation of α,β-unsaturated aldehydes in aqueous media with a water-soluble Pd(II)-sulfosalan complex catalyst. React. Kinet. Mech. Cat..

[44] Voronova K., Purgel M., Udvardy A., Bényei A.C., Kathó Á., Joó F. (2013). Hydrogenation and redox isomerization of allylic alcohols catalyzed by a new water-soluble Pd–tetrahydrosalen complex. Organometallics.

[45] Lihi N., Bunda S., Udvardy A., Joó F. (2020). Coordination chemistry and catalytic applications of Pd(II)–, and Ni(II)–sulfosalan complexes in aqueous media. J. Inorg. Biochem..

[46] Voronova K., Homolya L., Udvardy A., Bényei A.C., Joó F. (2014). Pd-tetrahydrosalan-type complexes as catalysts for Sonogashira couplings in water: Efficient greening of the procedure. ChemSusChem.

[47] Bunda S., Udvardy A., Voronova K., Joó F. (2018). Organic solvent-free, Pd(II)-salan complex-catalyzed synthesis of biaryls via Suzuki–Miyaura cross-coupling in water and air. J. Org. Chem..

[48] Singh U.P., Tomar K., Kashyap S. (2016). Interconversion of host–guest components in supramolecular assemblies of polycarboxylic acids and reduced Schiff bases. Struct. Chem..

[49] Hoshina G., Ohba S., Tsuchimoto M. (1999). (RS,SR)-N,N′-Bis(2-hydroxybenzyl)-1,2-diphenylethylenediamine. Acta Cryst..

[50] Cannadine J.C., Corden J.P., Errington W., Moore P., Wallbridge M.G.H. (1996). Wallbridge Two Schiff base ligands derived from 1,2-diaminocyclohexane. Acta Cryst..

[51] Muthuraman M., Nicoud J.F., Masse R., Desiraju G.R. (2001). Crystal structure of *N*,*N*’-di-5-nitrosalicylidene-(R,S)-l,2-cyclohexanediamine, C_20_H_20_N_4_O_6_. Z. Kristallogr. NCS.

[52] Bian H.D., Wang J., Wei Y., Tang J., Huang F.P., Yao D., Yu Q., Liang H. (2015). Superoxide dismutase activity studies of Mn(III)/Cu(II)/Ni(II) complexes with Schiff base ligands. Polyhedron.

[53] Bruker (2017). APEX3 and SAINT.

[54] Sheldrick G.M. (2015). SHELXT—Integrated space-group and crystal-structure determination. Acta Crystallogr. Sect. Found. Adv..

[55] Sheldrick G.M. (2008). A short history of SHELX. Acta Crystallogr. A.

[56] Dolomanov O.V., Bourhis L.J., Gildea R.J., Howard J.A.K., Puschmann H. (2009). OLEX^2^: A complete structure solution, refinement and analysis program. J. Appl. Cryst..

[57] Farrugia L.J. (2012). WinGX and ORTEP for Windows: An update. J. Appl. Cryst..

[58] Macrae C.F., Bruno I.J., Chisholm J.A., Edgington P.R., McCabe P., Pidcock E., Rodriguez-Monge L., Taylor R., Streek J.V.D., Wood P.A. (2008). Mercury CSD 2.0—New features for the visualization and investigation of crystal structures. J. Appl. Cryst..

[59] Westrip S.P. (2010). publCIF: Software for editing, validating and formatting crystallographic information files. J. Appl. Crystallogr..

[60] Spek A.L. (2020). checkCIF validation ALERTS: What they mean and how to respond. Acta Cryst. E.

