# Supplementary material for: Palladium (II)–Salan Complexes as Catalysts for Suzuki–Miyaura C–C Cross-Coupling in Water and Air. Effect of the Various Bridging Units within the Diamine Moieties on the Catalytic Performance
Source: Molecules. 2020 Sep 2;25(17):3993. doi: 10.3390/molecules25173993 (PMC7504744; doi:10.3390/molecules25173993)
Supplement: Supplementary file 1 [file molecules-25-03993-s001.pdf]

Supplementary Materials
to

Palladium(II)-Salan complexes as catalysts for Suzuki-Miyaura C-C cross coupling in water and air. Effect of the various bridging units within the diamine moieties on the catalytic performance. ¶

Szilvia Bunda[1,2], Krisztina Voronova[3], Ágnes Kathó[1], Antal Udvardy[1]*, and Ferenc Joó[1,4]*

[1]University of Debrecen, Department of Physical Chemistry, P.O.Box 400, Debrecen, H-4002 Hungary

[2]University of Debrecen, Doctoral School of Chemistry, P.O.Box 400, Debrecen, H-4002 Hungary

[3]Department of Chemistry, University of Nevada, Reno, Reno, NV 89557, USA

[4]MTA-DE Redox and Homogeneous Catalytic Reaction Mechanisms Research Group, P.O.Box 400, Debrecen, H-4002 Hungary

Correspondence: udvardya@unideb.hu; joo.ferenc@science.unideb.hu

¶Dedicated to Prof. P.H. Dixneuf for his outstanding contributions to organometallic chemistry and catalysis, and for his invaluable services to the scientific community.

Table of Contents

Chart 1. Salan ligands **1-5**, and their Pd(II)-complexes (**6-10)**, together with the intermediates of their synthesis (salens **11-15** and hydrogenated salens **21-25**) p. 3

Catalysis experiments and gas chromatographic analysis of the reaction mixtures p. 4

Experimental details for molecular structure determinations of sulfonated salan ligands and Pd(II)-complexes by SC-XRD p. 5

Crystallographic characterization of Sulfonated salan ligands **1-5** p. 6

Figures S1-S4, S6-S15, S17-S36. ORTEP diagrams, crystal packing views with H-bonds and π-π interactions, superpositions of known and newly determined structures: pp. 6-32

Figure S5. Measured and calculated powder diffraction data for **1**×2 H_2O p. 8

Figure S14. Measured and calculated powder diffraction data for **3** p. 16

Table S1. Hydrogen bonds in **1**×2 H_2O p. 7

Table S2. Hydrogen bonds in **2**×5.5 H_2O p. 10

Table S3. Hydrogen bonds in **3** p. 14

Table S4. Hydrogen bonds in **4**×H_2O×dmso p. 18

Table S5. Hydrogen bonds in **5b**×7H_2O p. 26

Table S6. Hydrogen bonds in **5cb**×6 H_2O p. 28

Table S7. Hydrogen bonds in **5ca**×2 H_2O p. 30

Table S8. Crystal data and details of measurements p. 33

References for the crystallographic section p. 35

Figures S37-S45. ^{1}H and ^{13}C{^{1}H} NMR spectra for **11**, **21**, **1**, and **6** pp. 37-42

Figures S46-S56. ^{1}H and ^{13}C{^{1}H} NMR spectra for **12**, **22**, **2**, and **7** pp. 43-50

Figures S57-S65. ^{1}H and ^{13}C{^{1}H} NMR spectra for **13**, **23**, **3**, and **8** pp. 51-56

Figures S66-S76. ^{1}H and ^{13}C{^{1}H} NMR spectra for **14**, **24**, **4**, and **9** pp. 57-63

Figures S77-S87. ^{1}H and ^{13}C{^{1}H} NMR spectra for **15a**, **25a**, **5a**, and **10a** pp. 64-71

Figures S88-S98. ^{1}H and ^{13}C{^{1}H} NMR spectra for **15b**, **25b**, **5b**, and **10b** pp. 72-79

Figures S99-S109. ^{1}H and ^{13}C{^{1}H} NMR spectra for **15c**, **25c**, **5c**, and **10c** pp. 80-87

Figures S110-S111. ^{1}H and ^{13}C{^{1}H} NMR spectra for in situ prepared solutions of Na_2[Pd(dPhHSS] **(9**) p. 88

Chart 1. Salan ligands (hydrogenated sulfonated salens, **1-5)** and their Pd(II)-complexes (**6-10)** used in this study, together with the intermediates of their synthesis (salens 11-15 and hydrogenated salens 21-25). Ligands **1-5** were isolated as zwitterions, complexes **6-10** as Na-salts.

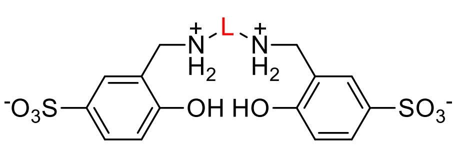

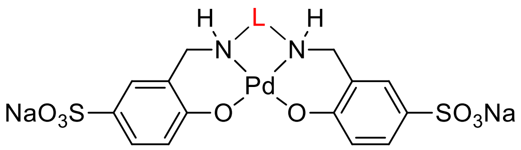

L=				
ethylene	**HSS**	**1**	**Na_2[Pd(HSS)]**	**6**
1,3-propylene	**PrHSS**	**2**	**Na_2[Pd(PrHSS)]**	**7**
1,4-butylene	**BuHSS**	**3**	**Na_2[Pd(BuHSS)]**	**8**
1,2-diphenylethylene	**dPhHSS**	**4**	**Na_2[Pd(dPhHSS)]**	**9**
1,2-cyclohexylene	**CyHSS**	**5**	**Na_2[Pd(CyHSS)]**	**10**

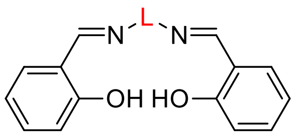

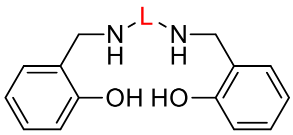

L=				
ethylene	S	**11**	HS	**21**
1,3-propylene	PrS	**12**	PrHS	**22**
1,4-butylene	BuS	**13**	BuHS	**23**
1,2-diphenylethylene	dPhS	**14**	dPhHS	**24**

Abbreviations:

HSS (**1**) – *N,N'*-*bis*(2-hydroxy-5-sulfonatobenzyl)-1,2-diaminoethane;
PrHSS (**2**) - *N,N'*-*bis*(2-hydroxy-5-sulfonatobenzyl)-1,3-diaminopropane;
BuHSS (**3**) - *N,N'*-*bis*(2-hydroxy-5-sulfonatobenzyl)-1,4-diaminobutane;
dPhHSS (**4**) - *N,N'*-*bis*(2-hydroxy-5-sulfonatobenzyl)-1,2-diphenyl-1,2-diaminoethane;
CyHSS (**5**) - *N,N'*-*bis*(2-hydroxy-5-sulfonatobenzyl)-1,2-diaminocyclohexane;
Na_2[Pd(HSS)] (**6**) – disodium[(*N,N'*-*bis*(2-hydroxy-5-sulfonatobenzyl)-1,2-diaminoethano)palladate(II)]
Na_2[Pd(PrHSS)] (**7**) – disodium[(*N,N'*-*bis*(2-hydroxy-5-sulfonatobenzyl)-1,3-diaminopropano)palladate(II)];
Na_2[Pd(BuHSS)] (**8**) – disodium[(*N,N'*-*bis*(2-hydroxy-5-sulfonatobenzyl)-1,4-diaminobutano)palladate(II)];
Na_2[Pd(dPhHSS)] (**9**) – disodium[(*N,N'*-*bis*(2-hydroxy-5-sulfonatobenzyl)-1,2-diphenyl-1,2-diaminoethano)palladate(II)];
Na_2[Pd(CyHSS)] (**10**) – disodium[*N,N'*-*bis*(2-hydroxy-5-sulfonatobenzyl)-1,2-diaminocyclohexano)palladate(II)].

Catalysis experiments and gas chromatographic analysis of the reaction mixtures

Stock solutions of the catalysts (Na_2[Pd(HSS)], Na_2[Pd(PrHSS)], Na_2[Pd(BuHSS)], Na_2[Pd(dPhHSS)], *rac*-Na_2[Pd(CyHSS)], Na_2[Pd(*cis*-CyHSS)]) Na_2[Pd(*trans*-CyHSS)]) were prepared by dissolving 5.0×10^{-7} mol complex in 6 mL water. In general, 0.5 mmol aryl-halide, 0.75 mmol boronic acid derivative (1.5 mmol in the reactions of aryl-dihalides), and 0.5 mmol base (Cs_2CO_3) were used in each reaction. Good quality distilled water was used as solvent, the organic phase was comprised of the substrates. 3 mL of water was used in each reaction. The reactions were carried out at 80 °C in 30-120 min reaction time.

At the end of the reactions, the mixtures were allowed to cool to room temperature and then were extracted by chlorofom (2 mL). After separation of the phases (15-20 min) the organic phase was removed by a Pasteur pipette and filtered through a short $MgSO_4$ plug.

Gas chromatographic determinations were carried out with the use of an Agilent Technologies 7890A type chromatograph, equipped with a flame ionization detector (FID) and an autosampler. HP-5 (30 m × 0.32 mm × 0.25 µm) and OPTIMA (30 m × 0.32 mm × 1.25 µm) capillary columns were used with the following temperature program: 130 °C for 5 min, ramp to 250 °C (60 °C/min), hold at this temperature for 6 min. All components were separated on the baseline. Samples of 1 µl were injected, and the detector was set to 300 °C. Gases, such as N_2 (carrier) and H_2 (for FID) were supplied by gas generators.

Products were identified by their retention times (based upon calibration with the corresponding standards). Product distribution was calculated from the integrated areas of the chromatographic peaks. Calibration of the detector sensitivity for all compounds was carried out in the full concentration range which allowed the measurements to be run without an internal standard. Conversions were calculated for the aryl-halide reactants.

Experimental details for molecular structure determinations of sulfonated salan ligands and Pd(II)-complexes by SC-XRD

Crystal data, and details of data collection and structure refinement are summarized in Table S8. Crystals were mounted on MITEGEN loops, and diffraction intensity data were measured on a Bruker Venture D8 diffractometer (INCOATEC IµS 3.0 dual Cu*K*α and Mo*K*α sealed tube microsources, Photon II Charge-Integrating Pixel Array detector). The data sets were collected and integrated using the *APEX3* software package [S1]. Multi-scan absorption corrections were performed using *SADABS*. The molecular structures were solved with the use of dual methods (*SHELXT*) [S2] and refined on F^2 using the *SHELXL* program [S3] incorporated into the Olex^2 Crystallographic Software & Services [S4] and *WinGX* suite [S5]. All non-hydrogen atoms were refined anisotropically. The crystal **1**×2H_2O was refined as a 2-component twin with a BASF factor of 0.589(5). C8 atom is disordered (59%, 41%) and the value of Z' is 0.5, only half of the formula unit is present in the asymmetric unit, with the other half consisting of symmetry equivalent atoms [Symmetry code: –x,1–y,–z]. In **3** and **4,** Z'=0.5 and symmetry operators are –x,1–y,–z and 1–x,1–y,1–z, to complete the molecule.

The hydrogen atoms of the zwitterionic compounds were placed at idealized positions and refined using a riding model. The positions of hydrogen atoms of H_2O were determined based on the electron density distribution. In compounds **2, 5b, 5cb**, the protons of H_2O molecules could not be located from the electron density map; here we present the best refinement result.

Crystals of **5ca** were very small and low diffracting. **8** was also measured by Rigaku RAXIS-RAPID II diffractometer using a Mo-Kα source for crystals.

In **7** and **8,** RIGU restraints were used, all crystal were twins, of poor quality, and all refinements were uncertain. Despite that, the connectivities in both Pd(II)-sulfosalan complexes were clearly defined; however the inorganic polymers were disordered and did not allow better refinement results.

Data were analyzed by using PLATON [S6] and figures and CIFs for the paper were prepared using the Mercury CSD 4.3.0 software [S7] and pubCIF [S8].

Data sets with structural factors were deposited in the Cambridge Crystallographic Data Centre (CCDC) numbers of 2020275–2020282 and 2020437.

Crystallographic characterization of Sulfonated salan ligands **1-5**

Crystals of **1**×2H_2O were obtained from water and belong to the monoclinic $P2_1/c$ space group. The structure of the molecule is shown on Figure S1, left. The value of *Z*' is 0.5, the asymmetric unit contains half of the zwitterionic ligand (Figure S1, right) and one molecule of water and the other half of the salan was generated by the symmetry operator, −x, 1−y, −z).

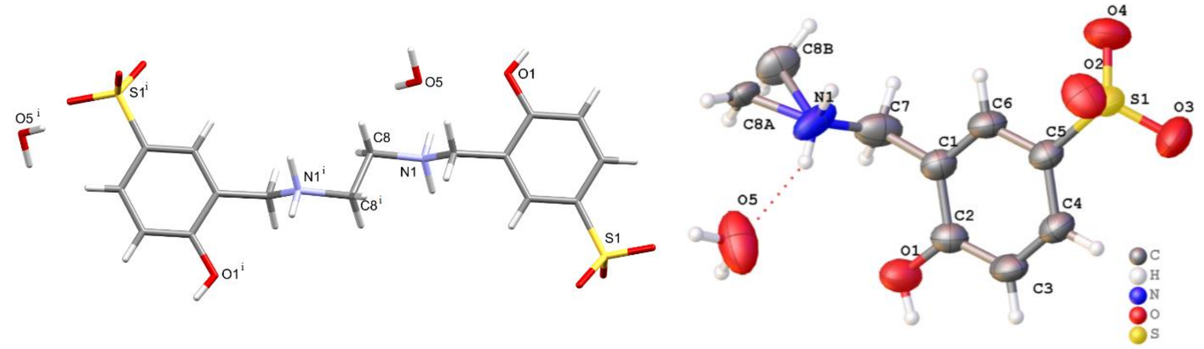

Figure S1. Capped sticks representations of **1**×2H_2O (left) and ORTEP diagram of the asymmetric unit of **1**×2 H_2O showing the atom labelling scheme (right). (Thermal ellipsoids are shown at a 50% probability level. Symmetry code: (i) –x,1–y, –z.)

The crystal lattice is stabilized by the π–π interactions between the aromatic rings (3.861(3) Å, slippages: 1.406 Å and 1.729 Å) and by an extended network of hydrogen bonds (Figures S2–S3, Table S1).

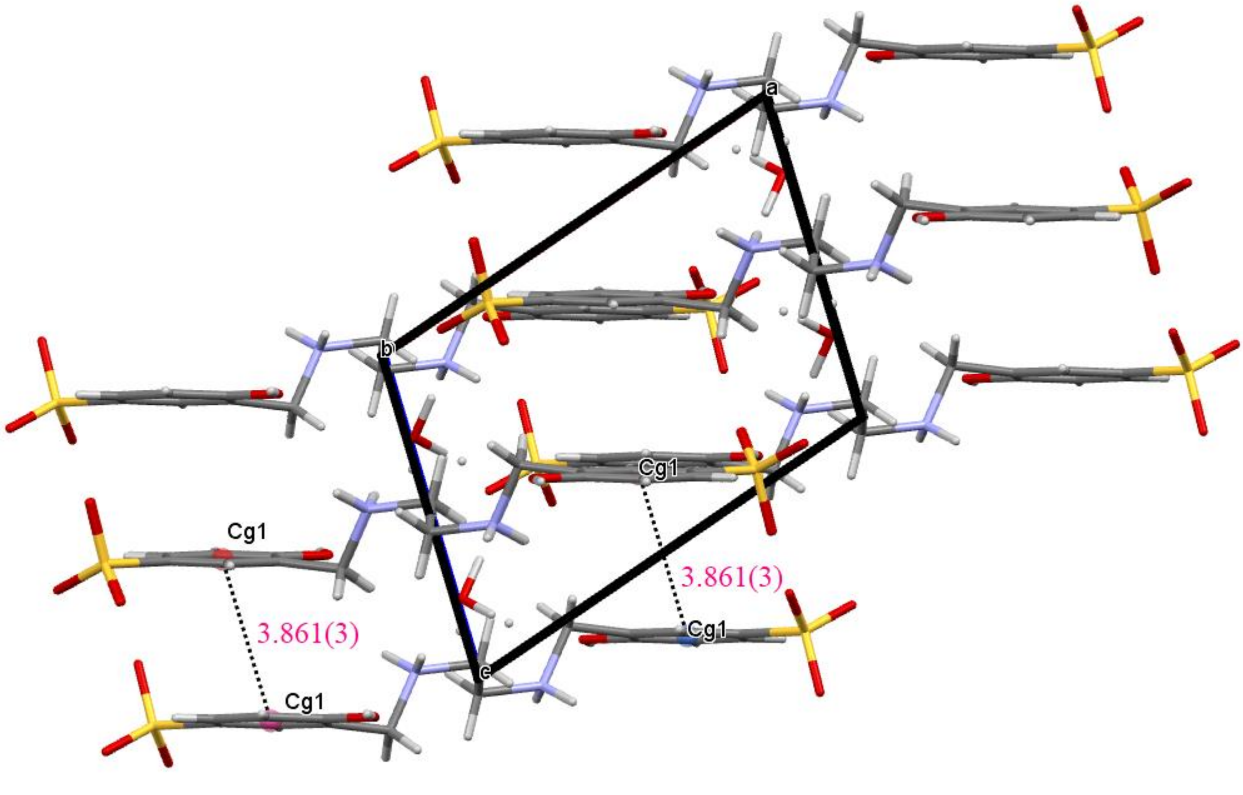

Figure S2. Partial packing view of **1**×2 H_2O on axis "**b**" with π–π interactions. [Cg_1: C1C2C3C4C5C6, symmetry codes: x,1/2–y, –1/2+z and x,1/2–y,1/2+z]

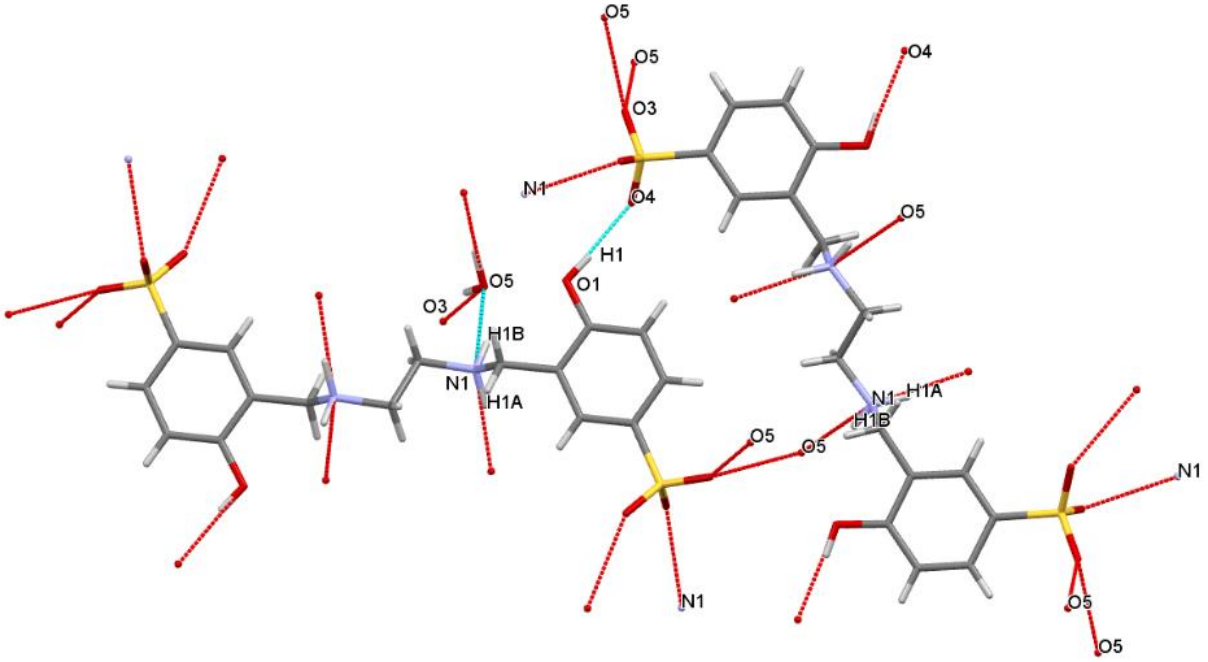

Figure S3. Partial packing view of **1**×2 H_2O with hydrogen bonds.

Table S1. Hydrogen bonds in **1**×2 H_2O

D – H....A	D – H	H...A	D...A	D – H...A
O1 – H1 .. O4$^{(i)}$	0.8200	1.9000	2.705(4)	169.00
N1 – H1A .. O2$^{(ii)}$	1.00(6)	1.79(6)	2.775(6)	171(6)
O5 – H5A .. O3$^{(iii)}$	0.96(6)	1.75(6)	2.705(7)	171(6)
O5 – H5B .. O3$^{(iv)}$	0.99(6)	1.80(6)	2.757(7)	162(5)
C8A – H8AB .. O2$^{(v)}$	0.9700	2.6000	3.328(11)	132.00
C7 – H7A .. O4$^{(vi)}$	0.9700	2.3800	3.239(7)	148.00
C7 – H7B .. O1	0.9700	2.4100	2.776(6)	102.00
C7 – H7B .. O5$^{(i)}$	0.9700	2.5200	3.432(8)	157.00

Symmetry codes (i) 1–x, –1/2+y,1/2–z; (ii) 1–x,1–y,1–z, (iii) x,1/2–y, –1/2+z; (iv) –1+x,y,z; (v) –1+x,y, –1+z; (vi) 1–x,1–y, –z].

The C8 carbon atom is disordered (with 0.41 and 0.59 occupancies). Previously, we obtained crystals of the same compound as a dmso-solvate (**1**×dmso) in which the C8-C8$^{(i)}$ bond length was 1.495 Å [S9]. In **1**×2H_2O the same distance is somewhat larger, 1.529(11) Å. Another difference in the structures of these solvomorphs is in that the aromatic rings are at 34.95° angle to each other (see the superposition of the two molecules in Figure S4).

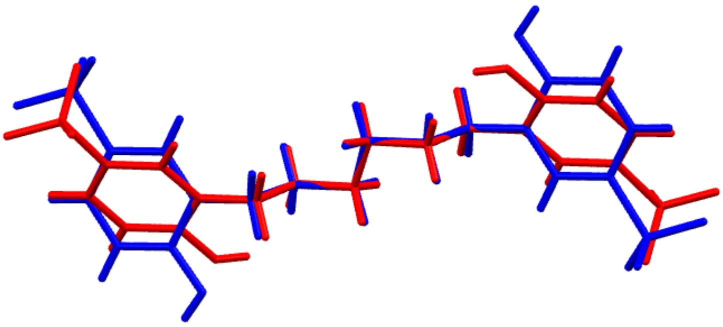

Figure S4. Superposition of **1**×2 H_2O (blue) [this work] and **1**×2dmso (red) [S9]. (Solvent molecules are omitted for the clarity.)

The powder diffraction pattern calculated from the cell parameters of the crystals obtained from water and the one measured experimentally on the powdery product yielded by the synthesis, are identical (Figure S5), meaning that the microcrystalline product also contains two molecules of water for one HSS molecule.

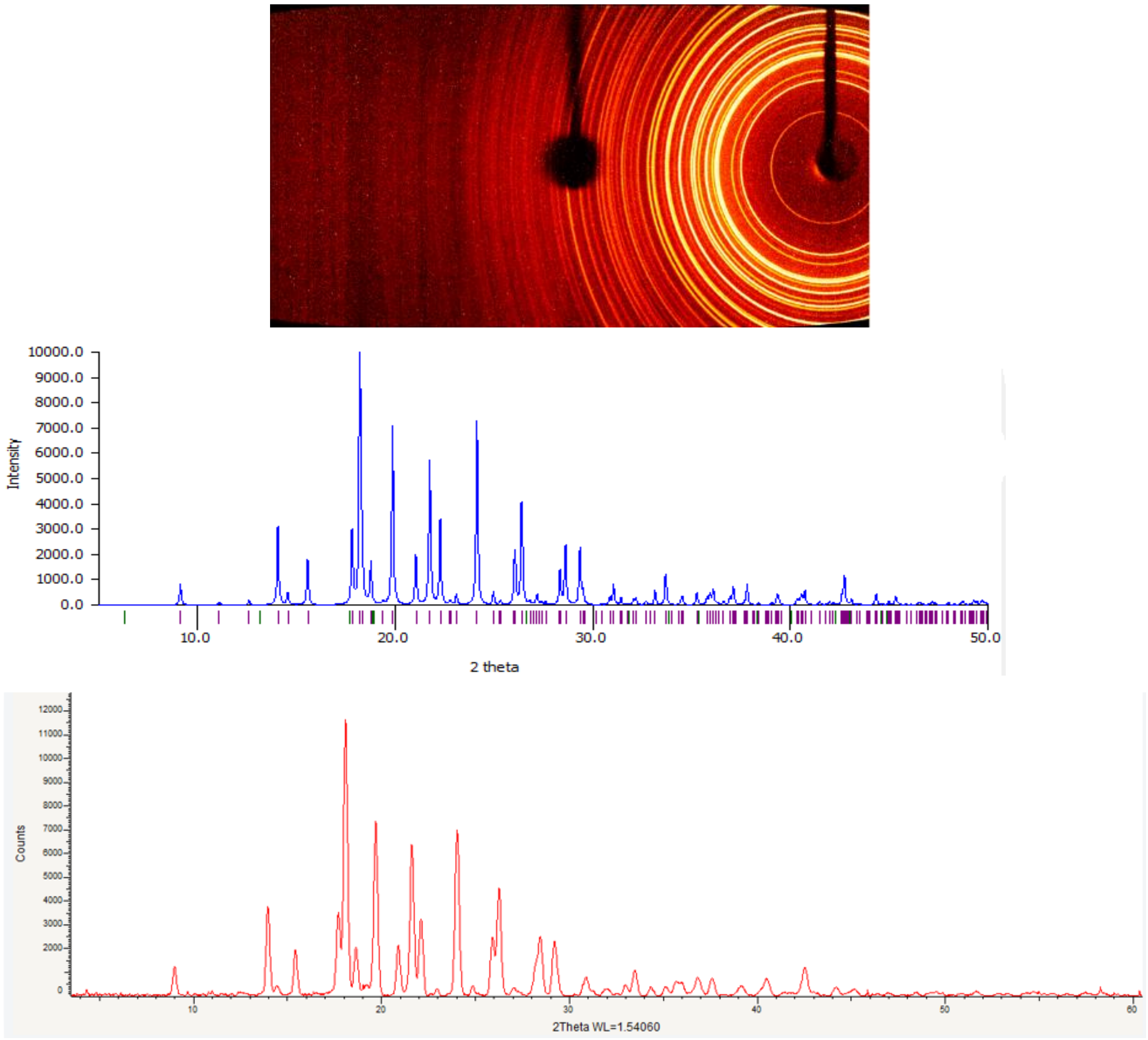

Figure S5. X-ray powder diffraction patterns of **1**×2 H_2O (upper: simulated by Mercury, lower: measured diffraction patterns).

Crystals of PrHSS (**2**) were also obtained from water at room temperature. The compound crystallizes in the orthorhombic *Fdd2* space group, and, in addition to the zwitterionic ligand, the asymmetric unit contains five full water molecules and another one with half crystallographic occupancy (Figure S6).

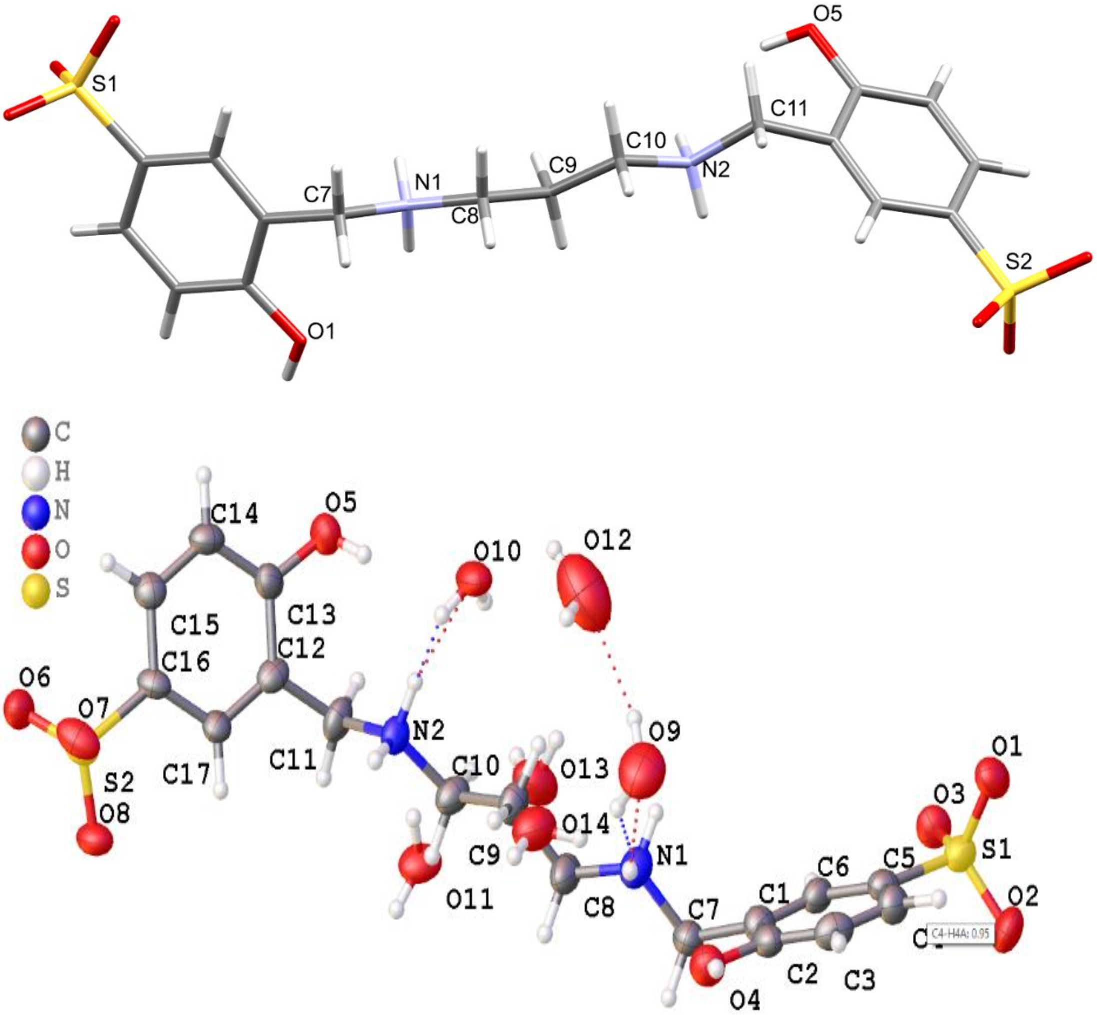

Figure S6. Capped sticks representation of **2**×5.5H_2O, lattice water molecules are omitted for clarity (upper).ORTEP diagram of the asymmetric unit of **2**×5.5 H_2O showing the atom labelling scheme (lower). (Thermal ellipsoids are shown at 50% probability level.)

Due to the large number of water molecules and to the presence of O- and N-atoms in the ligand, strong hydrogen bonds are formed within the lattice (Table S2 and Figures S7, S8).

Table S2. Hydrogen bonds in **2**×5.5 H_2O.

D – H....A	D – H	H...A	D...A	D – H...A
N1 – H1A .. O4	0.9100	2.4700	3.043(13)	121.00
N1 – H1A .. O9	0.9100	2.1000	2.772(13)	130.00
N2 – H2A .. O10	0.9100	1.9000	2.791(9)	165.00
N2 – H2B .. O3$^{(ii)}$	0.9100	1.9300	2.787(10)	157.00
O4 – H4 .. O11$^{(iii)}$	0.8400	1.8700	2.678(10)	162.00
O5 – H5 .. O10	0.8400	2.3000	3.132(11)	168.00
O9 – H9C .. O12	0.8700	2.1200	2.94(2)	156.00
O9 – H9D .. N1	0.8700	2.1100	2.772(13)	132.00
O10 – H10C .. O12$^{(iv)}$	0.8700	2.1400	2.856(18)	140.00
O10 – H10D .. N2	0.8700	2.1200	2.791(9)	133.00
O10 – H10D .. O5$^{(iv)}$	0.8700	2.4700	3.132(11)	133.00
O11 – H11D .. O4$^{(v)}$	0.8700	2.1600	2.678(10)	118.00
O12 – H12A .. O2$^{(vi)}$	0.8700	2.1500	2.780(19)	129.00
O12 – H12B .. O10	0.8700	2.3700	2.856(18)	115.00
O13 – H13B .. O6$^{(i)}$	0.8700	2.0100	2.829(12)	158.00
O14 – H14B .. O9	0.8700	2.4600	2.844(15)	108.00
C4 – H4A .. O8$^{(vii)}$	0.9500	2.5400	3.241(12)	131.00
C6 – H6 .. O3	0.9500	2.5100	2.896(12)	104.00
C11 – H11A .. O5	0.9900	2.4300	2.780(13)	100.00
C15 – H15 .. O7$^{(iv)}$	0.9500	2.5300	3.301(12)	138.00
C17 – H17 .. O8	0.9500	2.5300	2.900(12)	103.00

Symmetry codes: (i) x,y,–1+z (ii) x,y,1+z (iii) –1/4+x,5/4–y,–1/4+z (iv) 1–x,1–y,z , (v) 1/4+x,5/4–y,1/4+z (vi)–1/4+x,5/4–y,3/4+z (vii) –1/4+x,5/4–y,–5/4+z

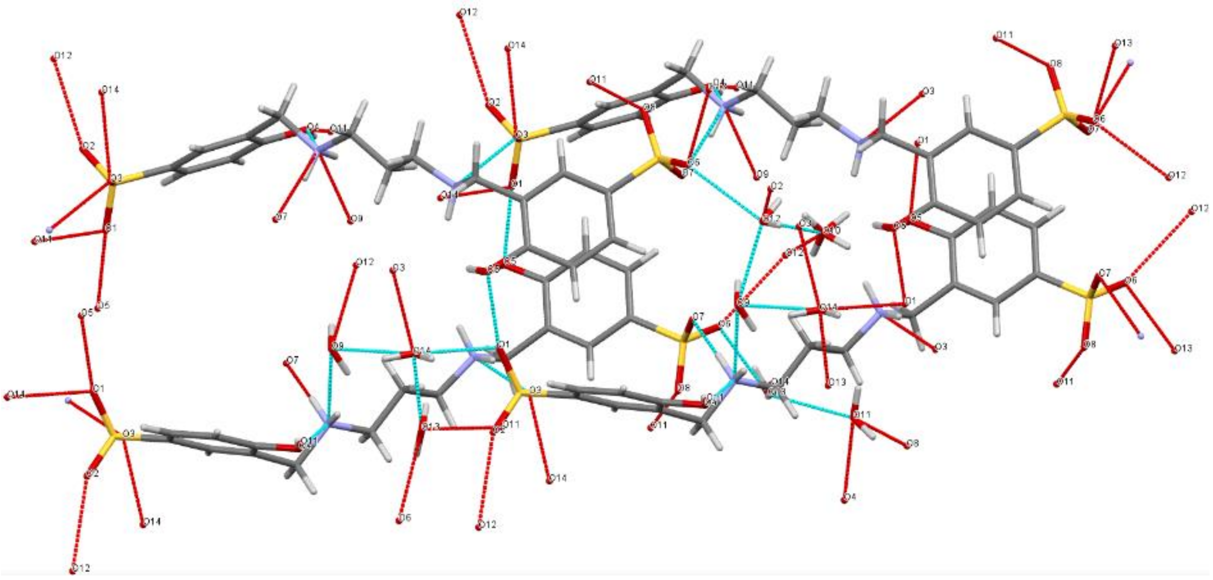

Figure S7. Partial packing view of **2**×5.5 H_2O with hydrogen bonds.

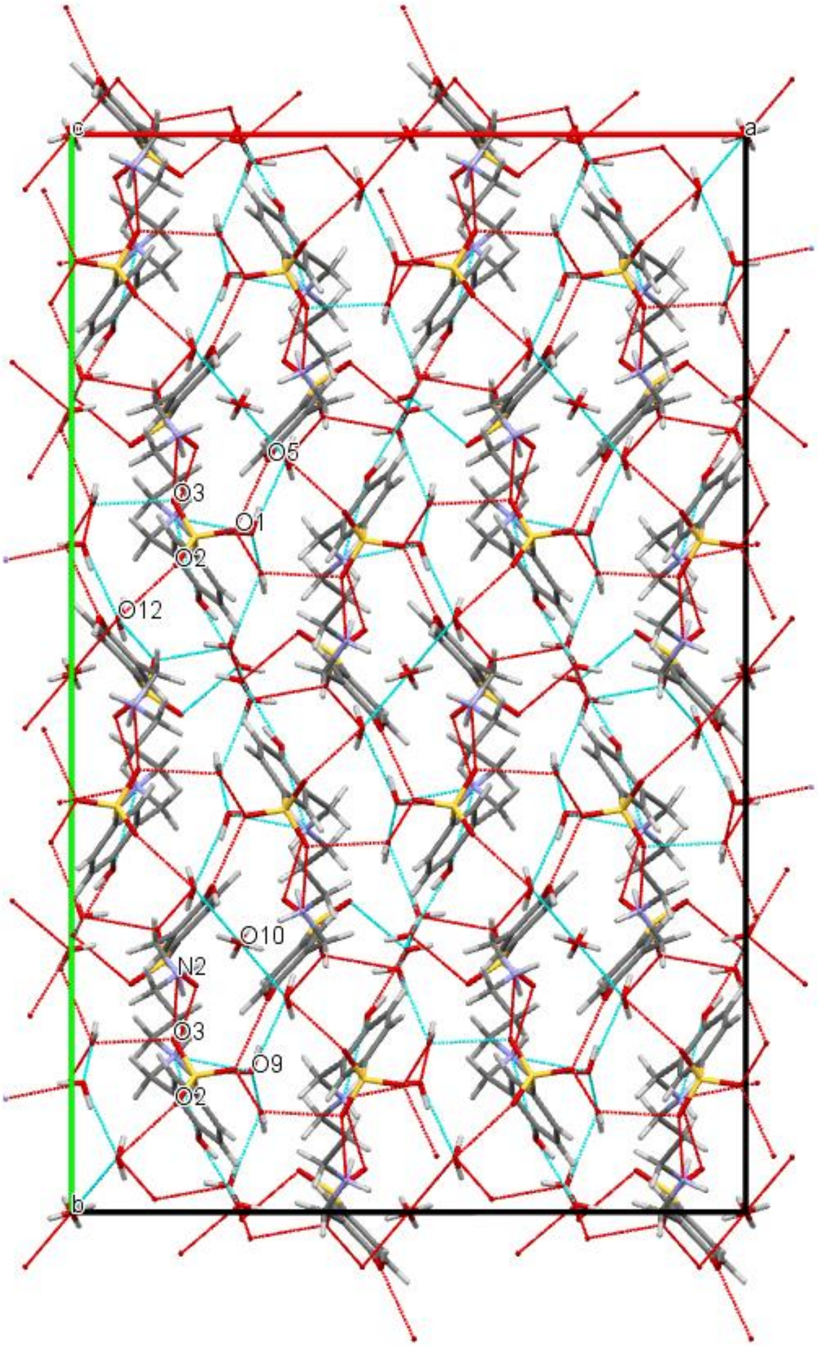

Figure S8. A unit cell of **2**×5.5 H_2O with hydrogen bonds viewed along axis "**c**"

In addition to the hydrogen bonds, the crystal architecture is also stabilized by the π–π interactions between the aromatic rings (3.696(6) Å, slippages:1.315 Å) (Figure S9). The C7–N1 and C11–N2 bond lengths are 1.510(13) Å, and 1.487(14) Å long, which refers to C-N single bonds (as expected in a hydrogenated sulfosalen molecule). The structure also reveals that the sulfonate group occupies the *para*-position relative to the phenolic –OH.

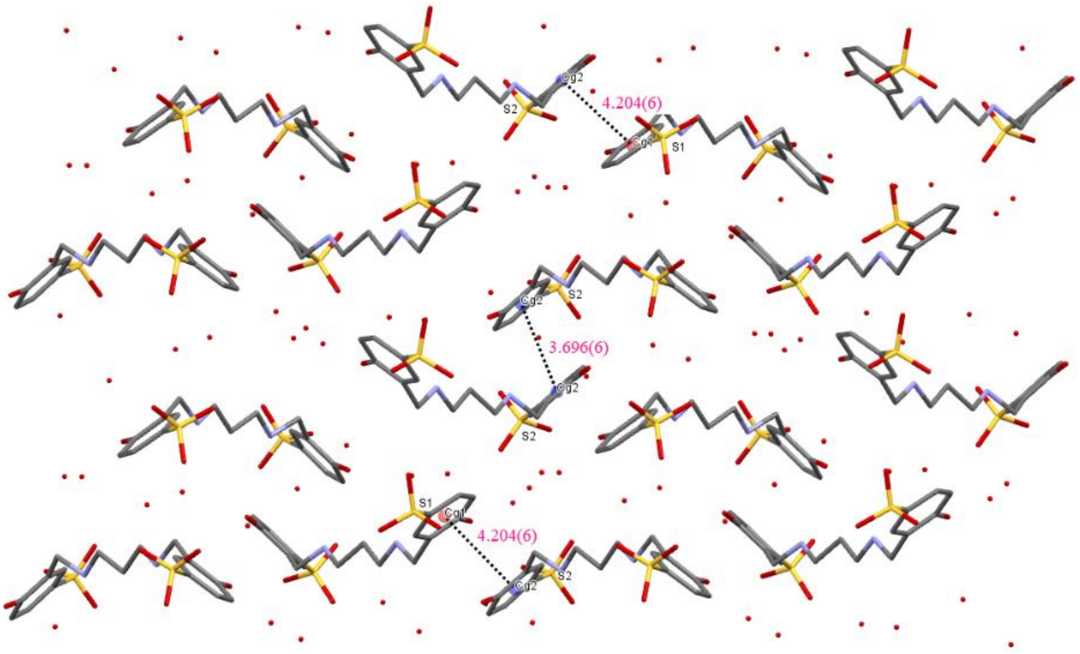

Figure S9. Partial packing view of **2**×5.5 H_2O along axis "**b**" with π–π interactions. All hydrogen atoms are omitted for clarity.

[Cg_1: C1C2C3C4C5C6, Cg_2: C12C13C14C15C16C17, symmetry codes: Cg_1-Cg_2, 5/4–x,1/4+y,–3/4+z; Cg_2-Cg_1, –5/4–x,–1/4+y,3/4+z; Cg_2-Cg_2, 1–x,1–y,z

The starting compound for the synthesis of PrHSS (**2**), i.e *N*,*N*'-bis(2-hydroxybenzyl)-1,3-diaminopropane, PrHS, was previously crystallized with various aromatic polycarboxylates [S10]. SC-XRD studies revealed the protonation of the secondary amine groups of PrHS, similar to the case of PrHSS (**2**), and the structures of the two molecules are very similar in other respects, too (see the superposition of the two molecules in Figures S10, S11).

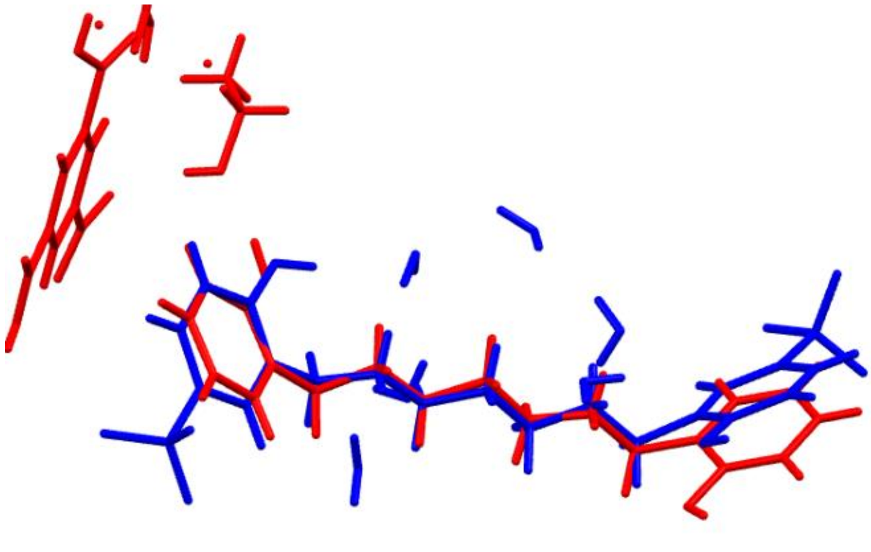

Figure S10. Superposition of *N*,*N'*-bis(2-hydroxybenzyl)propane-1,3-diaminium bis(dihydrogen benzene-1,3,5-tricarboxylate)×ethanol×methanol solvate hemihydrate (red) [S10] and **2**×5.5 H_2O (blue) [this work].

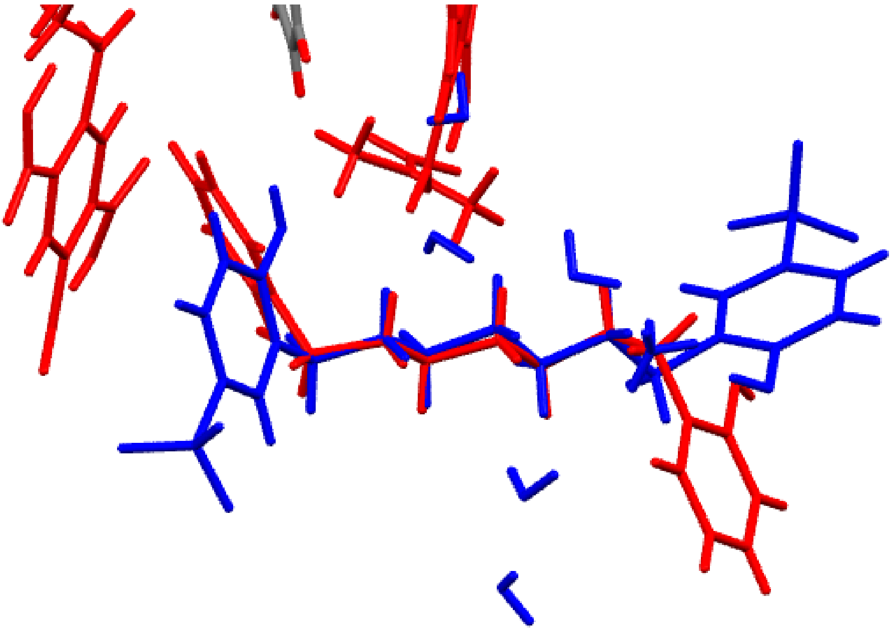

Figure S11. Superposition of bis(*N*,*N'*-bis(2-hydroxybenzyl)propane-1,3-diaminium) dihydrogen benzene-1,2,4,5-tetracarboxylate *N*,*N*-dimethylformamide solvate (red) [S10] and **2**×5.5 H_2O (blue) [this work].

BuHSS (**3**) was also crystallized from water. In contrast to HSS and PrHSS, the asymmetric unit contains no solvent molecules, only the zwitterionic sulfosalan (Figure S12).

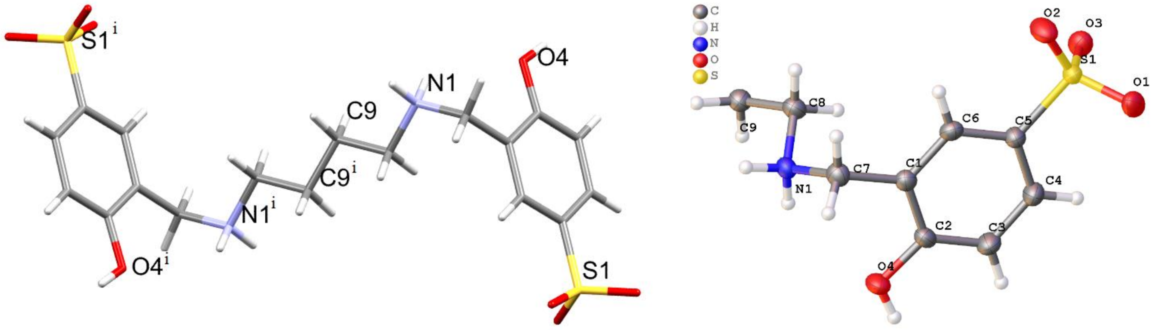

Figure S12. Capped sticks representation of **3** (left). [Symmetry code: (i) –x,1–y,–z] and a ORTEP diagram of the asymmetric unit of **3** showing the atom labelling scheme (right)**.** (Thermal ellipsoids are shown at 50% probability level.)

The compound crystallizes in the monoclinic $P2_1/c$ space group. Oxygen atoms of -SO_3^- group with hydrogen atoms of -N*H* and –O*H* generate a hydrogen-bonding network in the crystal (Figure S13, Table 3) and weak π–π interactions can be detected between the aromatic rings (Figure S14).

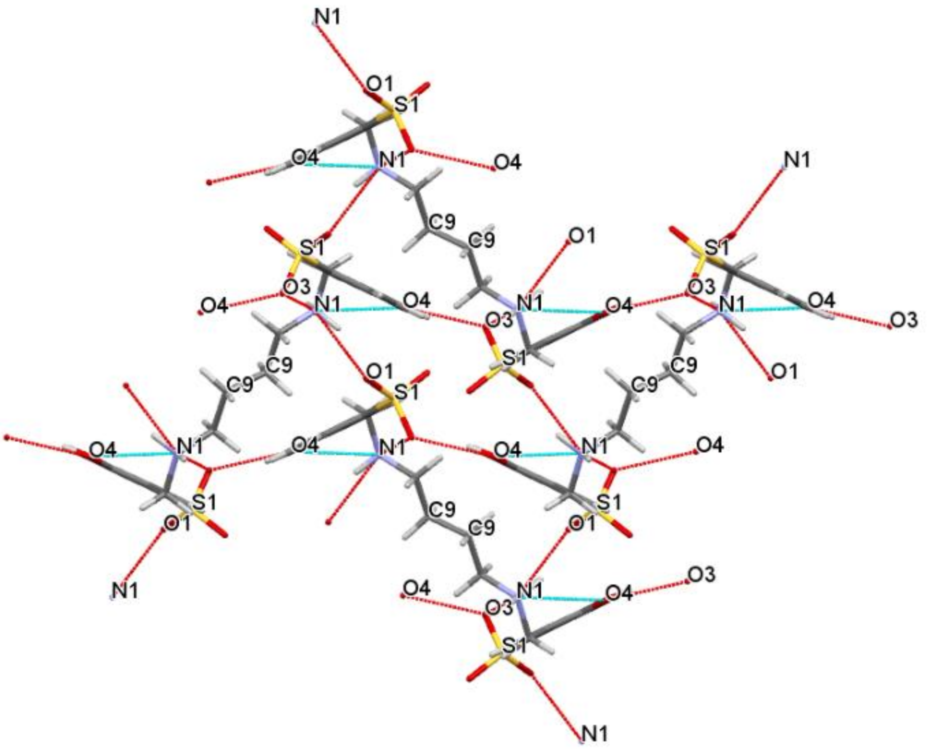

Figure S13. Partial packing view of **3** with hydrogen bonds.

Table S3. Hydrogen bonds in **3**

D – H....A	D - H	H...A	D...A	D - H...A
N1 – H1A .. O4	0.9100	2.4900	3.046(3)	119.00
N1 – H1A .. O1$^{(i)}$	0.9100	2.0200	2.823(4)	146.00
N1 – H1B .. O3$^{(ii)}$	0.9100	1.9200	2.815(4)	169.00
O4 – H4 .. O3$^{(iii)}$	0.8400	1.8600	2.671(3)	163.00
C6 – H6 .. O2	0.9500	2.4900	2.878(4)	104.00
C7 – H7A .. O2$^{(iv)}$	0.9900	2.5200	3.361(3)	143.00
C8 – H8B .. O2$^{(iv)}$	0.9900	2.4000	3.291(4)	150.00
C9 – H9B .. O1$^{(iii)}$	0.9900	2.6000	3.258(3)	124.00

Symmetry codes: (i) 1+x,1/2–y,1/2+z; (ii) 1+x,y,z; (iii) 1–x,–1/2+y,3/2–z; (iv) 1–x,1–y,1–z

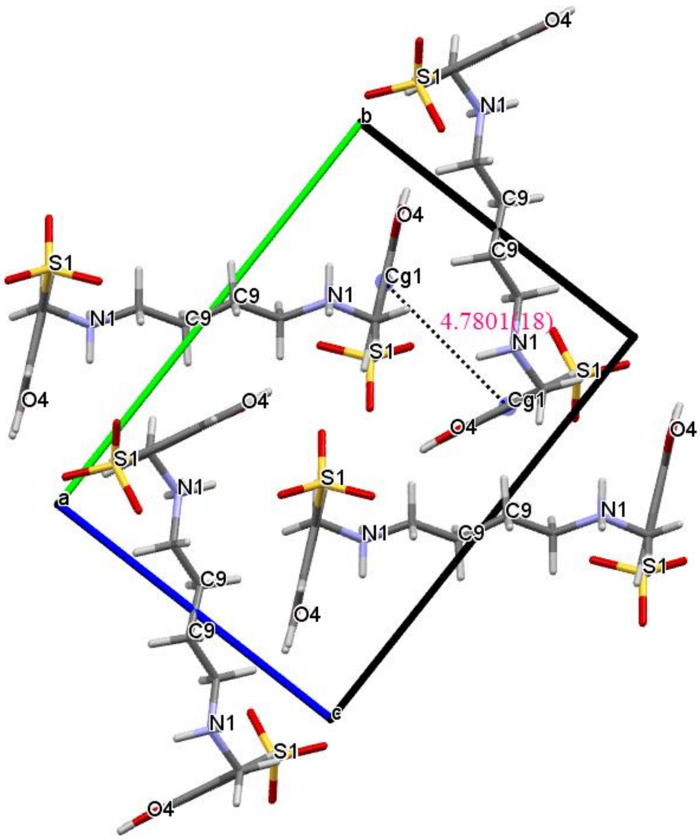

Figure S14. Partial packing view of **3** along axis “**a**” with weak π–π interactions [Cg_1: C1C2C3C4C5C6, symmetry codes: Cg_1-Cg_1 x,1/2–y,–1/2+z and X,1/2–y,1/2+z]

Compared to the structure of BuHSS published by us earlier [S11] it is seen, that although the N1–C7–C1 angles are almost the same (114.28° and 114.4°) in the two molecules, the N1–C7–C1–C8 torsion angles are significantly different (–179.36°, –62.43°), that is the positions of the aromatic groups are different (Figure S15).

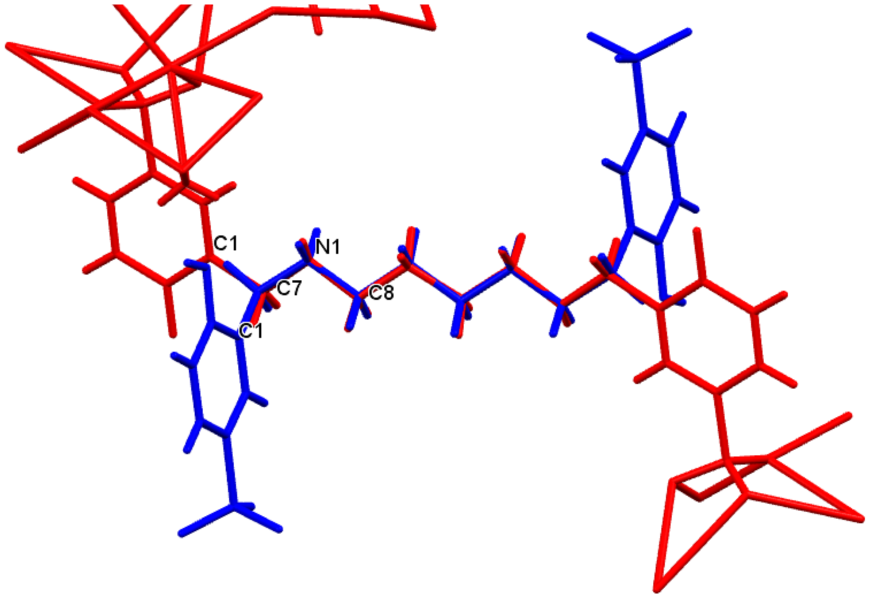

Figure S15. Superposition of **3** (blue) [this work] and n-K_4[μ_8-BuHSS][μ_2-H_2O]$_4$[H_2O]$_6$ (red) [S11].

Powder diffraction pattern of **3** was calculated from the crystal parameters and was found in agreement with the experimentally determined diffractogram (Figure S16).

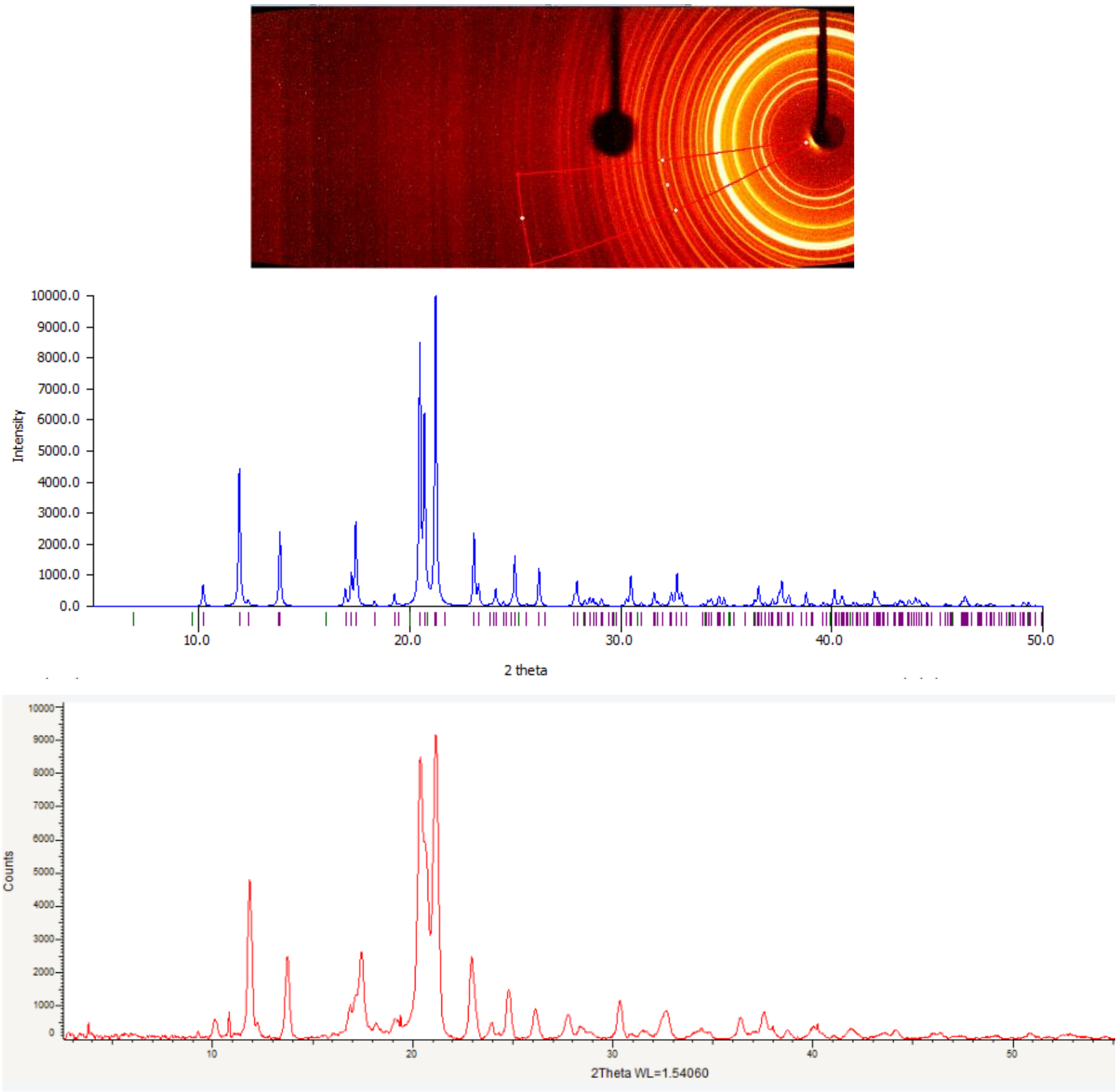

Figure S16. X-ray powder diffraction patterns of **3** (upper: simulated by Mercury, lower: measured diffraction patterns).

Unfortunately, we could not obtain crystals of dPhHSS (**4**) from water. The compound was finely dispersed in wet dmso and left to stand for three weeks at room temperature, after which X-ray quality crystals could be collected. The compound crystallizes in the monoclinic $P2_1/c$ space group; the asymmetric unit contains half of the zwitterionic molecule (Figure S17) (Z'=0.5, Symmetry code: 1–x,1–y,1–z) together with one-one molecule of water and dmso, each.

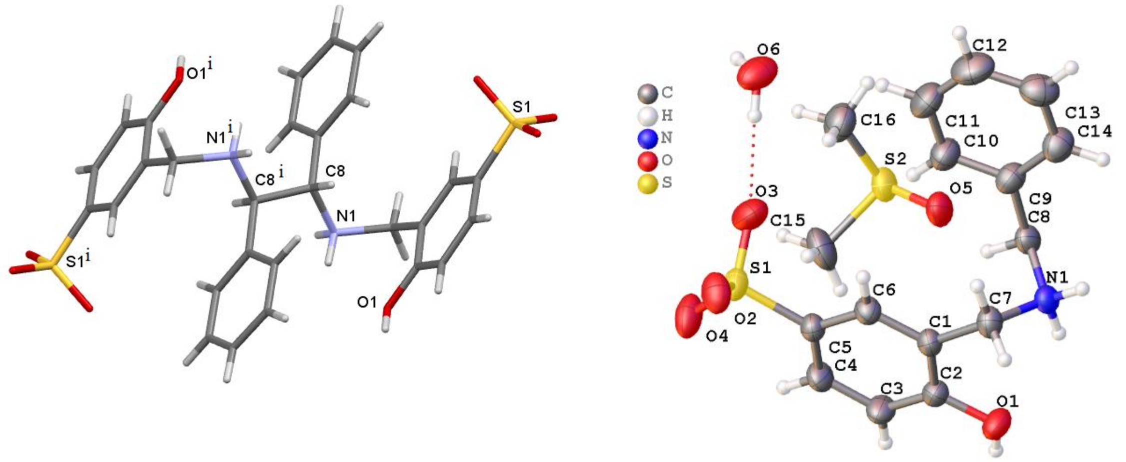

Figure S17. Capped sticks representation of **4**×H_2O×dmso (left). (Solvents molecules are omitted for clarity. Symmetry code: (i) 1–x,1–y,1–z. An ORTEP diagram of the asymmetric unit of **4**×H_2O×dmso showing the atom labelling scheme(right). (Thermal ellipsoids are shown at a 50% probability level.)

Despite the presence of several aromatic rings there are only very weak π–π interactions between them (distances are > 4Å) (Figure S18) but the crystal lattice is stabilized by a strong hydrogen bond network (Figure S19, Table S4).

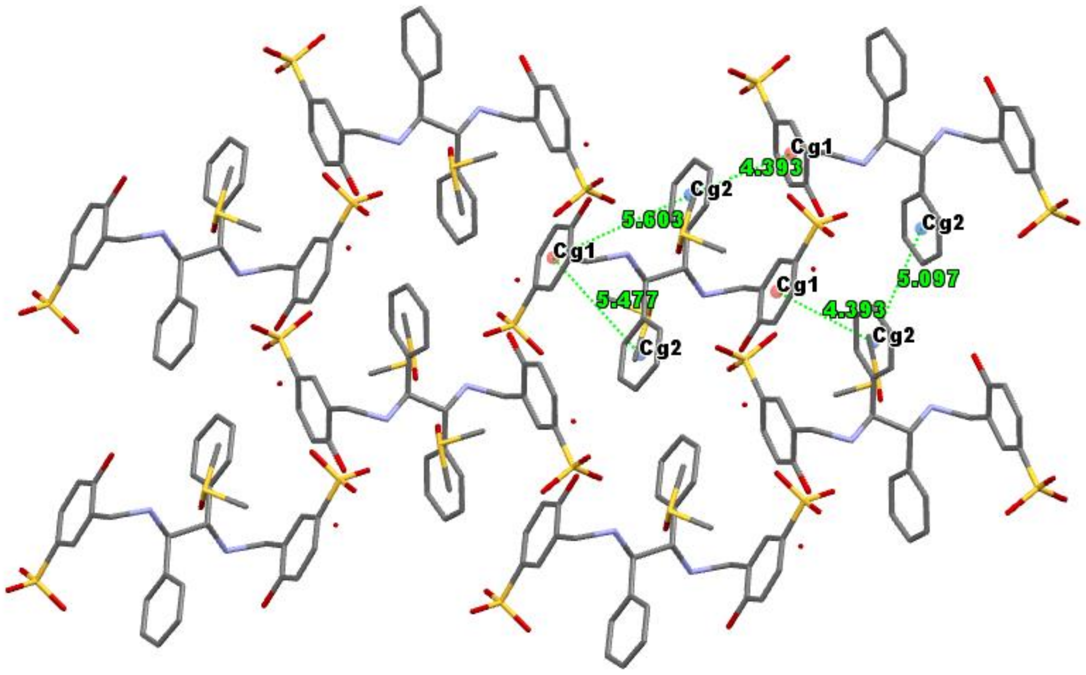

Figure S18. Partial packing view of **4**×H_2O×dmso along axis "c" with very weak π–π interactions.
All hydrogen atoms are omitted for clarity.
[Cg_1: C1C2C3C4C5C6 and Cg_1: C9C10C11C12C13C14 Symmetry codes and distances:
Cg_1-Cg_2, x,y,z, 5.4773(17)Å; Cg_1- Cg_2 1–x,1/2+y,1/2–z, 4.3930(18)Å; Cg_1-Cg_2, 1–x,1–y,1–z 5.6026(17)Å; Cg_2 - Cg_1 1–x,–1/2+y,1/2–z 4.3930(18); Cg_2-Cg_1, 1–x,1–y,1–z, 5.6026(17) Å; Cg_2-Cg_2, 1–x, –y,1–z 5.0970(19) Å.]

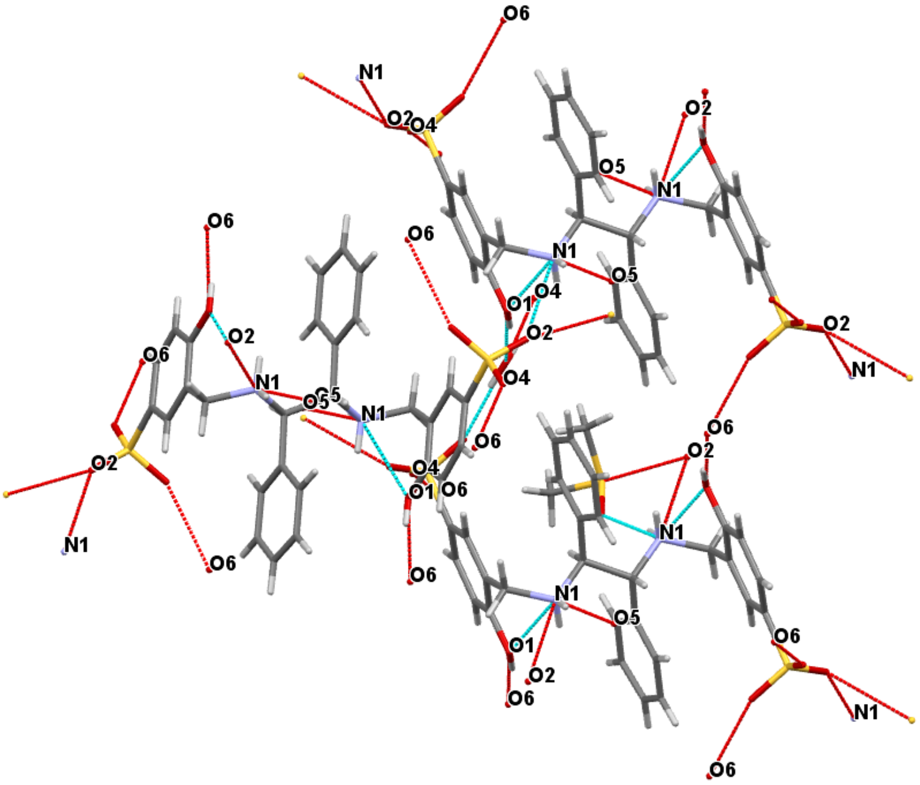

Figure S19. Partial packing view of **4**×H_2O×dmso with hydrogen bonds.

Table S4. Hydrogen bonds in **4**×H_2O×dmso

D – H....A	D - H	H...A	D...A	D - H...A
O1 – H1 .. O6$^{(i)}$	0.8200	1.9300	2.739(3)	172.00
N1 – H1A .. O1	0.8900	2.2300	2.852(3)	126.00
N1 – H1B .. O2$^{(ii)}$	0.8900	2.5300	2.914(3)	107.00
O6 – H6A .. O3	0.8500	2.0200	2.863(3)	172.00
O6 – H6B .. O4$^{(iv)}$	0.8500	2.0200	2.849(3)	165.00
C3 – H3 .. O6$^{(i)}$	0.9300	2.5500	3.246(4)	132.00
C4 – H4 .. O4	0.9300	2.4800	2.875(4)	105.00
C7 – H7A .. O2$^{(v)}$	0.9700	2.4900	3.123(4)	123.00
C8 – H8 .. O5$^{(iii)}$	0.9800	2.3700	3.131(4)	134.00
C14 – H14 .. O5$^{(iii)}$	0.9300	2.3900	3.192(4)	144.00
C16 – H16C .. O2$^{(vi)}$	0.9600	2.6000	3.252(4)	126.00

Symmetry codes: (i) x,1+y,z; (ii) 1-x,1/2+y,1/2-z; (iii) 1-x,1-y,1-z; (iv) -x,-1/2+y,1/2-z; (v) 1-x,1/2+y,1/2-z, (vi) x,1/2-y,1/2+z

The molecular structure of the compound proves that the starting salen (dPhS) was hydrogenated and sulfonated. In the absence of any chiral influence during crystallization, the compound was obtained with *meso* configuration. Superposition of the structures of the salan ligand, *meso* (RS,SR)-*N*,*N*'-bis(2-hydroxybenzyl)-1,2-diphenyl-1,2-diaminoethane [S12] and its sulfonated product, dPhHSS (**4**) can be seen on Figure S20 .

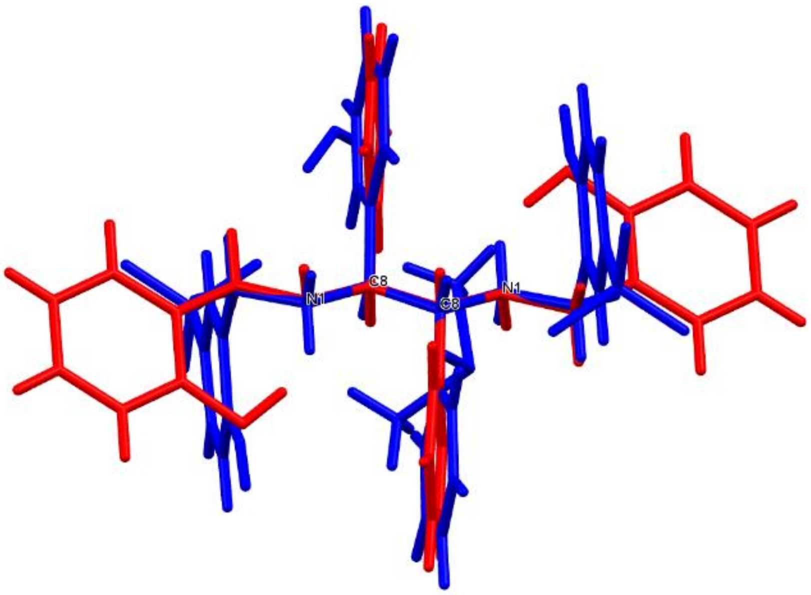

Figure S20. Superposition of (RS,SR)-*N*,*N'*-bis(2-hydroxybenzyl)-1,2-diphenylethylene-diamine (red) [S12] and **4**×dmso×H_2O (blue) [this work].

±-*trans*-CyHSS (**5b**) yielded crystals from water in suitable quality for SC-XRD measurements. The compound crystallized in the triclinic $P\bar{1}$ space group, and –in addition to the zwitterionic molecule– it contained seven water molecules in the asymmetric unit (Figure S21, Figure 22).

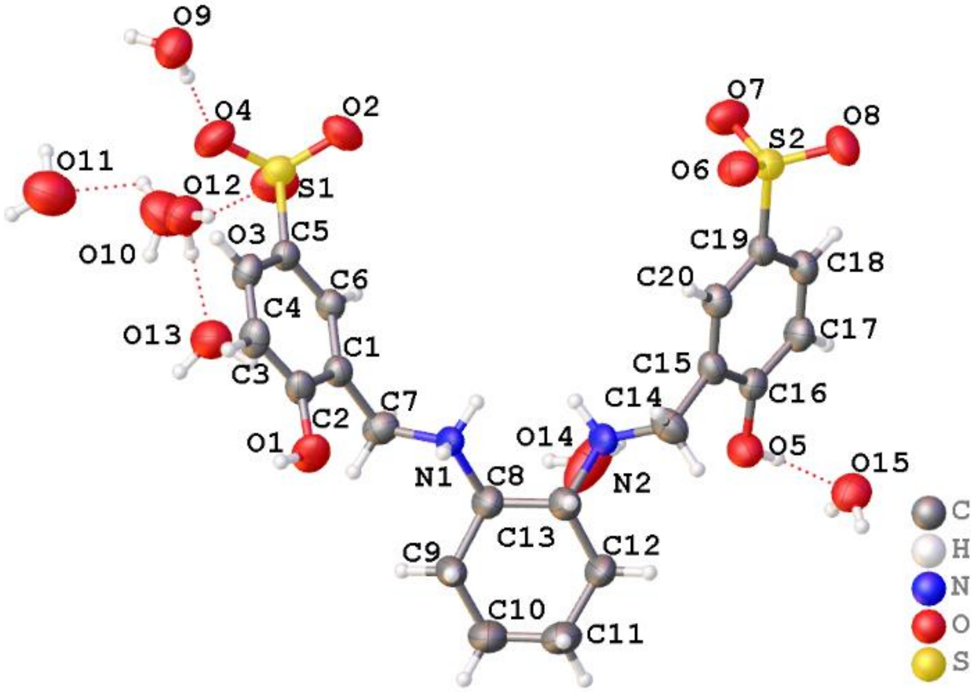

Figure S21. ORTEP diagram of the asymmetric unit of **5b**×7 H_2O showing the atom labelling scheme. (Thermal ellipsoids are shown at a 50% probability level.)

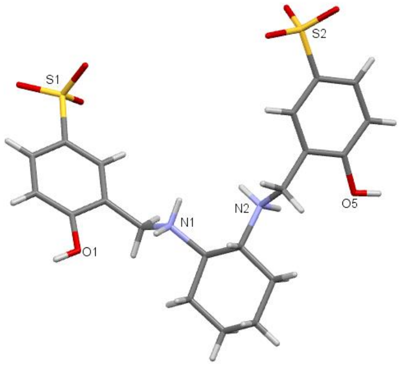

5b

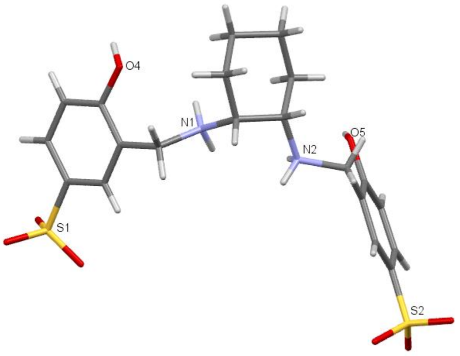

5ca

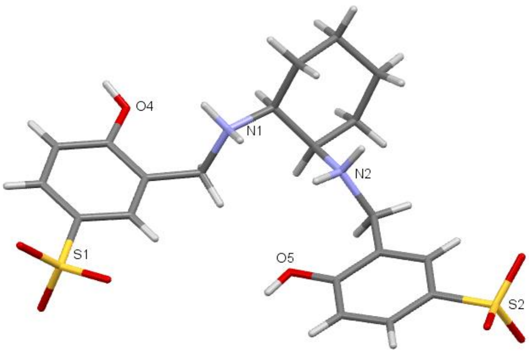

5cb

Figure S22. Structures of ±-*trans*-CyHSS×7H_2O (**5b;** $P\bar{1}$), *cis*-CyHSS×2H_2O (**5ca;** $P2_1/c$), *cis*-CyHSS×6H_2O (**5cb;** $C2/c$). (Water molecules are omitted for clarity.)

The relative configuration of the molecule supports that *trans*-CyHSS was obtained. Since the synthesis and crystallization of the compound was carried out with no chiral influence, the absolute configuration is unknown. The structure of the starting salen for the synthesis of **5b**, i.e. (±)-*trans*-CyS has been determined earlier – the major difference in the two structures is in the position of the aromatic rings [S13] (Figure S23).

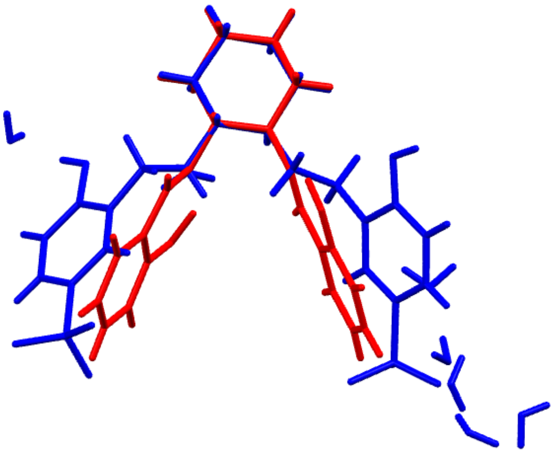

Figure S23. Superposition of **5b** (blue) [this work] and trans-2,2'-((1,2-Cyclohexanediyl)bis(nitrile-methylidyne))bis-phenol (red) [S13].

A racemic mixture of *cis*-CyHSS and *trans*-CyHSS was also subjected to crystallization from water (Figure S22), however, the procedure yielded only crystals of *cis*-CyHSS (**5cb**). This compound crystallized in the monoclinic *C*2/*c* space group, and the asymmetric unit contained the zwitterionic sulfosalan and six water molecules (Figure S24).

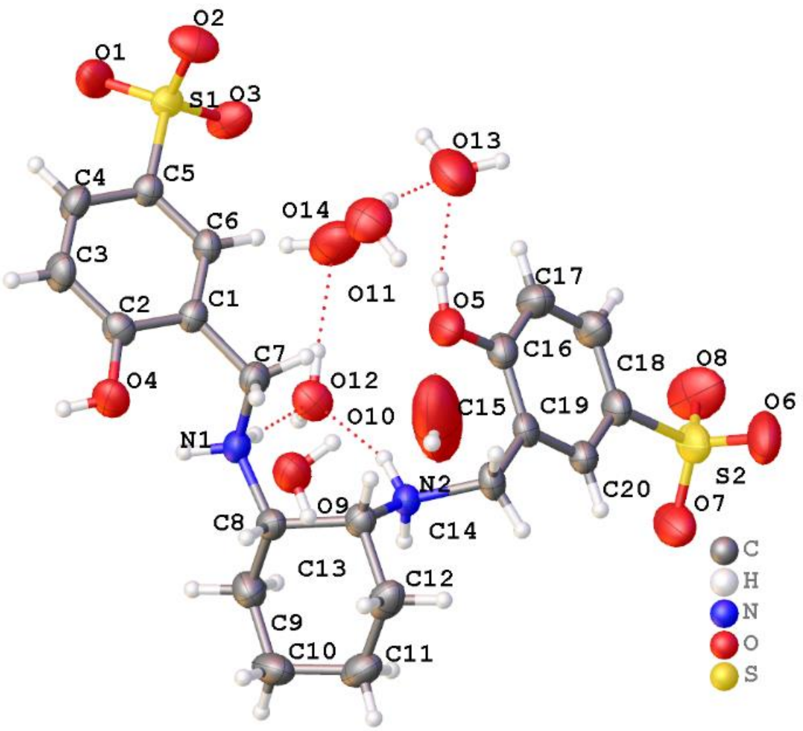

Figure S24. ORTEP diagram of the asymmetric unit of **5cb**×6 H_2O showing the atom labelling scheme. (Thermal ellipsoids are shown at a 50% probability level.)

Crystals of **5ca** synthesized with enantiomerically pure *cis*-1,2-diaminocyclohexane could also be obtained from water (Figure S22). In this case, the crystals belong to the monoclinic $P2_1/c$ space group and the asymmetric unit contained only two water molecules in addition to the zwitterionic sulfosalan (Figure S25). Overlay of the structures of **5ca** and **5cb** (Figure S26) shows, that the cyclohexyl rings precisely overlap and only the position of the aromatic rings are different. This may be due to the different number of water molecules in the asymmetric unit which allows different degree of rotation around the flexible bonds.

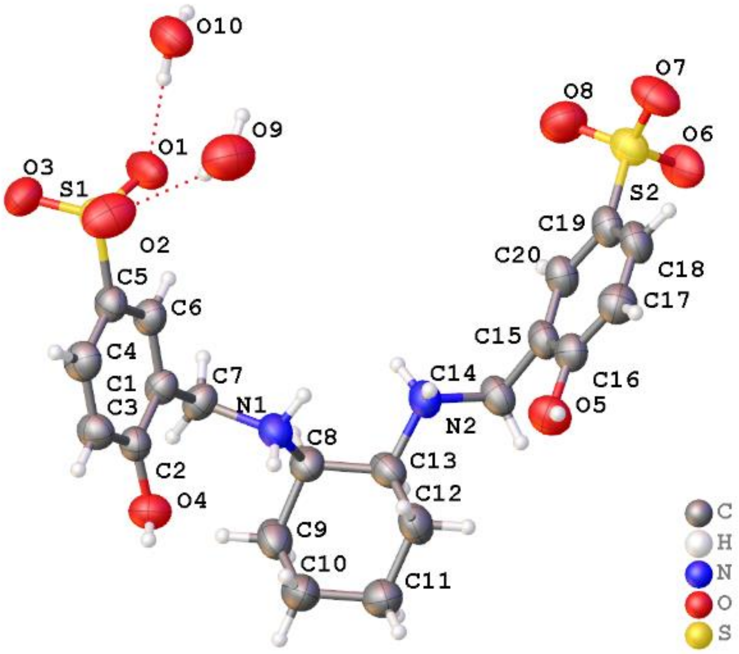

Figure S25. ORTEP diagram of the asymmetric unit of **5ca**×2 H_2O showing the atom labelling scheme. (Thermal ellipsoids are shown at a 50% probability level.)

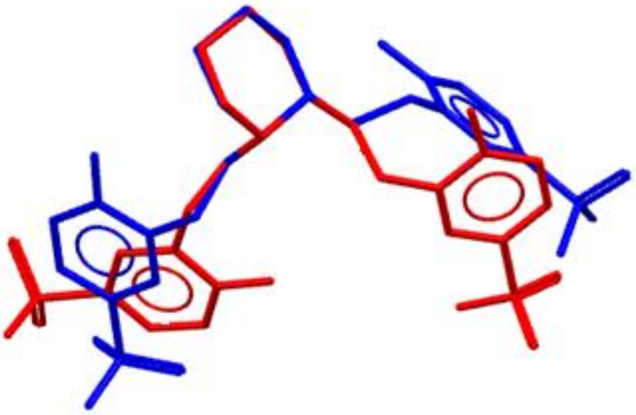

Figure S26. Superposition of **5ca** (blue) and **5cb** (red). (Water molecules and all hydrogen atoms are omitted for the clarity.)

The structure of **5ca** also shows, that the *cis*-conformation in the Schiff-base formed in the reaction of salicylaldehyde and *cis*-1,2-diaminocyclohexane is retained throughout hydrogenation and sulfonation. The cyclohexyl ring of the sulfonated product, *cis*-CyHSS overlaps precisely with the cyclohexyl ring in *N,N'*-di-5-nitrosalicylidene-(*R,S*)-l,2-cyclohexanediamine, published by Desiraju et al. [S14] (see superposition of the molecules, Figure S27)

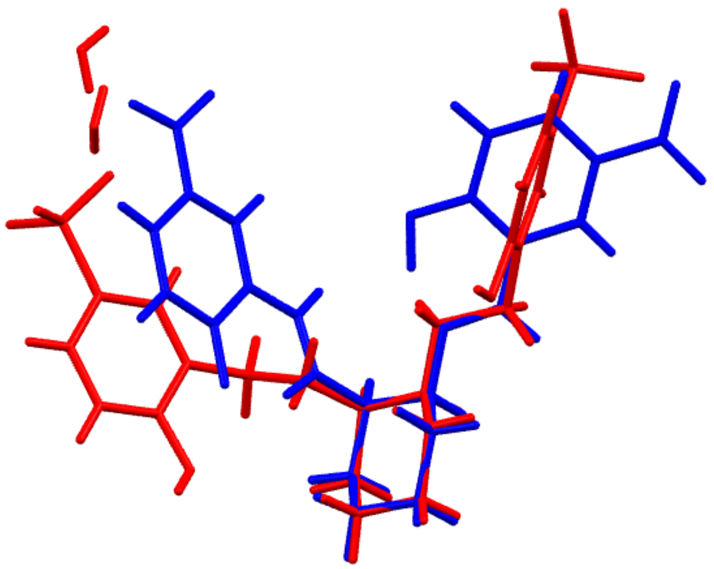

Figure S27. Superposition of **5ca** (red) [this work] and *N,N'*-di-5-nitrosalicylidene-(*R,S*)-1,2-cyclohexanediamine (blue) [S14].

The extensive H-bond networks and π–π interactions in the various CyHSS molecules are shown in Figures S28a,b, Table 5; Figures S29a,b Table 6; Figures S30a,b Table 7.

*Palladium(II) complexes of PrHSS (7) and BuHSS (**8**)*

Na_2[Pd(PrHSS)] (**7**) and Na_2[Pd(BuHSS)] (**8**) were dissolved in 1M KOH solution which were layered by 2-propanol. After a month, yellow crystals of the potassium salts could be collected which were subjected to SC-XRD measurements at 5 °C. K_2[Pd(PrHSS)] (**7'**) crystallizes in the frequently found orthorhombic space group, the centrosymmetric *Pnma* (Z'=0.5, symmetry code: +x, 1/2–y, +z), while K_2[Pd(BuHSS)] (**8'**) belongs to the orthorhombic $P2_12_12_1$ space group. Pillai and co-workers crystallized [Pd(BuHS)] complex from ethanol-water mixture (Figure S36), [S15].

The packing diagrams of the two complexes reveal, that the complexes are placed within the lattice in layers, and the sulfosalan complexes are held together by inorganic polymer chains (Figures S31-S35). In the case of both complexes, the 2D structures are shaped by the electrostatic and van der Waals interactions between the K^+ ions and the O-atoms of the sulfonate groups of the ligand and water molecules, together with the hydrogen bonds within the lattice. Similar polymeric chains were detected by us in crystals of the n-K_4[μ_8-BuHSS][μ_2-

$H_2O]_4[H_2O]_6$ sulfosalan [S11] and in the cases of Ni(II)- and Cu(II)-complexes of *bis*(salicylidene)-1,2-diaminocyclohexane, CyS [S16].

Diffraction measurements were made on several crystals of both complexes at 150 K and at room temperature. Since the crystals were twinned and the polymer chains flexible, despite all our efforts, all *R* values were higher than 10 % together with *wR2*-s >25 %. Due to these errors, the bond lengths and angles determined for the complexes are not suitable for discussion. Nevertheless, the SC-XRD measurements yielded clear atomic connectivities in both cases (Figure S36) and –together with the spectroscopic data– prove the structures of the complexes.

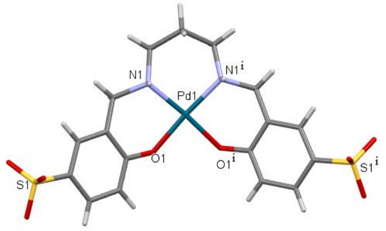

7’

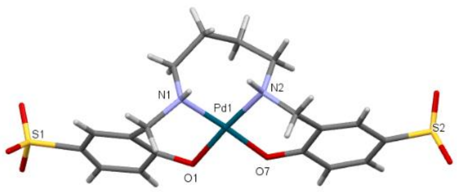

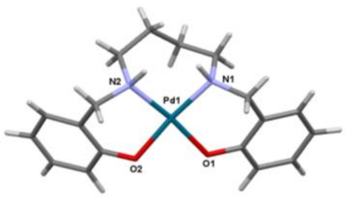

8’ **[Pd(BuHS)]**

Figure S36. Capped sticks views of K_2[Pd(PrHSS)], (**7’**) [Symmetry code: (i) +x, 1/2–y, +z] and K_2[Pd(BuHSS)], (**8’**) (solvents and the flexible polymer chains –linked together by K^+ and water molecules– are omitted for clarity), and capped sticks view of [Pd(BuHS)], [S15].

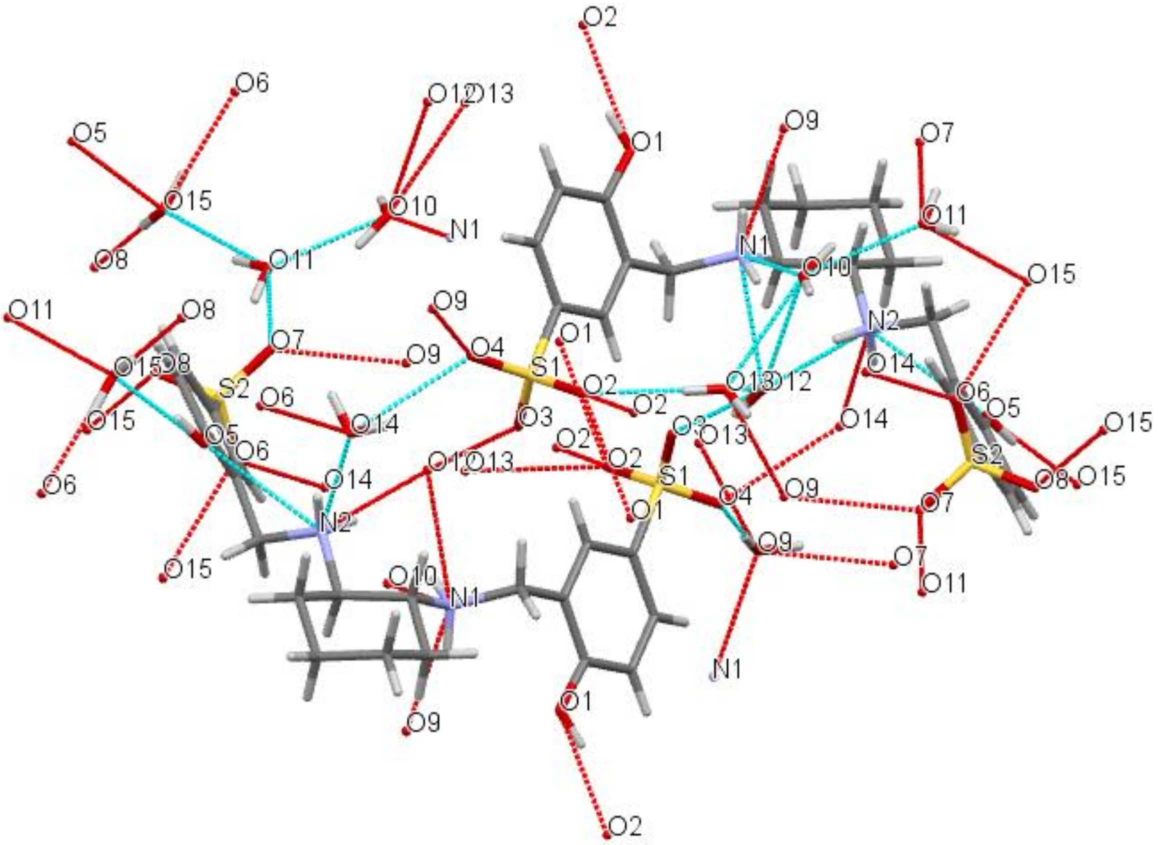

Figure S28a. Partial packing view of **5b**×7 H_2O with hydrogen bonds.

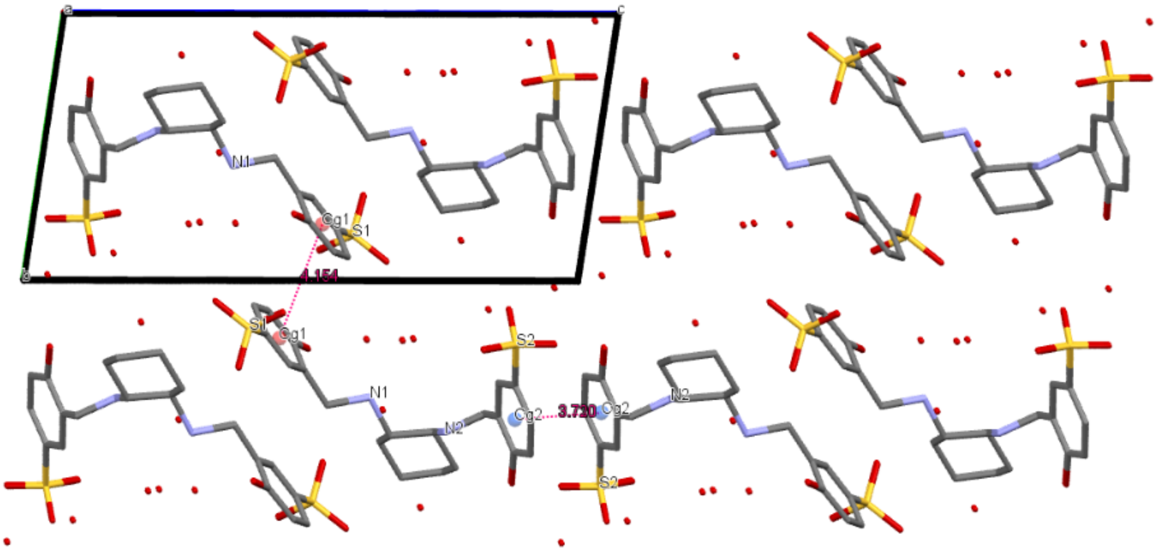

Figure S28b. Partial packing view of **5b**×7 H_2O with π–π interactions. All hydrogen atoms are omitted for clarity. [Cg_1: C1C2C3C4C5C6 and Cg_1: C15C16C17C18C19C20, symmetry codes and interaction lengths: Cg_1-Cg_1 2–x,2–y,1–z, 4.154(2)Å; Cg_2 - Cg_2, 1–x,1–y,–z 3.720(2) Å]

Table S5. Hydrogen bonds in **5b**×7 H_2O

D – H....A	D - H	H...A	D...A	D – H...A
O1 – H1 .. O2$^{(i)}$	0.8200	1.9400	2.719(4)	159.00
N1 – H1A .. O9$^{(ii)}$	0.8900	2.0300	2.876(5)	159.00
N1 – H1B .. O10$^{(iii)}$	0.8900	2.2000	2.946(5)	142.00
N1 – H1B .. O12$^{(ii)}$	0.8900	2.2600	2.976(5)	137.00
N2 – H2A .. O5	0.8900	2.5600	3.068(4)	117.00
N2 – H2A .. O14	0.8900	1.9900	2.816(6)	155.00
N2 – H2B .. O12$^{(iii)}$	0.8900	1.9600	2.802(5)	157.00
O5 – H5 .. O15	0.8200	1.8800	2.694(4)	170.00
O9 – H9C .. O4	0.8500	1.8900	2.745(5)	178.00
O9 – H9D .. O7$^{(iv)}$	0.8500	1.9600	2.779(4)	161.00
O10 – H10D .. O11	0.8500	2.1500	2.781(7)	131.00
O11 – H11C .. O15$^{(v)}$	0.8500	2.1500	2.969(6)	162.00
O12 – H12C .. O13	0.8500	1.8900	2.738(5)	177.00
O12 – H12D .. O3	0.8500	1.8900	2.690(4)	156.00
O13 – H13B .. O2$^{(iii)}$	0.8500	2.1600	3.003(4)	173.00
O14 – H14D .. O4$^{(iii)}$	0.8500	2.1800	2.920(5)	146.00
O15 – H15B .. O6$^{(iii)}$	0.8500	1.9900	2.829(4)	167.00
C4 – H4 .. O4	0.9300	2.5800	2.932(5)	103.00
C7 – H7B .. O3$^{(iii)}$	0.9700	2.3800	3.328(5)	166.00
C13 – H13 .. O7$^{(i)}$	0.9800	2.5400	3.498(5)	167.00
C14 – H14B .. O11$^{(iii)}$	0.9700	2.6000	3.540(6)	164.00
C20 – H20 .. O6	0.9300	2.5700	2.934(5)	103.00

Symmetry codes: (i)1+x,y,z (ii) 2–x,2–y,1–z (iii)1–x,1–y,1–z (iv) 1–x,2–y,1–z (v) x,y,1+z

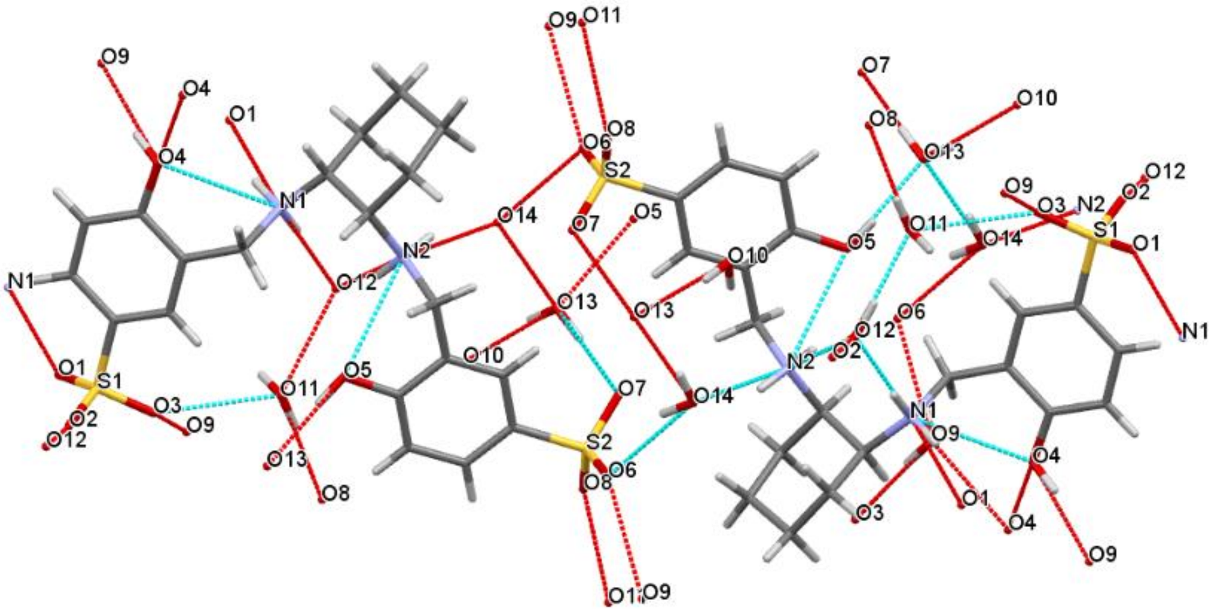

Figure S29a. Partial packing view of **5cb**×6 H_2O with hydrogen bonds.

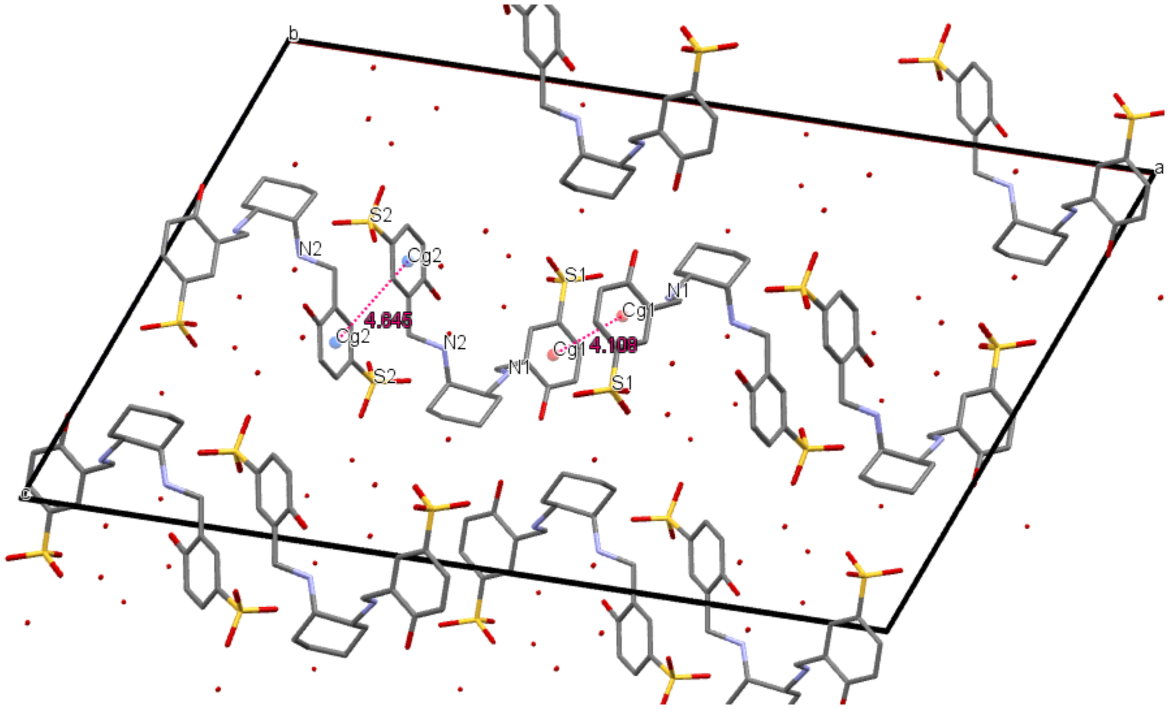

Figure S29b. Partial packing view of **5cb**×6 H_2O with π–π interactions. All hydrogen atoms are omitted for clarity. [Cg_1: C1C2C3C4C5C6 and Cg_1: C15C16C17C18C19C20. Symmetry codes and bond lengths: Cg_1-Cg_1 1–x,1–y,1–z, 4.1082(19)Å; Cg_2 - Cg_2, 1/2–x,3/2–y,1–z 4.645(2) Å]

Table S6. Hydrogen bonds in **5cb**×6 H_2O

D — H....A	D - H	H...A	D...A	D - H...A
N1 – H1A .. O1$^{(i)}$	0.8900	2.0000	2.849(4)	158.00
N1 – H1B .. O12	0.8900	1.9000	2.770(3)	168.00
N2 – H2A .. O14$^{(ii)}$	0.8900	1.9300	2.779(4)	159.00
N2 – H2B .. O5	0.8900	2.5000	3.001(4)	116.00
N2 – H2B .. O12	0.8900	2.0700	2.907(4)	156.00
O4 – H4 .. O9$^{(iii)}$	0.8200	1.8700	2.680(4)	169.00
O5 – H5 .. O13	0.8200	1.9100	2.666(4)	153.00
O9 – H9C .. O3$^{(iv)}$	0.8500	1.9200	2.769(4)	171.00
O9 – H9D .. O6$^{(v)}$	0.8500	2.0600	2.856(4)	156.00
O10 – H10C .. O13$^{(ii)}$	0.8500	2.3800	2.767(8)	109.00
O10 – H10D .. O3$^{(ii)}$	0.8500	2.5800	3.081(8)	118.00
O11 – H11D .. O8$^{(vi)}$	0.8500	1.9400	2.784(5)	172.00
O12 – H12C .. O2$^{(ii)}$	0.8500	1.9300	2.748(4)	161.00
O12 – H12D .. O11	0.8500	1.8700	2.718(4)	176.00
O13 – H13A .. O10$^{(vii)}$	0.8500	1.9500	2.767(8)	161.00
O13 – H13B .. O7$^{(vii)}$	0.8500	2.0400	2.831(5)	155.00
O14 – H14C .. O13	0.8500	2.0000	2.842(5)	170.00
O14 – H14D .. O6$^{(v)}$	0.8500	2.1000	2.897(5)	155.00
C6 – H6 .. O3	0.9300	2.5500	2.900(4)	103.00
C7 – H7A .. O9	0.9700	2.4600	3.402(4)	163.00
C9 – H9B .. O1$^{(i)}$	0.9700	2.5500	3.336(5)	138.00
C11 – H11B .. O6$^{(viii)}$	0.9700	2.5600	3.367(5)	141.00
C14 – H14B .. O7$^{(viii)}$	0.9700	2.5900	3.521(4)	162.00
C18 – H18 .. O8	0.9300	2.6000	2.940(6)	102.00

Symmetry codes: (i) 1–x,1–y,1–z, (ii) x,1+y,z, (iii) 1–x,y,3/2–z, (iv) x,1–y,1/2+z, (v) 1/2–x,3/2–y,1–z, (vi) 1/2–x,–1/2+y,1/2–z, (vii) x,–1+y,z, (viii) 1/2–x,5/2–y,1–z

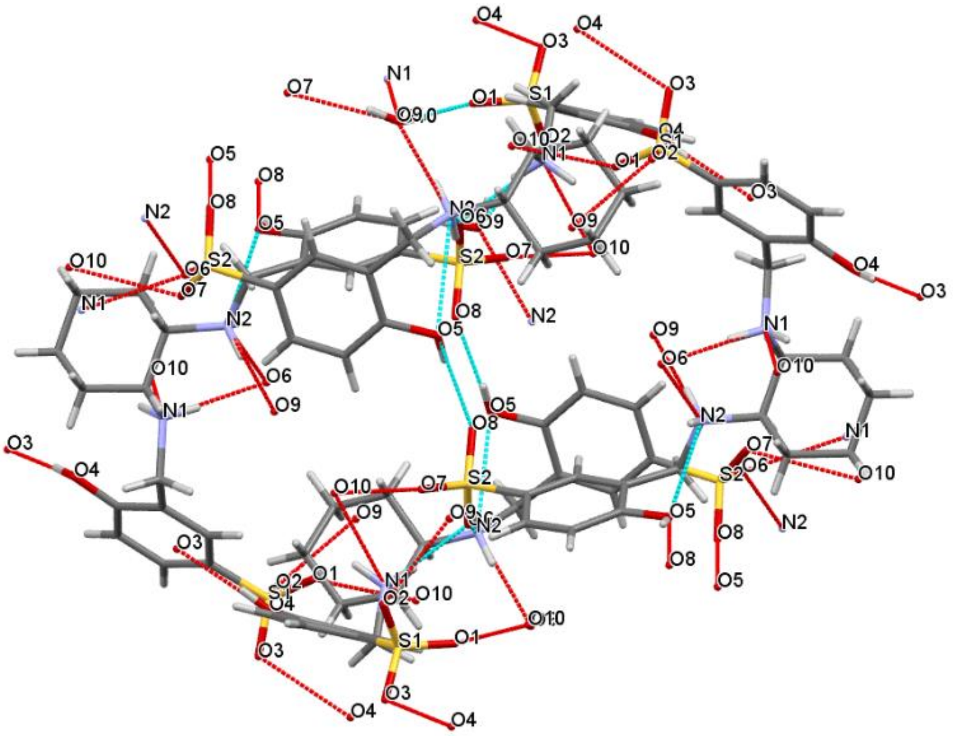

Figure S30a. Partial packing view of **5ca**×2 H_2O with hydrogen bonds.

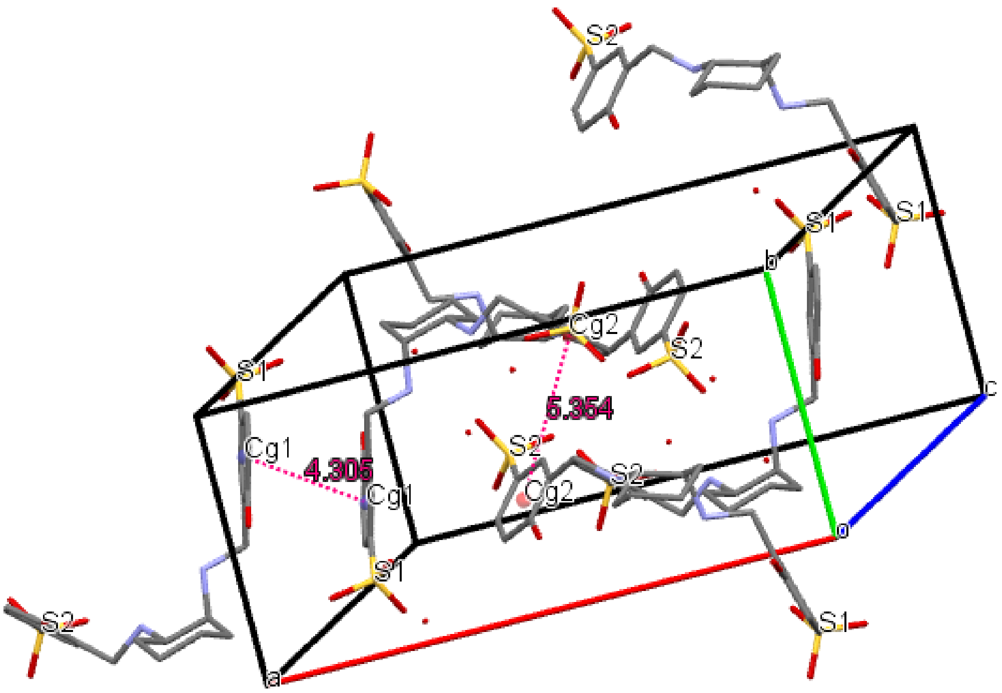

Figure S30b. Partial packing view of **5ca** ×2 H_2O with very weak π–π interactions. All hydrogen atoms are omitted for clarity.

[Cg_1: C1C2C3C4C5C6 and Cg_1: C15C16C17C18C19C20, symmetry codes and bond lengths: Cg_1-Cg_1 2–x,1–y,1–z, 4.305(4)Å; Cg_2 - Cg_2, 1–x,1/2+y,1/2–z 5.345(4) Å]

Table S7. Hydrogen bonds in **5ca**×2 H_2O

D – H....A	D - H	H...A	D...A	D – H...A
N1 – H1A .. O6$^{(i)}$	0.8900	1.9200	2.807(7)	173.00
N1 – H1B .. O4	0.8900	2.5700	3.090(7)	118.00
N1 – H1B .. O10$^{(ii)}$	0.8900	2.1300	2.854(7)	138.00
N2 – H2A .. O9$^{(iii)}$	0.8900	1.8700	2.745(8)	169.00
N2 – H2B .. O5	0.8900	2.5000	2.996(8)	116.00
N2 – H2B .. O6$^{(i)}$	0.8900	2.0800	2.749(6)	131.00
O4 – H4 .. O3$^{(ii)}$	0.8200	1.9300	2.750(6)	175.00
O5 – H5 .. O8$^{(iii)}$	0.8200	1.9200	2.688(7)	155.00
O9 – H9D .. O2	0.8500	2.0100	2.709(9)	139.00
O10 – H10C .. O1	0.8500	1.9100	2.763(6)	177.00
O10 – H10D .. O7$^{(iv)}$	0.8500	2.0700	2.890(6)	160.00
C4 – H4A .. O3$^{(v)}$	0.9300	2.5400	3.392(8)	153.00
C6 – H6 .. O1	0.9300	2.5400	2.908(7)	104.00
C7 – H7B .. O3$^{(vi)}$	0.9700	2.5500	3.516(8)	178.00
C8 – H8 .. O2$^{(iii)}$	0.9800	2.4100	3.364(8)	164.00
C9 – H9A .. O3$^{(vi)}$	0.9700	2.4000	3.340(9)	163.00
C14 – H14A .. O5$^{(vii)}$	0.9700	2.5300	3.276(8)	134.00

Symmetry codes: (i) 1-x,-1/2+y,1/2-z (ii) x,1/2-y,1/2+z (iii) x,1/2-y,-1/2+z (iv) 1-x,1-y,-z (v) 2-x,-y,1-z (vi) 2-x,1-y,1-z (vii) 1-x,2-y,1-z

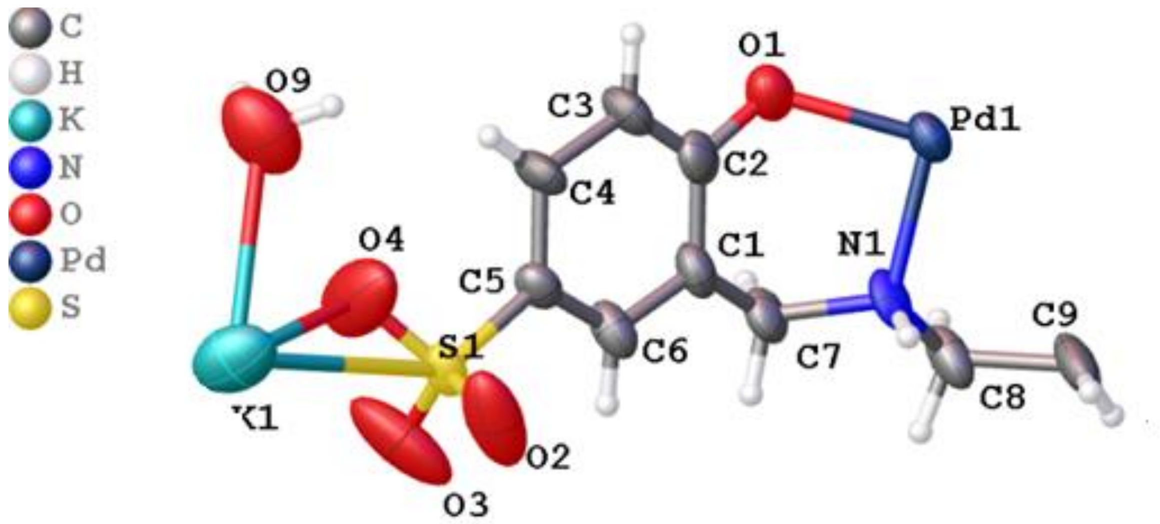

Figure S31. ORTEP diagram of the asymmetric unit of [Pd(PrHSS)]$^{2-}$ showing the atom labelling scheme. (Thermal ellipsoids are shown at a 50% probability level.)

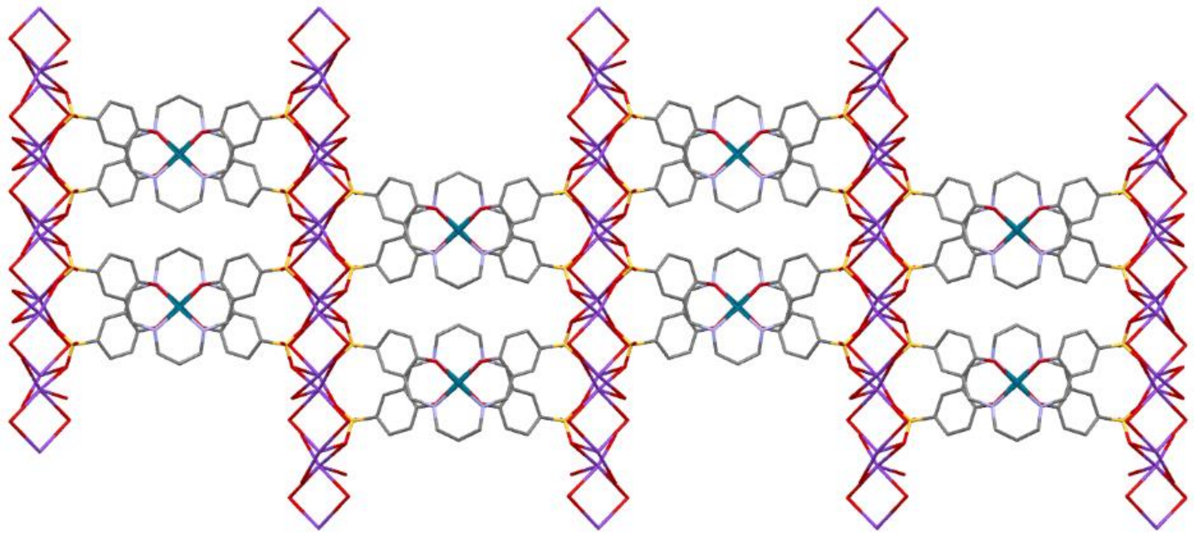

Figure S32. Packing view of $[Pd(PrHSS)]^{2-}$ along axis "**a**" (all hydrogen atoms are omitted for clarity.)

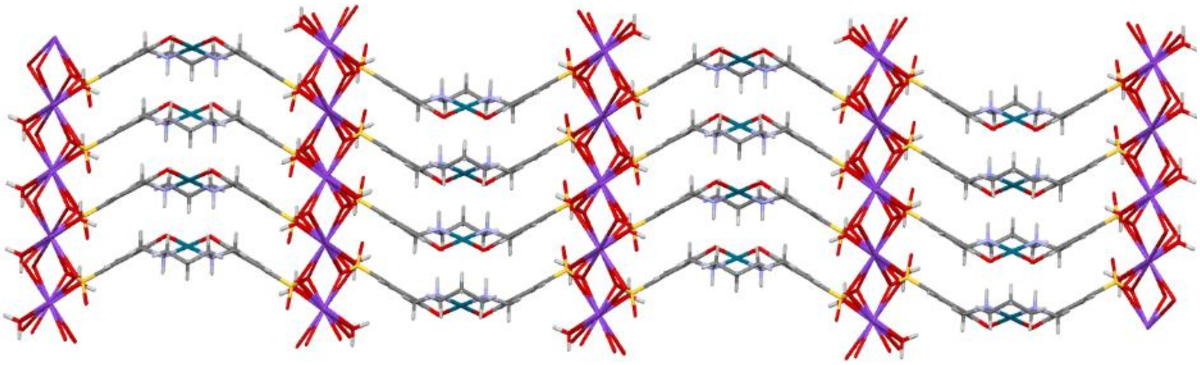

Figure S33. Packing view of $[Pd(PrHSS)]^{2-}$ along axis "c" (all hydrogen atoms are omitted for clarity.)

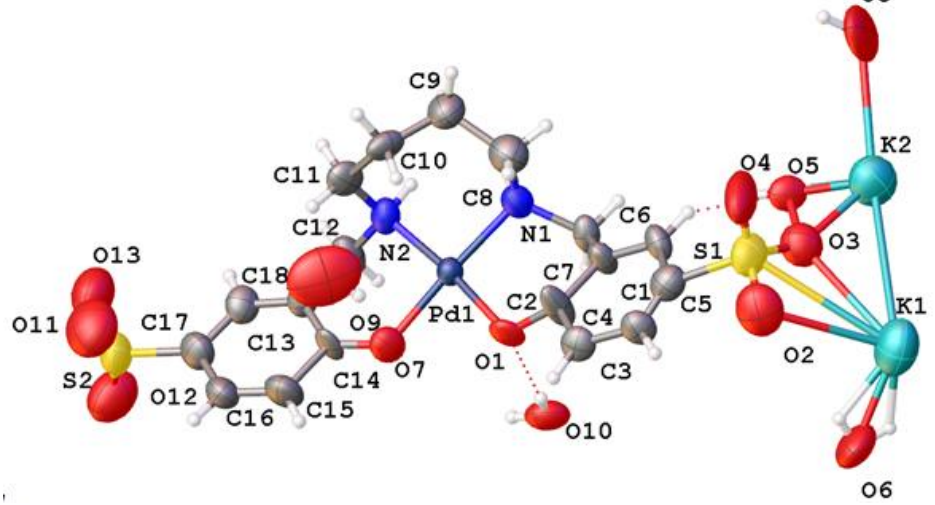

Figure S34. ORTEP diagram of the asymmetric unit of $[Pd(BuHSS)]^{2-}$ showing the atom labelling scheme.

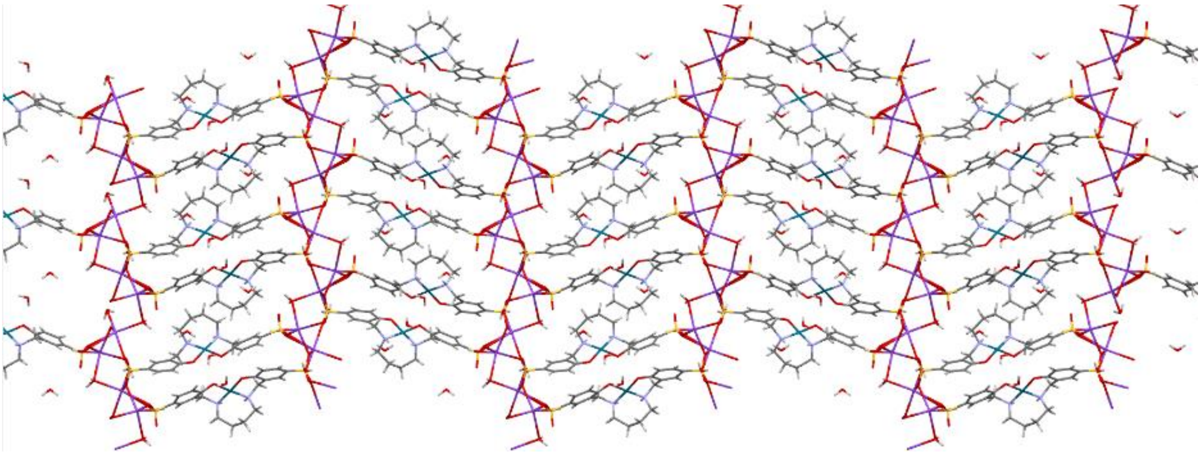

Figure S35. Partial packing view of $[Pd(BuHSS)]^{2-}$ along axis "**c**"

Table S8. Crystal data and details of measurements

	**1**×2H_2O	**2**×5.5H_2O	**3**	**4**×2 dmso× 2H_2O
Chemical formula	$C_{16}H_{20}N_2O_8S_2$, 2H_2O	2($C_{17}H_{22}N_2O_8S_2$), 11(H_2O)	$C_{18}H_{24}N_2O_8S_2$	$C_{28}H_{28}N_2O_8S_2$·2(C_2H_6OS), 2(H_2O)·
Formula weight	468.49	1091.14	460.51	776.93
Crystal size [mm]	0.09 × 0.11 × 0.15	0.12 × 0.17 × 0.12	0.05 × 0.09 × 0.11	0.07 × 0.11 × 0.14
T [K]	295.15	150	150	297
λ [Å]	Mo*K*α (λ = 0.71073)	Mo*K*α (λ = 0.71073)	Mo*K*α (λ = 0.71073)	Cu*K*α (λ = 1.54178)
Crystal system	monoclinic	orthorhombic	monoclinic	monoclinic
Space group	$P2_1/c$	*Fdd2*	$P2_1/c$	$P2_1/c$
Crystal habit, colour	block, colourless	block, colourless	block, colourless	block, colourless
a [Å]	10.1586(16)	23.3049(19)	9.0322(7)	10.0166(3)
b [Å]	14.056(2)	37.284(3)	12.7728(8)	10.3136(3)
c [Å]	7.3816(10)	11.6424(9)	9.4938(7)	17.4524(5)
α [°]	90	90	90	90
β [°]	107.615(6)		106.576(3)	98.804(2)
γ [°]	90		90	90
V [Å^3]	1004.6(3)	10116.2(14)	1049.75(13)	1781.72(9)
Z	2	8	2	2
ρ_{calcd} [g cm^{-3}]	1.549	1.433	1.457	1.448
μ [mm^{-1}]	0.324	0.278	0.302	3.006
2Θ range [°]	5.11 – 52.93	5.102 – 50.804	4.706 – 51.944	8.934 - 144.478
Index ranges	$-12 \le h \le 12$, $0 \le k \le 17$, $0 \le l \le 9$	$-28 \le h \le 28$, $-44 \le k \le 40$, $-14 \le l \le 13$	$-11 \le h \le 11$, $-15 \le k \le 15$, $-11 \le l \le 1$	$-12 \le h \le 12$, $-12 \le k \le 12$, $-21 \le l \le 21$
Total reflections	2043	15959	25254	12293
Unique reflections	2043	4521 [R_{int} = 0.069]	2041 [R_{int} = 0.093]	3474 [R_{int} = 0.0627]
Data/restraints/parameters	2043/0/159	4521/1/332	2041/0/137	3474/0/232
Final R indices [$F^2 > 2\sigma(F^2)$]	0.0713	0.0799	0.0479	0.0548
R indices (all data, w$R(F^2)$)	0.1969	0.2299	0.1460	0.1545
Goodness of fit (GOF) on F^2	1.047	1.056	1.109	1.047
$\Delta\rho_{max}/\Delta\rho_{min}$ [eÅ^{-3}]	0.68/–0.54	1.84/-0.51	0.51/-0.31	0.50/-0.45
CCDC	2020275	2020276	2020277	2020278

	**5b**×7H_2O	**5ca**×2H_2O	**5cb**×6H_2O	**7** K_2[Pd(PrHSS)]	**8** K_2[Pd(BuHSS)]
Chemical formula	$C_{20}H_{26}N_2O_8S_2$, 7H_2O	$C_{20}H_{36}N_2O_8S_2$, 2 H_2O	$C_{20}H_{36}N_2O_8S_2$, 6 H_2O	$C_{17}H_{22}K_2N_2O_{10}PdS_2$	$C_{18}H_{26}K_2N_2O_{13}PdS_2$
Formula weight	612.66	522.58	594.64	663.08	727.13
Crystal size [mm]	0.09 × 0.14 × 0.18	0.257 × 0.143 × 0.117	0.14 × 0.19 × 0.32	0.22 × 0.15 × 0.079	0.21 × 0.17 × 0.11
T [K]	193.15	295.17	298.15	297.22	149.99
λ [Å]	Mo*K*α (λ = 0.71073)	CuKα (λ = 1.54178)	MoKα (λ = 0.71073)	Mo*K*α (λ = 0.71073)	Mo*K*α (λ = 0.71073)
Crystal system	triclinic	monoclinic	monoclinic	orthorhombic	orthorhombic
Space group	$P\bar{1}$	$P2_1/c$	$C2/c$	*Pmna*	$P2_12_12_1$
Crystal habit, colour	colorless, plate	block, colourless	block, colourless	yellow, plate	yellow, plate
a [Å]	8.4773(5)	18.1286(19)	34.475(2)	7.9909(14)	7.1625(2)
b [Å]	9.8170(7)	8.7933(9)	8.2430(6)	33.129(5)	10.7350(5)
c [Å]	18.7465(13)	15.3451(16)	20.9017(16)	9.038(3)	36.4200(10)
α [°]	96.376(3)	90	90	90	90
β [°]	93.943(2)	110.197(6)	111.517	90	90
γ [°]	111.362(2)	90	90	90	90
V [Å^3]	1433.85(17)	2295.8(4)	5528.8(7)	2392.5(9)	2800.31(15)
Z	2	4	8	4	4
ρ_{calcd} [g cm^{-3}]	1.419	1.512	1.408	1.841	1.725
μ [mm^{-1}]	0.257	2.639	0.260	1.354	1.173
2Θ range [°]	4.404 – 50.784	5.194 – 136.912	5.08 –52.868	4.672 - 52.118	5.796 - 59.456
Index ranges	$-10 \leq h \leq 10$, $-11 \leq k \leq 11$, $-22 \leq l \leq 22$	$-19 \leq h \leq 16$, $-10 \leq k \leq 10$, $-13 \leq l \leq 18$	$-42 \leq h \leq 42$, $-10 \leq k \leq 10$, $-26 \leq l \leq 26$	$-9 \leq h \leq 9$, $-40 \leq k \leq 40$, $-11 \leq l \leq 11$	$-9 \leq h \leq 9$, $-14 \leq k \leq 14$, $-50 \leq l \leq 50$
Total reflections	64805	12741	32936	49746	247929
Unique reflections	5265 [R_{int} = 0.0799]	3823 [R_{int} = 0.1040]	5668 [R_{int} =0.0432]	2399 [R_{int} = 0.1091]	24729
Data/restraints/parameters	5265/0/375	3823/0/316	5668/0/363	2399/150/157	24729/343/357
Final R indices [F^2> 2σ(F^2)]	0.0669	0.0895	0.0654	0.2040	0.1491
R indices (all data, wR(F^2))	0.1976	0.2800	0.1757	0.5450	0.4074
Goodness of fit (GOF) on F^2	1.037	1.057	1.053	2.824	1.711
$\Delta\rho_{max}/\Delta\rho_{min}$ [eÅ^{-3}]	1.40/-0.45	0.77/-0.47	0.86/-0.51	15.56/-6.07	3.84/-2.62
CCDC	2020279	2020280	2020281	2020282	2020437

References for the crystallographic section

[S1] Bruker (2017). *APEX3* and *SAINT*. Bruker AXS Inc., Madison, Wisconsin, USA, **2017**.

[S2] Sheldrick, G.M. SHELXT—Integrated space-group and crystal-structure determination. *Acta Crystallogr. Sect. Found. Adv.* **2015**, *71*, 3–8.

[S3] Sheldrick, G.M. A short history of SHELX. *Acta Crystallogr.* A **2008**, *64*, 112–122.

[S4] Dolomanov, O.V.; Bourhis, L.J.; Gildea, R.J.; Howard, J.A.K.; Puschmann, H. $OLEX^2$: A complete structure solution, refinement and analysis program. *J. Appl. Cryst*. **2009**, *42*, 339–341.

[S5] Farrugia, L. J. WinGX and ORTEP for Windows: an update *J. Appl. Cryst.* **2012**, *45*, 849–854.

[S6] Spek, A.L; *Acta Cryst.* **2020**, E76, 1-11.

[S7] Macrae, C.F.; Bruno, I.J.; Chisholm, J.A.; Edgington, P.R.; McCabe, P.; Pidcock, E.; Rodriguez-Monge, L.; Taylor, R.; Streek, J.V.D.; Wood, P.A. Mercury CSD 2.0—New features for the visualization and investigation of crystal structures. *J. Appl. Cryst.* **2008**, *41*, 466–470.

[S8] Westrip, S.P. publCIF: Software for editing, validating and formatting crystallographic information files. *J. Appl. Crystallogr.* **2010**, *43*, 920–925.

[S9] Voronova, K.; Purgel, M.; Udvardy, A.; Bényei, A. C.; Kathó, Á.; Joó, F. Hydrogenation and Redox Isomerization of Allylic Alcohols Catalyzed by a New Water-Soluble Pd–tetrahydrosalen Complex. *Organometallics* **2013**, *32* 4391–4401.

[S10] Singh, U.P.; Tomar, K.; Kashyap, S. Interconversion of host–guest components in supramolecular assemblies of polycarboxylic acids and reduced Schiff bases. *Struct Chem* **2016**, *27*, 1027–1040.

[S11] Voronova, K.; Homolya, L.; Udvardy, A.; Bényei, A. C.; Joó, F. Pd-tetrahydrosalan-type complexes as catalysts for Sonogashira couplings in water: Efficient greening of the procedure *ChemSusChem* **2014**, *7* 2230–2239.

[S12] Hoshina, G.; Ohba, S.; Tsuchimoto, M. (RS,SR)-N,N'-Bis(2-hydroxybenzyl)-1,2-diphenylethylenediamine *Acta Cryst.* **1999,** C55, IUC9900030.

[S13] Cannadine, J. C.; Corden, J. P.; Errington, W.; Moore, P.; Wallbridge, M.G.H. Two Schiff Base Ligands Derived from 1,2-Diaminocyclohexane *Acta Cryst.* **1996**, C52, 1014–1017.

[S14] Muthuraman, M.; Nicoud, J.-F.; Masse, R., Desiraju, G. R. Crystal structure of *N,N'*-di-5-nitrosalicylidene-(*R*,*S*)-l,2-cyclohexanediamine, $C_{20}H_{20}N_4O_6$ *Z. Kristallogr.* **2001**, NCS 216 383–384.

[S15] Pillai, M. R. A.; Schlemper, E.O. A palladium(II) complex of a tetradentate amine-phenol ligand, *J. Chem. Cryst.* **1994,** *24*, 129-132.

[S16] Bian, H. D.; Wang, J.; Wei, Y.; Tang, J.; Huang, F. P.; Yao, D., Yu, Q.; Liang, H. Superoxide dismutase activity studies of Mn(III)/Cu(II)/Ni(II) complexes with Schiff base ligands. *Polyhedron*, **2015**, *20*, 147–153.

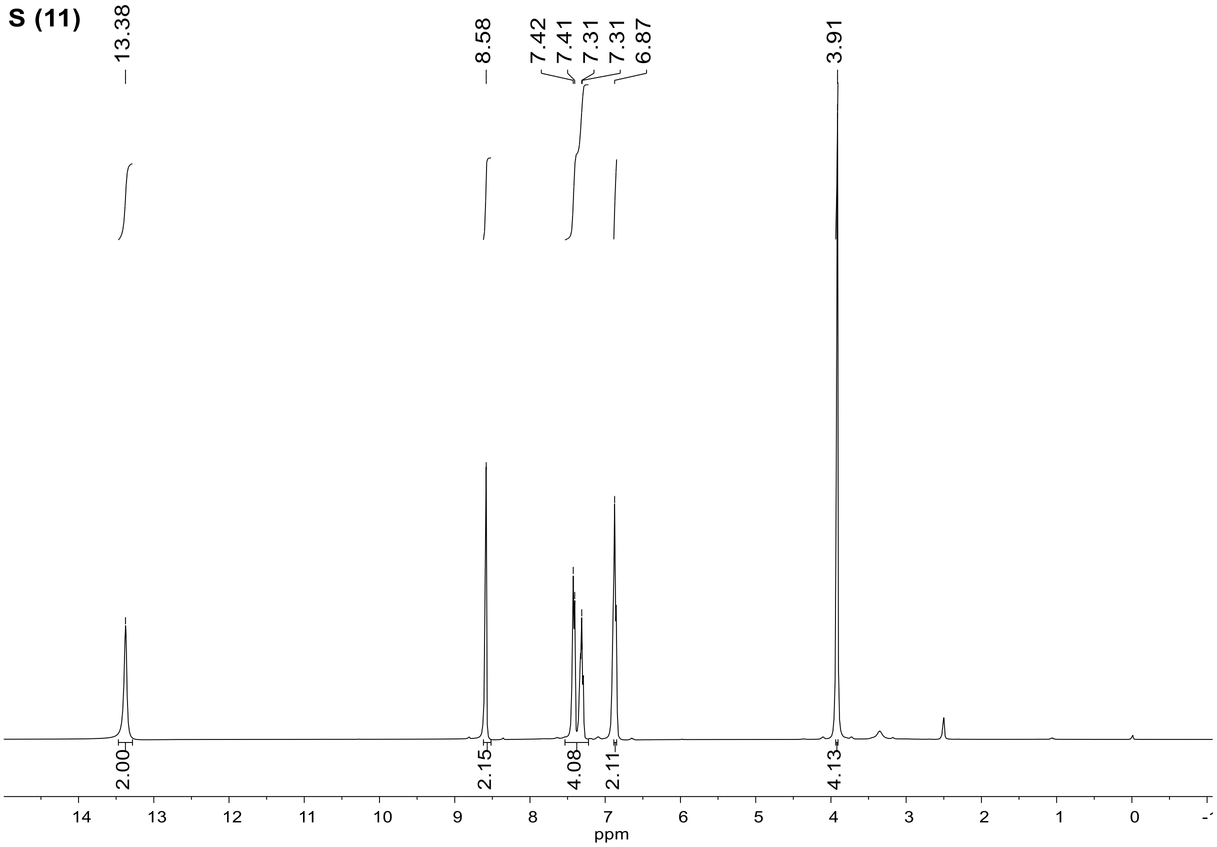

Figure S37. ^{1}H NMR spectrum of **S (11)** (360 MHz, 298K, d^{6}-dmso)

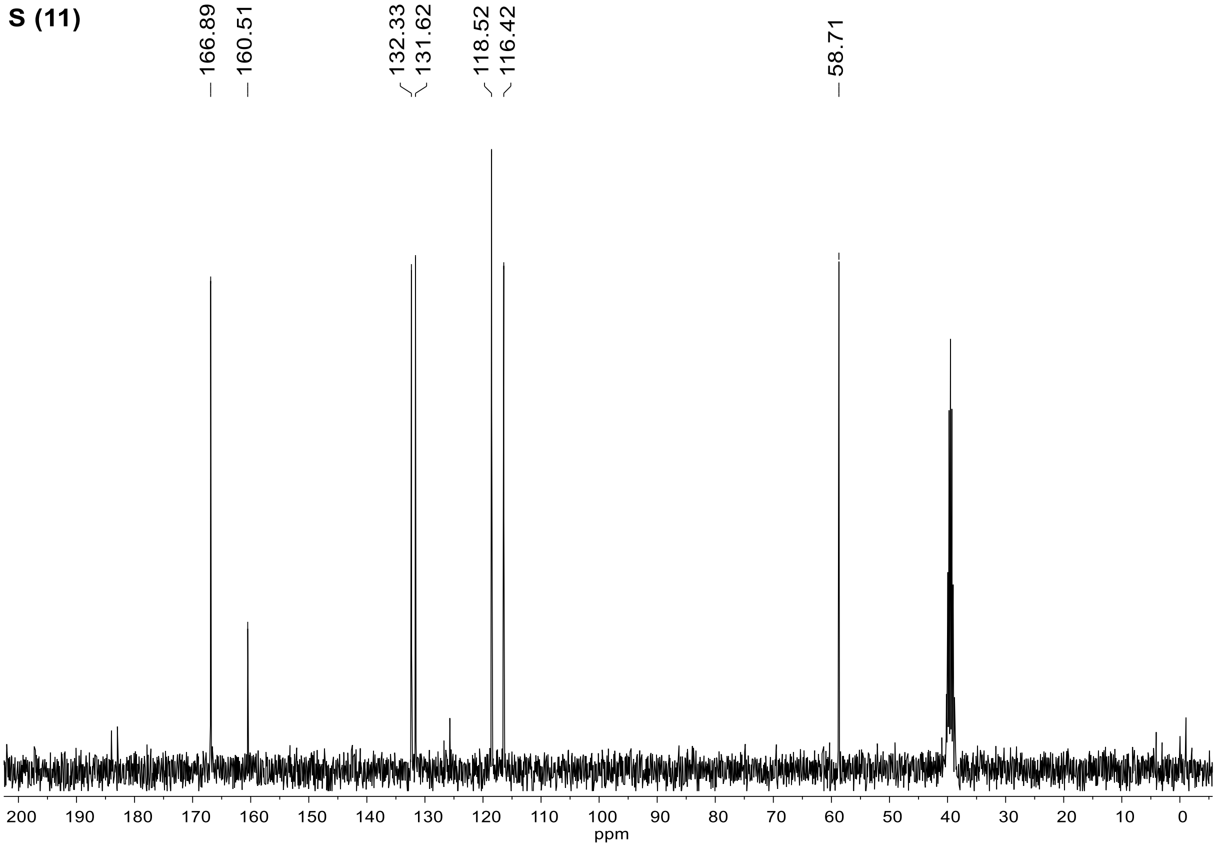

Figure S38. $^{13}C\{^{1}H\}$ NMR spectrum of **S (11)** (90 MHz, 298K, d^{6}-dmso)

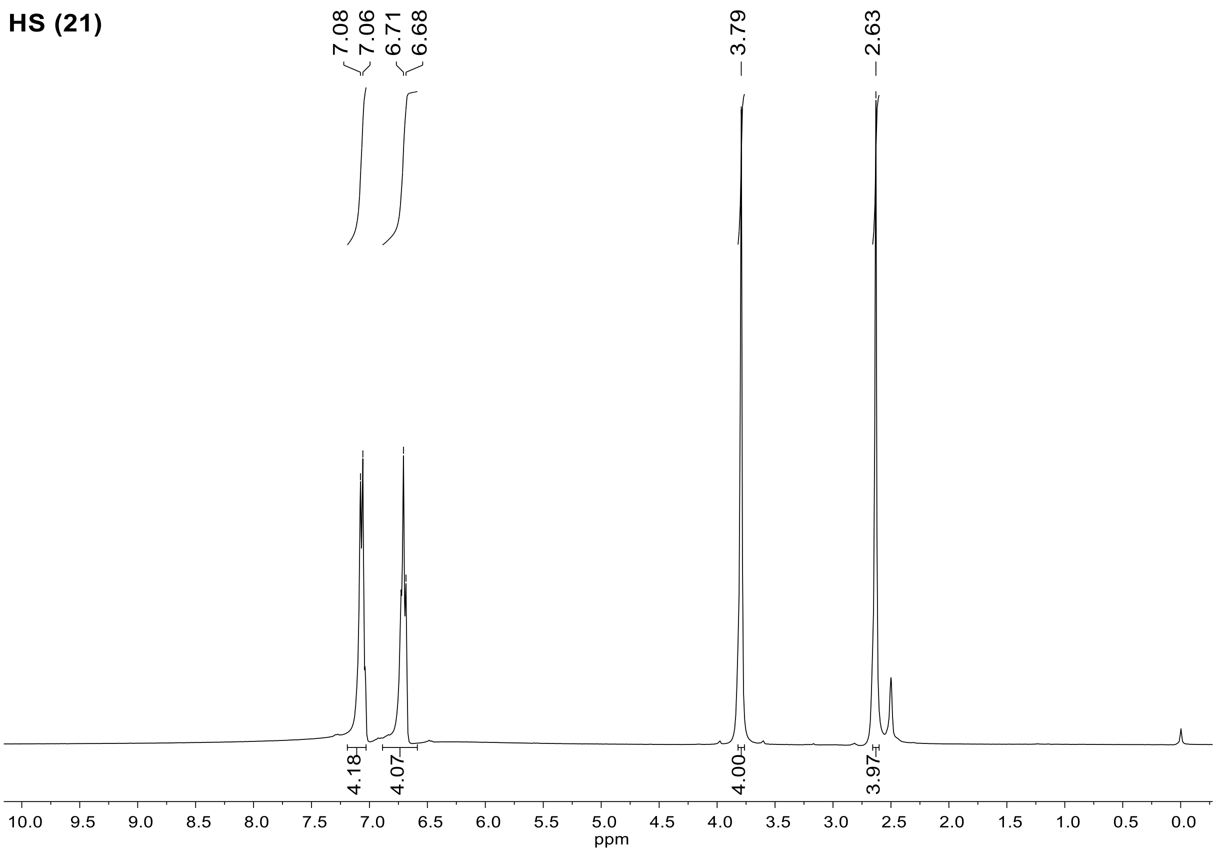

Figure S39. ^{1}H NMR spectrum of **HS (21)** (360 MHz, 298K, d^6-dmso)

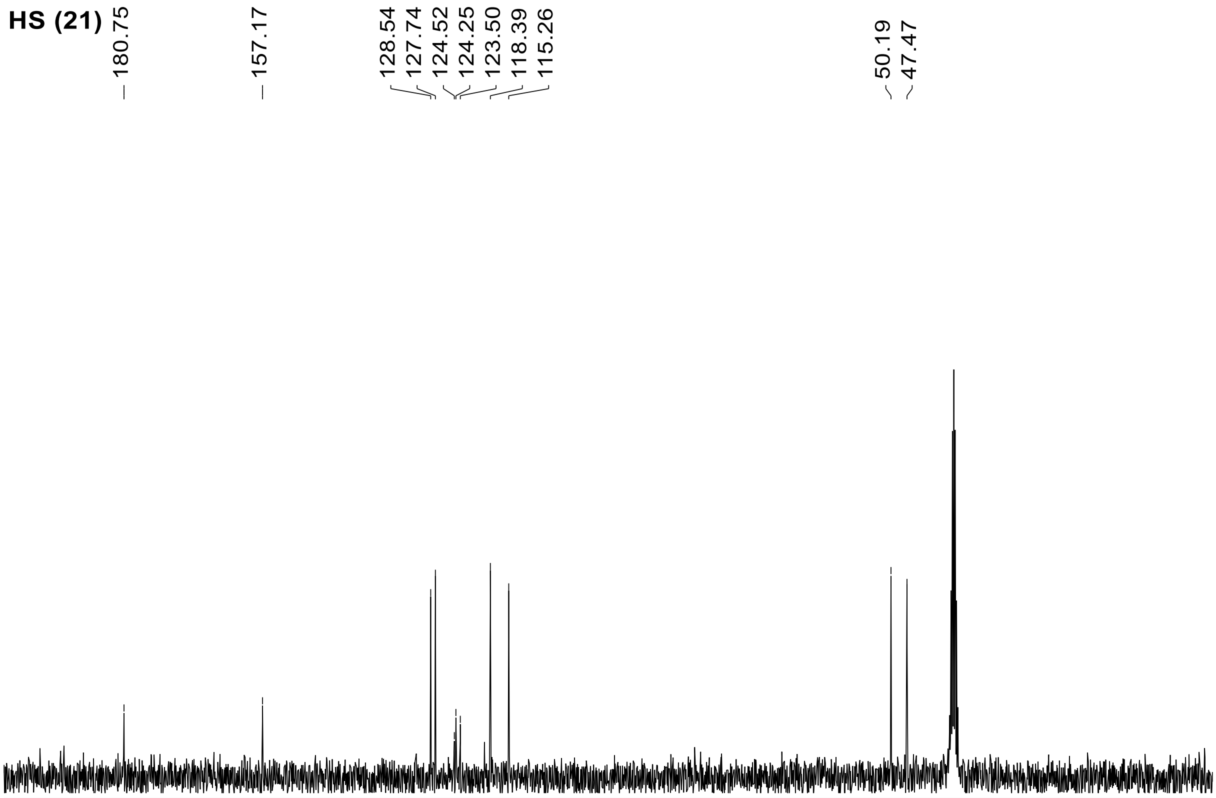

Figure S40. $^{13}C\{^1H\}$ NMR spectrum of **HS (21)** (90 MHz, 298K, d^6-dmso)

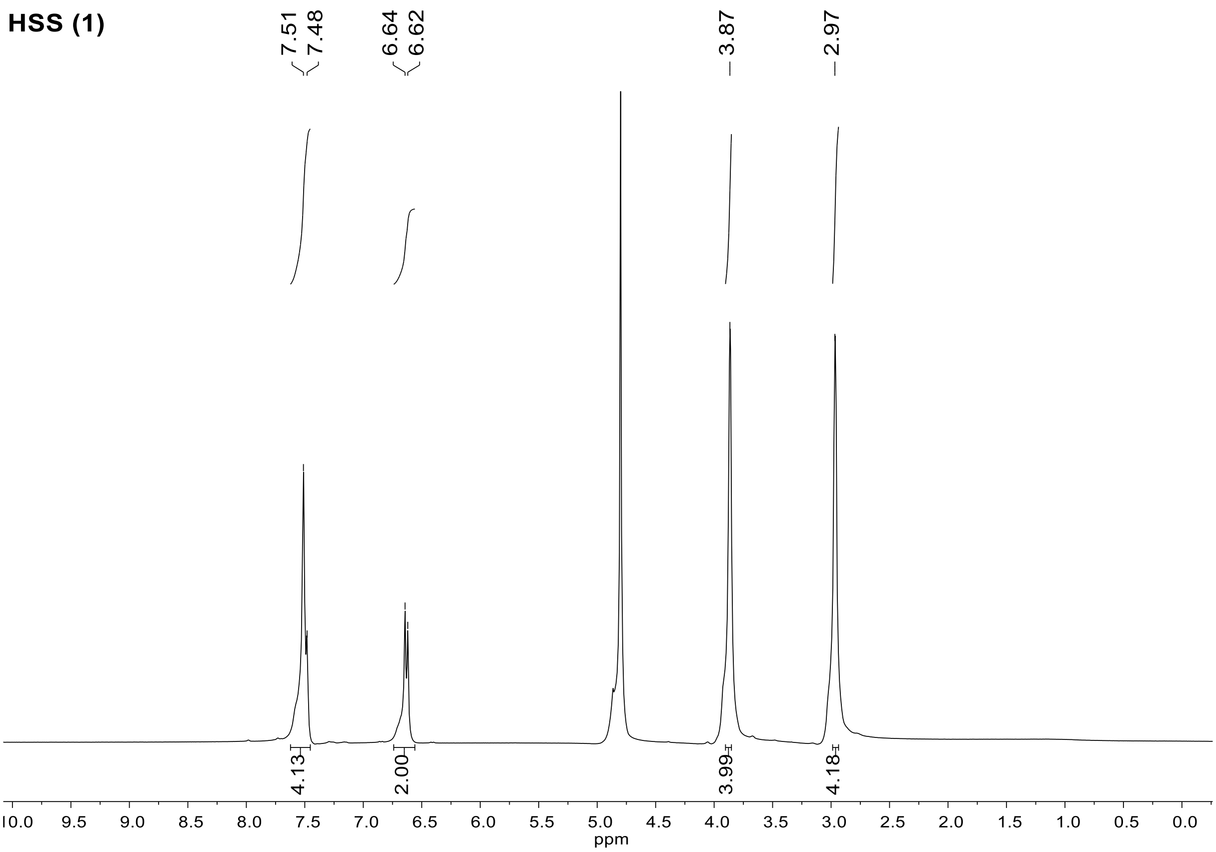

Figure S41.A. ^{1}H NMR spectrum of **HSS (1)** (360 MHz, 298K, D_2O)

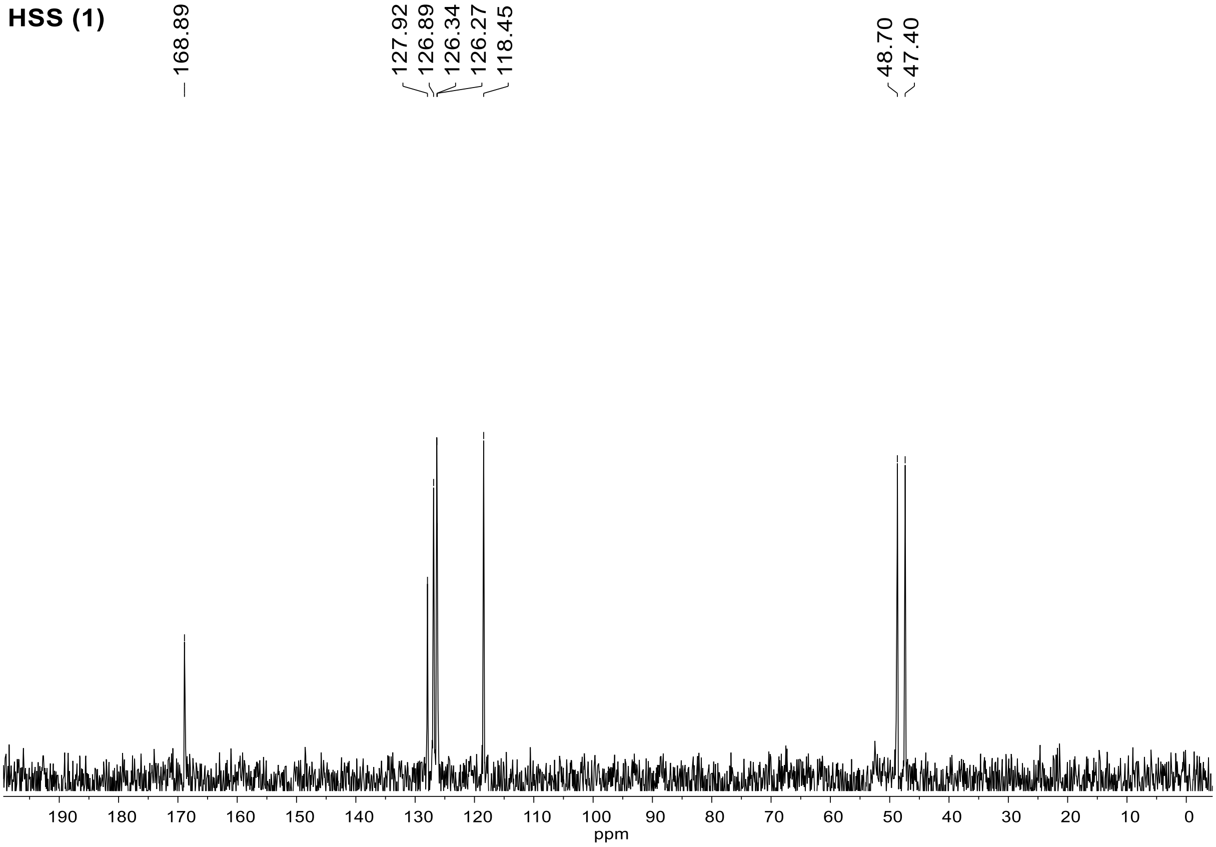

Figure S42. $^{13}C\{^{1}H\}$ NMR spectrum of **HSS (1)** (90 MHz, 298K, D_2O)

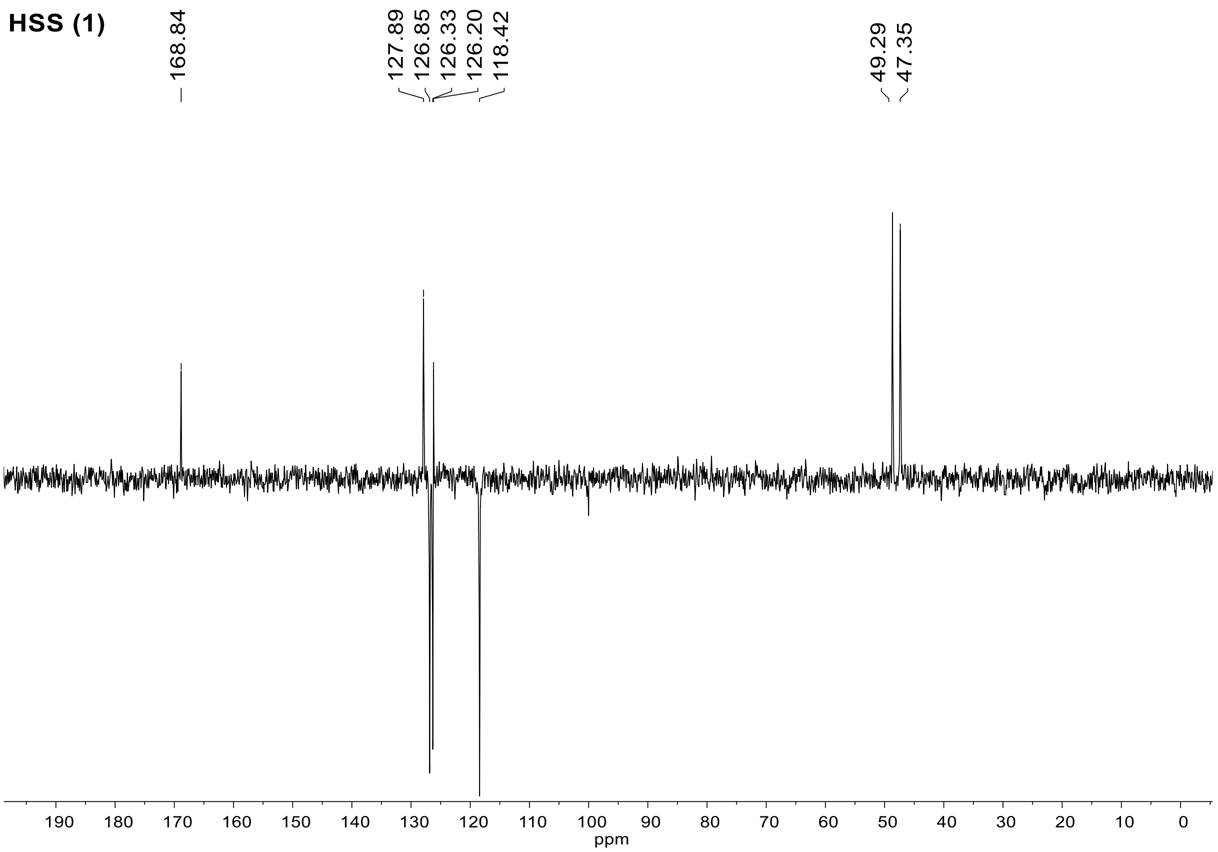

Figure S43. $^{13}C\{^{1}H\}$ NMR spectrum of **HSS (1)** (90 MHz, 298K, D_2O)

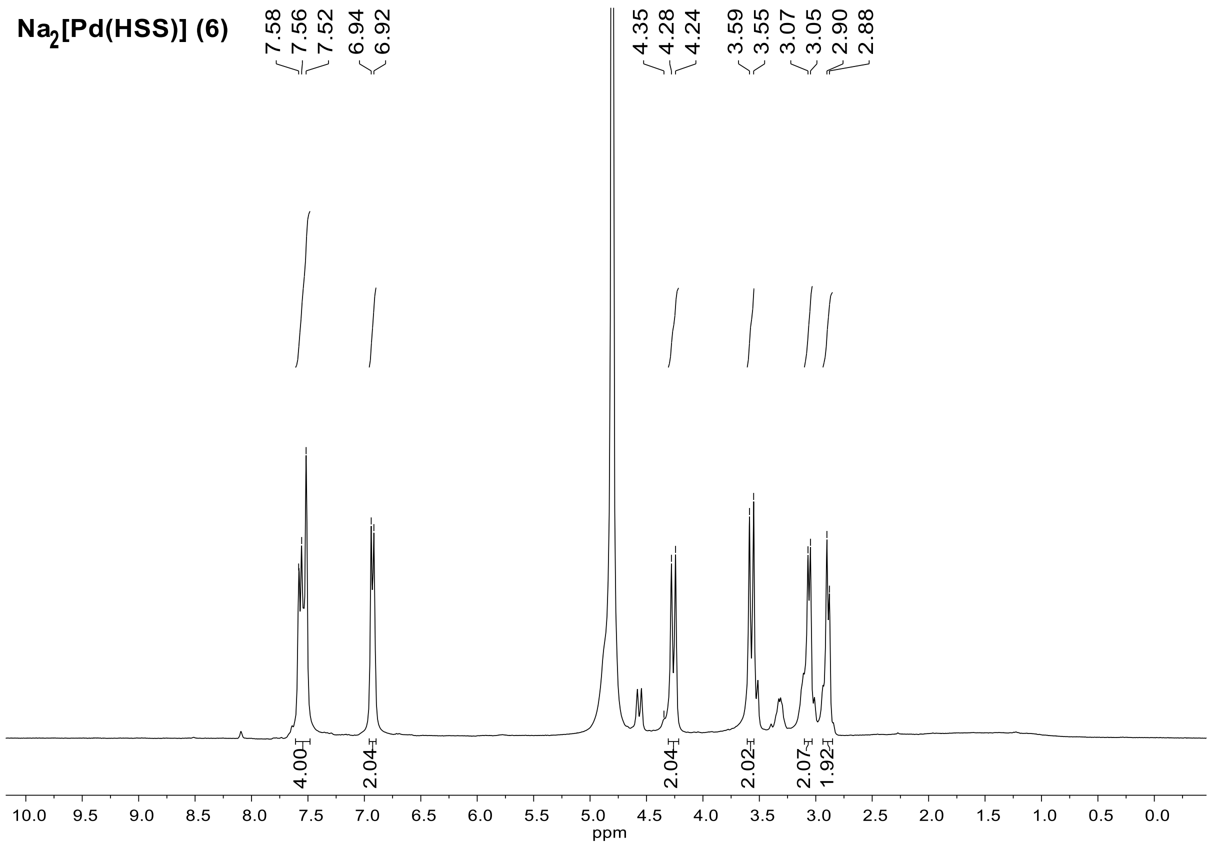

Figure S44.A. ^{1}H NMR spectrum of **Na_2[Pd(HSS)] (6)** (360 MHz, 298K, D_2O)

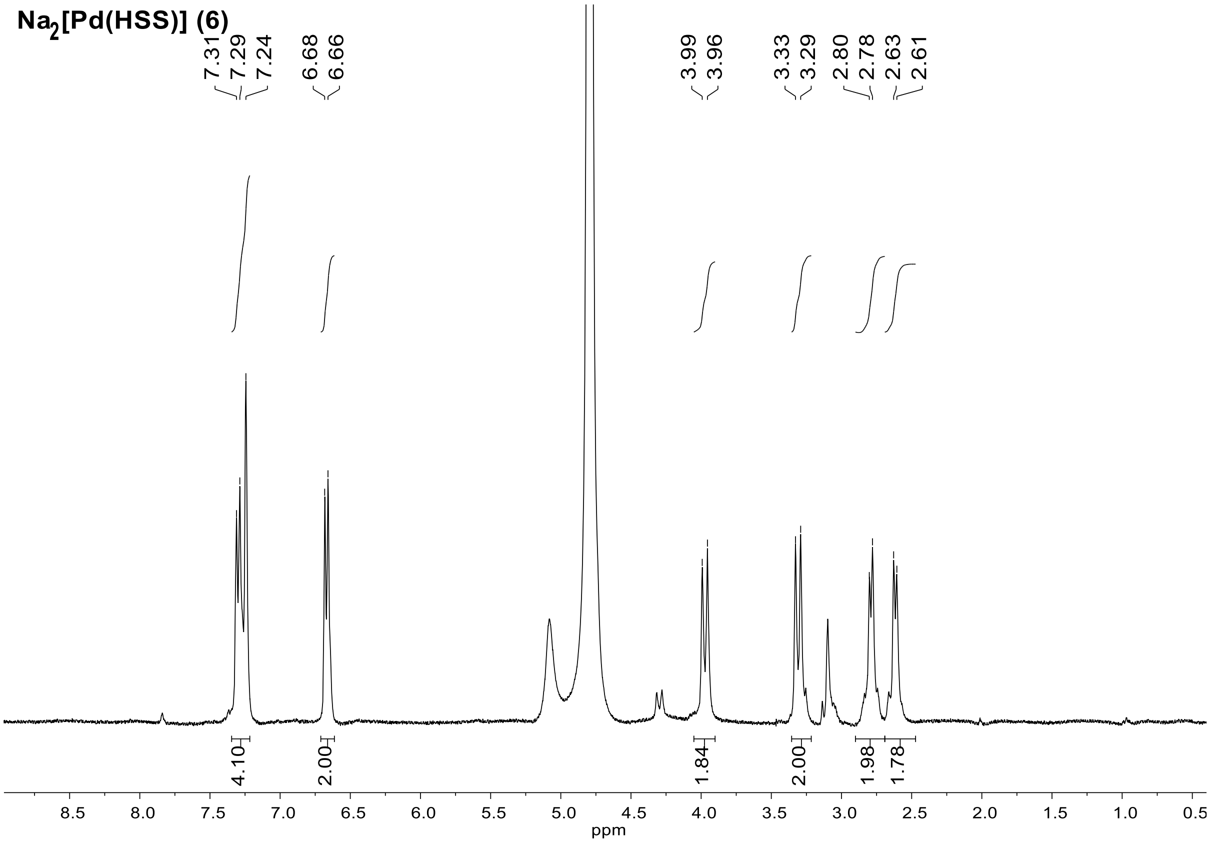

Figure S44.B. ^{1}H NMR spectrum of **Na_2[Pd(HSS)] (6)** (360 MHz, 268K, D_2O+CD_3OD)

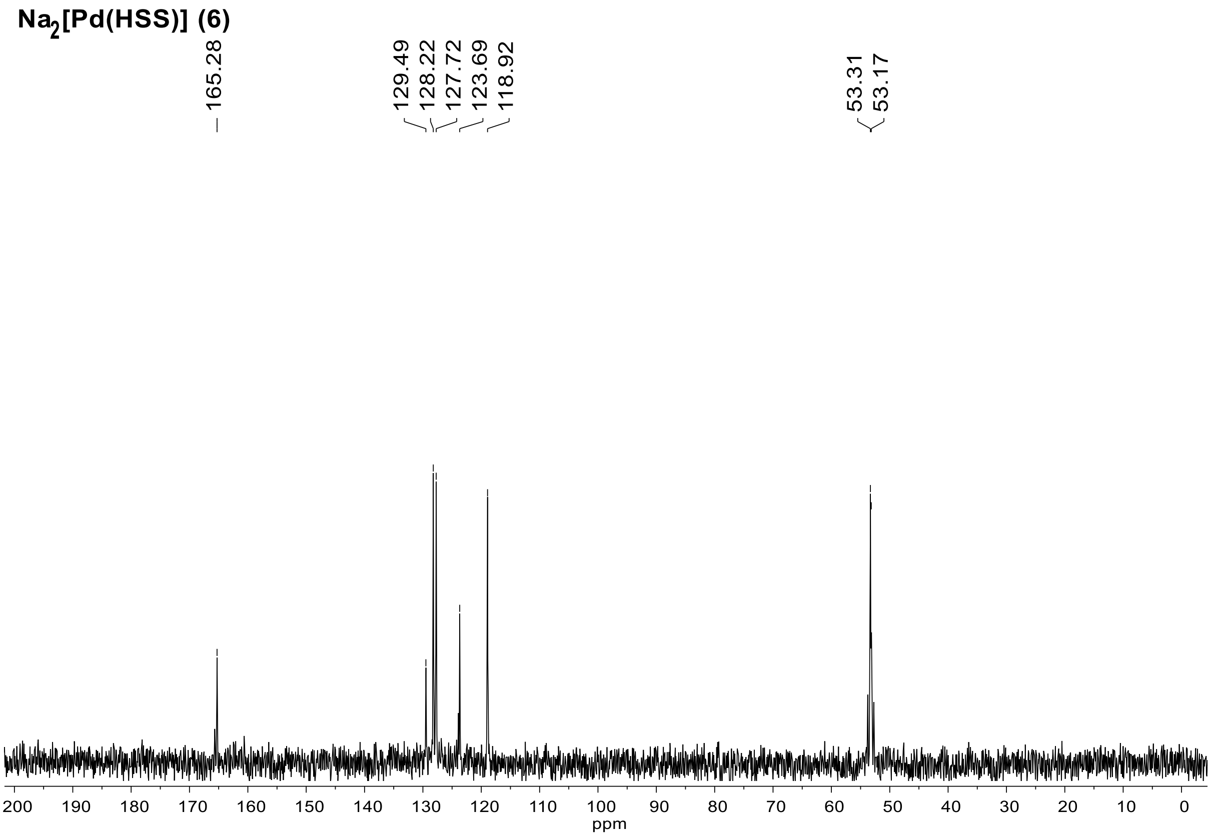

Figure S45. $^{13}C\{^{1}H\}$ NMR spectrum of **Na_2[Pd(HSS)] (6)** (90 MHz, 298K, D_2O)

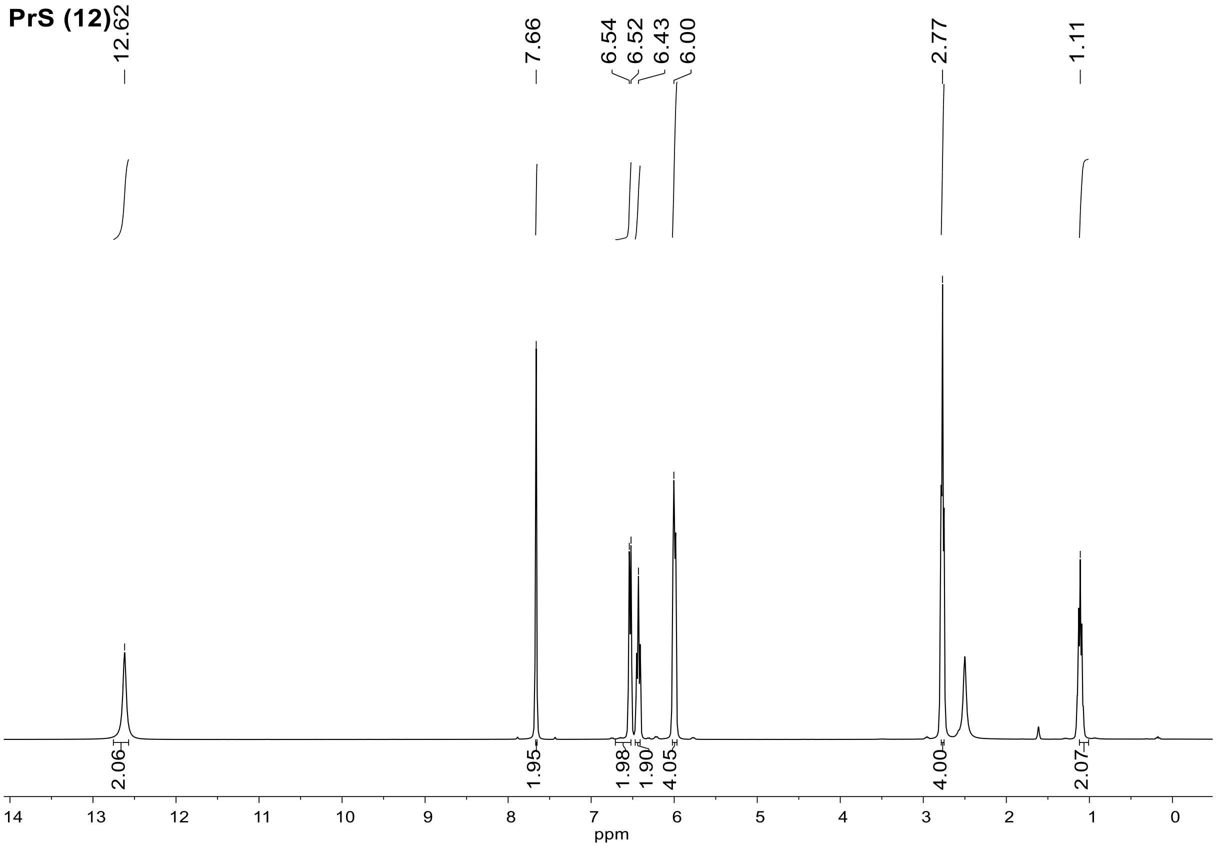

Figure S46. ^{1}H NMR spectrum of **PrS (12)** (360 MHz, 298K, d^{6}-dmso)

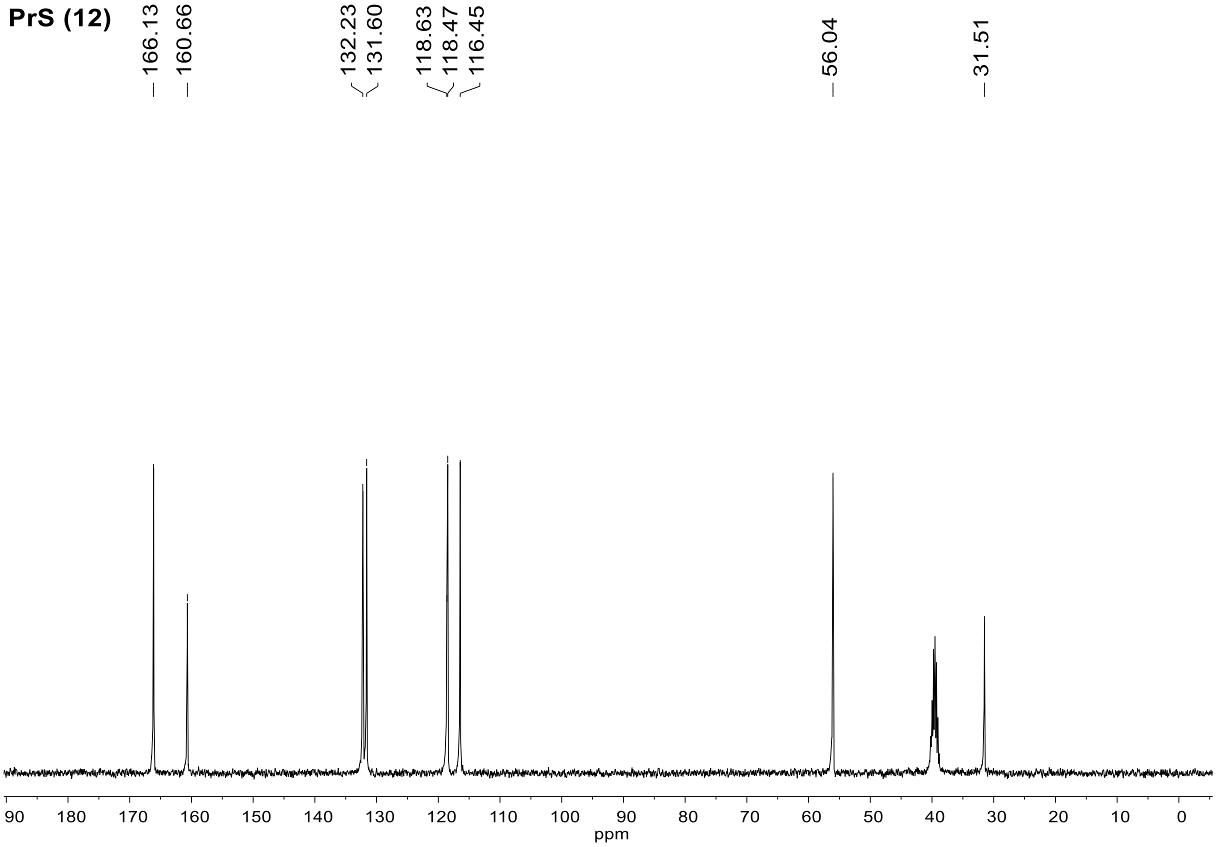

Figure S47. ^{13}C{^{1}H} NMR spectrum of **PrS (12)** (90 MHz, 298K, d^{6}-dmso)

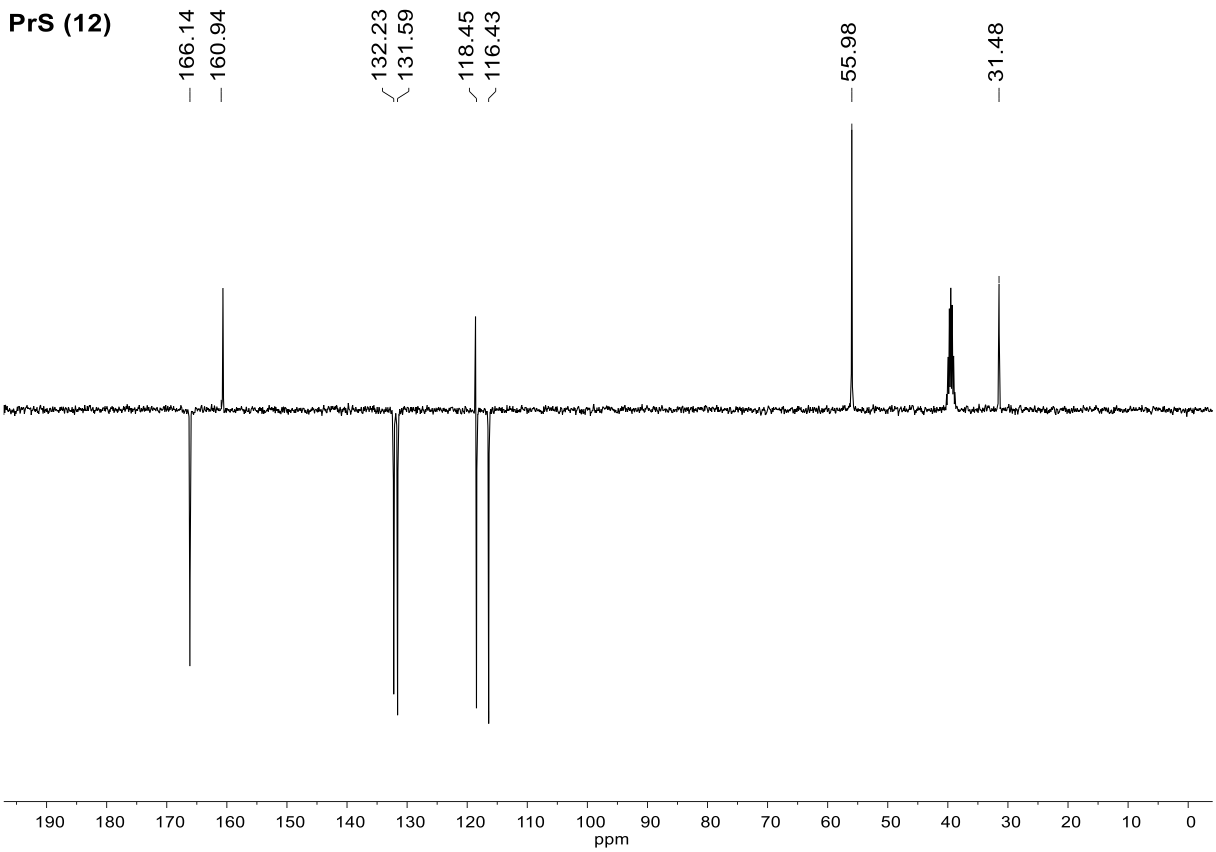

Figure S48. $^{13}C\{^{1}H\}$ NMR spectrum of **PrS (12)** (90 MHz, 298K, d^6-dmso)

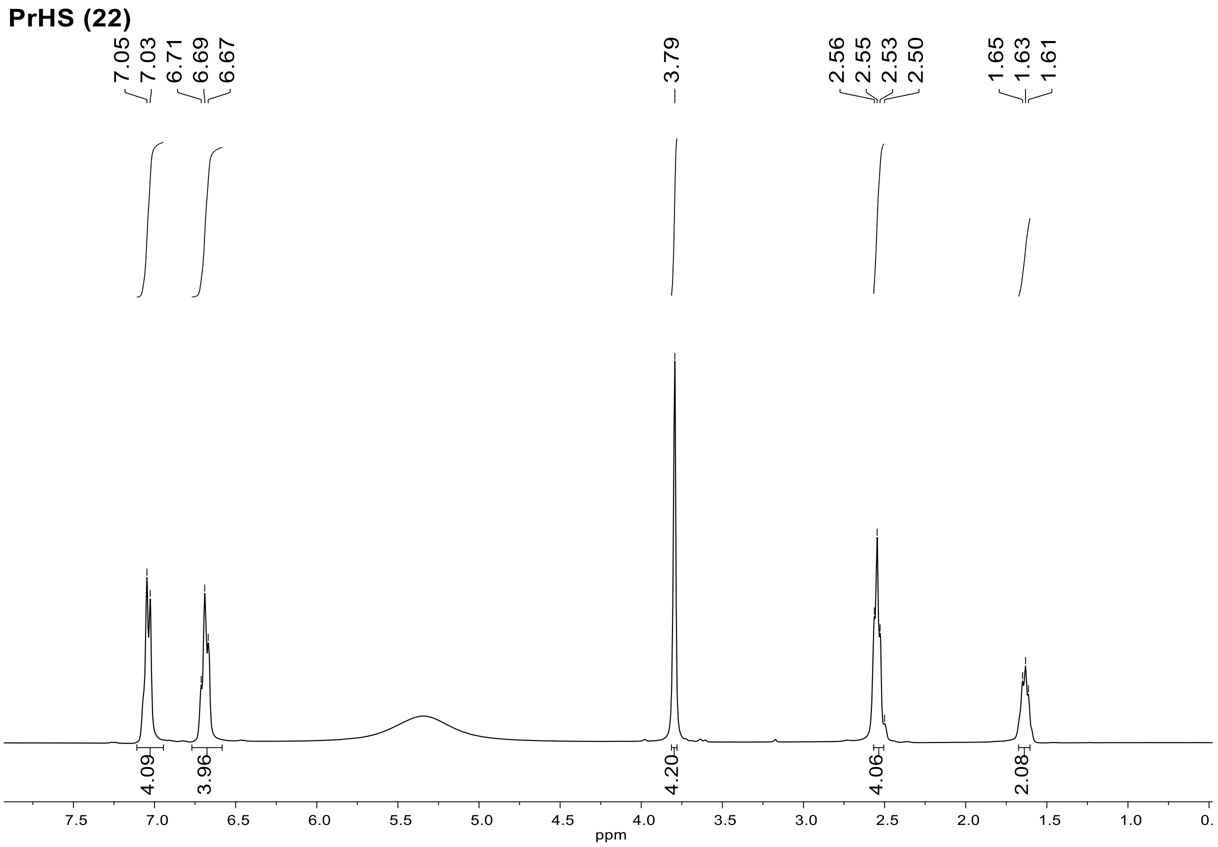

Figure S49. ^{1}H NMR spectrum of **PrHS (22)** (360 MHz, 298K, d^6-dmso)

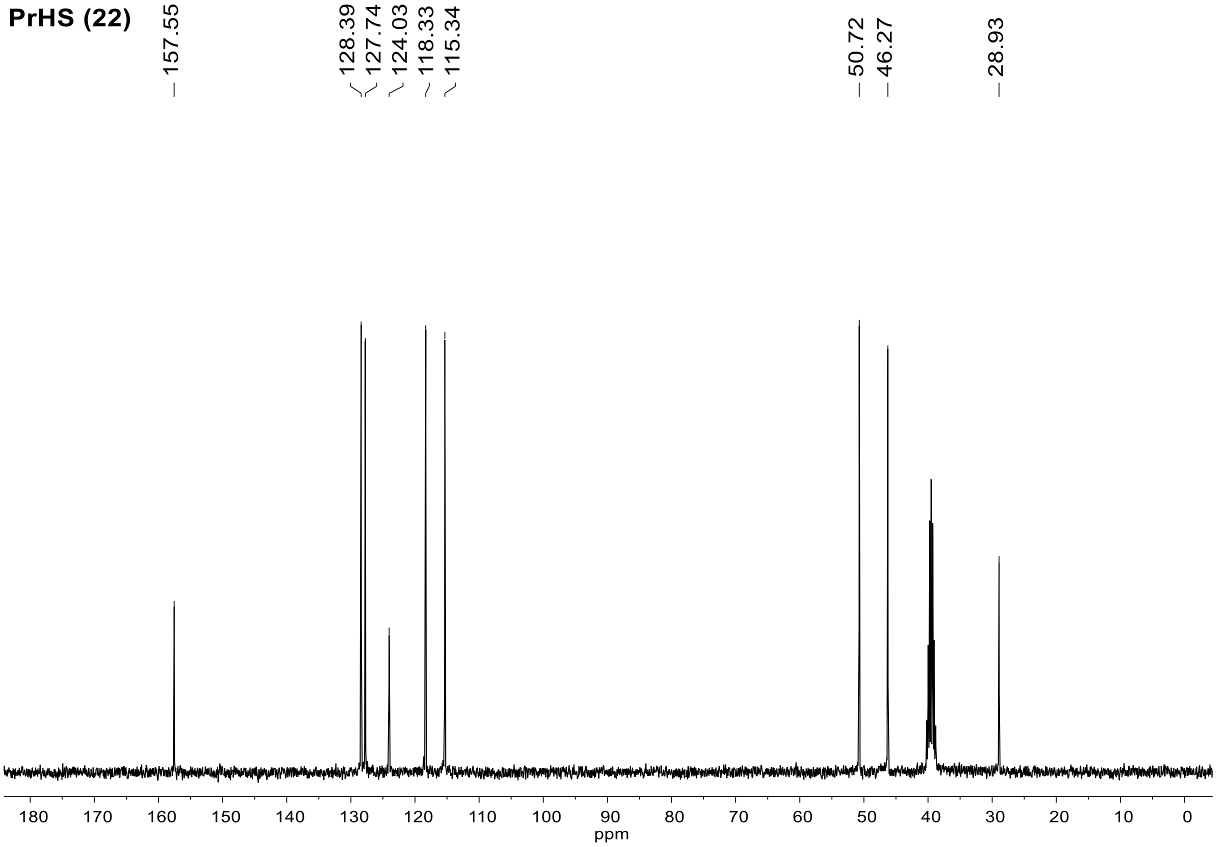

Figure S50. $^{13}C\{^{1}H\}$ NMR spectrum of **PrHS (22)** (90 MHz, 298K, d^6-dmso)

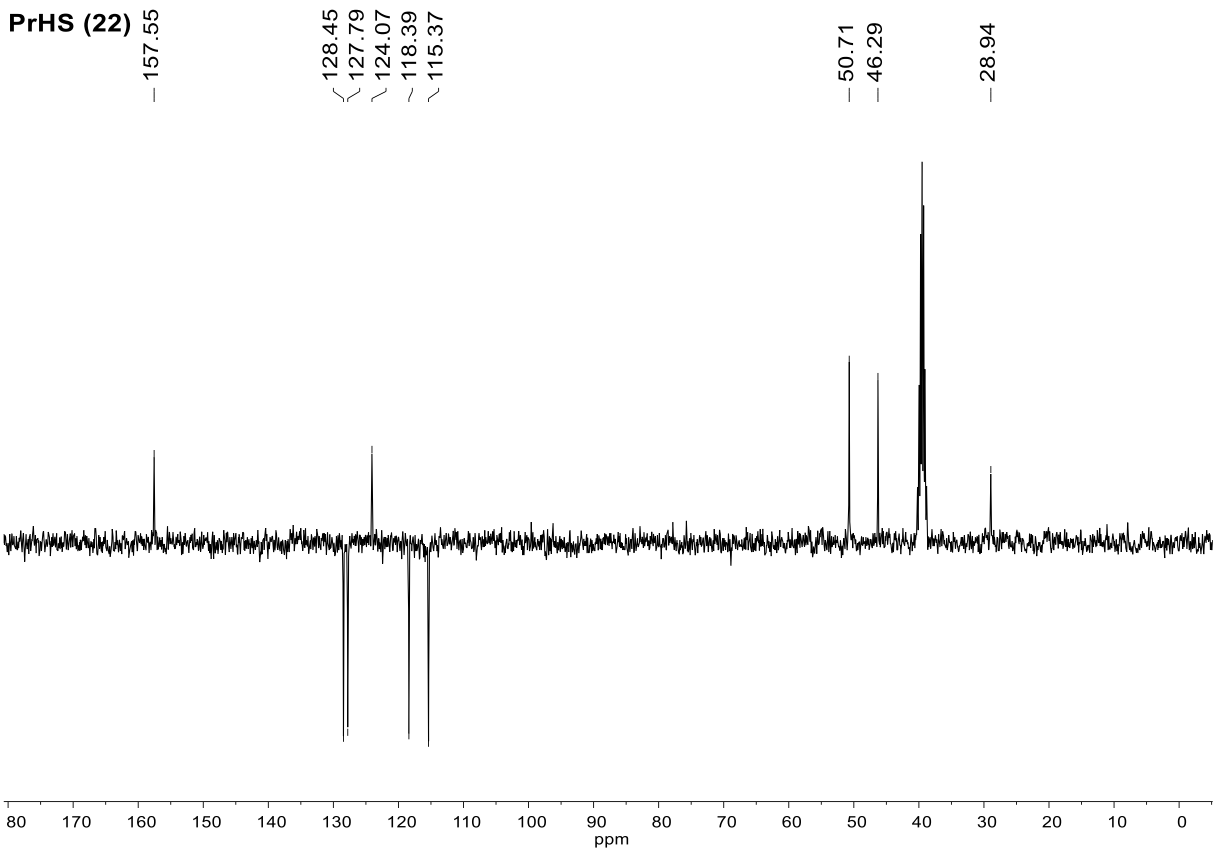

Figure S51. $^{13}C\{^{1}H\}$ NMR spectrum of **PrHS (22)** (90 MHz, 298K, d^6-dmso)

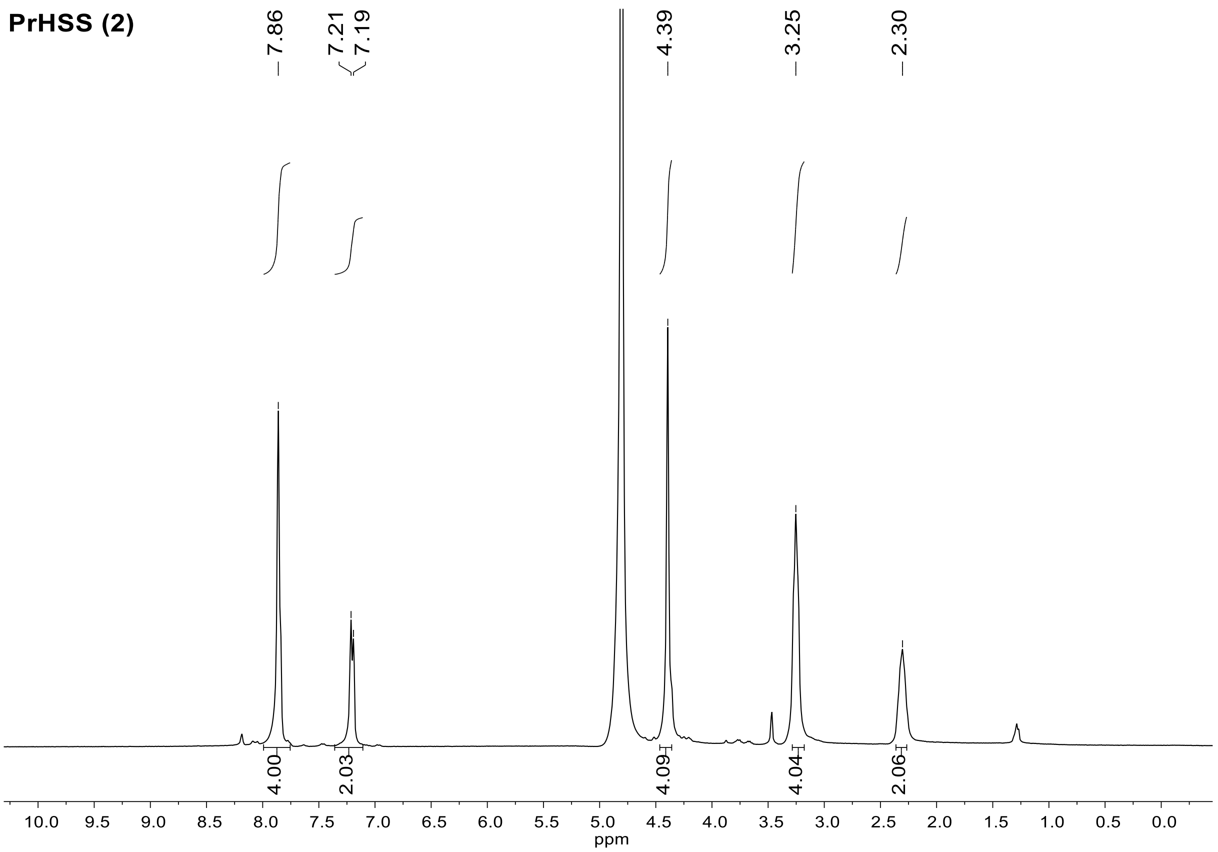

Figure S52. ^{1}H NMR spectrum of **PrHSS (2)** (360 MHz, 298K, D_2O)

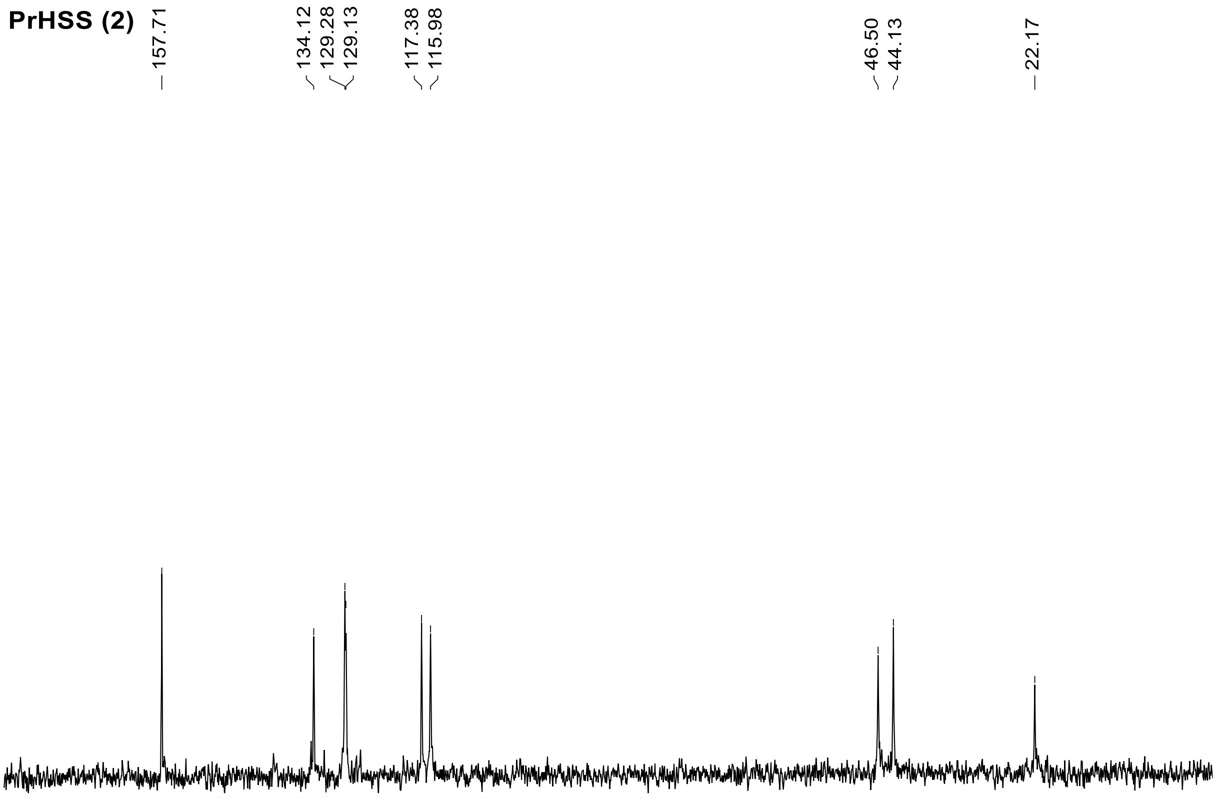

Figure S53. $^{13}C\{^{1}H\}$ NMR spectrum of **PrHSS (2)** (90 MHz, 298K, D_2O)

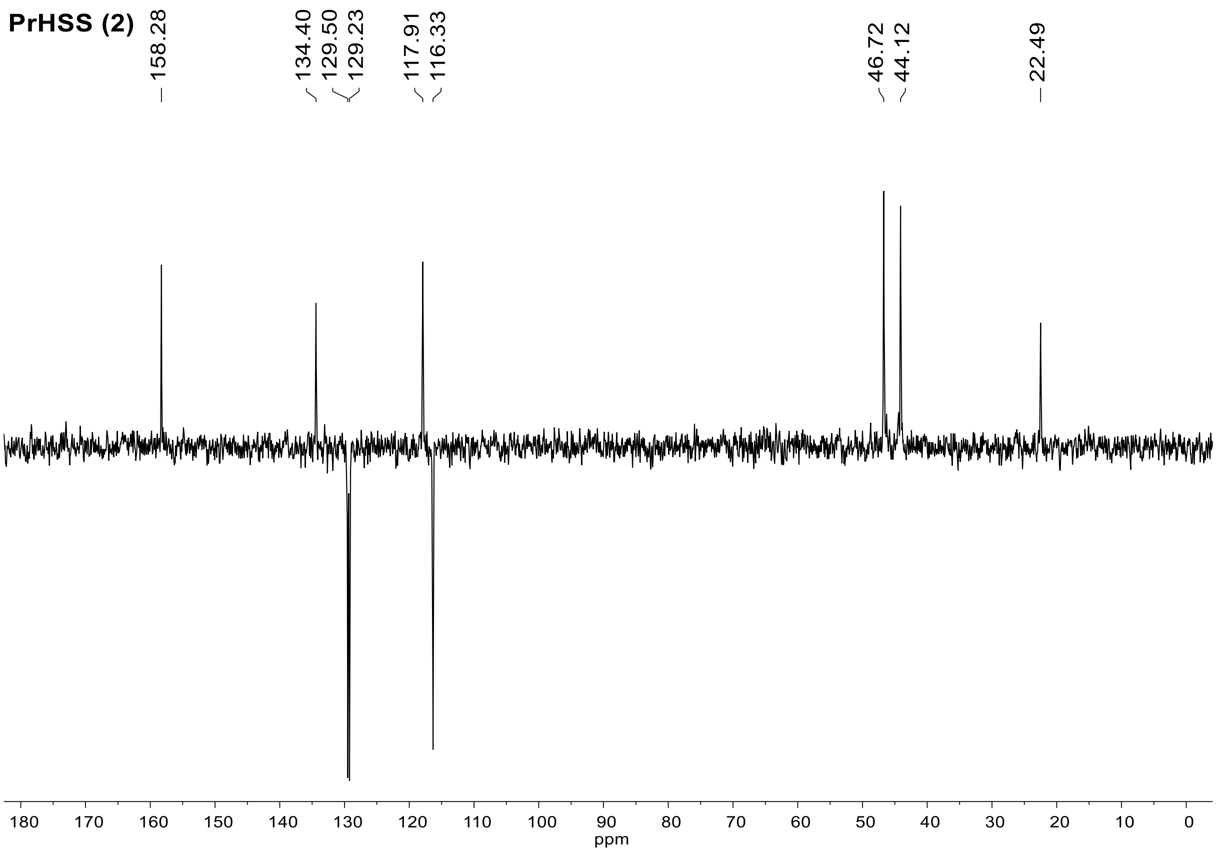

Figure S54. $^{13}C\{^{1}H\}$ NMR spectrum of **PrHSS (2)** (90 MHz, 298K, D_2O)

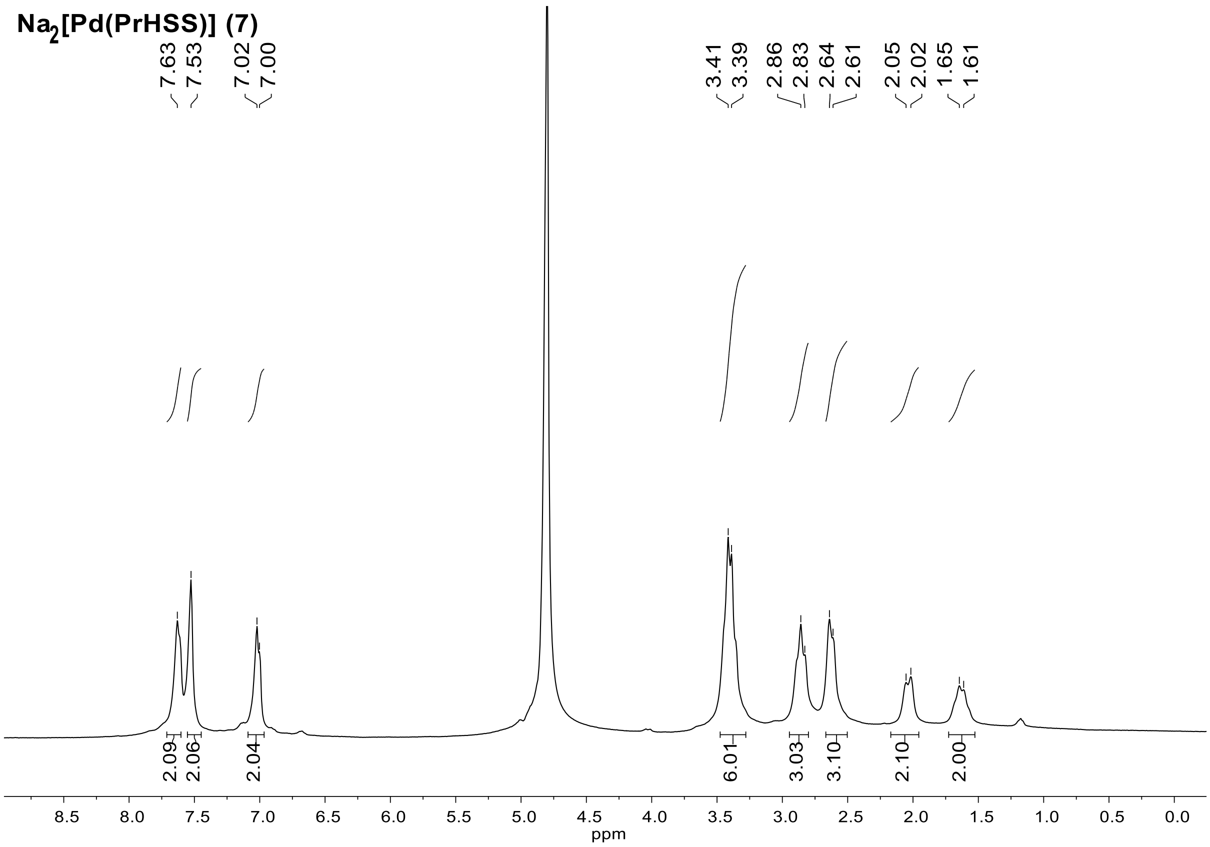

Figure S55.A. ^{1}H NMR spectrum of **Na_2[Pd(PrHSS)] (7)** (360 MHz, 298K, D_2O)

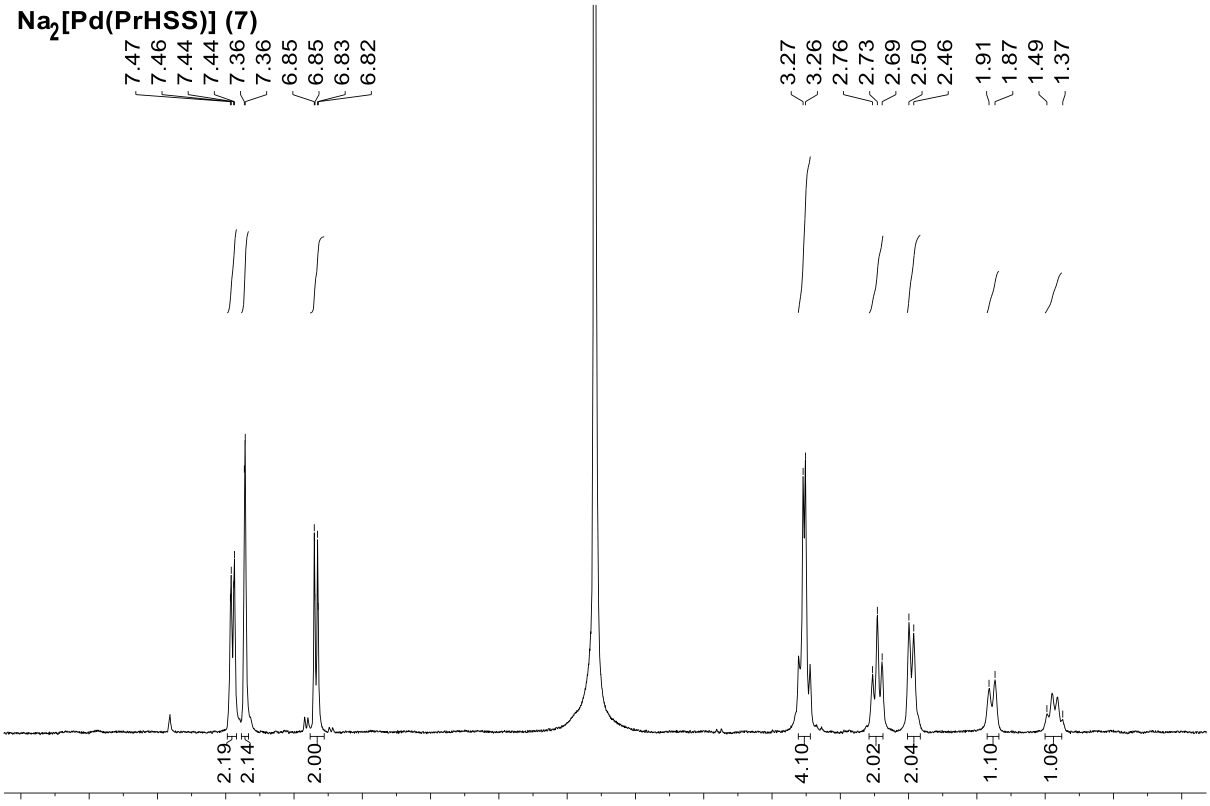

Figure S55.B. ^{1}H NMR spectrum of **Na_2[Pd(PrHSS)] (7)** (360 MHz, 273 K, D_2O+CD_3OD)

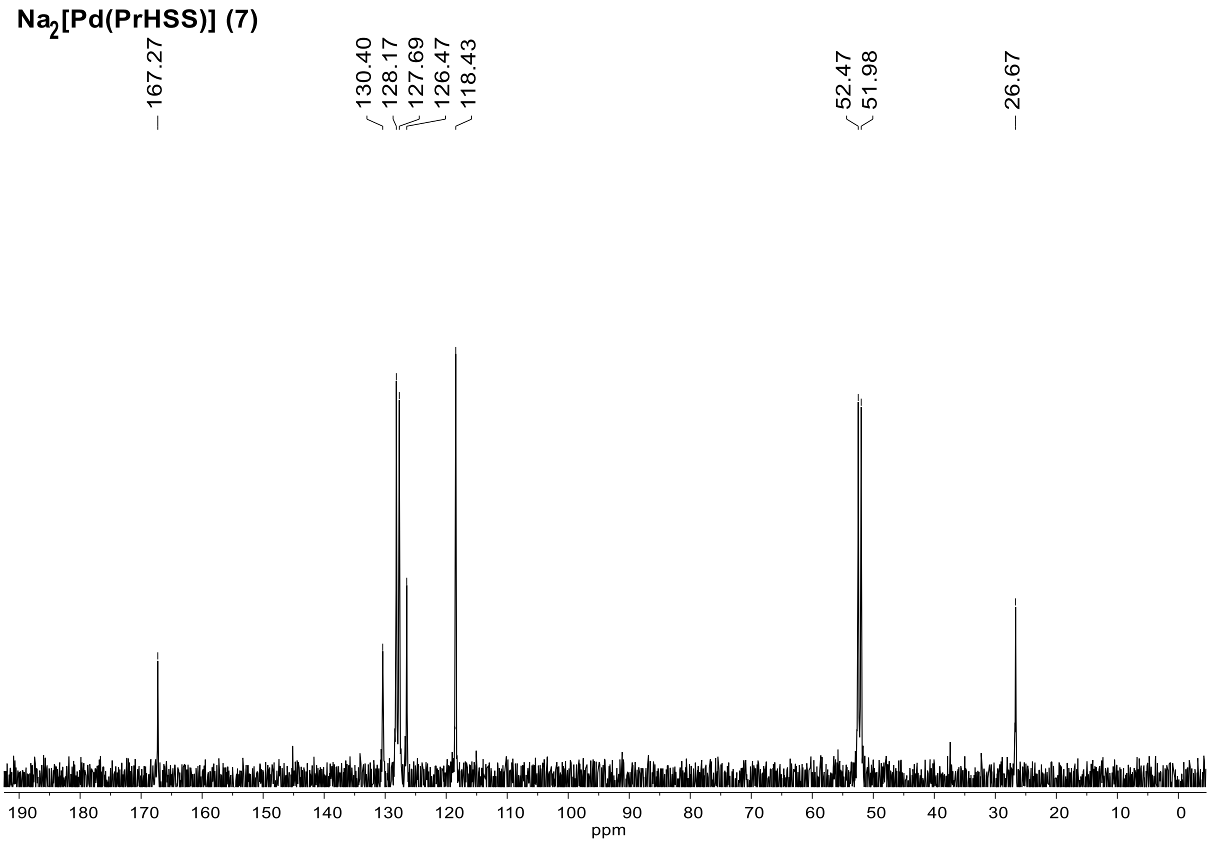

Figure S56. $^{13}C\{^{1}H\}$ NMR spectrum of **Na_2[Pd(PrHSS)] (7)** (90 MHz, 298K, D_2O)

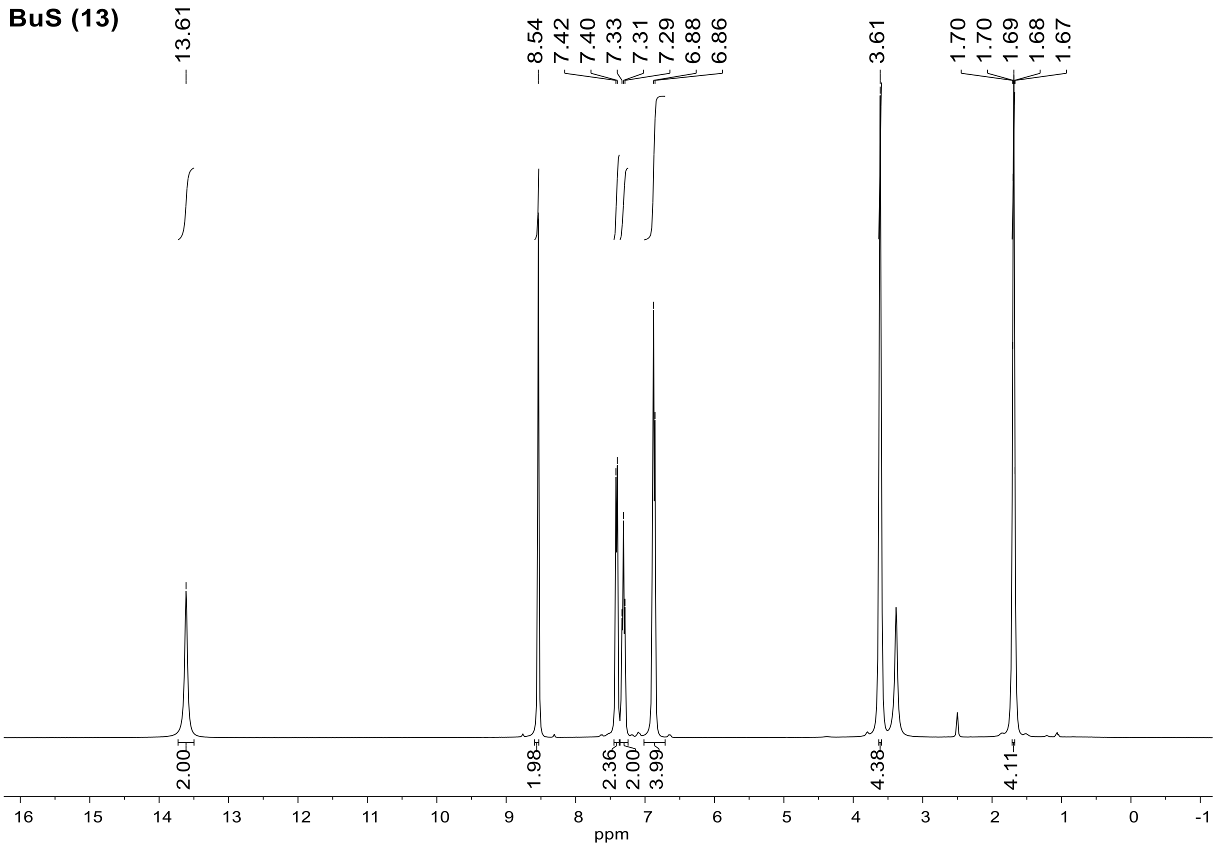

Figure S57. ^{1}H NMR spectrum of **BuS (13)** (360 MHz, 298K, d^6-dmso)

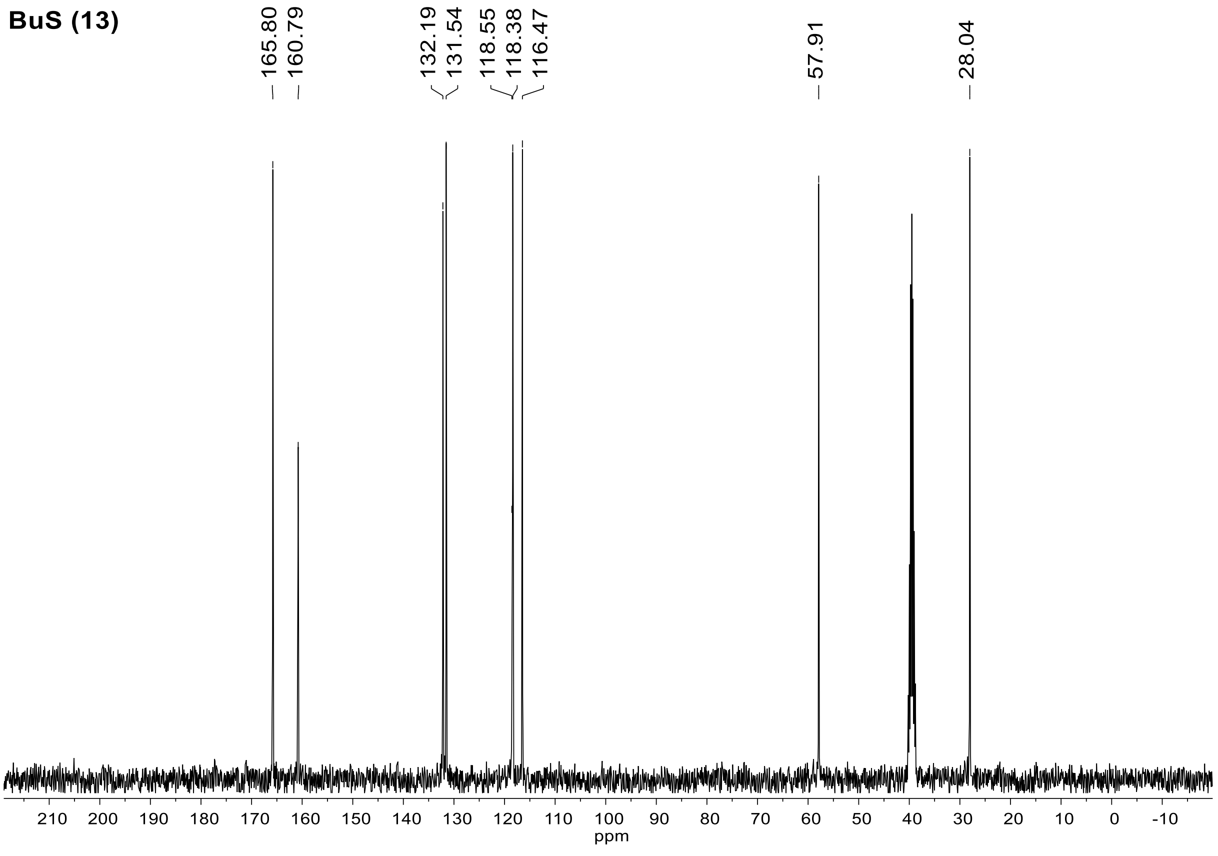

Figure S58. $^{13}C\{^{1}H\}$ NMR spectrum of **BuS (13)** (90 MHz, 298K, d^6-dmso)

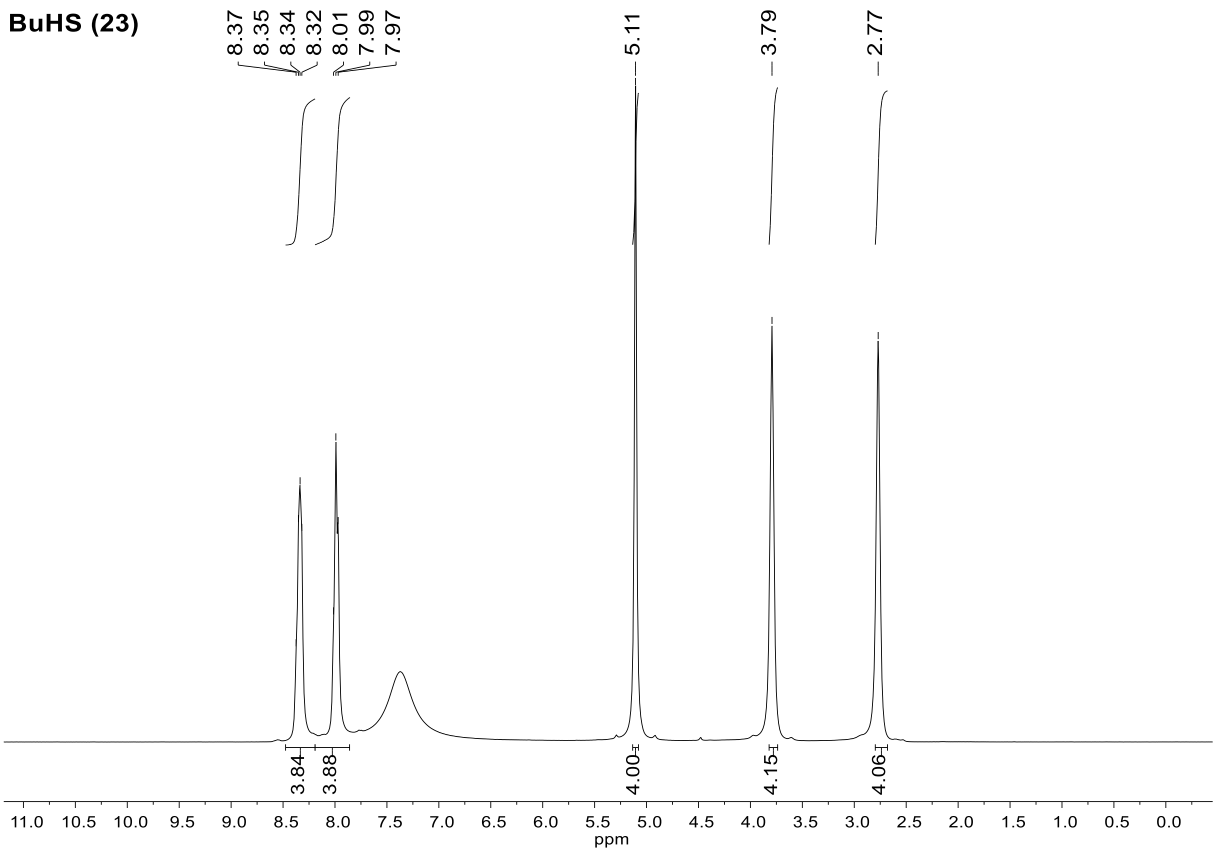

Figure S59. ^{1}H NMR spectrum of **BuHS (23)** (360 MHz, 298K, d^{6}-dmso)

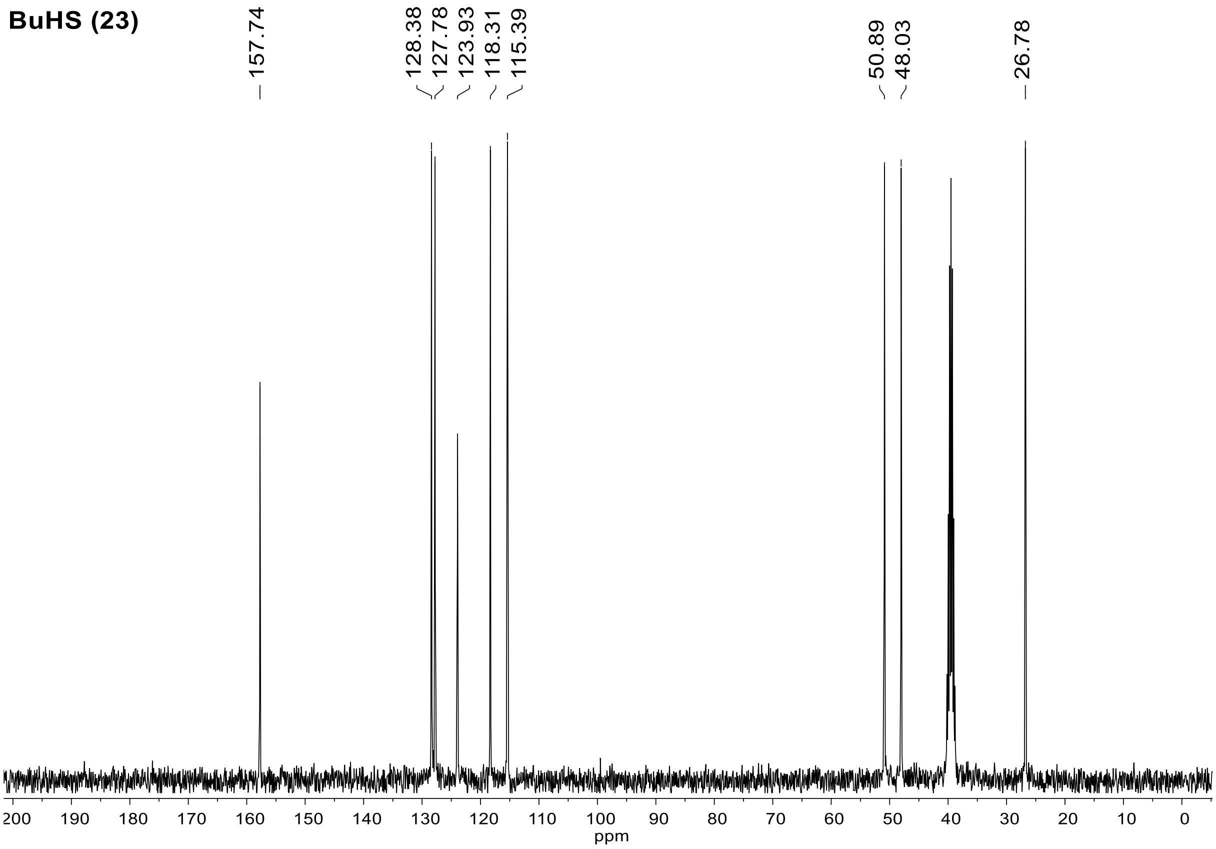

Figure S60. $^{13}C\{^{1}H\}$ NMR spectrum of **BuHS (23)** (90 MHz, 298K, d^{6}-dmso)

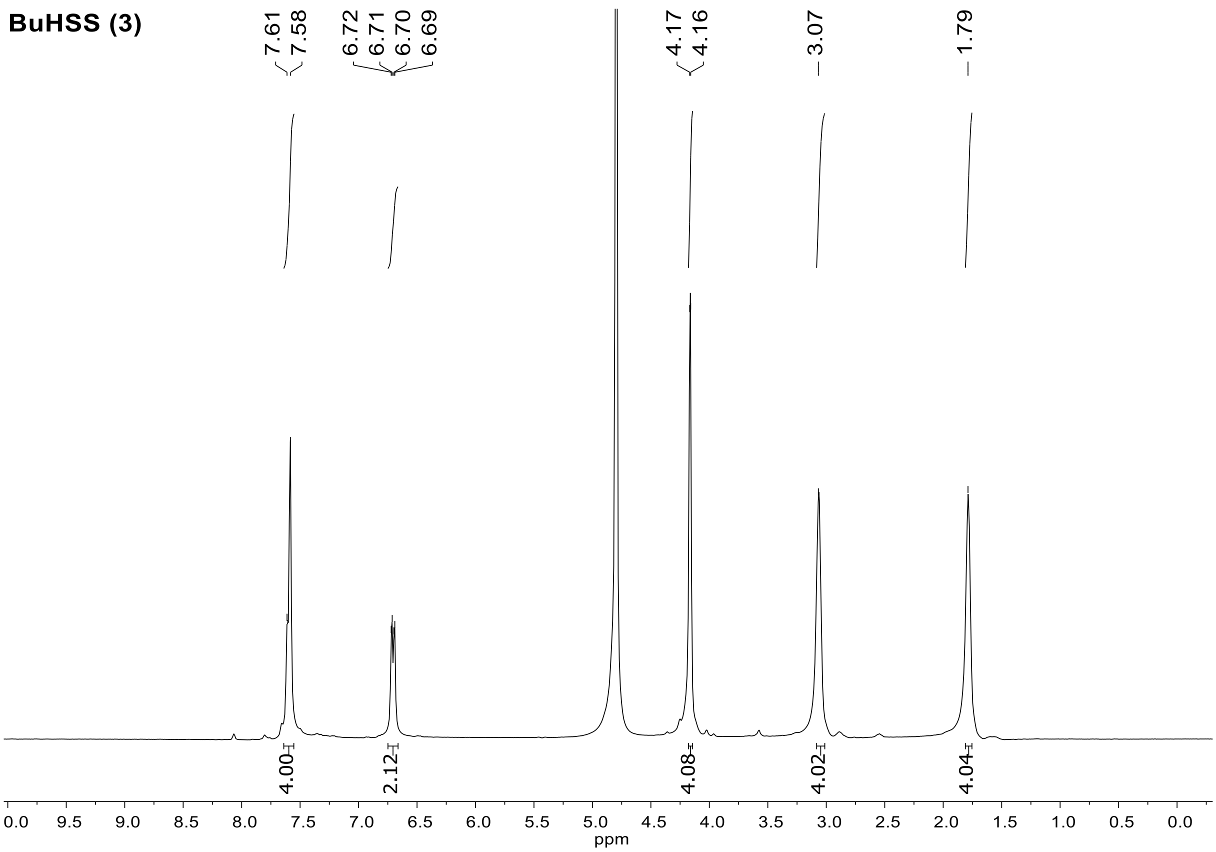

Figure S61. ^{1}H NMR spectrum of **BuHSS (3)** (360 MHz, 298K, D_2O)

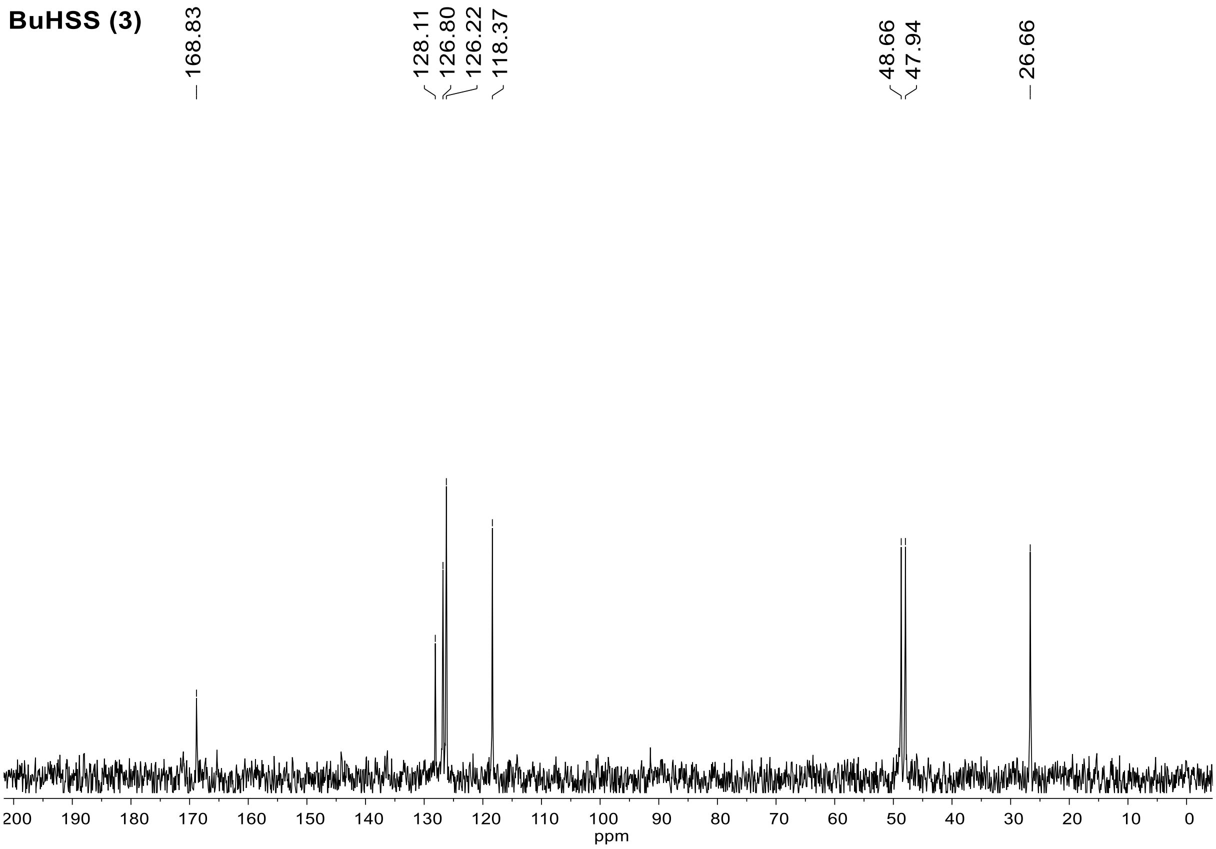

Figure S62. $^{13}C\{^{1}H\}$ NMR spectrum of **BuHSS (3)** (90 MHz, 298K, D_2O)

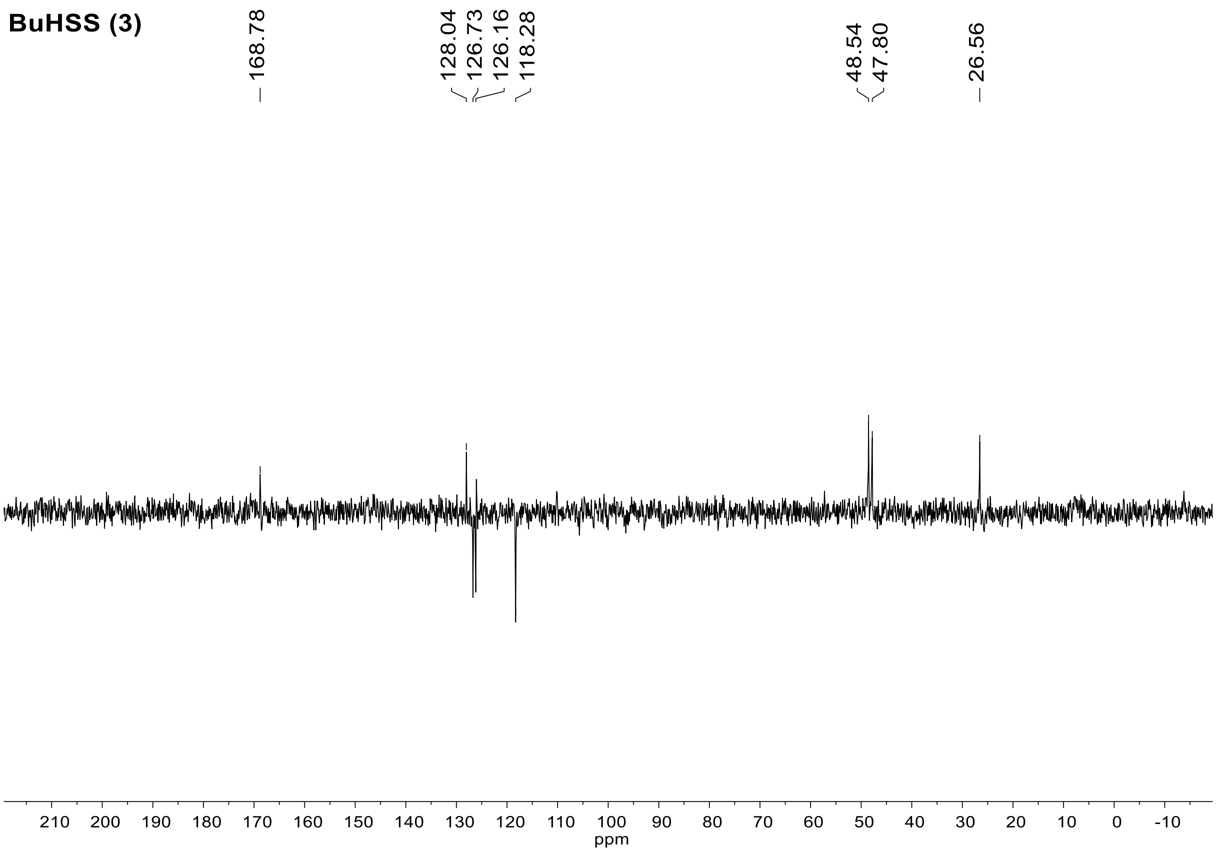

Figure S63. $^{13}C\{^{1}H\}$ NMR spectrum of **BuHSS (3)** (90 MHz, 298K, D_2O)

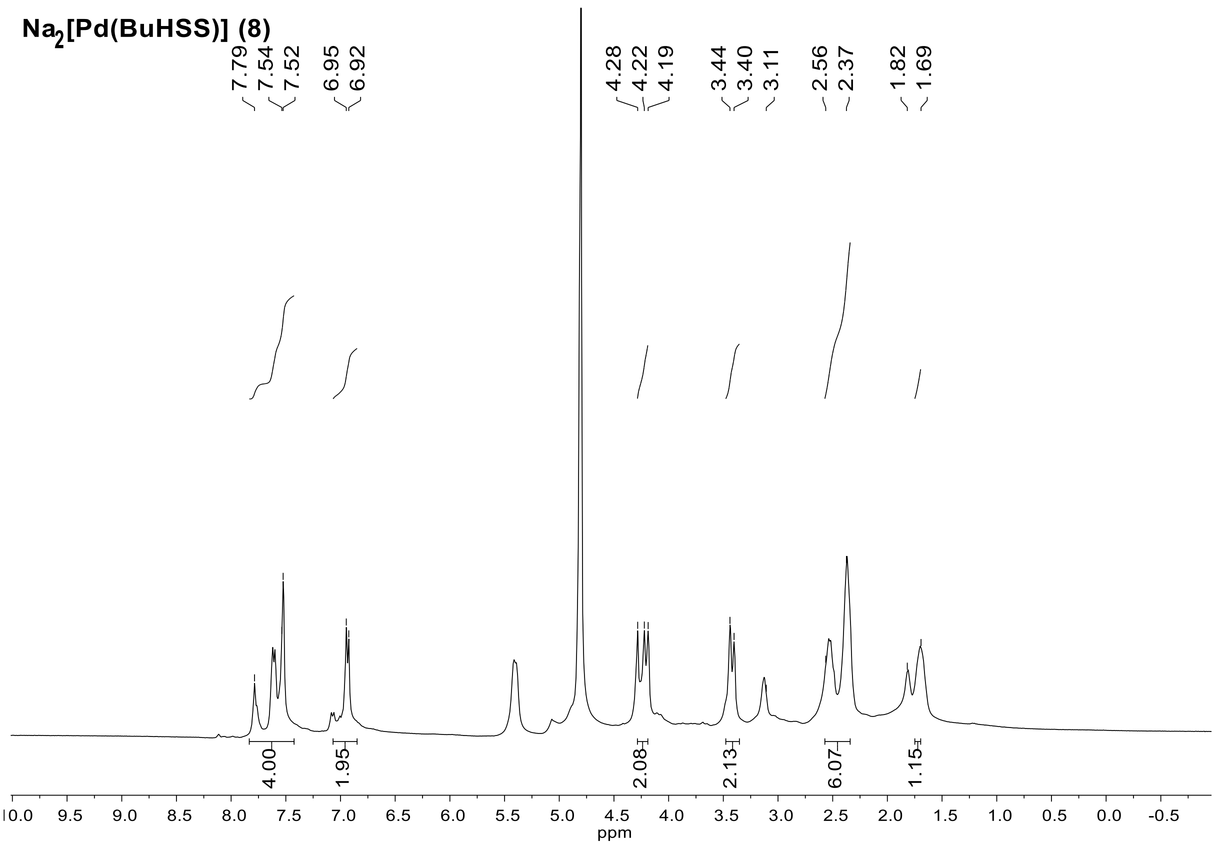

Figure S64.A. ^{1}H NMR spectrum of **Na_2[Pd(BuHSS)] (8)** (360 MHz, 298K, D_2O)

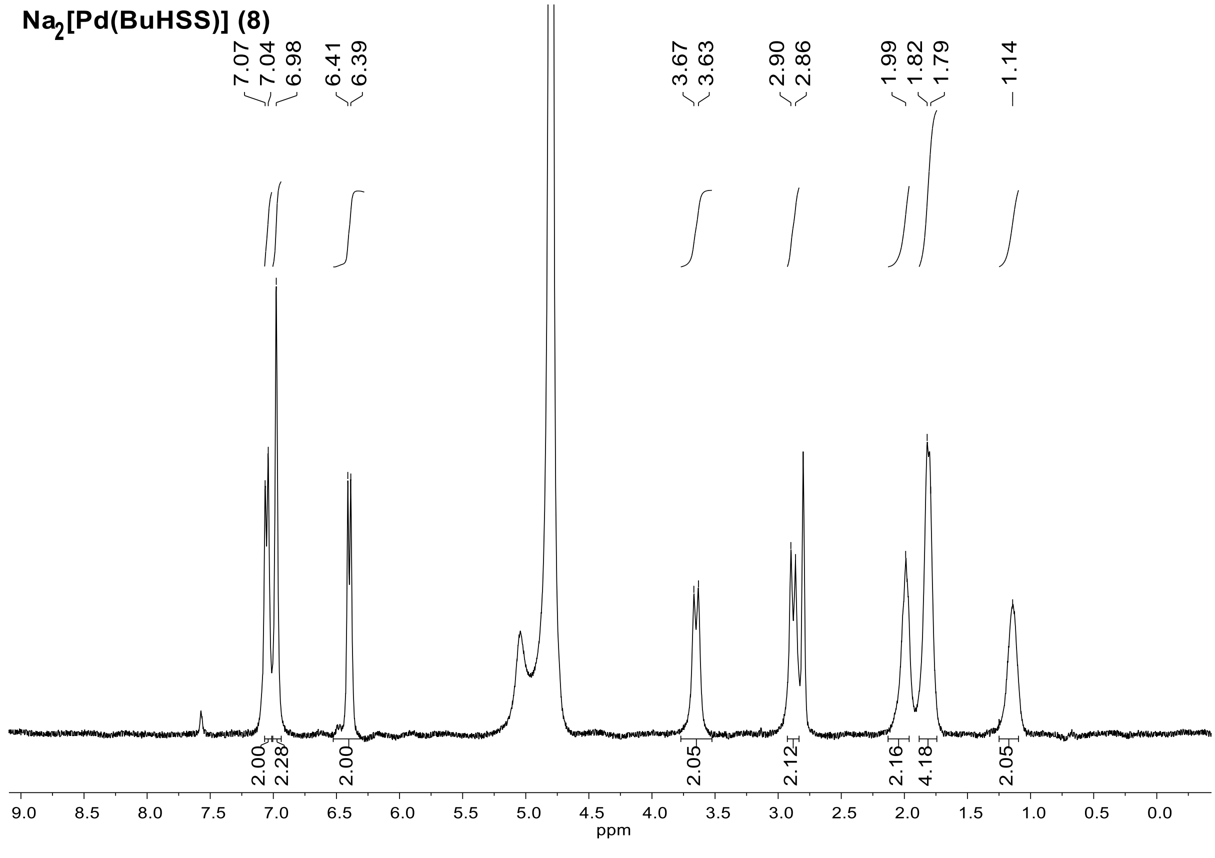

Figure S64.B. ^{1}H NMR spectrum of **Na_2[Pd(BuHSS)] (8)** (360 MHz, 268K, D_2O+CD_3OD)

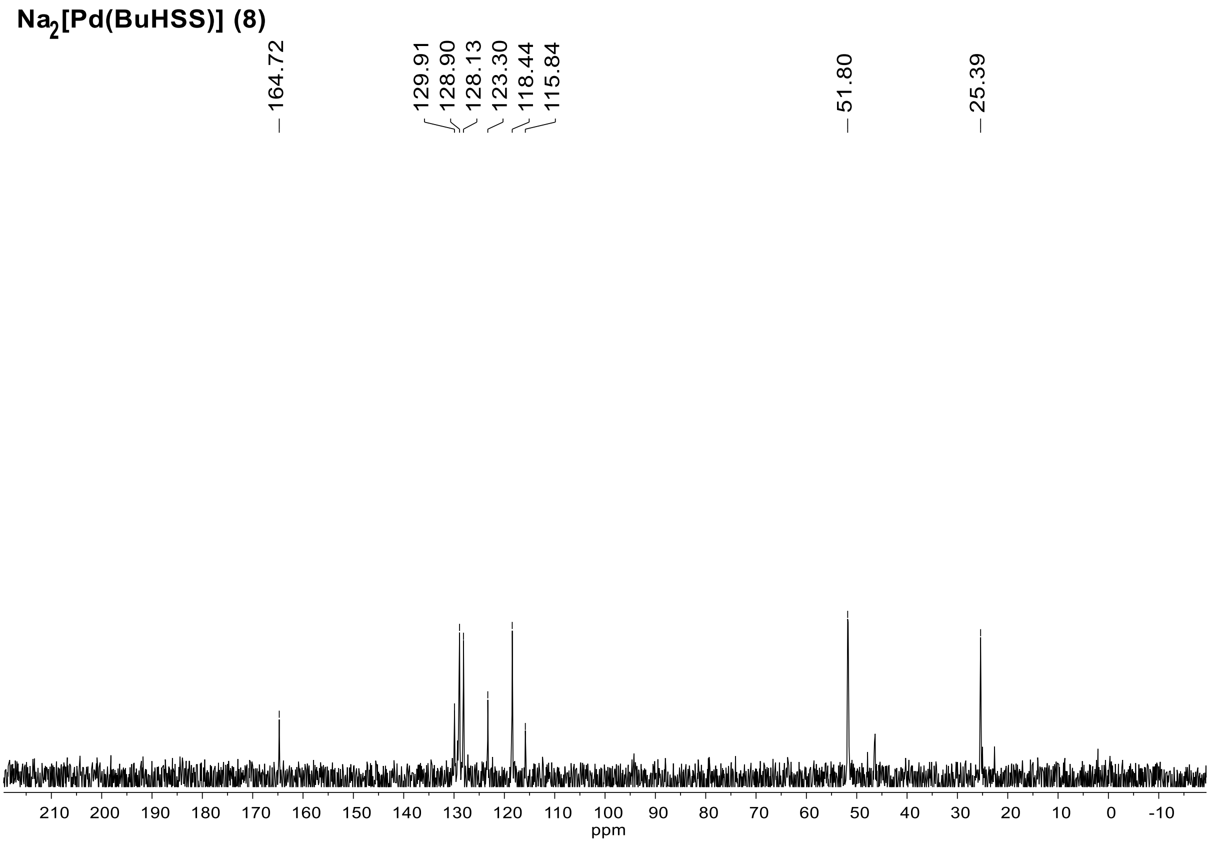

Figure S65. $^{13}C\{^{1}H\}$ NMR spectrum of **Na_2[Pd(BuHSS)] (8)** (90 MHz, 298K, D_2O)

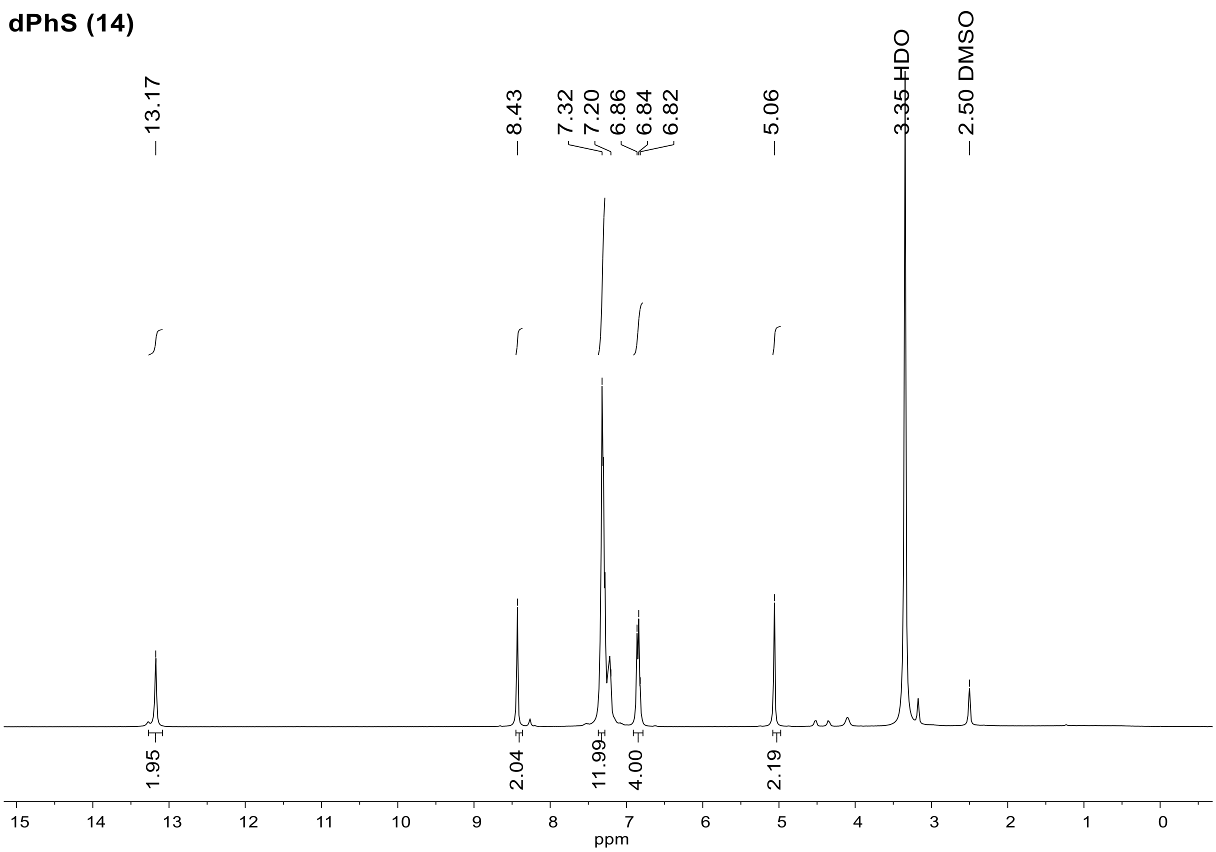

Figure S66. ^{1}H NMR spectrum of **dPhS (14)** (360 MHz, 298K, d^6-dmso)

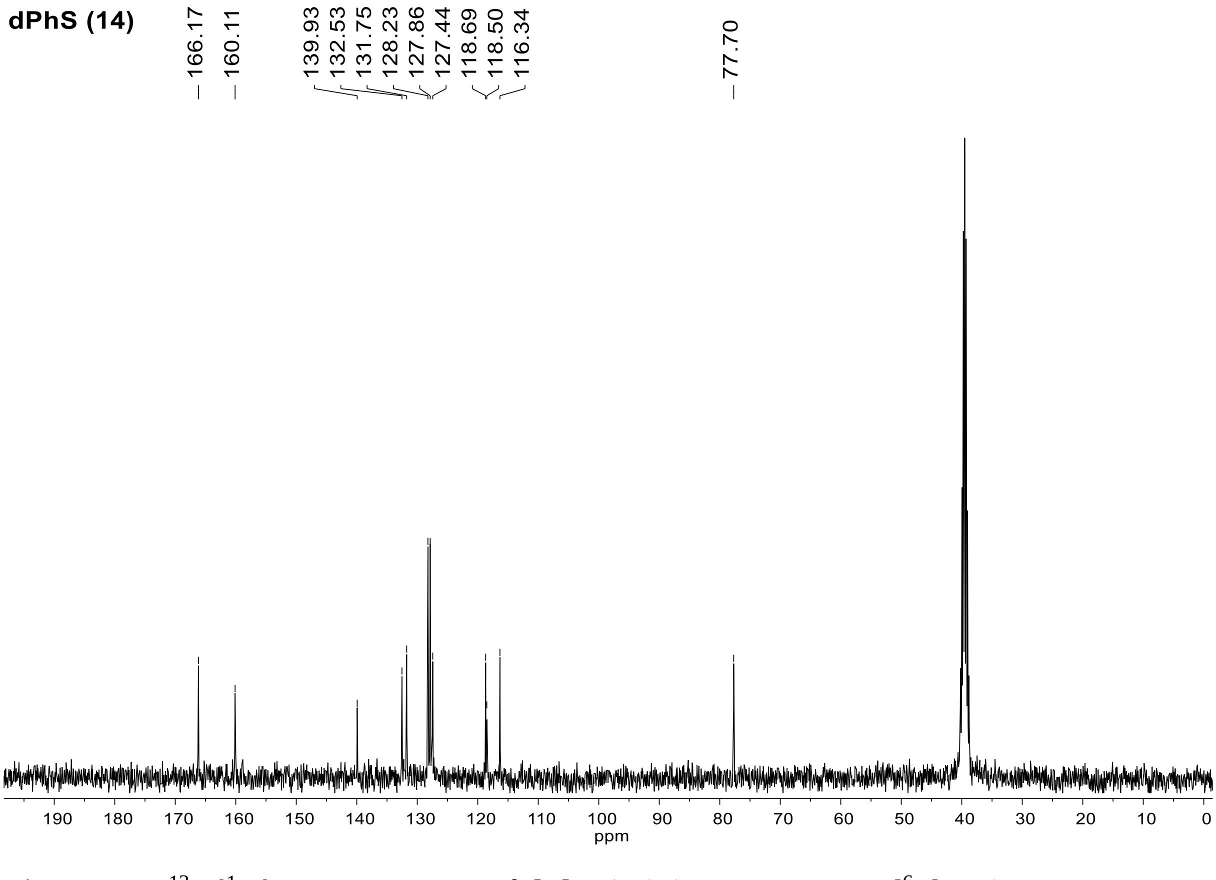

Figure S67. $^{13}C\{^{1}H\}$ NMR spectrum of **dPhS (14)** (90 MHz, 298K, d^6-dmso)

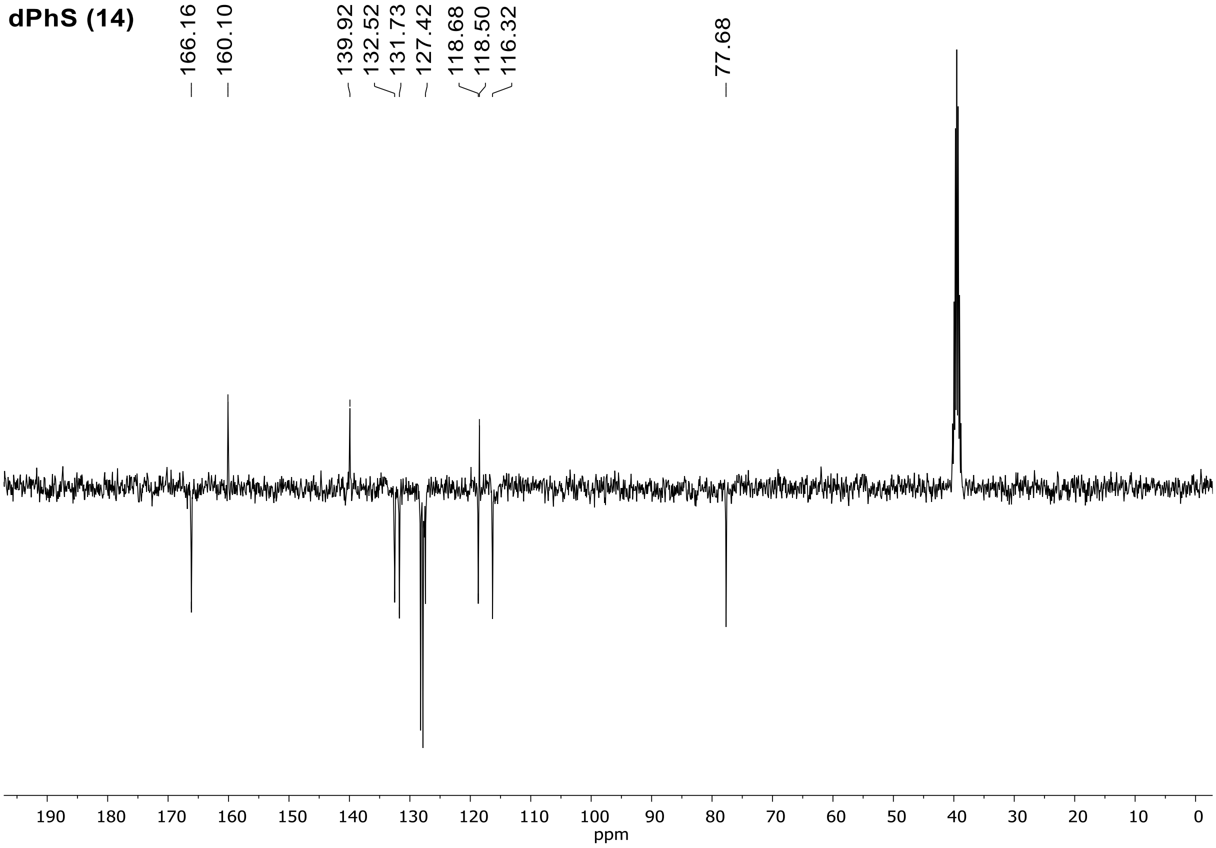

Figure S68. $^{13}C\{^{1}H\}$ NMR spectrum of **dPhS (14)** (90 MHz, 298K, d^6-dmso)

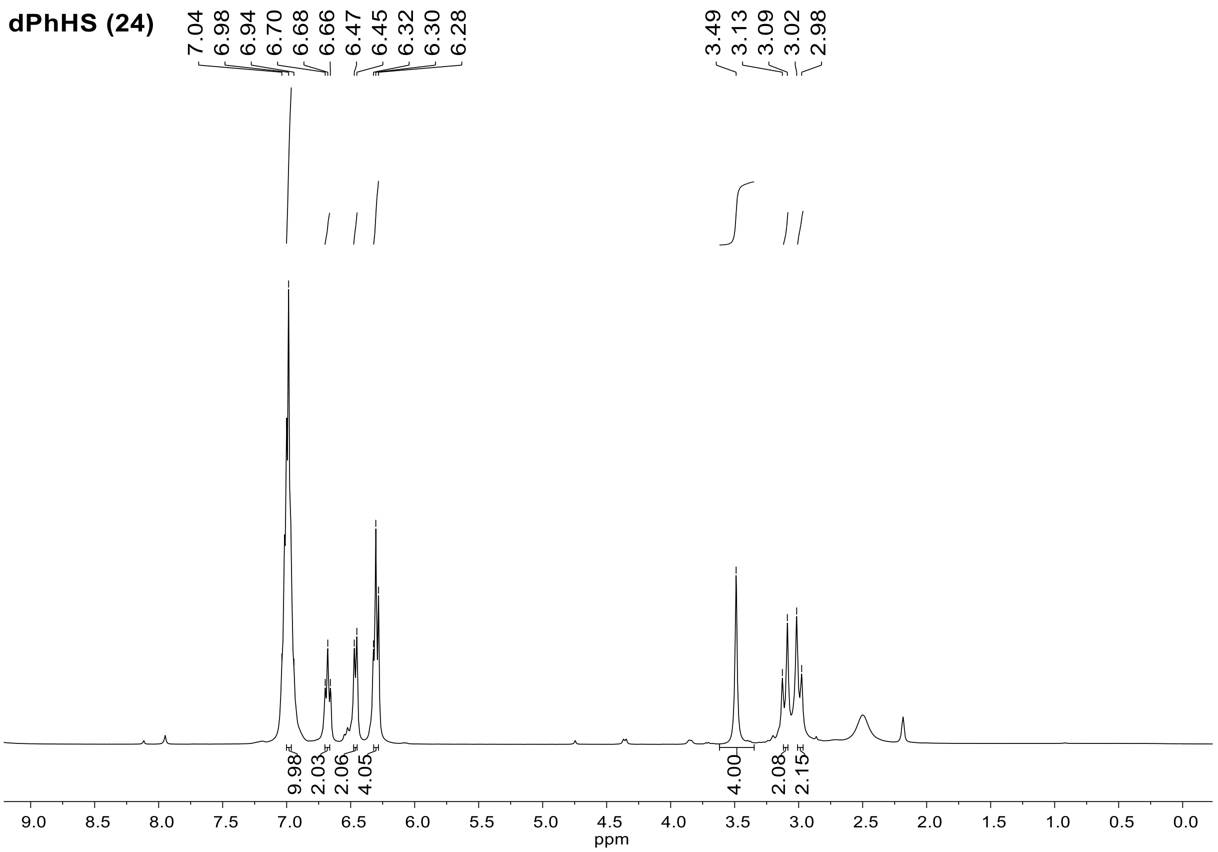

Figure S69. ^{1}H NMR spectrum of **dPhHS (24)** (360 MHz, 298K, d^{6}-dmso)

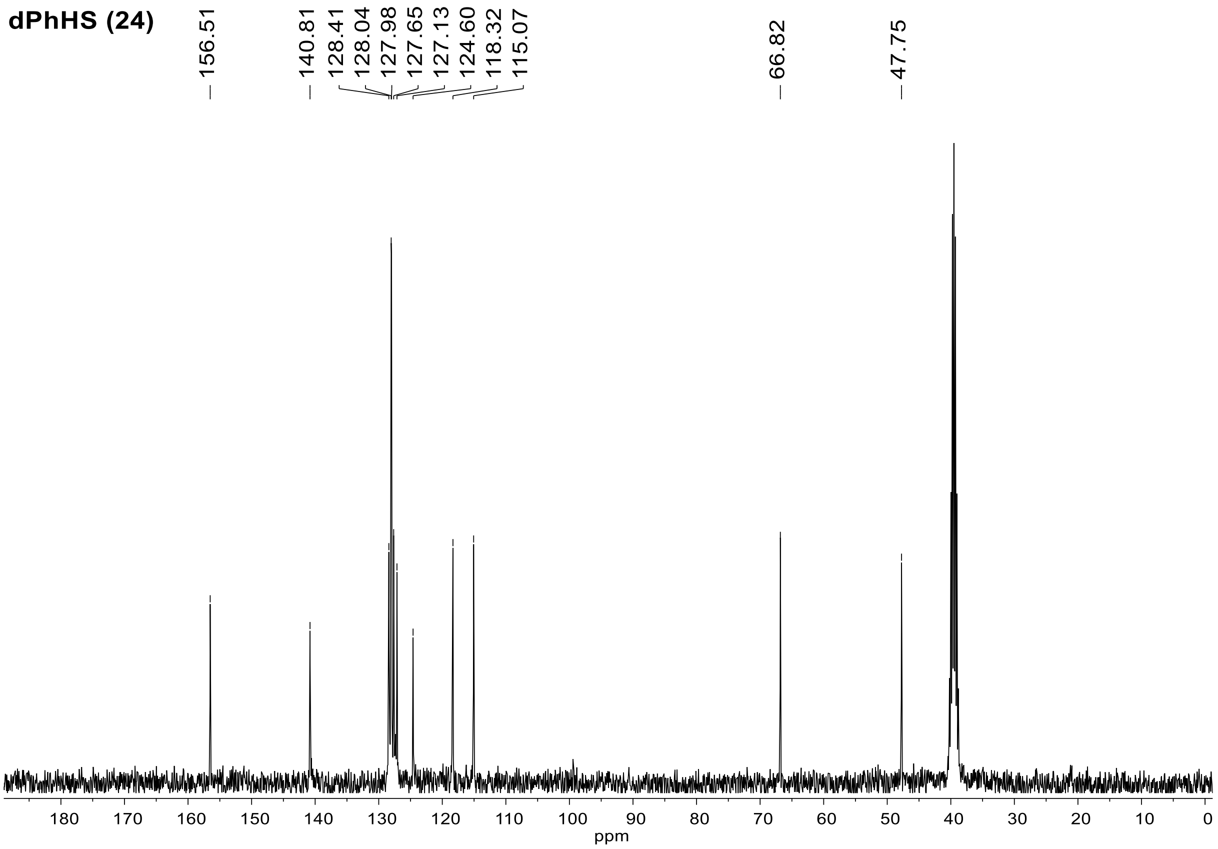

Figure S70. $^{13}C\{^{1}H\}$ NMR spectrum of **dPhHS (24)** (90 MHz, 298K, d^{6}-dmso)

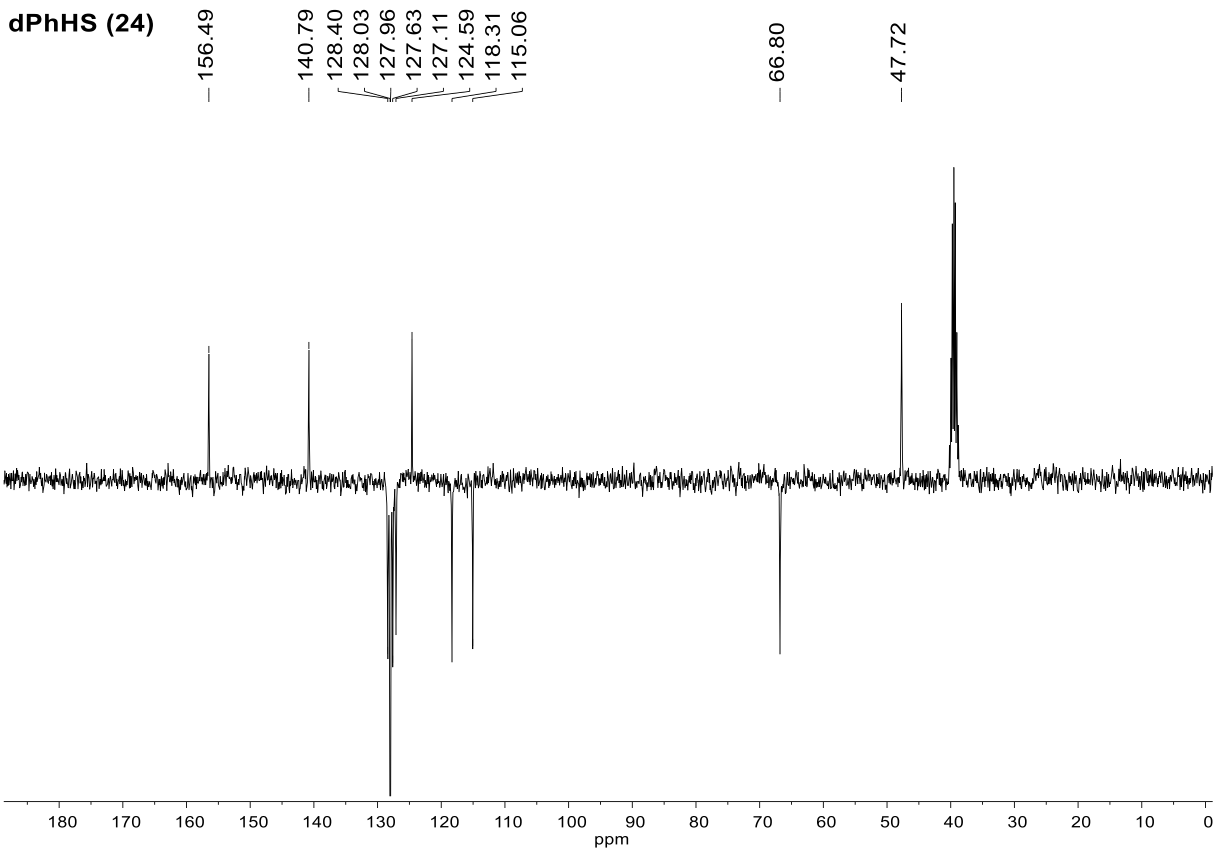

Figure S71. $^{13}C\{^{1}H\}$ NMR spectrum of **dPhHS (24)** (90 MHz, 298K, d^6-dmso)

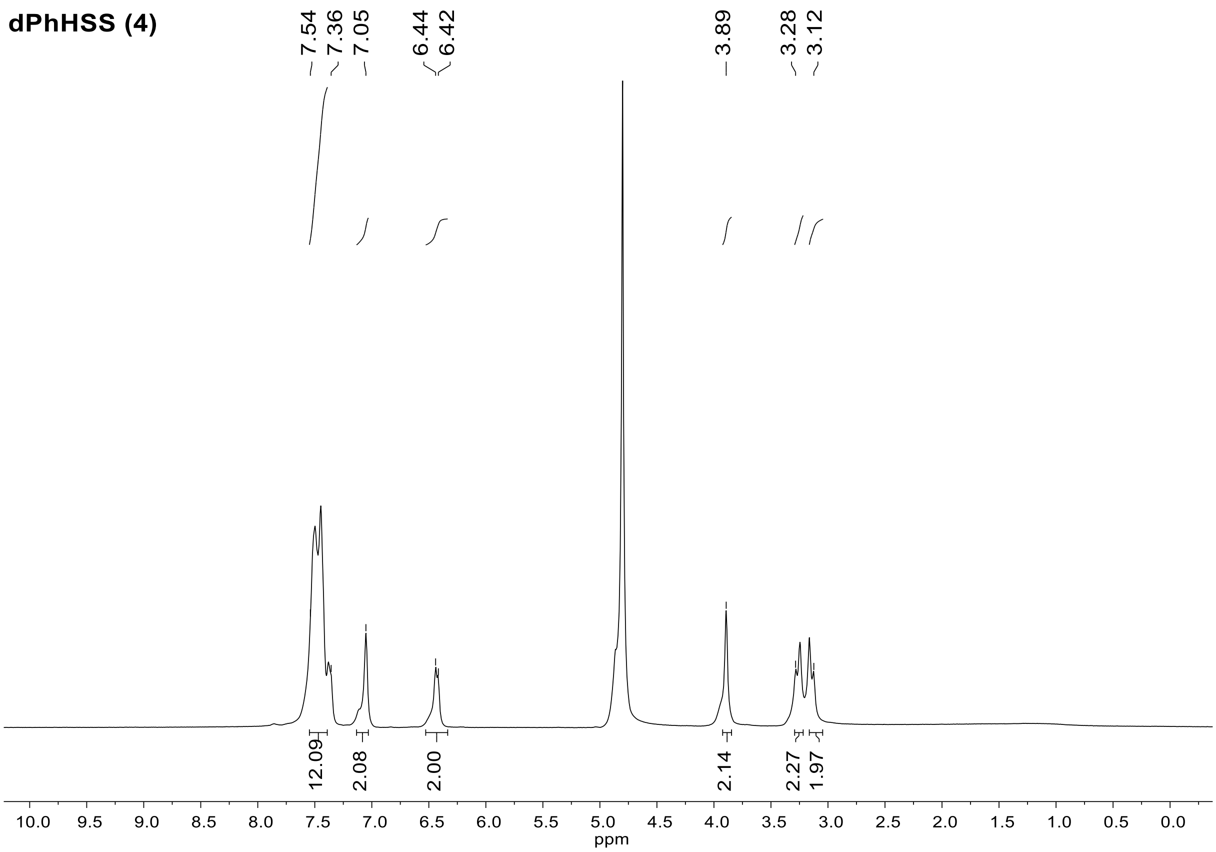

Figure S72. ^{1}H NMR spectrum of **dPhHSS (4)** (360 MHz, 298K, D_2O)

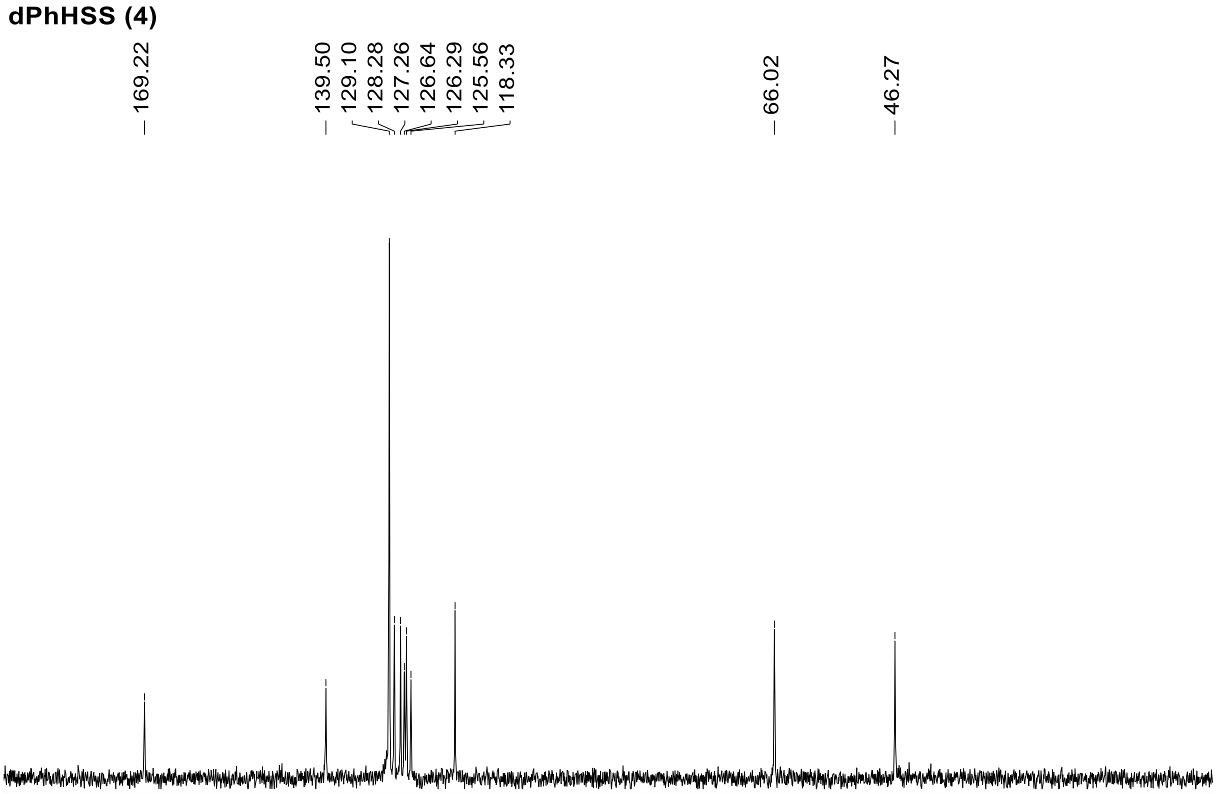

Figure S73. $^{13}C\{^{1}H\}$ NMR spectrum of **dPhHSS (4)** (90 MHz, 298K, D_2O)

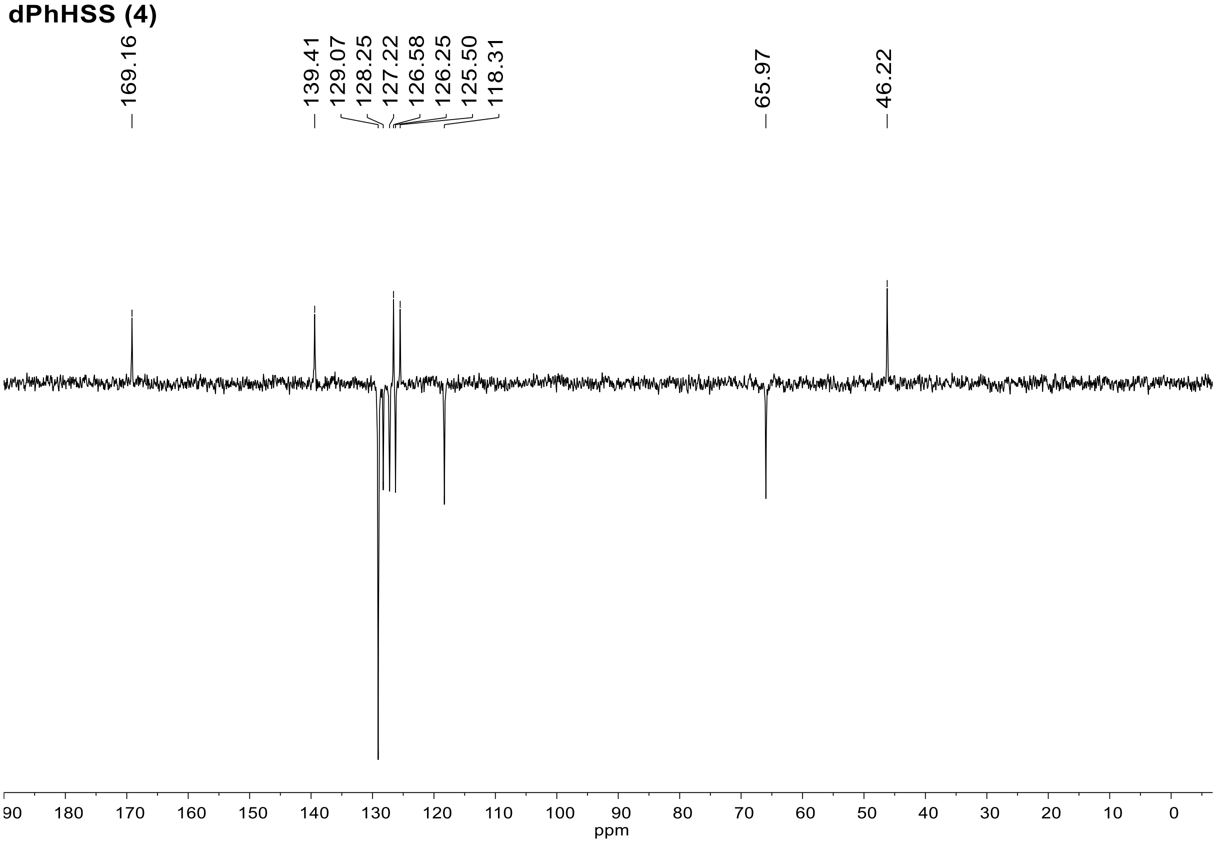

Figure S74. $^{13}C\{^1H\}$ NMR spectrum of **dPhHSS (4)** (90 MHz, 298K, D_2O)

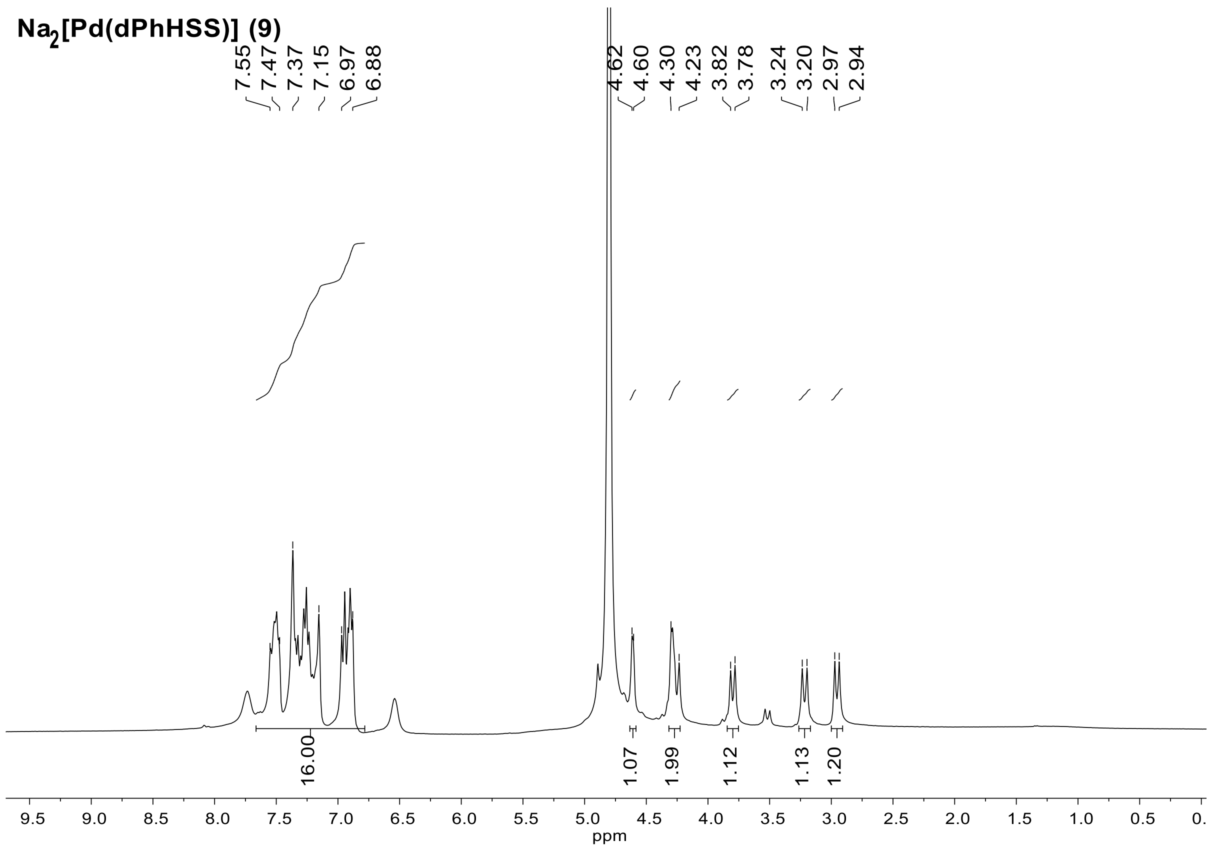

Figure S75. ^{1}H NMR spectrum of **Na_2[Pd(dPhHSS)] (9)** (360 MHz, 298K, D_2O)

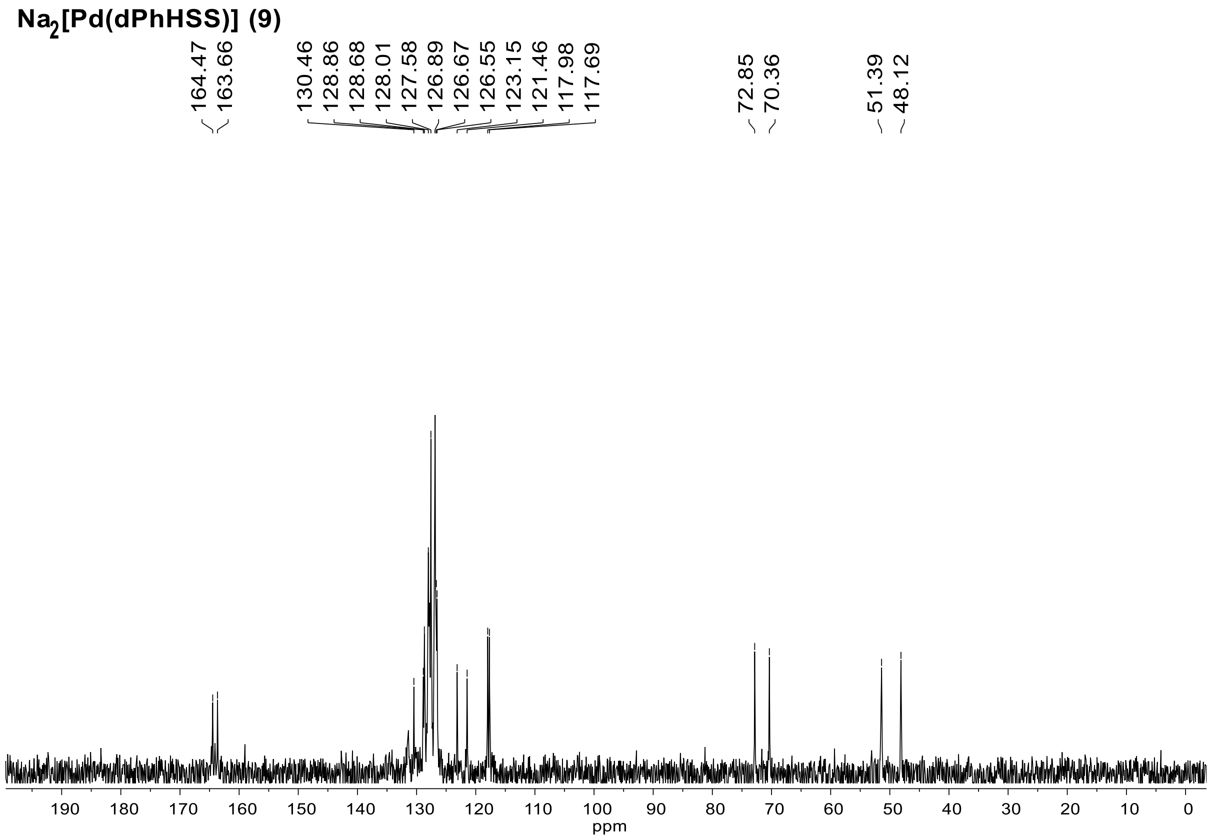

Figure S76. ^{13}C{^{1}H} NMR spectrum of **Na_2[Pd(dPhHSS)] (9)** (90 MHz, 298K, D_2O)

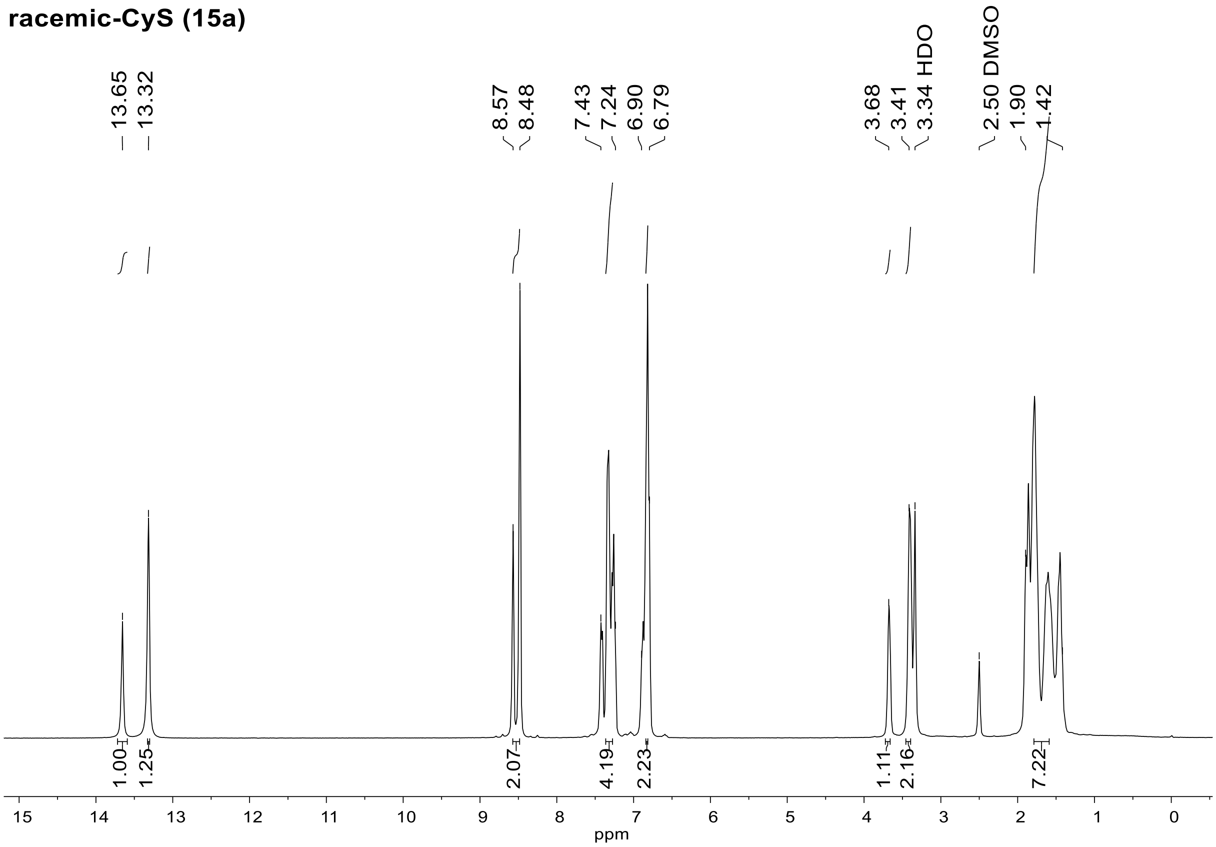

Figure S77. ^{1}H NMR spectrum of ***rac*-CyS (15a)** (360 MHz, 298K, d^{6}-dmso)

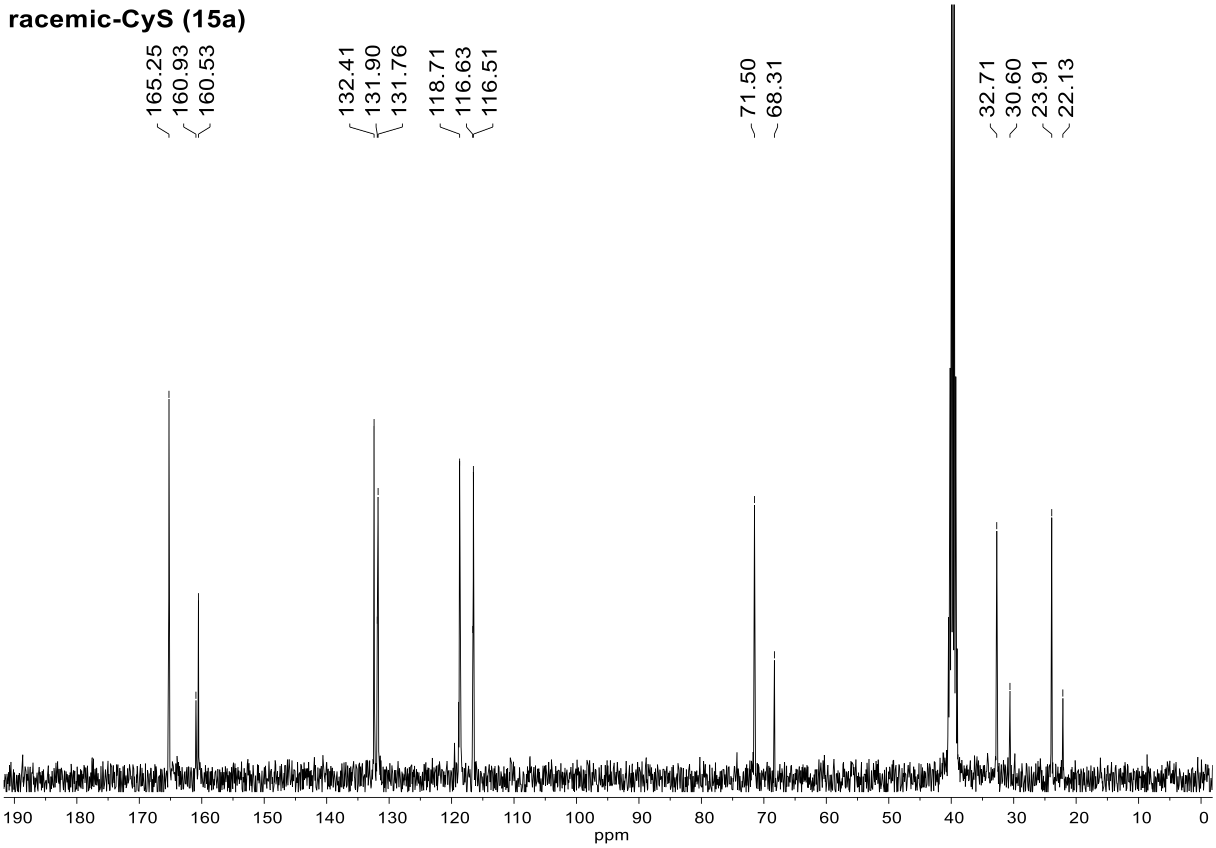

Figure S78. ^{13}C{^{1}H} NMR spectrum of ***rac*-CyS (15a)** (90 MHz, 298K, d^{6}-dmso)

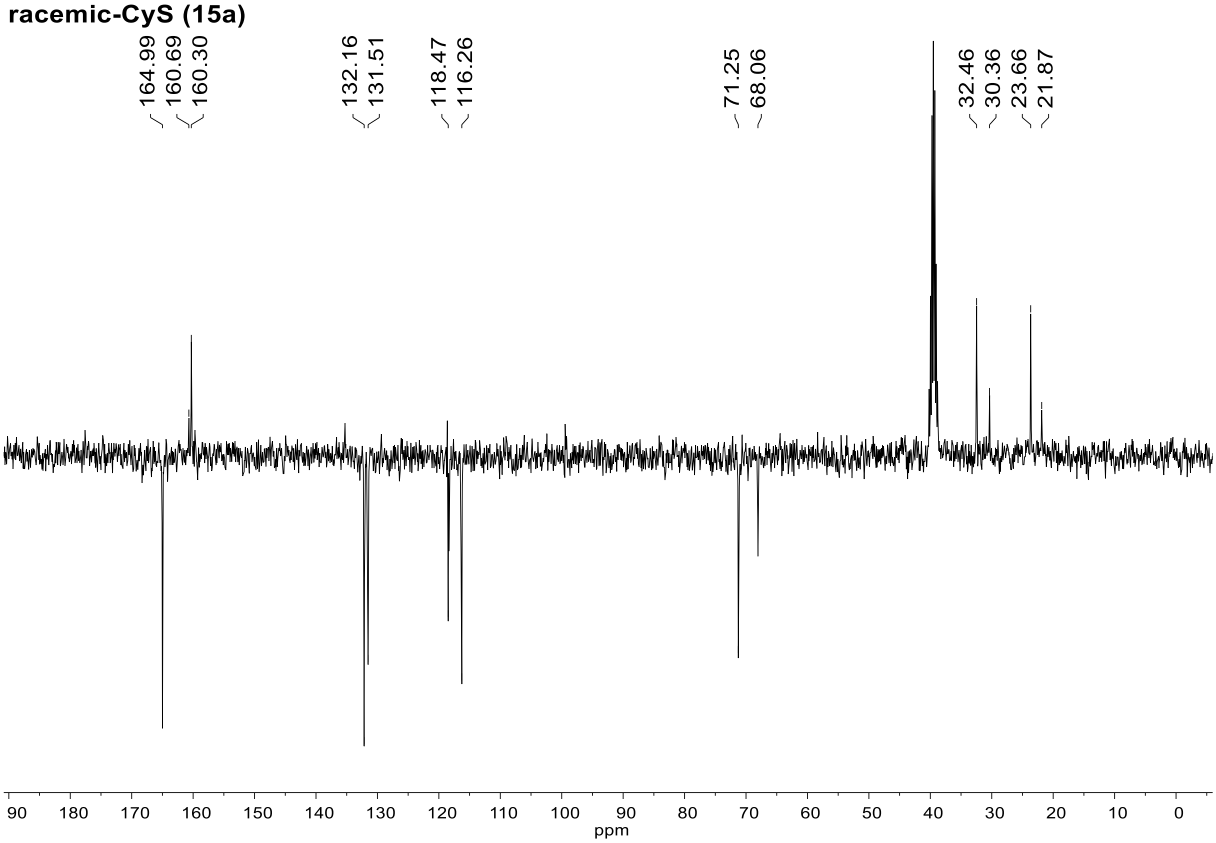

Figure S79. $^{13}C\{^{1}H\}$ NMR spectrum of ***rac*-CyS (15a)** (90 MHz, 298K, d^6-dmso)

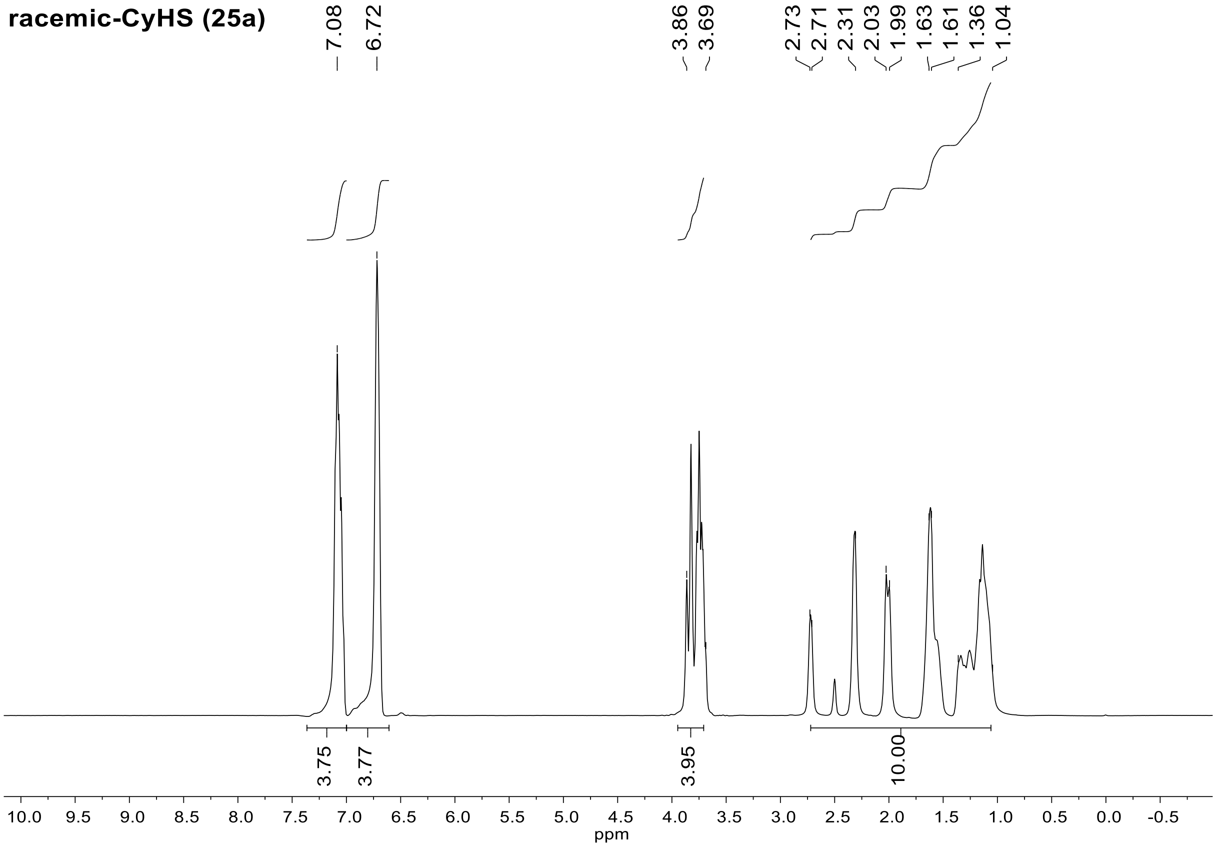

Figure S80. ^{1}H NMR spectrum of ***rac*-CyHS (25a)** (360 MHz, 298K, d^6-dmso)

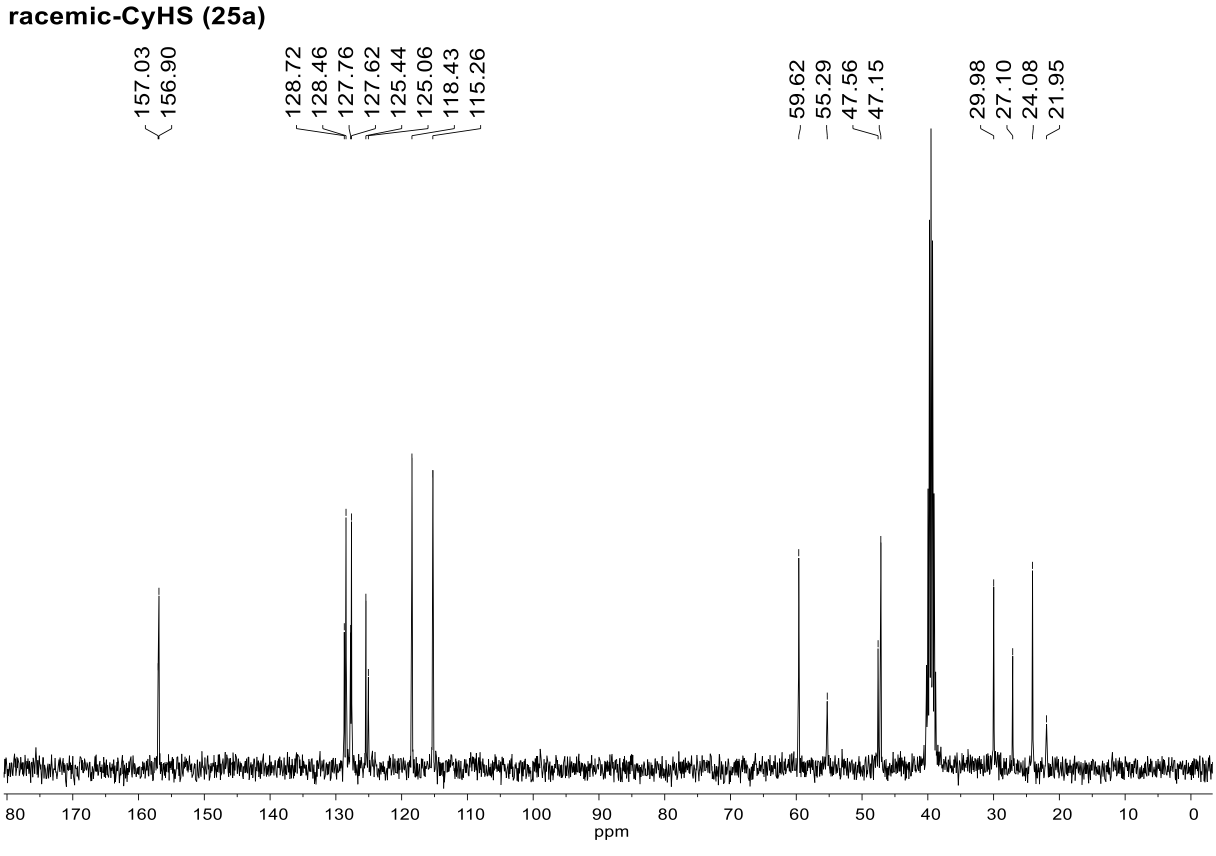

Figure S81. $^{13}C\{^{1}H\}$ NMR spectrum of ***rac*-CyHS (25a)** (90 MHz, 298K, d^6-dmso)

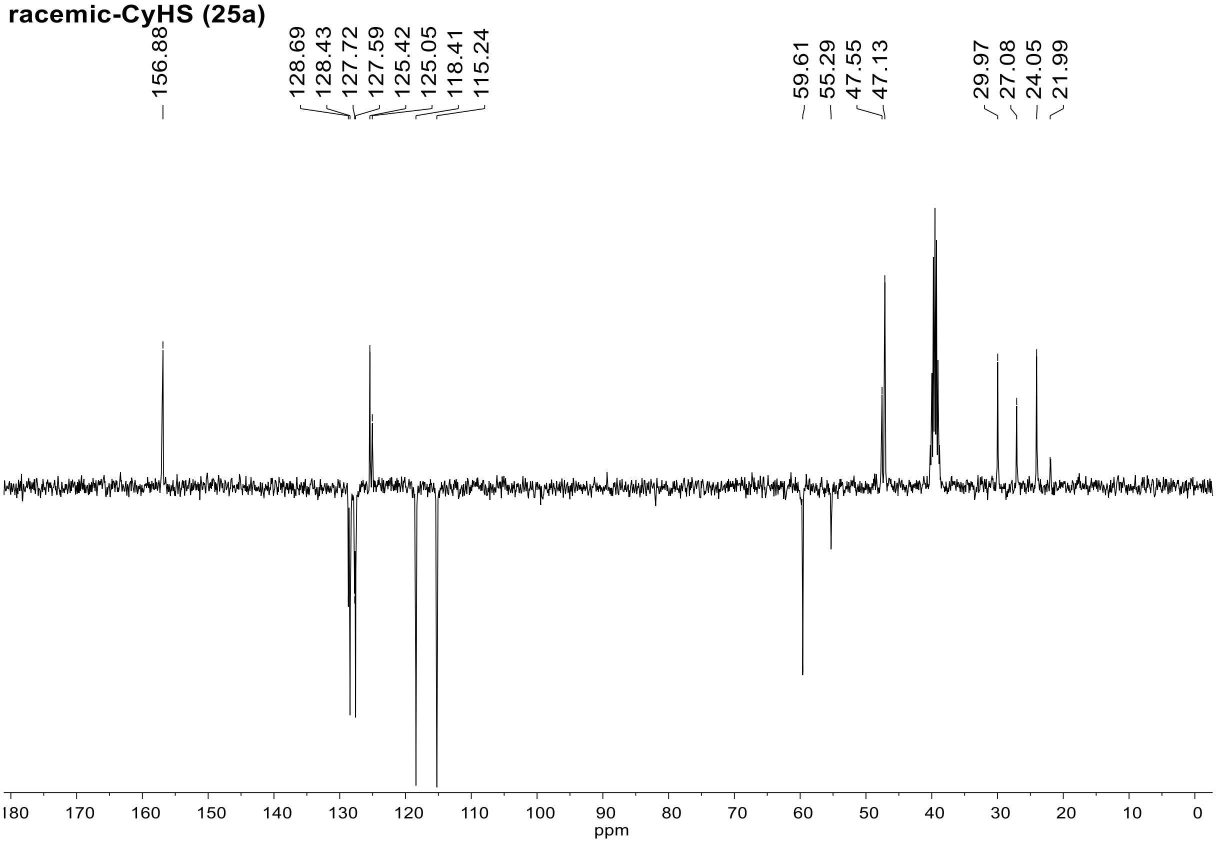

Figure S82. $^{13}C\{^{1}H\}$ NMR spectrum of ***rac*-CyHS (25a)** (90 MHz, 298K, d^6-dmso)

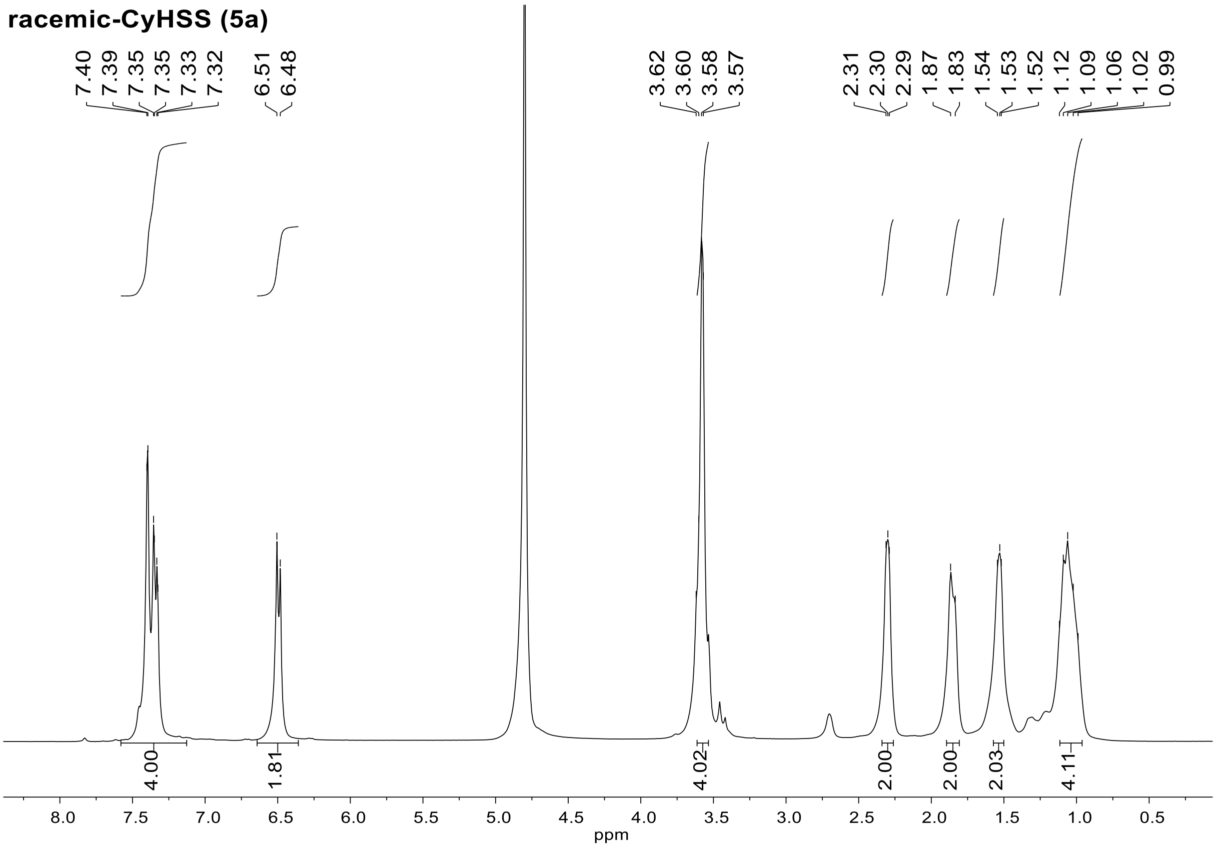

Figure S83. ^{1}H NMR spectrum of ***rac*-CyHSS (5a)** (360 MHz, 298K, D_2O)

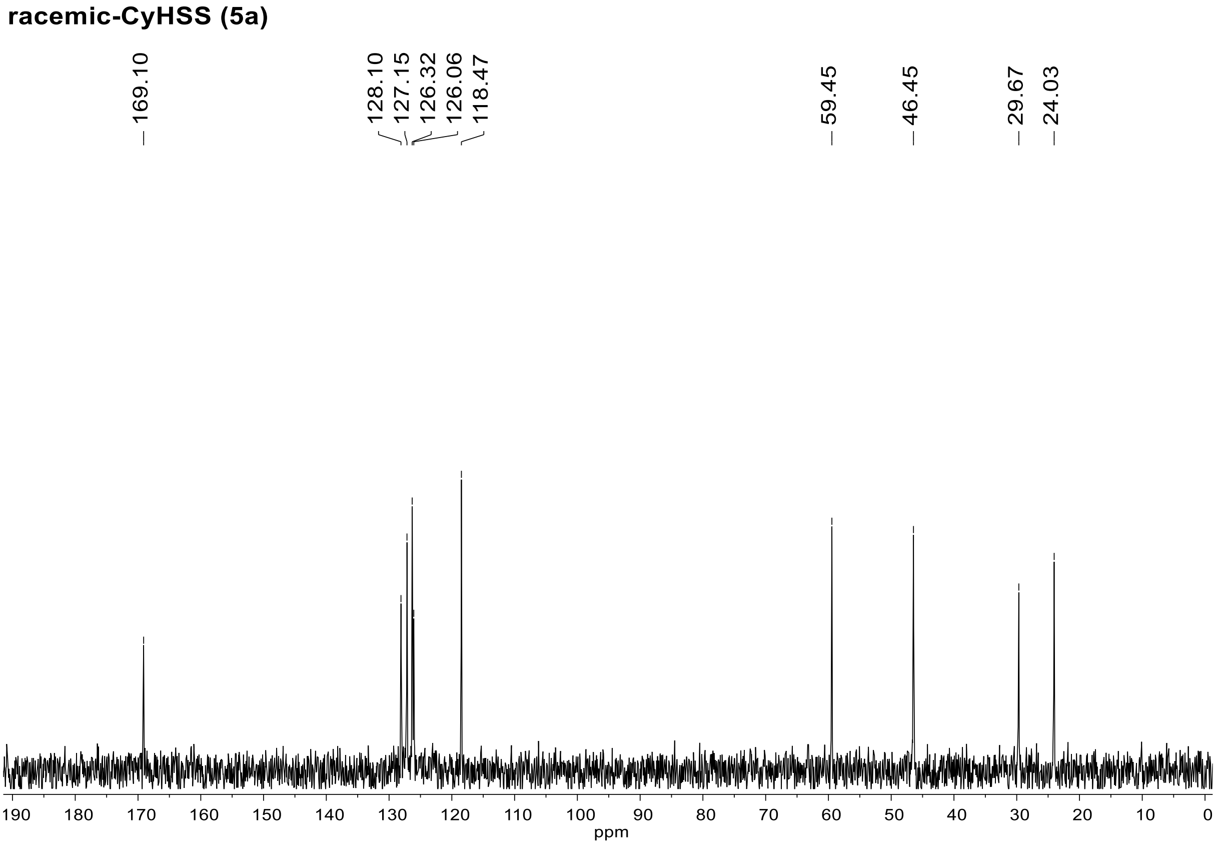

Figure S84. $^{13}C\{^1H\}$ NMR spectrum of ***rac*-CyHSS (5a)** (90 MHz, 298K, D_2O)

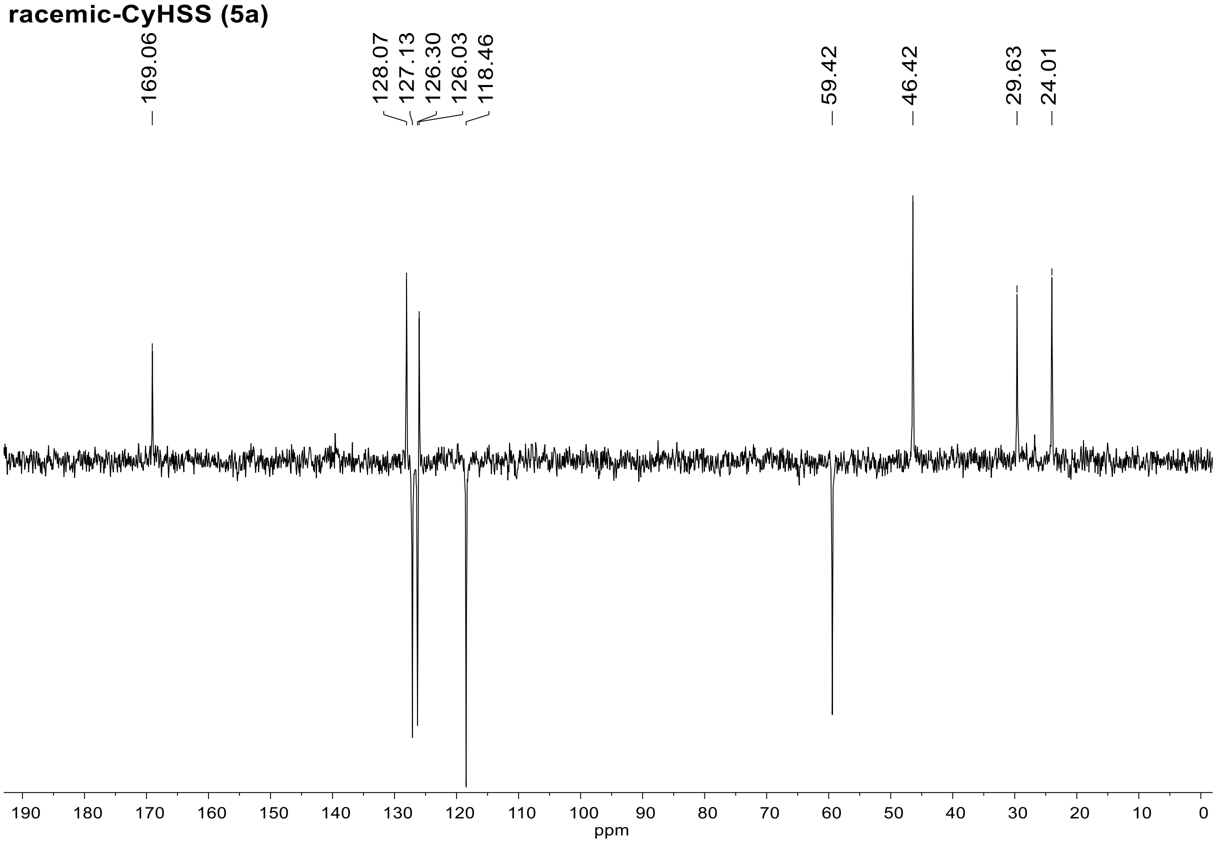

Figure S85. $^{13}C\{^1H\}$ NMR spectrum of ***rac*-CyHSS (5a)** (90 MHz, 298K, D_2O)

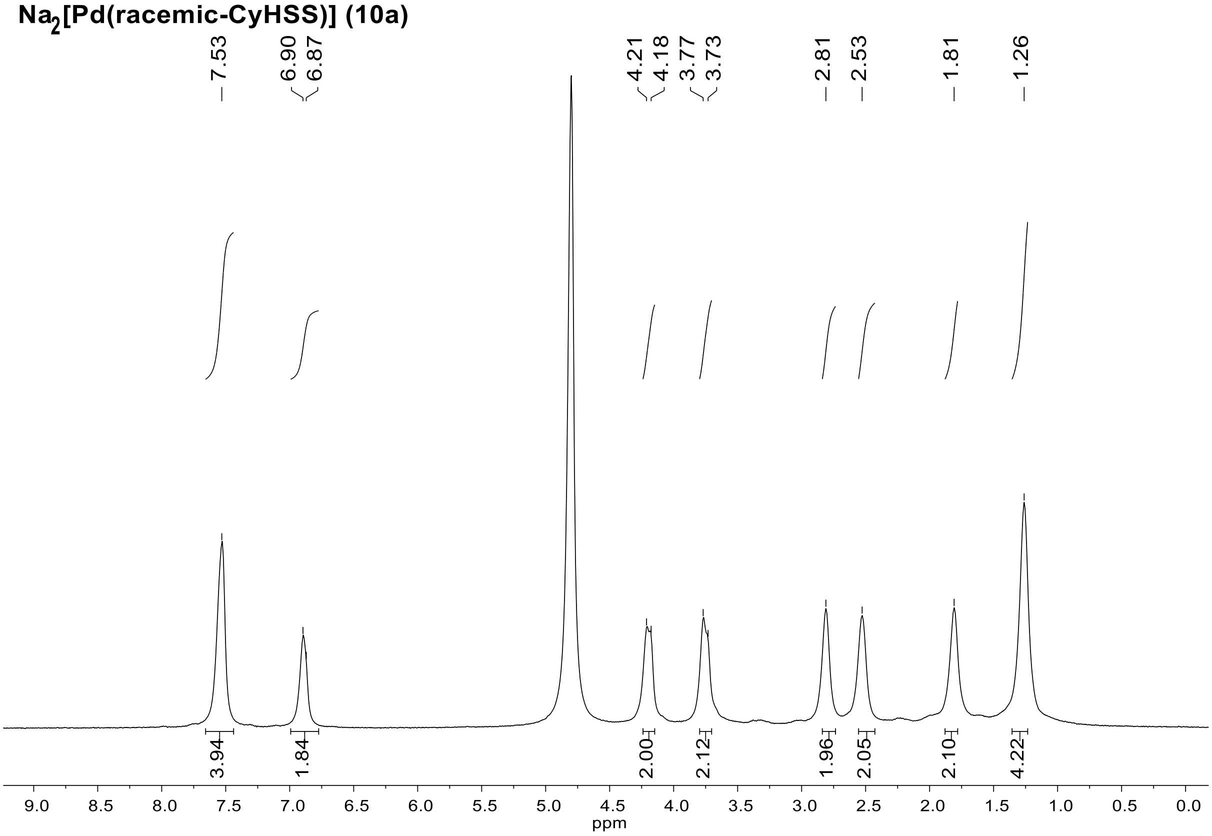

Figure S86.A. ^{1}H NMR spectrum of ***rac*-Na2[Pd(CyHSS)] (10a)** (360 MHz, 298K, D_2O)

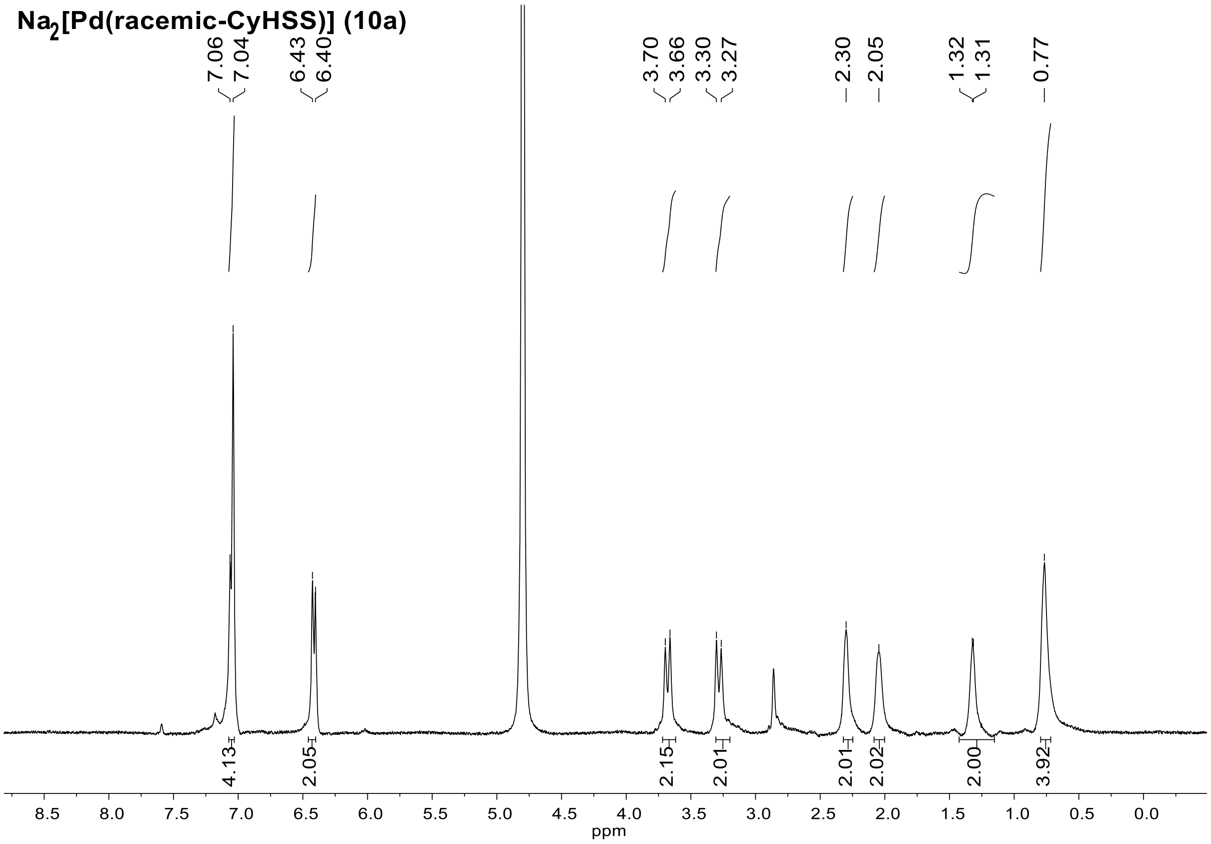

Figure S86.B. ^{1}H NMR spectrum of ***rac*-Na2[Pd(CyHSS)] (10a)** (360 MHz, 268K, D_2O+CD_3OD)

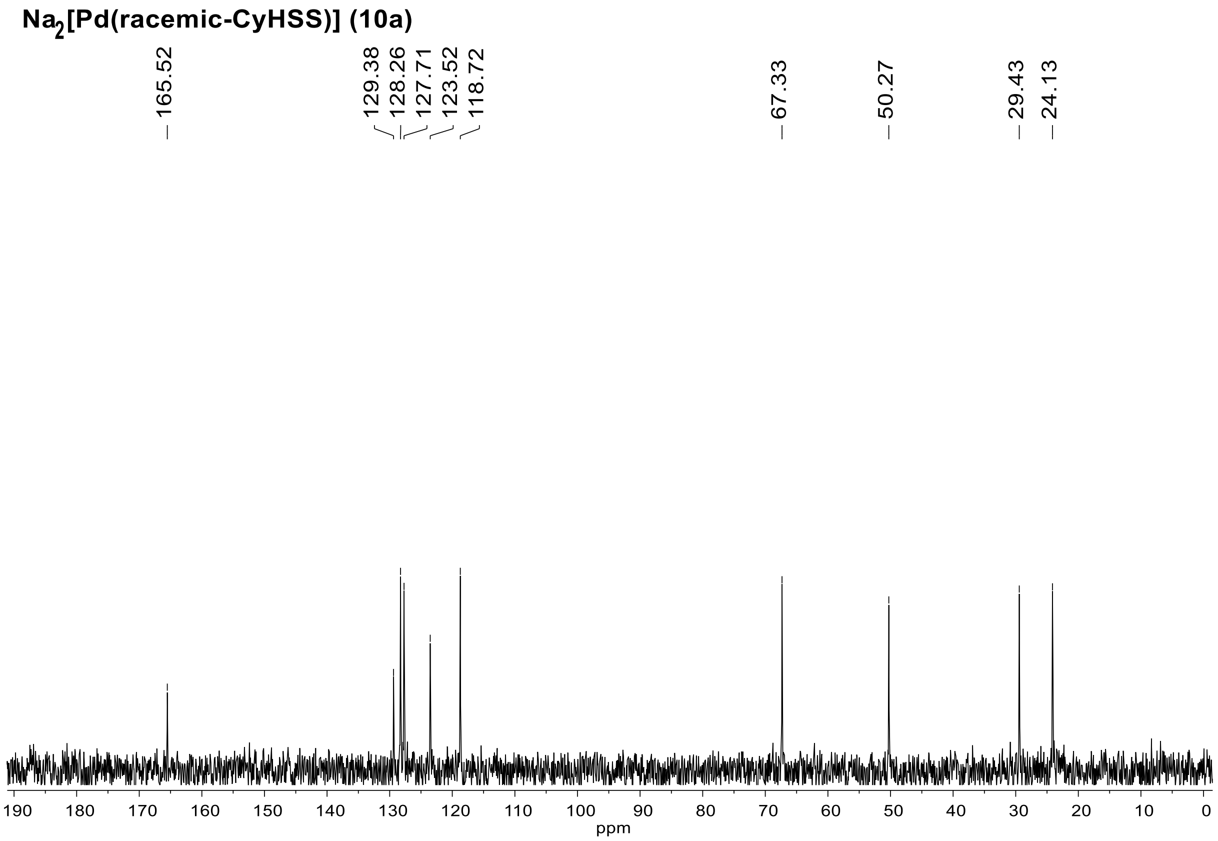

Figure S87. $^{13}C\{^1H\}$ NMR spectrum of ***rac*-Na$_2$[Pd(CyHSS)] (10a)** (90 MHz, 298K, D_2O)

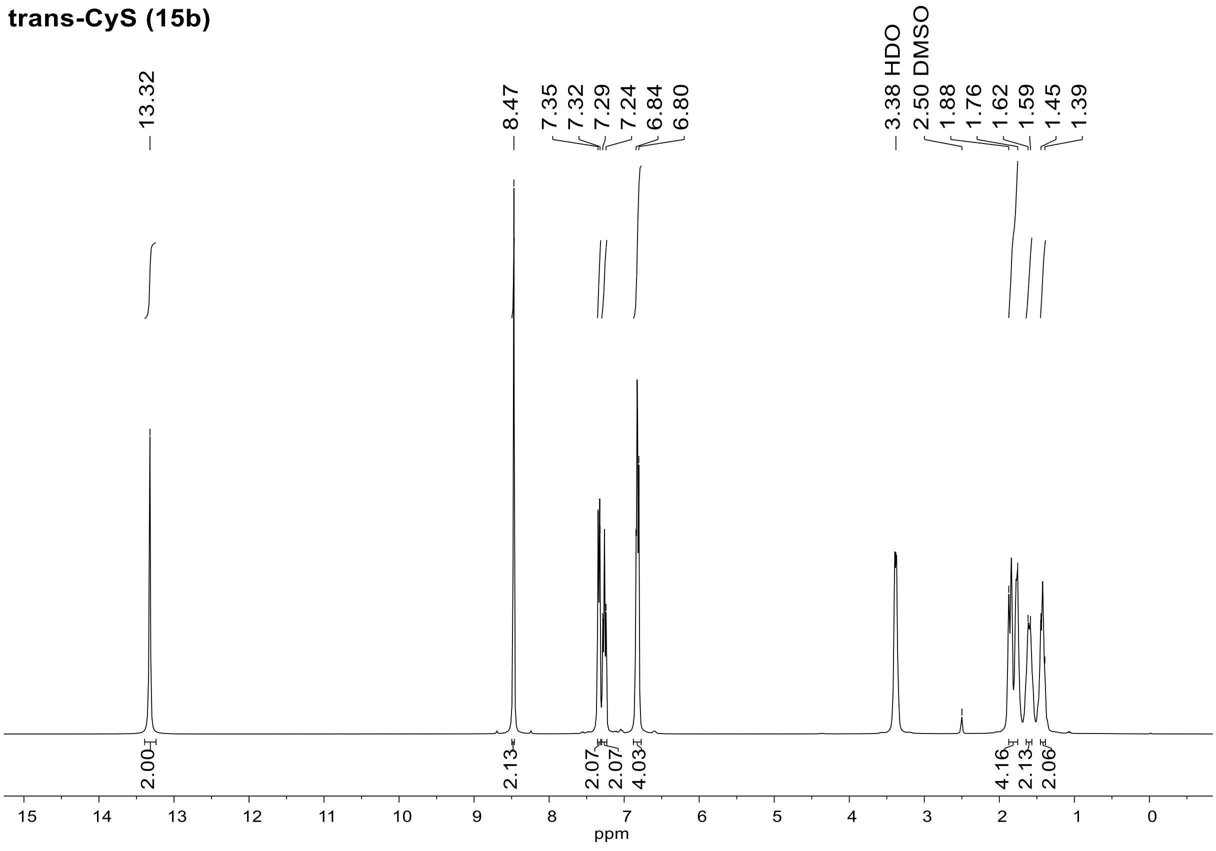

Figure S88. ^{1}H NMR spectrum of ***trans*-CyS (15b)** (360 MHz, 298K, d^6-dmso)

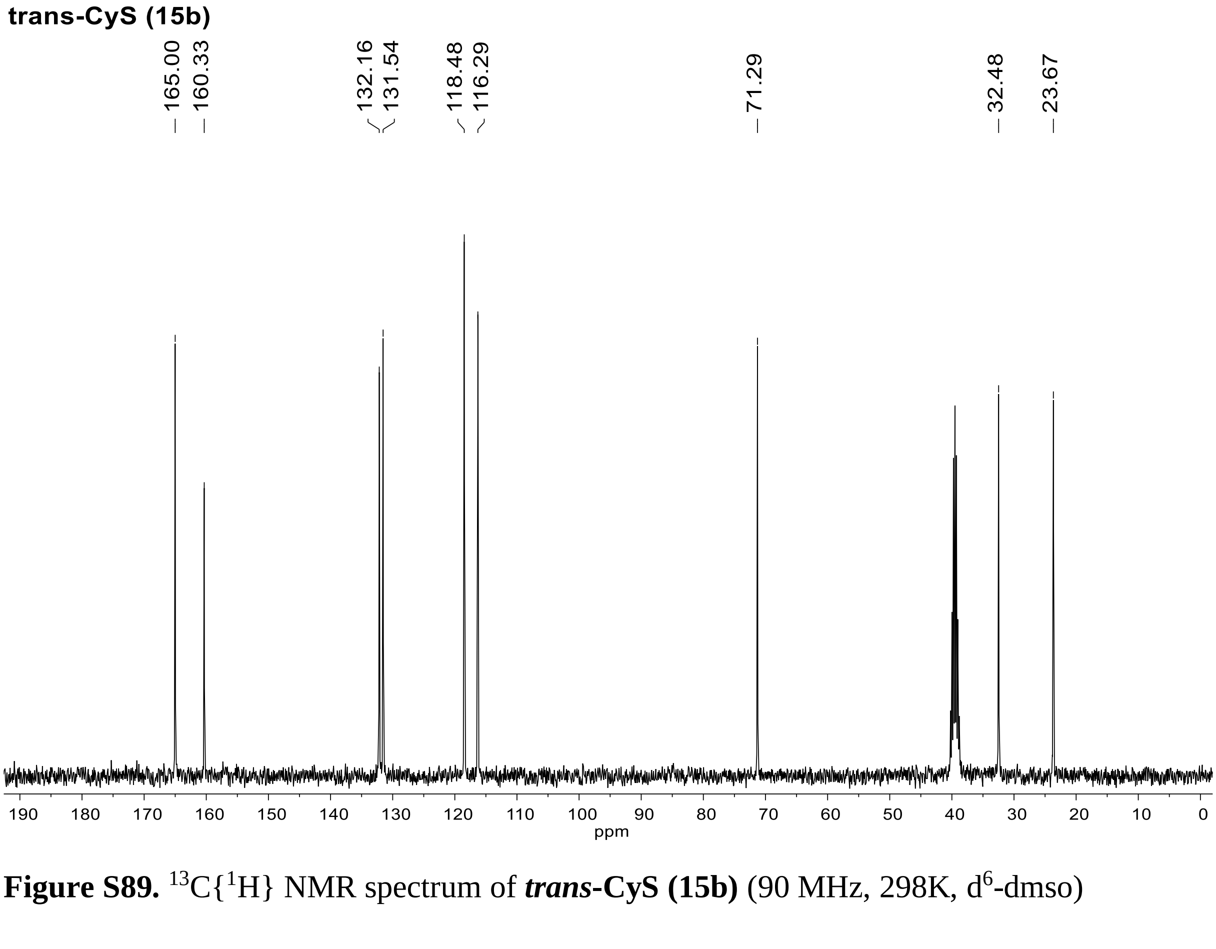

Figure S89. ^{13}C{^{1}H} NMR spectrum of ***trans*-CyS (15b)** (90 MHz, 298K, d^6-dmso)

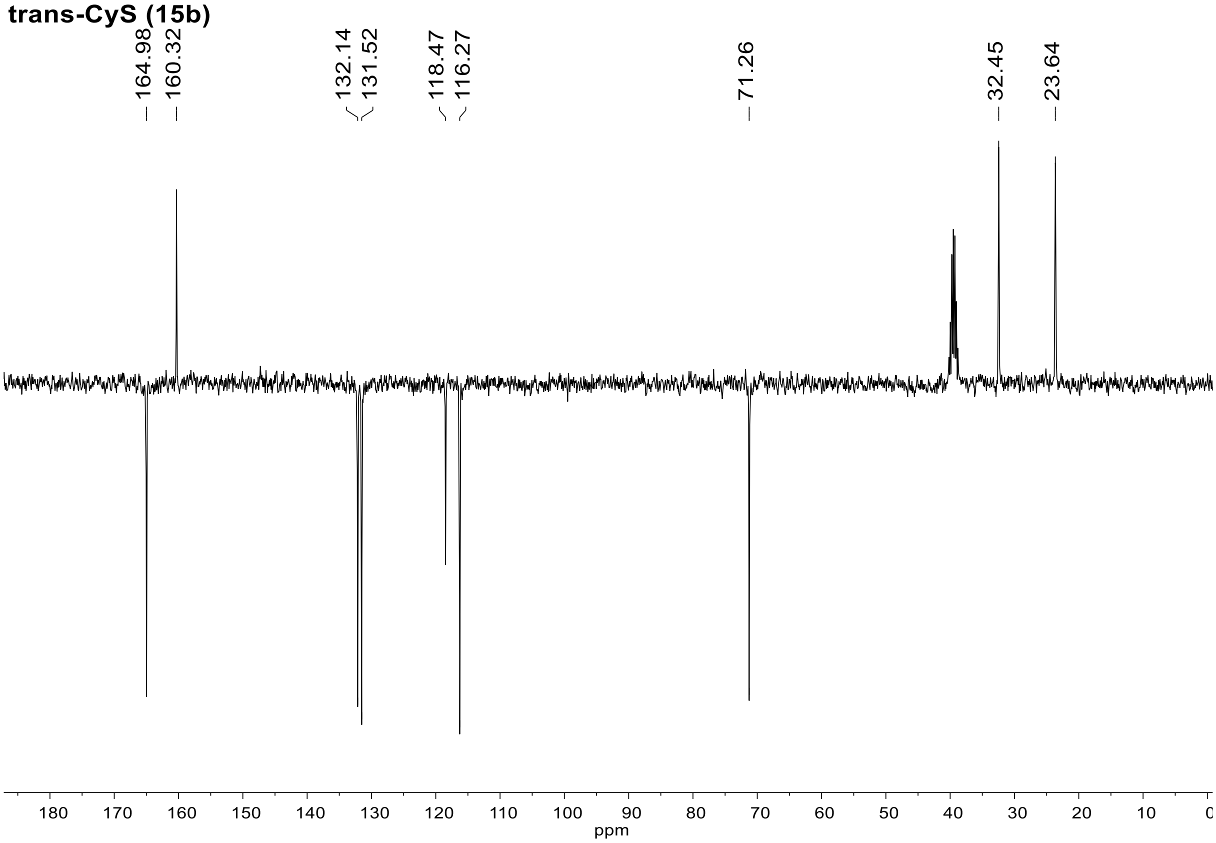

Figure S90. $^{13}C\{^{1}H\}$ NMR spectrum of ***trans*-CyS (15b)** (90 MHz, 298K, d^6-dmso)

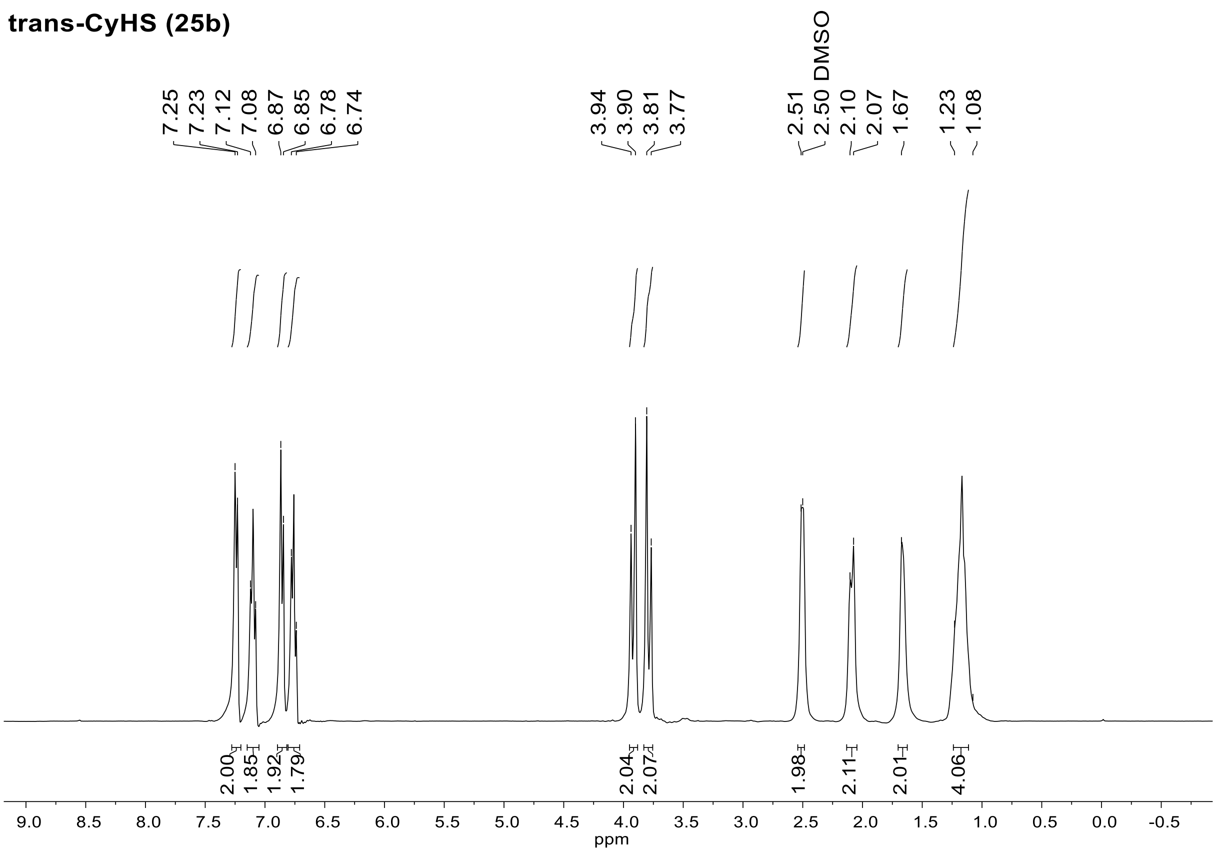

Figure S91. ^{1}H NMR spectrum of ***trans*-CyHS (25b)** (360 MHz, 298K, d^{6}-dmso)

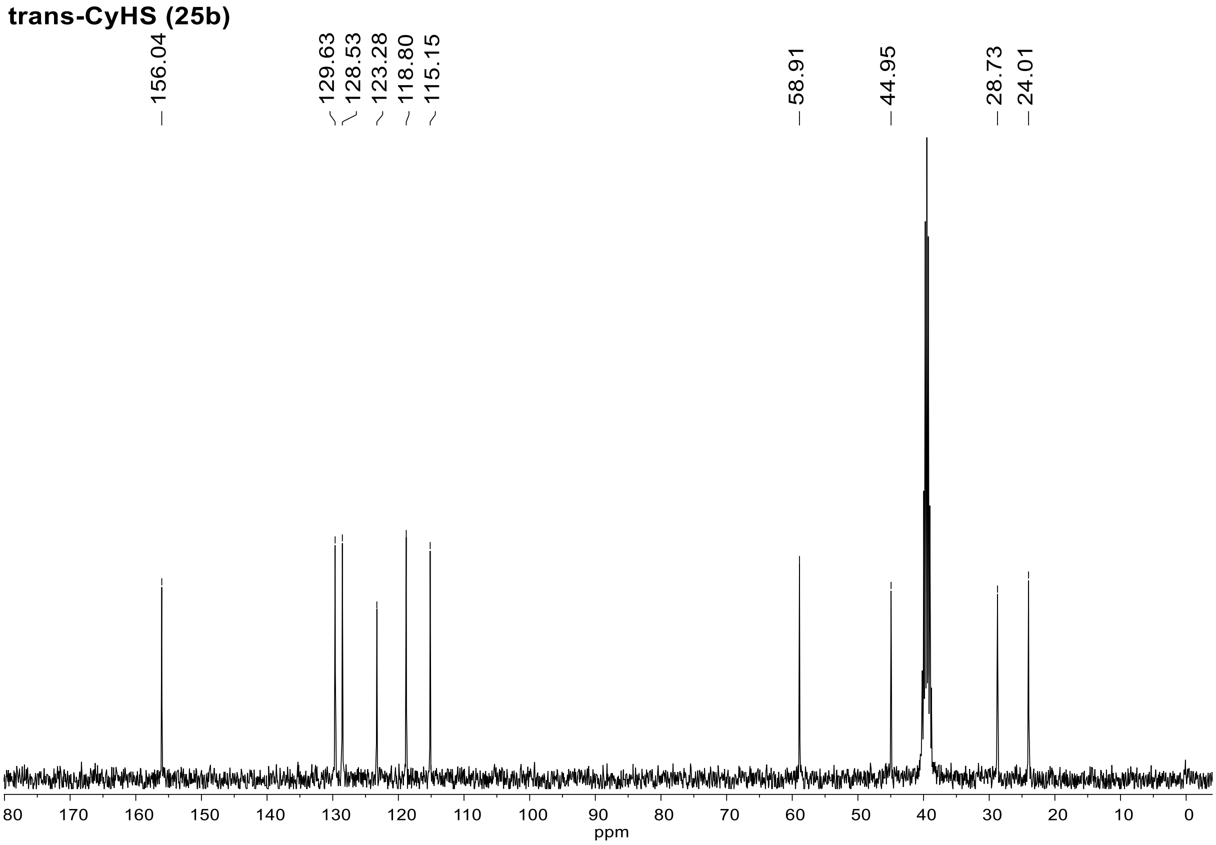

Figure S92. ^{13}C{^{1}H} NMR spectrum of ***trans*-CyHS (25b)** (90 MHz, 298K, d^{6}-dmso)

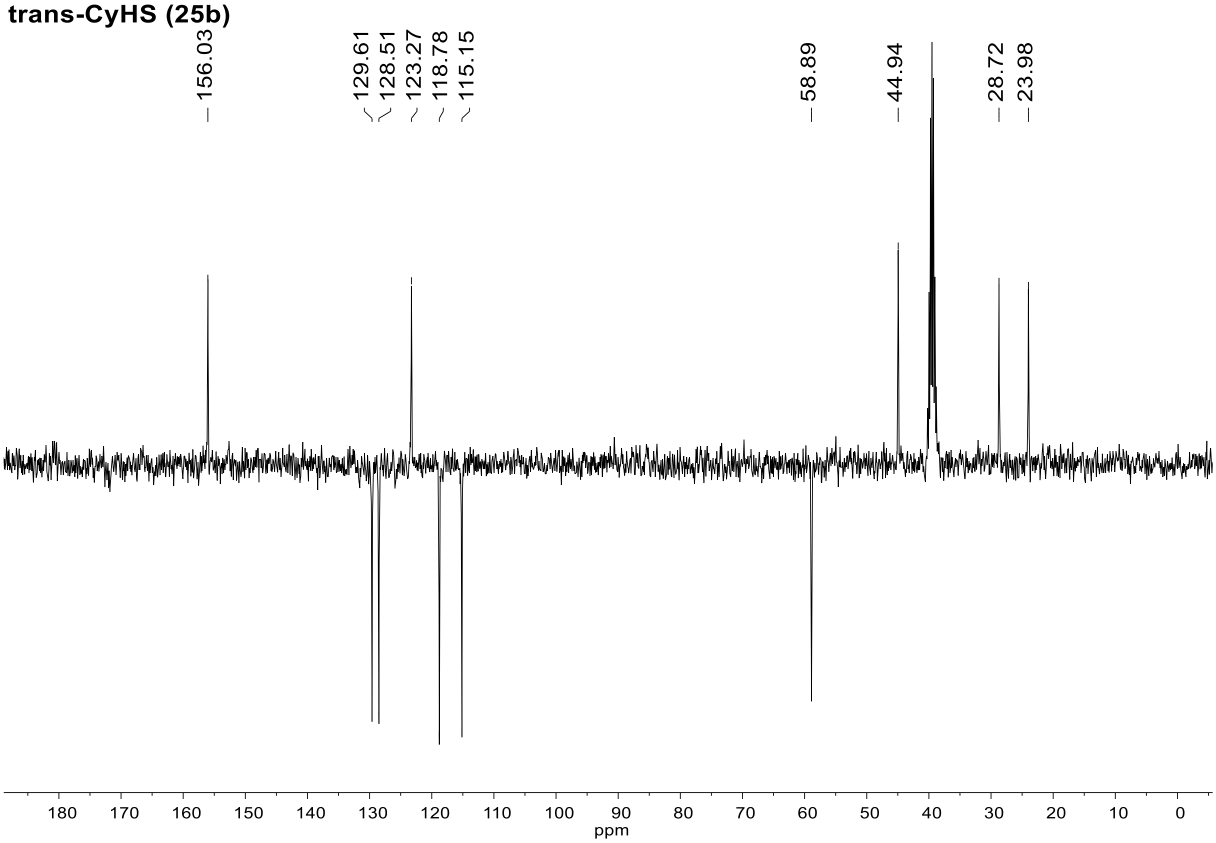

Figure S93. $^{13}C\{^{1}H\}$ NMR spectrum of ***trans*-CyHS (25b)** (90 MHz, 298K, d^6-dmso)

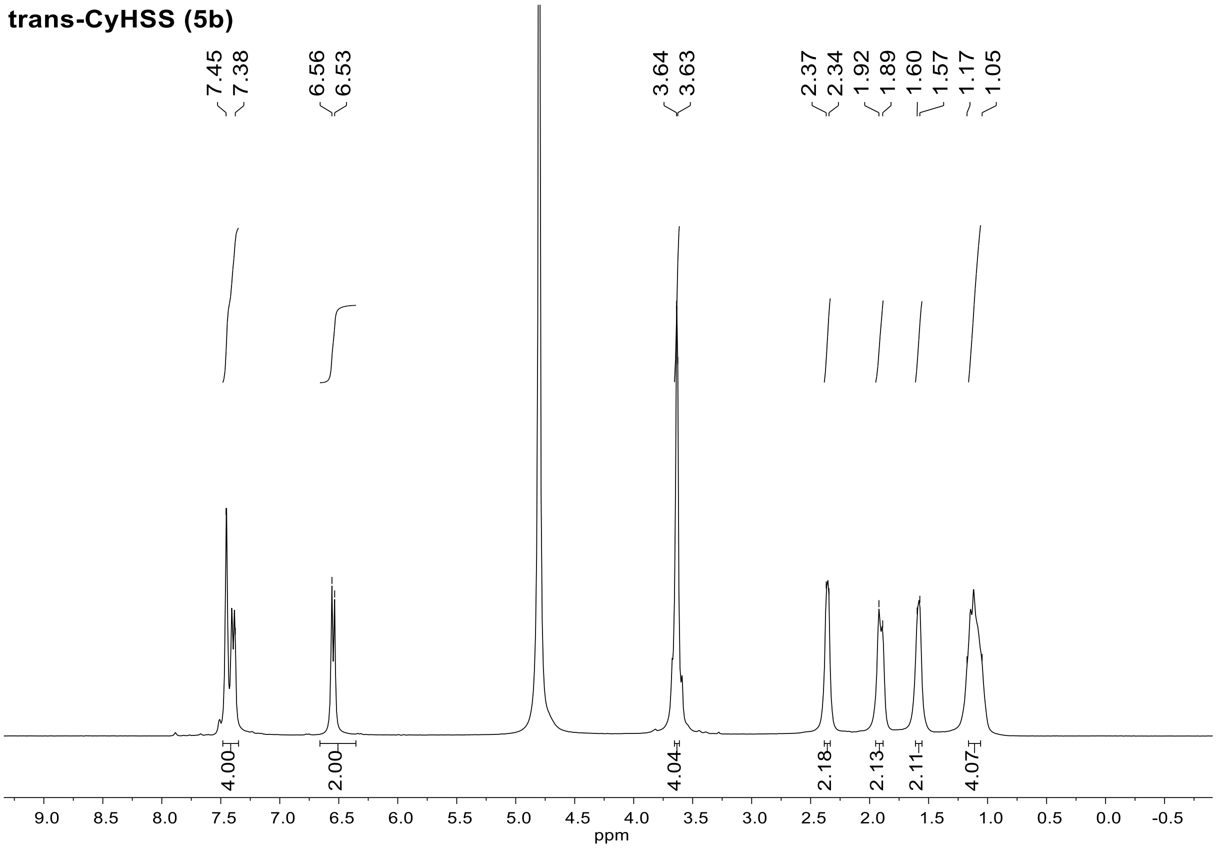

Figure S94. ^{1}H NMR spectrum of ***trans*-CyHSS (5b)** (360 MHz, 298K, D_2O)

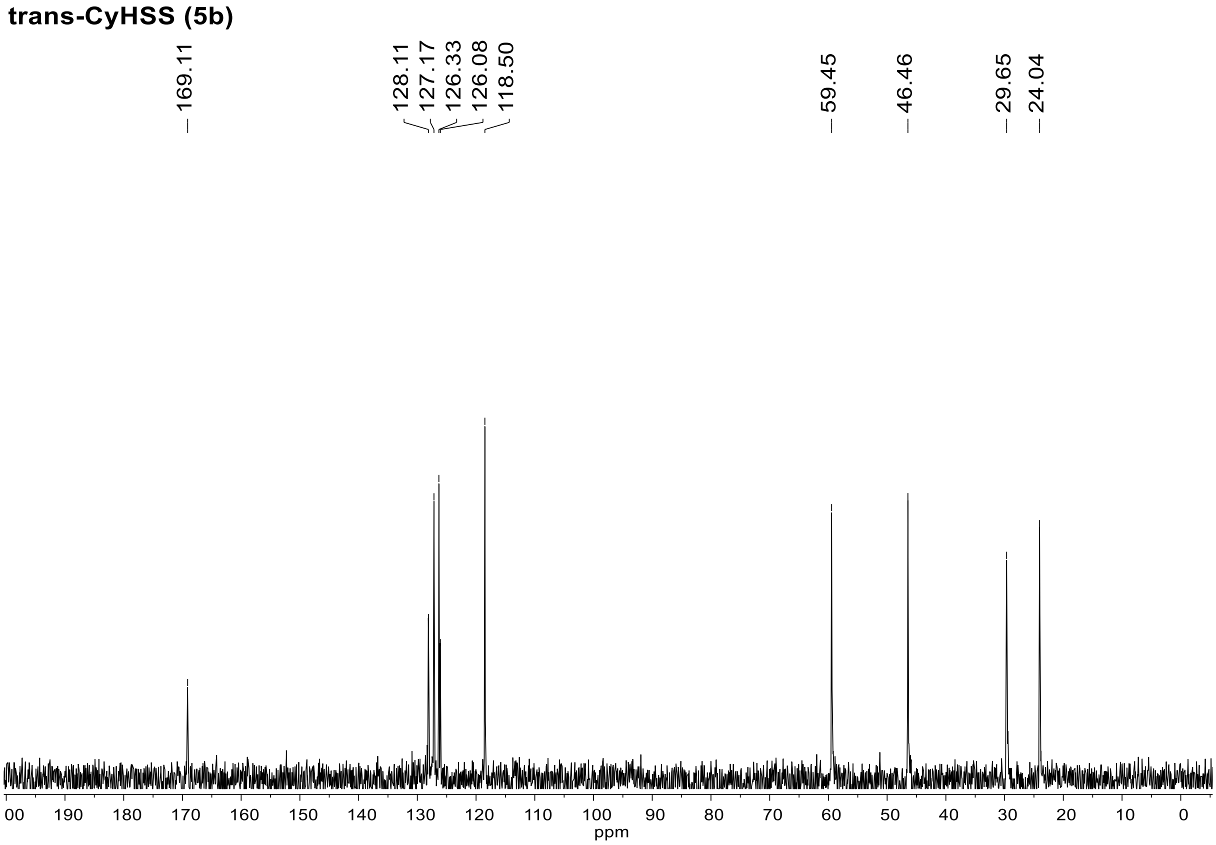

Figure S95. $^{13}C\{^1H\}$ NMR spectrum of ***trans*-CyHSS (5b)** (90 MHz, 298K, D_2O)

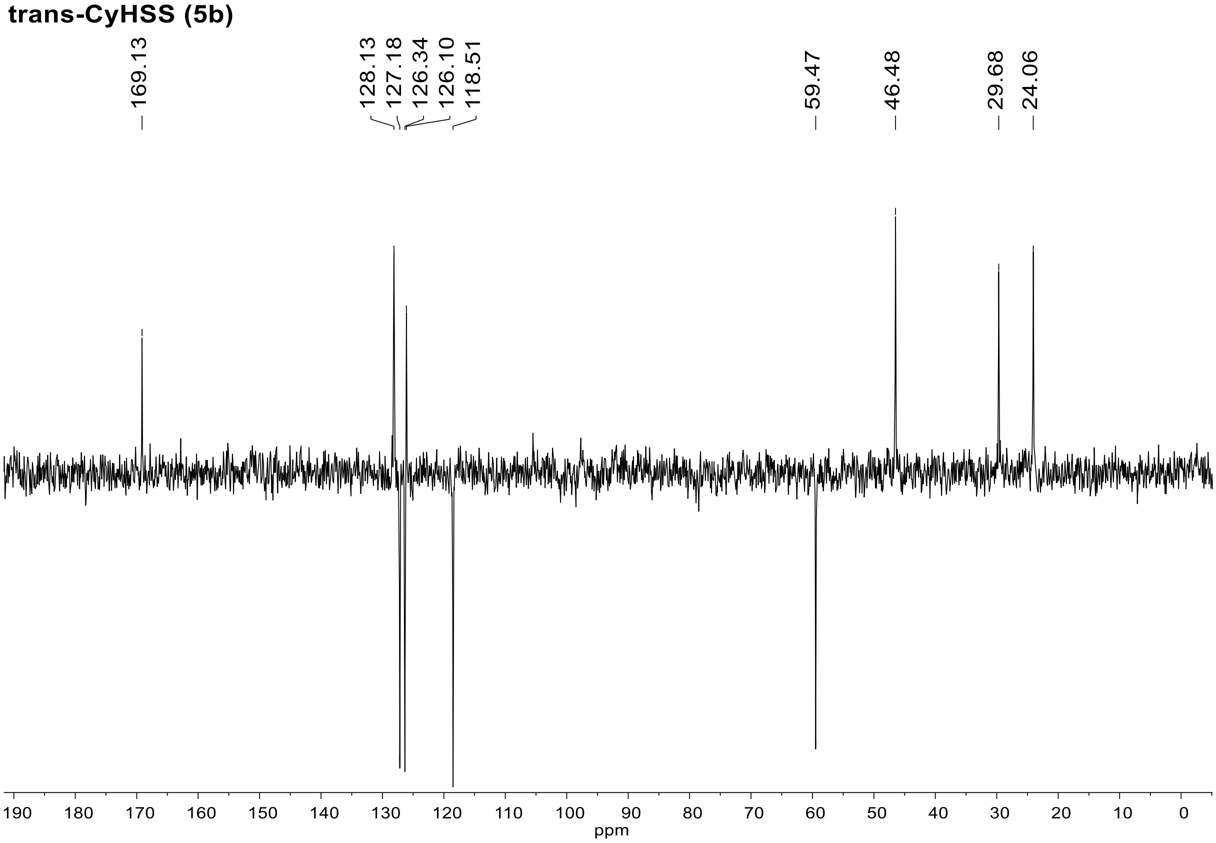

Figure S96. $^{13}C\{^{1}H\}$ NMR spectrum of ***trans*-CyHSS (5b)** (90 MHz, 298K, D_2O)

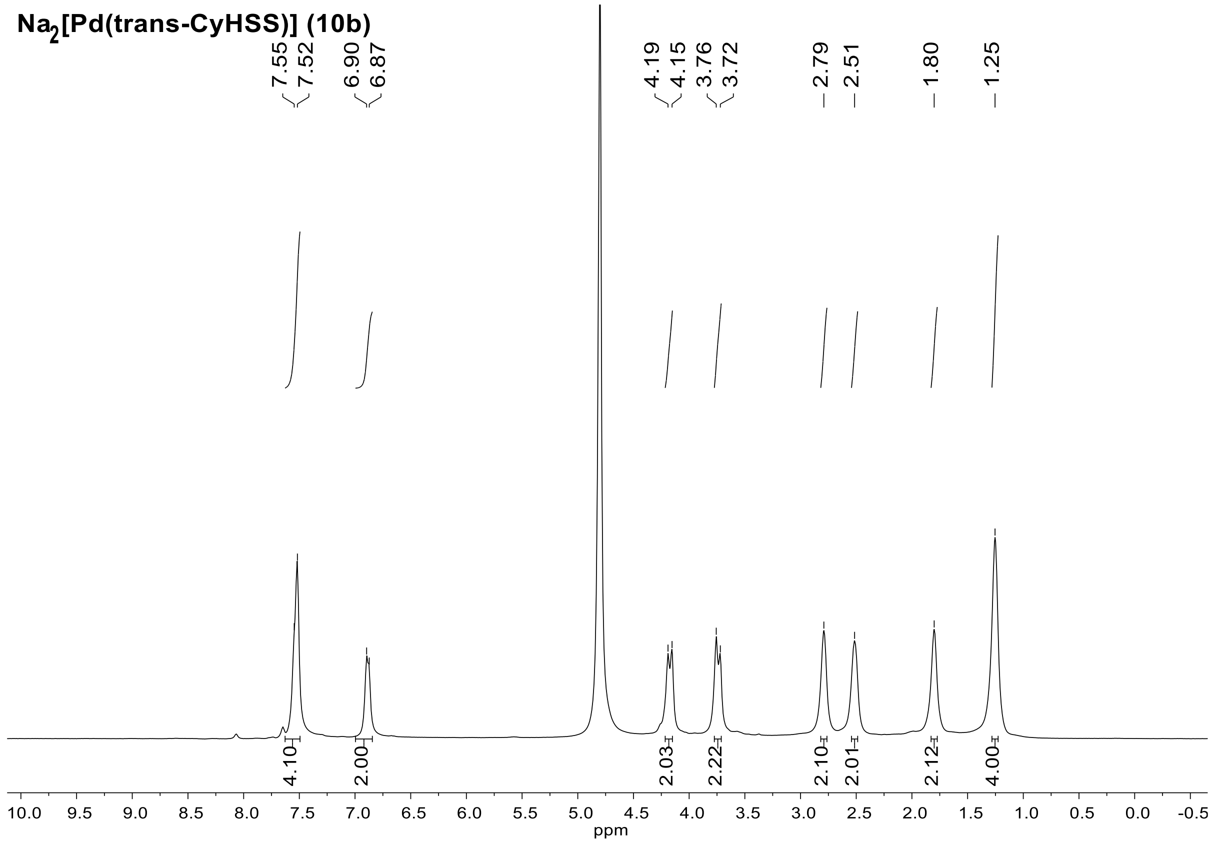

Figure S97.A. ^{1}H NMR spectrum of **Na_2[Pd(*trans*-CyHSS)] (10b)** (360 MHz, 298K, D_2O)

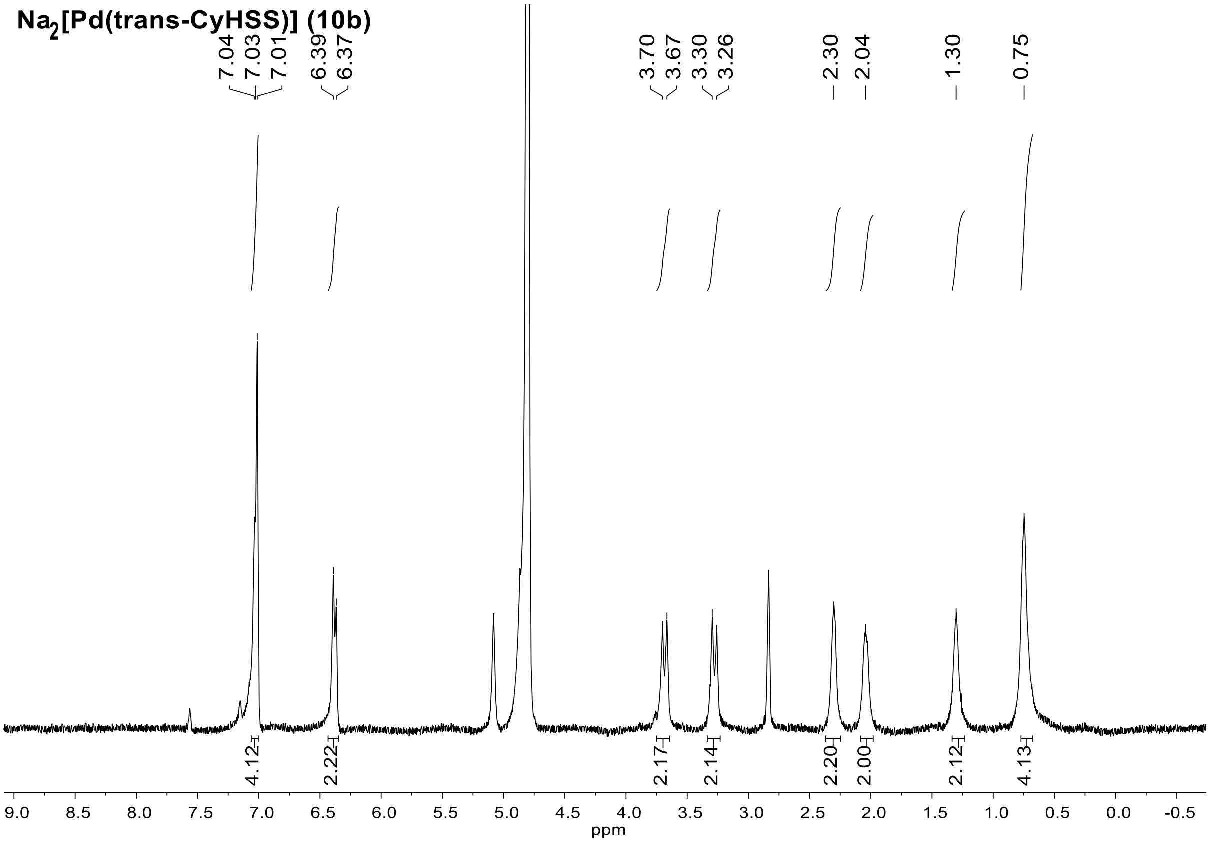

Figure S97.B. ^{1}H NMR spectrum of **Na_2[Pd(*trans*-CyHSS)] (10b)** (360 MHz, 268K, D_2O+CD_3OD)

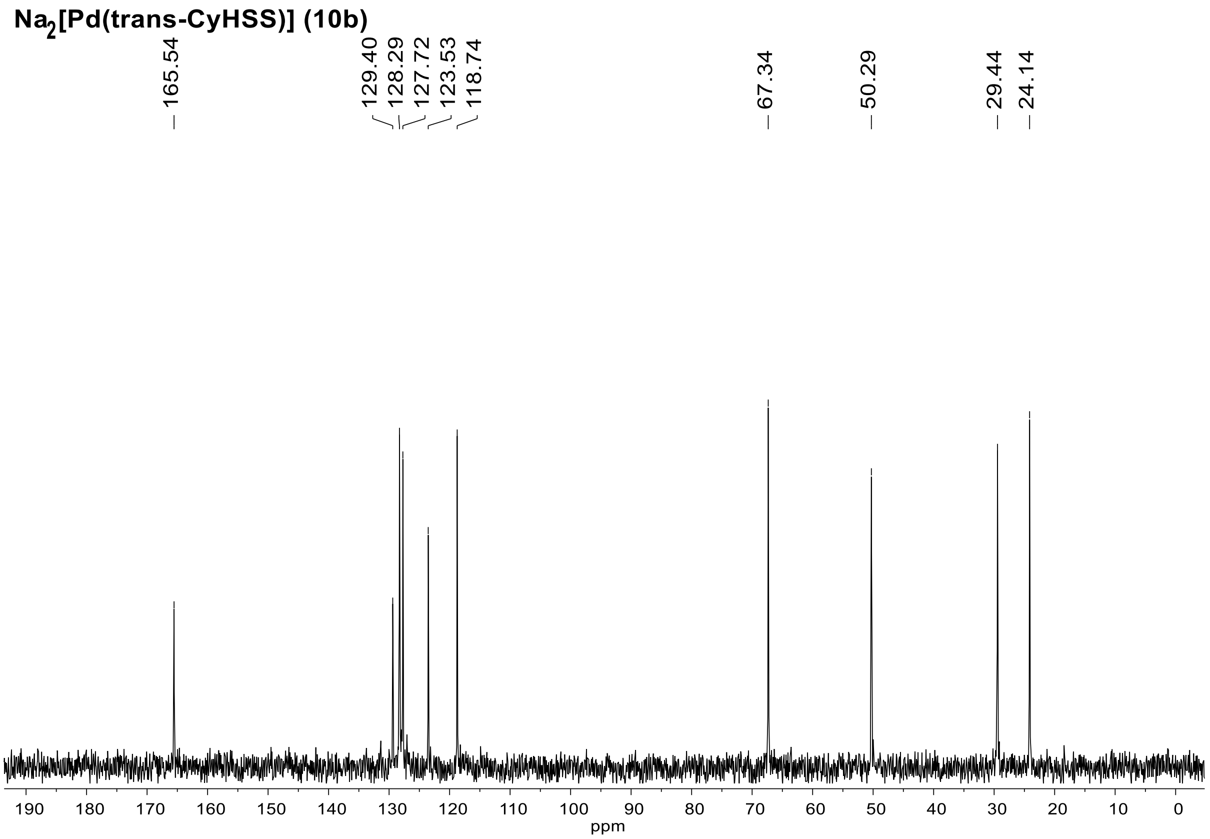

Figure S98. $^{13}C\{^1H\}$ NMR spectrum of **Na_2[Pd(*trans*-CyHSS)] (10b)** (90 MHz, 298K, D_2O)

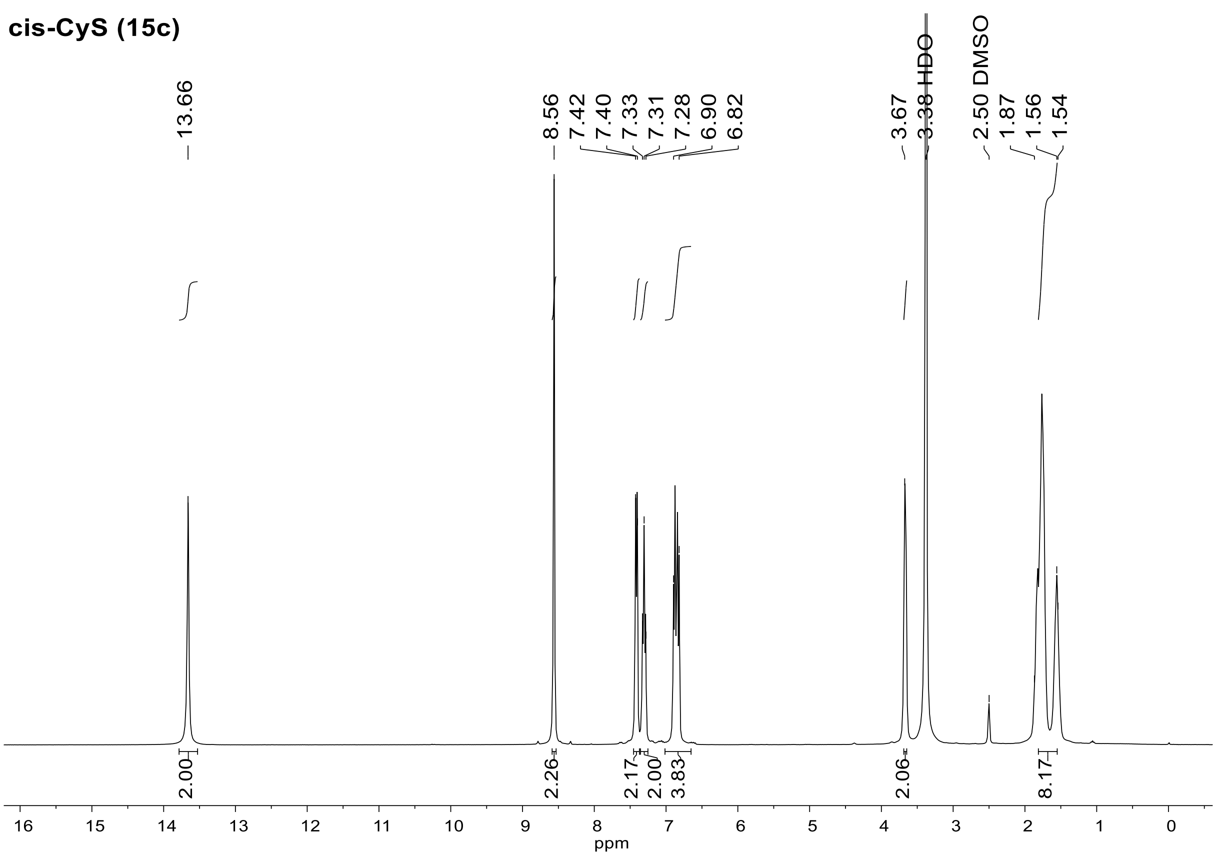

Figure S99. ^{1}H NMR spectrum of ***cis*-CyS (15c)** (360 MHz, 298K, d^{6}-dmso)

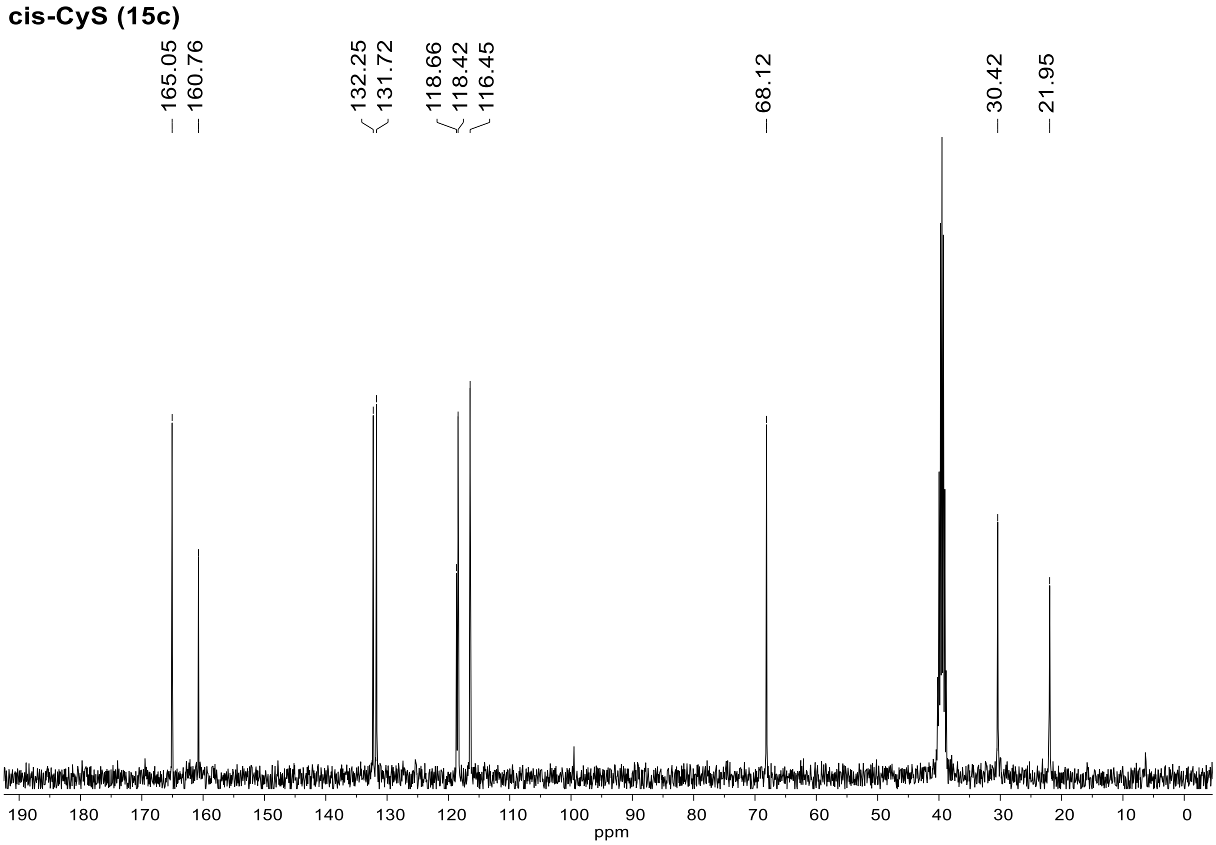

Figure S100. ^{13}C{^{1}H} NMR spectrum of ***cis*-CyS (15c)** (90 MHz, 298K, d^{6}-dmso)

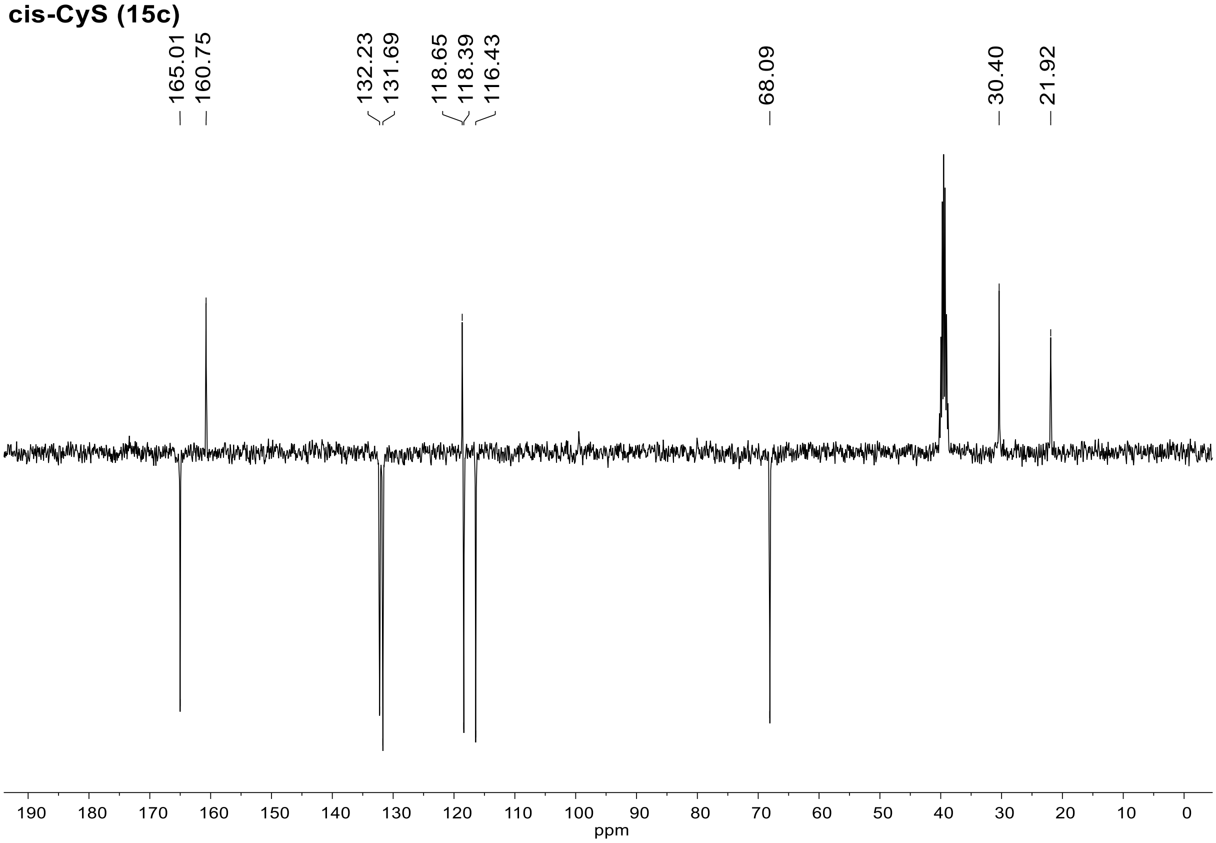

Figure S101. $^{13}C\{^1H\}$ NMR spectrum of ***cis*-CyS (15c)** (90 MHz, 298K, d^6-dmso)

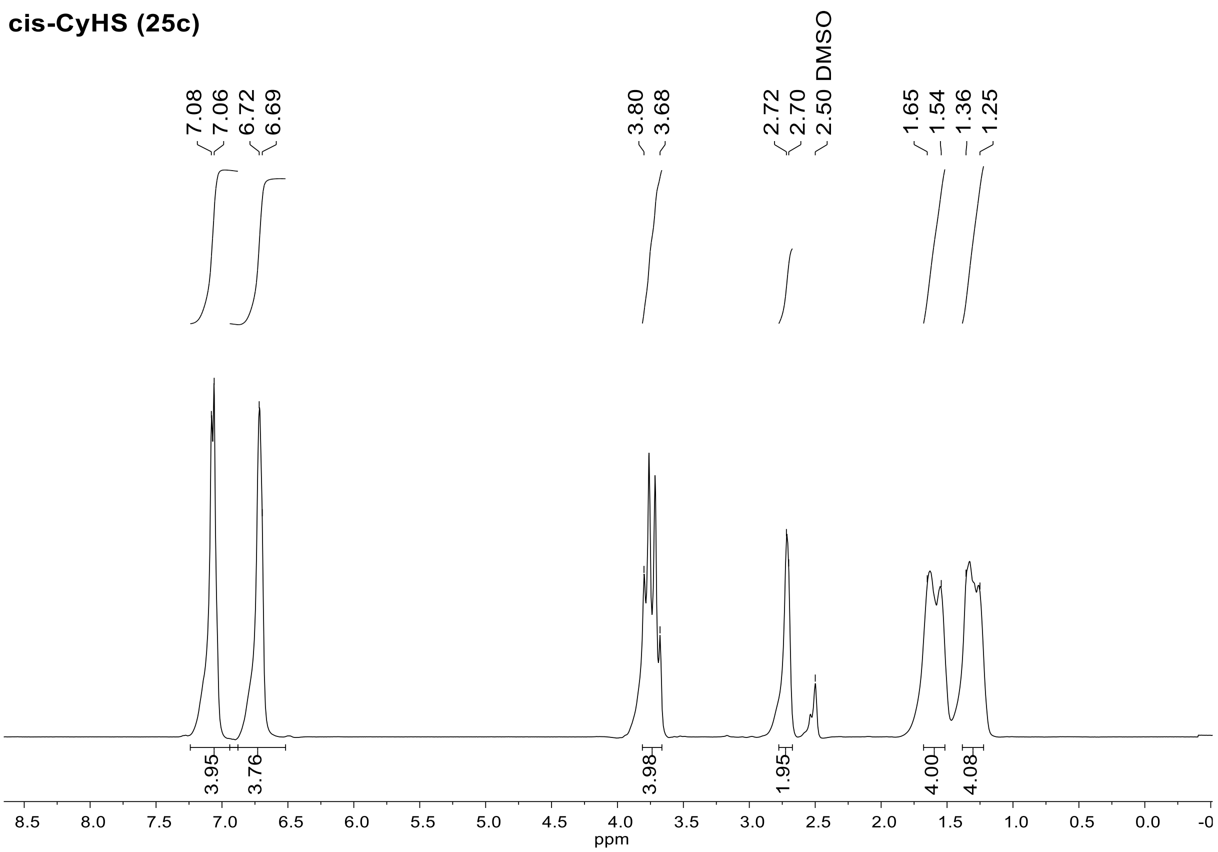

Figure S102. ^{1}H NMR spectrum of ***cis*-CyHS (25c)** (360 MHz, 298K, d^{6}-dmso)

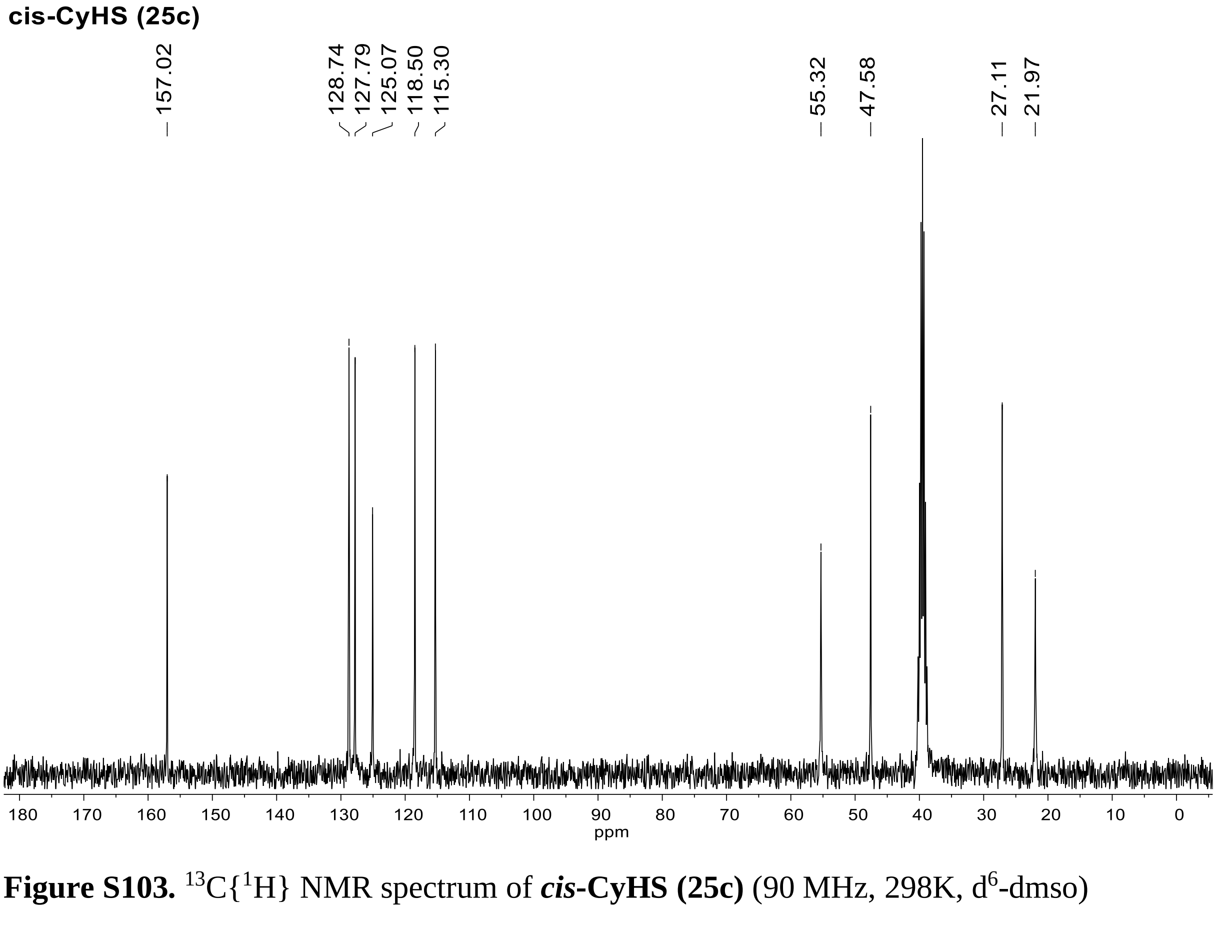

Figure S103. ^{13}C{^{1}H} NMR spectrum of ***cis*-CyHS (25c)** (90 MHz, 298K, d^{6}-dmso)

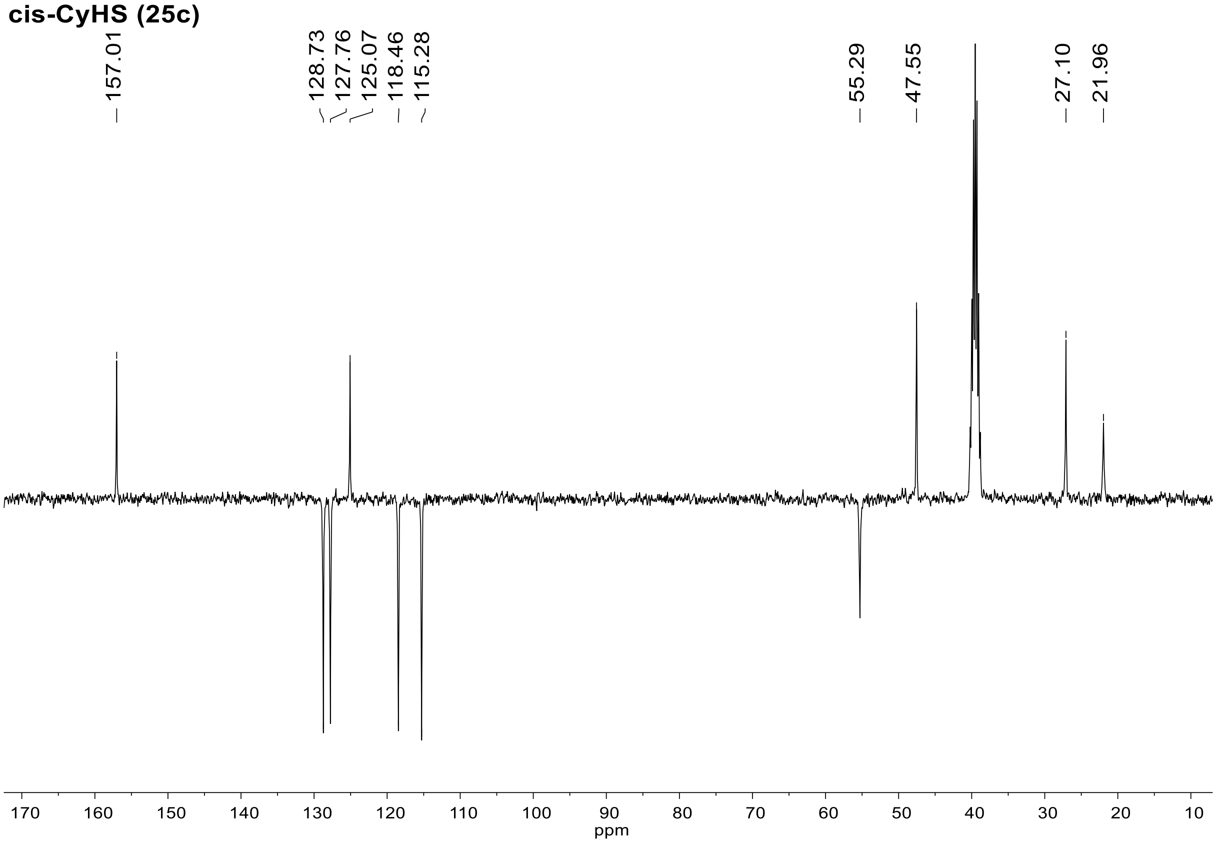

Figure S104. $^{13}C\{^{1}H\}$ NMR spectrum of ***cis*-CyHS (25c)** (90 MHz, 298K, d^6-dmso)

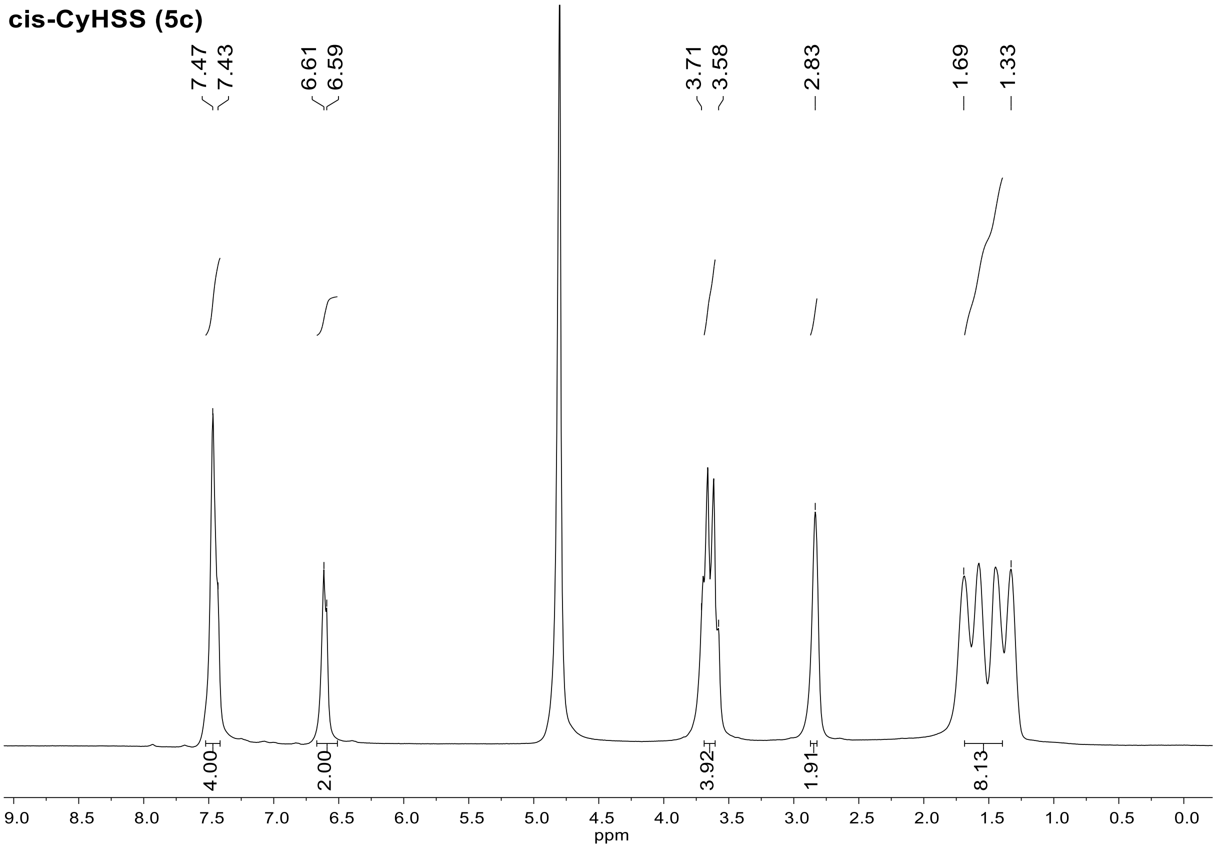

Figure S105. ^{1}H NMR spectrum of ***cis*-CyHSS (5c)** (360 MHz, 298K, D_2O)

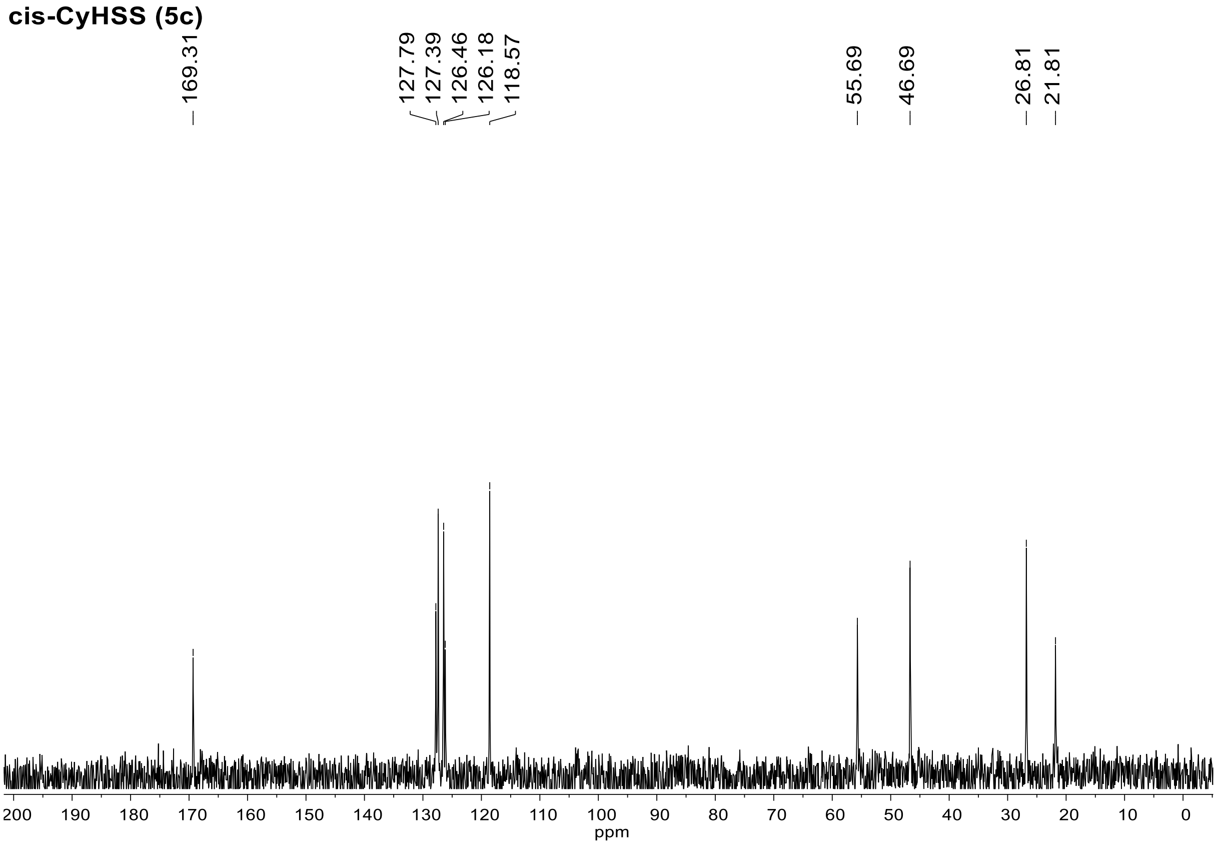

Figure S106. $^{13}C\{^{1}H\}$ NMR spectrum of ***cis*-CyHSS (5c)** (90 MHz, 298K, D_2O)

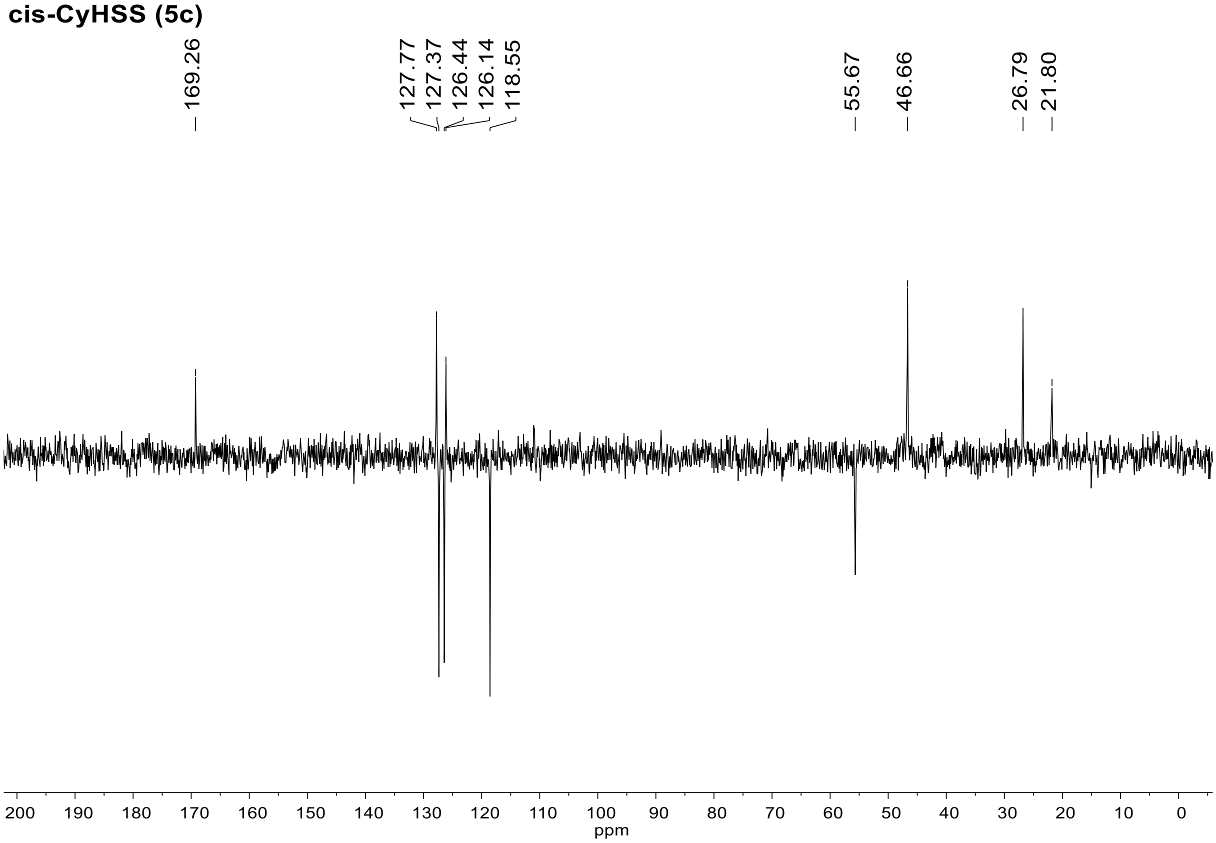

Figure S107. $^{13}C\{^{1}H\}$ NMR spectrum of ***cis*-CyHSS (5c)** (90 MHz, 298K, D_2O)

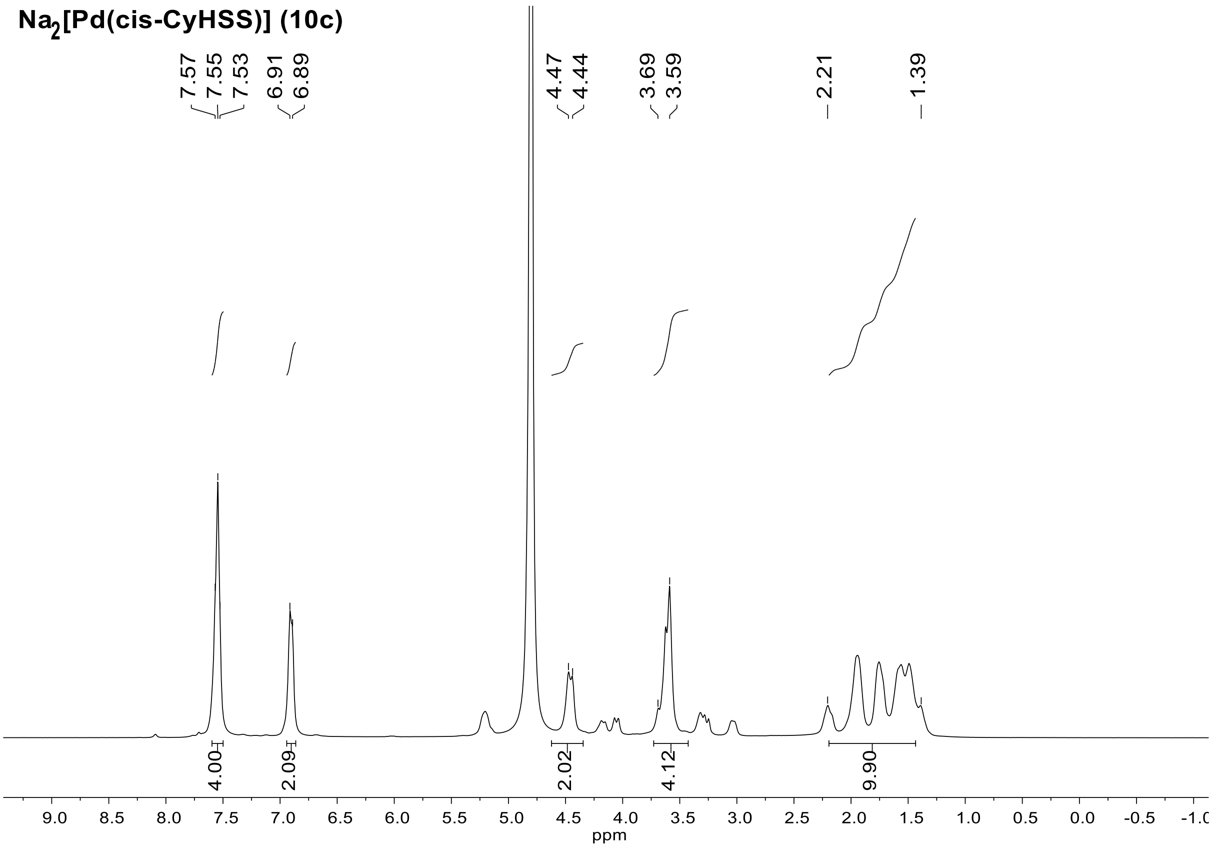

Figure S108.A. ^{1}H NMR spectrum of **Na_2[Pd(*cis*-CyHSS)] (10c)** (360 MHz, 298K, D_2O)

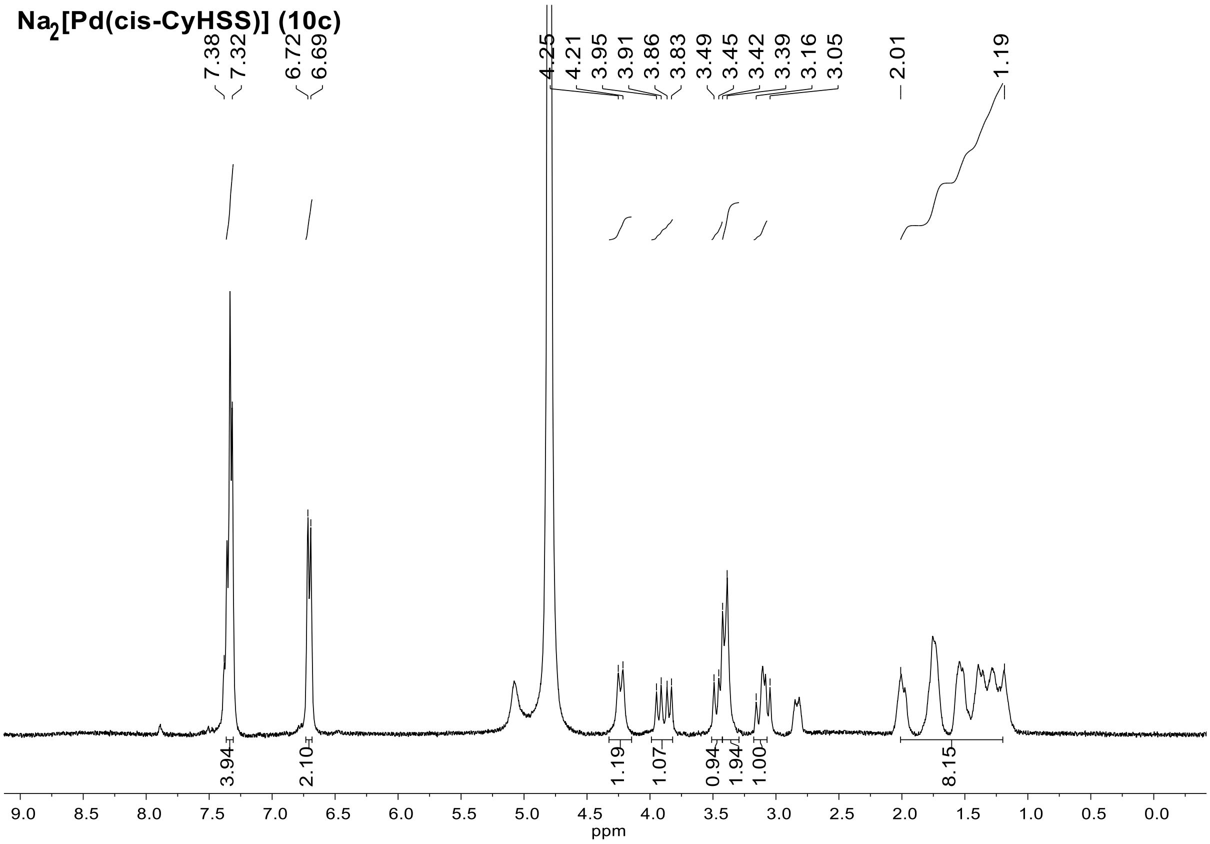

Figure S108.B. ^{1}H NMR spectrum of **Na_2[Pd(*cis*-CyHSS)] (10c)** (360 MHz, 268K, D_2O+CD_3OD)

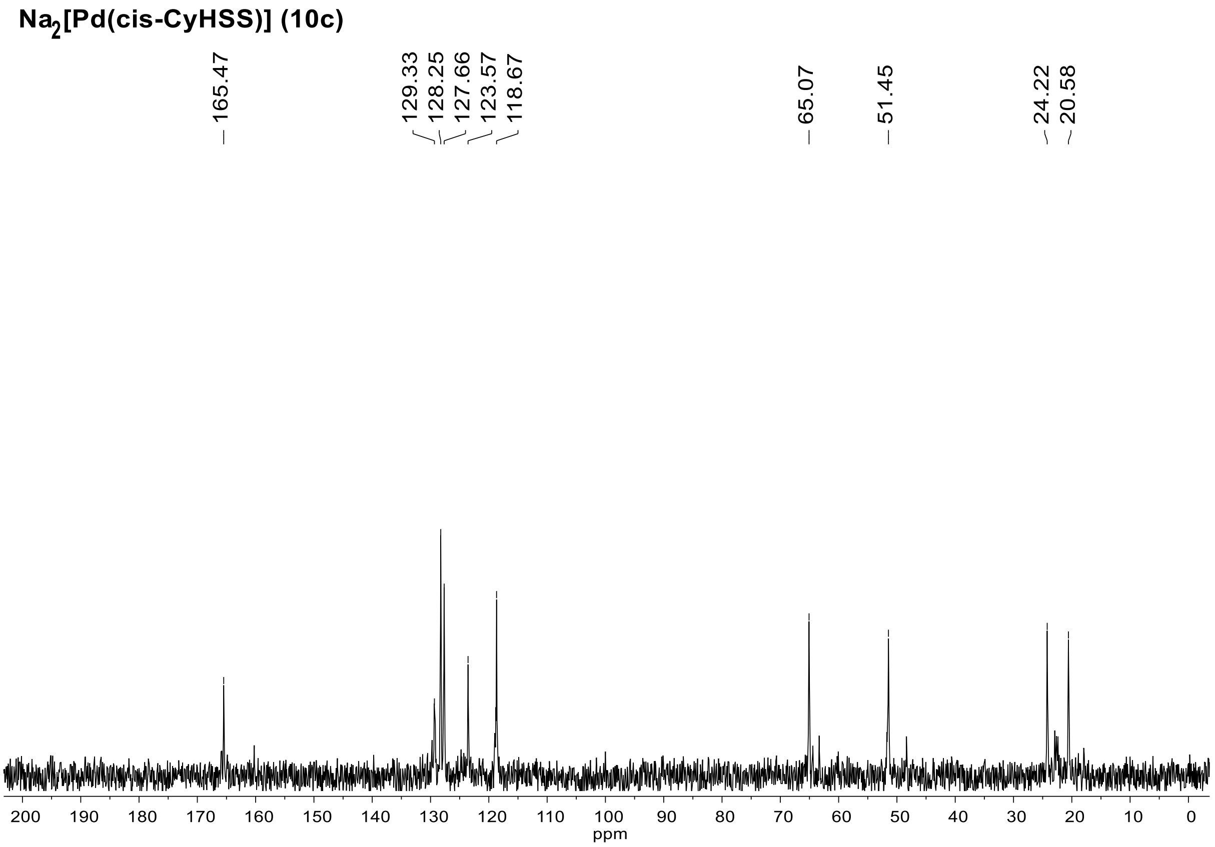

Figure S109. $^{13}C\{^{1}H\}$ NMR spectrum of **Na$_2$[Pd(*cis*-CyHSS)] (10c)** (90 MHz, 298K, D_2O)

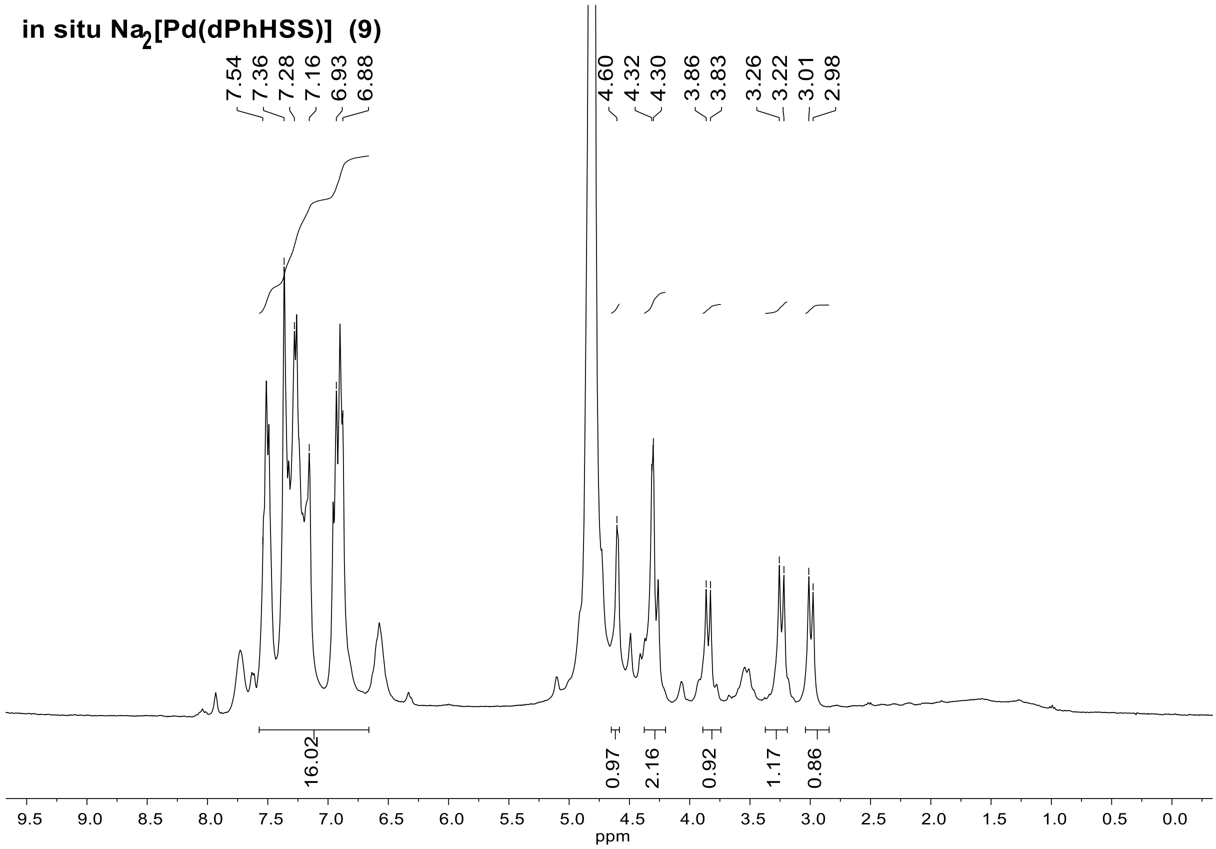

Figure S110. ^{1}H NMR spectrum of in situ prepared **Na_2[Pd(dPhHSS)] (9)** (360 MHz, 298K, D_2O)

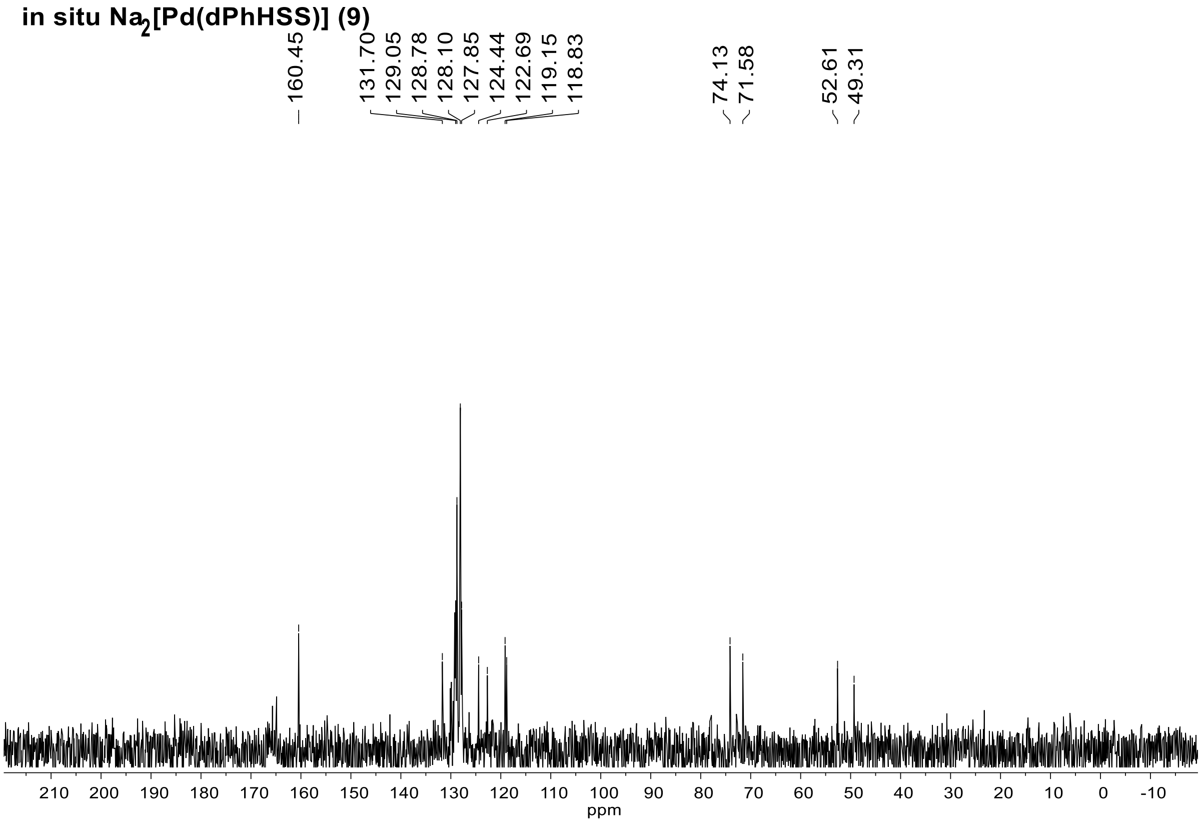

Figure S111. $^{13}C\{^{1}H\}$ NMR spectrum of in situ prepared **Na_2[Pd(dPhHSS)] (9)** (90 MHz, 298K, D_2O)
